# *De Novo* Hybrid Assembly of the *Salvia miltiorrhiza* Mitochondrial Genome Provides the First Evidence of the Multi-Chromosomal Mitochondrial DNA Structure of *Salvia* Species

**DOI:** 10.3390/ijms232214267

**Published:** 2022-11-17

**Authors:** Heyu Yang, Haimei Chen, Yang Ni, Jingling Li, Yisha Cai, Binxin Ma, Jing Yu, Jiehua Wang, Chang Liu

**Affiliations:** 1 School of Environmental Science and Engineering, Tianjin University, Tianjin 300072, China; 2 Institute of Medicinal Plant Development, Chinese Academy of Medical Sciences, Peking Union Medical College, Beijing 100193, China

**Keywords:** *Salvia miltiorrhiza*, lamiales, mitogenome, multi-chromosomal structure, MTPT

## Abstract

*Salvia miltiorrhiza* has been an economically important medicinal plant. Previously, an *S. miltiorrhiza* mitochondrial genome (mitogenome) assembled from Illumina short reads, appearing to be a single circular molecule, has been published. Based on the recent reports on the plant mitogenome structure, we suspected that this conformation does not accurately represent the complexity of the *S. miltiorrhiza* mitogenome. In the current study, we assembled the mitogenome of *S. miltiorrhiza* using the PacBio and Illumina sequencing technologies. The primary structure of the mitogenome contained two mitochondrial chromosomes (MC1 and MC2), which corresponded to two major conformations, namely, Mac1 and Mac2, respectively. Using two approaches, including (1) long reads mapping and (2) polymerase chain reaction amplification followed by Sanger sequencing, we observed nine repeats that can mediate recombination. We predicted 55 genes, including 33 mitochondrial protein-coding genes (PCGs), 3 rRNA genes, and 19 tRNA genes. Repeat analysis identified 112 microsatellite repeats and 3 long-tandem repeats. Phylogenetic analysis using the 26 shared PCGs resulted in a tree that was congruent with the phylogeny of Lamiales species in the APG IV system. The analysis of mitochondrial plastid DNA (MTPT) identified 16 MTPTs in the mitogenome. Moreover, the analysis of nucleotide substitution rates in Lamiales showed that the genes *atp4*, *ccmB*, *ccmFc*, and *mttB* might have been positively selected. The results lay the foundation for future studies on the evolution of the *Salvia* mitogenome and the molecular breeding of *S. miltiorrhiza*.

## 1. Introduction

*Salvia miltiorrhiza* (Danshen) is among the most economically important medicinal plants, and its products have been widely used for centuries to treat various human diseases, such as cardiovascular disease, dysmenorrhea, and amenorrhea [1]. The annual production of this species reaches 20,000 tons [2], and there have been 869 formulas containing *S. miltiorrhiza* that were stored in the Encyclopedia of Traditional Chinese Medicine (ETCM) database [3]. As a result, molecular breeding and synthetic biology studies have been conducted extensively to improve the yield of *S. miltiorrhiza* materials. Several nuclear genome sequences of *S. miltiorrhiza* have been reported [4,5,6,7]. In addition, a mitochondrial genome (mitogenome) has been reported before [8,9]. The mitogenome is a single circular molecule based on short reads generated using the next-generation DNA sequencing technologies. In numerous plant mitochondrial genomes, one or more pairs of repeats can act as inter- or intramolecular recombination sites and generate multiple alternative arrangements (isoforms) [10]. As a result, a previously reported mitogenome cannot capture the complete spectrum of isoforms resulting from repeat-mediated recombination.

By September 2022, 499 complete plant mitogenomes have been released in the GenBank [11]. These plant mitogenomes vary considerably in terms of genome size and architecture, gene composition, RNA editing potential, mutation rates of the protein-coding genes (PCGs), and the rate of recombination across different types of repeats [12,13,14,15,16,17]. The available mitogenomes will aid in the reconstruction of the ancestral angiosperm mitogenome and the understanding of its subsequent evolutionary changes [18,19], which lead to the currently extraordinary diversity of mitogenomes [20,21].

Analyses of the available mitogenomes revealed significant variations in the genes, introns, and RNA-editing capacities of plant mitogenomes [22,23]. Although the mitogenome of *Viscum* contains only 19 PCGs, the mitogenome of the liverwort genera comprises 39–42 PCGs [13,24]. For RNA-editing events, 427 and 441 C-to-U RNA-editing sites were found in the open reading frames of the mitogenomes of rapeseed and *Arabidopsis thaliana*, respectively, and 225 C-to-U RNA-editing sites were identified in the PCG regions of the *S. miltiorrhiza* mitogenome [9,23].

Research of the plant mitogenomes also focused on the intricate structure and revealed significant inter-specific variations in the structure of plant mitochondria [25]. The mitogenome of *Arabidopsis thaliana* was assembled as a standard and single circular structure [26], whereas, in another species (*Silene conica*), the mitogenome showed complex multichromosomal structures [15]. Multichromosomal mitochondrial genomes have also been reported in numerous plants, including cucumber (*Cucumis sativus*) [17], which harbors three circular chromosomes (1556, 84, and 45 Kb). Recently, the multichromosomal structure has also been observed in the mitochondrial genome of onion, and two circular chromosomes have been obtained (173,131 and 143,157 bp) [27].

*S. miltiorrhiza* is a member of the order Lamiales. This enormous angiosperm order is a member of the asterid clade and contains nearly 23,810 species, 1059 genera, and 24 families. Most species of this order, such as sesame, olive, jasmine, psyllium, and lavender, are known for their essential economical value [28,29]. However, the available mitogenomes from Lamiales plant lineages are limited, preventing the in-depth understanding of the mitogenome evolution in this group. The mitogenomes of only nine Lamiales plants, namely, *Ajuga reptans* (NC_023103.1) [30], *Castilleja paramensis* (NC_031806.1), *Boea hygrometrica* (NC_016741.1) [31], *Erythranthe lutea* (NC_018041.1) [20], *Hesperelaea palmeri* (NC_031323.1) [32], *Salvia miltiorrhiza* (NC_023209.1), *Utricularia reniformis* [33], *Rotheca serrata* (NC_049064.1), and *Scutellaria tsinyunensis* (MW553042.1) [34], have been reported.

The *S. miltiorrhiza* mitogenome has been reported as a single circular chromosome using the Illumina sequencing reads alone [9]. Here, we assembled the *S. miltiorrhiza* mitogenome based on the combination of short reads generated from Illumina technology and long reads from PacBio technology. The newly assembled mitogenome showed significantly different arrangements. The results suggest that the dominant form of the *S. miltiorrhiza* mitogenome contains two subgenomic chromosomes, and nine pairs of repeat sequences can mediate recombination, leading to a large collection of minor conformations.

## 2. Results

### 2.1. Structure Analysis of the S. miltiorrhiza Mitogenome

**Figure 1:**
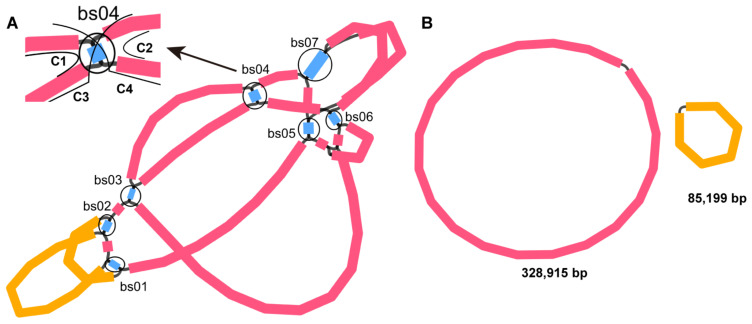
Schematic of the assembly steps of the *S. miltiorrhiza* mitogenome. (**A**) Unitig graph of the *S. miltiorrhiza* mitogenome was obtained from de novo assembly of Illumina reads with Unicycler. The unitig graph contained seven contigs (blue) that formed DBSs (bs01–07, black circle). Each DBS has four conformations (C1, C2, C3, and C4), as shown in the top left corner with bs04, as an example. (**B**) Schematic graph of MC1 (pink circle) and MC2 (orange circle) of *S. miltiorrhiza* after the DBSs were resolved by long reads. The contigs shown in pink and yellow correspond to chromosomes 1 and 2, respectively.

In total, 19 and 16 Gb of raw sequence data were generated from the PacBio RS (Menlo Pask, CA, USA) and Illumina (San Diego, CA, USA) platforms, respectively (Table S1). We extracted the mitochondrial short reads using GetOrganelle and conducted de novo assembly of the extracted reads using the Unicycler software [35], resulting in a unitig graph (Figure 1A). The unitig graph contained seven double bifurcating structures (DBS) (bs01–07). Each DBS has conformations 1, 2, 3, and 4 (C1, C2, C3, and C4). We used Unicycler software to resolve the DBSs. Unicycler mapped the long reads to the DBSs’ four conformations and identified those that were supported by more reads. These conformations were then used in the final assembly. The results of the Unicycler analysis were then loaded into the Bandage software. By using the “Merge all possible nodes” module of the Bandage software, we finally obtained two chromosomes of the mitogenome of *S. miltiorrhiza* (Figure 1B).

In parallel, we assembled the plastome sequence of *S. miltiorrhiza* using GetOrganelle. The size of the plastome was 151,394 bp, which is close to the size of the published chloroplast genome (151,328 bp) [36]. We compared the plastome sequences obtained here and the one published before and discovered their high similarity (Figure S1). The plastome sequence assembled from this study is provided in Supplementary File S1.

To confirm that the DBS conformations selected by Unicycler were supported by most long reads, we constructed sequences corresponding to the four conformations of bs01–07 (Supplementary File S2). We mapped the long PacBio reads to these sequences. Figure S2a–g show the mapping results. We also counted the reads mapped to each of the four conformations, and the results are shown in Table 1. We denoted the conformations found in the mitogenome assembly as major conformations (Mac1 and Mac2) and those not found in the mitogenome assembly as minor conformations (Mic1 and Mic2).

To validate the assembly result, we first mapped the long reads to the mitogenome sequences and obtained average coverage depths of 334.04 and 288.20 for mitogenome chromosomes 1 and 2 (MC1 and MC2), respectively (Figure S3a,b). The entire chromosomes were covered well by the reads, with the lowest coverage being 257. In parallel, we mapped the short reads to the mitogenome sequences, obtaining average coverage depths of 965.88 and 1153.56 for MC1 and MC2, respectively (Figure S3c,d).

Previously, an *S. miltiorrhiza* mitogenome assembled from Illumina short reads was published (NC_023209) [9]. We compared the sequences of the MC1 and MC2 obtained in this study with the published ones. The two assemblies of the *S. miltiorrhiza* mitogenome differed significantly (Figure S4). Numerous rearrangements were observed between the two assemblies, and the largest collinear block was 66,778 bp in length. To determine whether the PacBio reads supported NC_023209, we mapped the PacBio reads to the sequence of NC_023209 and discovered numerous regions not covered by PacBio reads, indicating the potential misassembly of this sequence (Figure S5).

### 2.2. Gene Content of S. miltiorrhiza Mitogenome

**Figure 2:**
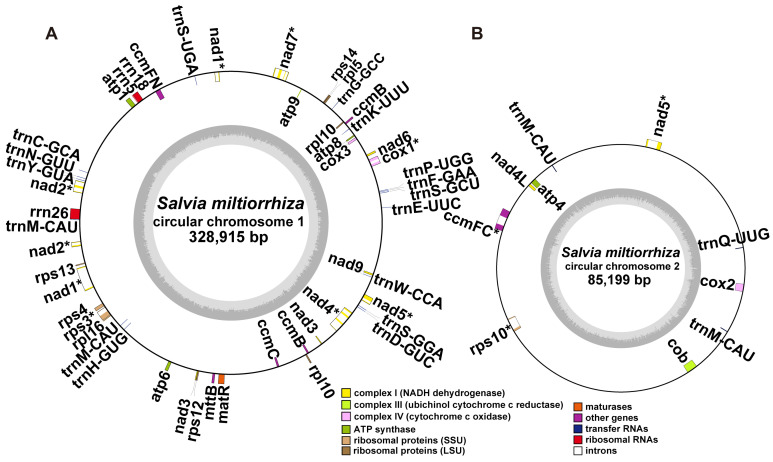
Schematic of the circular MC1 (**A**) and MC2 (**B**) of *S. miltiorrhiza*. The graph was drawn using PMGView (http://www.1kmpg.cn/pmgview (accessed on 26 June 2022)). Genes shown on the inside were on the negative strand, whereas those on the outside were on the positive strand. Genes with introns were highlighted using “*”. The gray circle represents the GC contents. The circle inside the GC content graph marks the 50% threshold. The colors indicate different functional categories shown in the legend.

**Figure 3:**
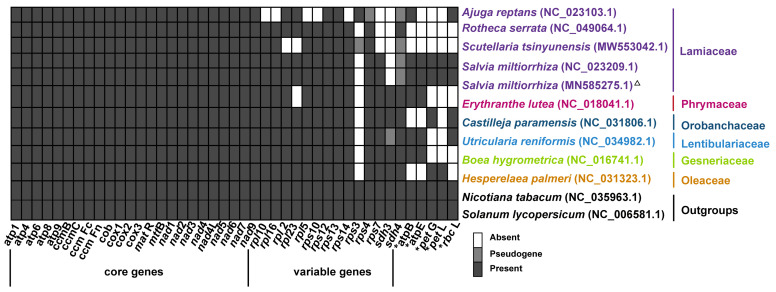
PCG contents in nine sequenced Lamiales mitogenomes. The X-axis shows the name of core and variable genes. Five PCGs, namely, *atpB*, *atpE*, *petG*, *petL*, and *rbcL*, were similar to the sequences from plastomes (labeled with “*”). The mitogenome generated in this study was labeled with the triangle. The species belonging to *Lamiaceae*, *Phrymaceae*, *Lentibulariaceae*, *Gesneriaceae*, and *Oleaceae* were labeled with the colors of purple, peach, dark green, blue, green, and orange, respectively.

The length of the mitogenome chromosome 1 and 2 (MC1 and MC2) was 328,915 bp and 85,199 bp, respectively, and the GC content was 44.62% and 44.43%, respectively. The mitogenome of *S. miltiorrhiza* contained a total of 33 PCGs, 3 rRNA genes (*rrn5*, *rrn18*, and *rrn26*), and 19 tRNA genes (Figure 2, Table 2). In angiosperm mitogenomes, there are a set of 24 core protein-coding genes mostly coding respiratory proteins and 17 variable protein genes coding ribosomal proteins [37]. The *S. miltiorrhiza* mitogenome included the entire set of 24 core PCGs and 9 out of 17 variable PCGs (Figure 3 and Table 2). The DNA sequences of two chromosomes of the mitogenome of *S. miltiorrhiza* are provided as fasta files, along with the annotation information provided as Genbank files (MN585275.1 and MN585276.1) and also deposited in the Figshare repository (https://doi.org/10.6084/m9.figshare.21195841 (accessed on 28 June 2022)).

Five (*cox1*, *rps3*, *cox2*, *ccmFc*, and *rps10*), two (*nad4* and *nad7*), and three (*nad1*, *nad2*, and *nad5*) PCGs contained two, four, and five exons, respectively. The remaining 23 PCGs had no intron. We counted the gene contents from 10 Lamiales mitogenomes and observed the absence of *sdh3* in the mitogenomes of Lamiaceae (Figure 3). The implication of this observation remains to be determined.

### 2.3. Repeat Sequences in the Lamiales Mitogenomes

Repeat sequences make up a large proportion of eukaryotic genomes; they play important roles in genome evolution and have been used widely as molecular markers for discrimination at the subspecies levels [38]. Simple sequence repeats (SSRs), also called microsatellite repeats, have been considered effective molecular markers due to their high variability in the whole plasmid. Thus, they can provide useful information for phylogenetic and population genetic studies [39]. By contrast, tandem repeats consist of multiple copies of repeat units (≥5 nucleotides) that are adjacent to one another.

To explore the potential roles of repeat sequences, we analyzed two types of repeat sequences in the mitogenome of *S. miltiorrhiza* and another nine species of Lamiales. We detected 112 SSRs, including 99 SSRs on MC1 and 13 on MC2. Among them, 35 (35.35%) SSRs in MC1 were tetra-nucleotide repeats, and 27 (27.27%), 16 (16.16%), 13 (13.13%), 4 (4.04%), and 4 (4.04%) were di-, tri-, mono-, penta-, and hexanucleotide repeats, respectively (Tables S2 and S3). In MC2, tetra- and tri-nucleotide repeats were the most abundant, with a ratio of 30.77% (4). The SSRs of tetranucleotide dominated *S. miltiorrhiza* (Table S3).

By contrast, one and two tandem repeat sequences were found in MC1 and MC2 of the *S. miltiorrhiza* mitogenome, respectively. These three repeats had lengths between 15 and 30 bp (Table S4). The two types of repeats had also been detected in the other nine Lamiales mitogenomes (Tables S3 and S4). In addition, the numbers of tandem repeats were not correlated with the genome size of the nine Lamiales mitogenomes and differed from the number of SSRs repeats that were positively correlated with the genome size in most of the nine Lamiales species (Tables S3 and S4).

### 2.4. Homologous Recombination Mediated by Repeats

**Figure 4:**
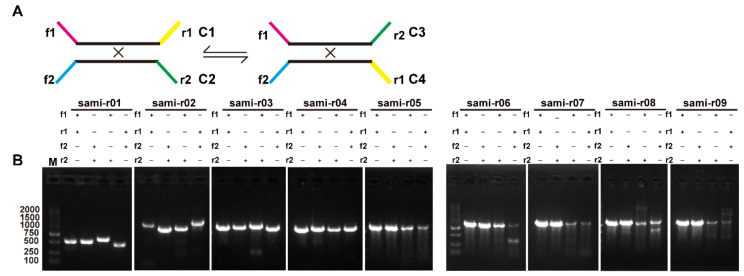
Verification of recombination products associated with the repeats on MC1 and MC2. (**A**) Schematic of the DBSs and their corresponding Mac and alternative conformations. The blank lines represent the two repeat units. The flanking sequences are shown in purple, yellow, green, and blue. The Mac were those observed in our genome assembly. (**B**) PCR amplification of products representing the Mac and Mic conformations. The primer combinations for r01–09 are shown. The Sanger sequencing results of the PCR products are displayed in Figure S6.

To explore the potential subgenomic structures of the *S. miltiorrhiza* mitogenome, we identified the repeats in the MC1 and MC2 using the BLASTN program with a cutoff of E-value = 10^−6^ and a word size = 7 [40]. We identified 72 high scoring pairs (HSPs) in total. For simplicity, we used HSPs and repeats interchangeably in the following text. To determine whether these repeats can mediate recombination, we extracted the sequences containing the repeats themselves and 500 bp sequences upstream and downstream of the repeats. We then switched the flanking regions to generate alternative conformations. These created sequences corresponded to four conformations, namely, C1, C2, C3, and C4 (Figure 4A). The sequences corresponding to these conformations are provided in Supplementary File S2. Here, C1 and C2 were found in the genome assembly. C3 and C4 were created by switching the flanking sequences of C1 and C2, respectively. C1 and C2 sequences were reverse-complementary to each other, and the same condition applied to C3 and C4 sequences. We mapped the long reads to the sequences of the four conformations. Nine pairs of repeats were likely associated with the homologous recombination based on the mapping result of the long reads (Table 1, highlighted with “^a^”). Figure S6a–i shows the mapping results.

Among the nine repeats, eight had both repeat subunits on MC1, and one repeat had one repeat unit on MC1 and another repeat unit on MC2. The length of these nine repeats, including six direct and three inverted repeats, ranged from 59 bp (r09) to 682 bp (r02). The recombination frequency was calculated by the numbers of long reads mapped to the Mic divided by those mapped to all conformations. The percentages of recombination products associated with the nine repeat sequences were as follows: r05 (2.34%), r04 (4.65%), r07 (6.94%), r01 (7.54%), r06 (9.48%), r09 (11.8%), r03 (13.79%), r08 (18.42%), and r02 (21.33%), in ascending order (Table 1).

To further validate the presence of homologous recombination products identified by long-read mapping, we designed primers (Table S5) to amplify the sequences corresponding to the four conformations. The polymerase chain reaction (PCR) products of the primer pairs F1 + R1 and F2 + R2 supported the presence of C1 and C2 conformations. By contrast, the PCR products of the primer pairs F1 + R2 and F2 + R1 supported the presence of C3 and C4 conformations, respectively (Figure 4A). Figure 4B shows the gel electrophoresis results of the PCR products. The observed band patterns of PCR products were consistent with the expected results. The PCR products were then subjected to Sanger sequencing. The Sanger sequencing results of the PCR products were aligned with the expected sequences, and they were mostly identical (Figure S7a–i).

We compared the contig sequences corresponding to the shared nodes of DBSs in the unitig graph. The contig sequences of the shared nodes of bs02, bs03, bs04, bs05, and bs06 were the same as r01, r05, r03, r02, and r04, respectively (Table 1). The HSPs r11 and r10 were the same as the shared contig sequences of bs01 and bs07, respectively. However, the four conformations were not verified successfully by PCR and Sanger sequencing experiments. The examination of their sequences showed that r11 was associated with very low recombination frequencies, and r10 was too long to be amplified by PCR (Table 1).

**Figure 5:**
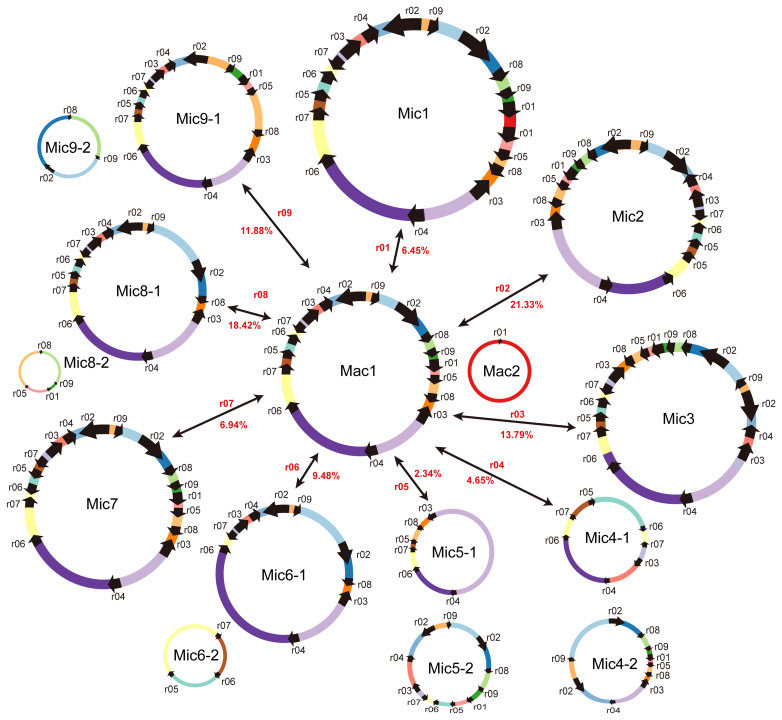
Potential conformations resulting from the recombination mediated by nine repeats (sami-r01 to sami-09) in the *S. miltiorrhiza* mitogenome. The black arrows on the circular molecules represent the repeat unit sequences, and the colored lines denote the DNA fragments between repeats. Mac and Mic represent the major and minor conformations resulting from the rearrangement, respectively. The dominant conformations included Mac1 and Mac2. Mic1 resulted from the sami-r01-mediated recombination with a recombination frequency of 6.45% and contained one circular molecule. Mic2, 3, and 7 resulted from r02, r03, and r07-mediated recombination from Mac1, respectively, with recombination frequencies of 21.33%, 13.79%, and 6.94%. They all contained one circular molecule. Mic4, 5, 6, 8, and 9 resulted from r04, r05, r06, sami-r08, and sami-r09-mediated recombination from Mac1, respectively, and their recombination frequencies were 4.65%, 2.34%, 9.48%, 18.42%, and 11.88%. They all split Mac1 into two Mic. r01 is an inter-molecular repeat and causes inter-chromosomal recombination. On the contrary, r02, r03, r04, r05, r06, and r07 lead to intra-chromosomal recombination.

To determine the number of conformations possibly contained by the mitogenome, we defined MC1 and MC2 as Mac1 and Mac2, respectively. We then inferred the potential Mic generated by nine r01–r09 (Figure 5). Mic2, Mic3, and Mic7 resulted from the recombination mediated by r02, r03, and r07 from Mac1, respectively. Their recombination frequencies were 21.33% (Mic2), 13.79% (Mic3), and 6.94% (Mic7), and they contained the rearranged structure of Mac1. The r04, r05, r06, r08, and r09 can split Mac1 into two Mic (Mic4-1, Mic4-2, Mic5-1, Mic5-2, Mic6-1, Mic6-2, Mic8-1, Mic8-2, Mic9-1, and Mic9-2), with the recombination frequencies of 4.65%, 2.34%, 9.48%, 18.42%, and 11.88%, respectively (Figure 5).

### 2.5. Identification of Mitochondrial Plastid Sequences (MTPTs)

**Figure 6:**
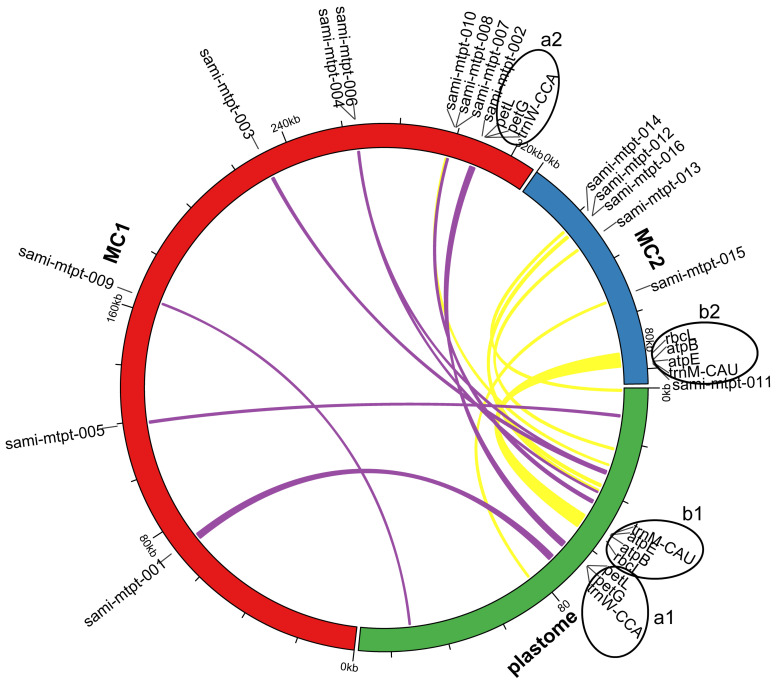
Circular map showing the distribution of MTPT on MC1 and MC2 of the *S. miltiorrhiza* mitogenome. The red and blue lines of the outer circle represent the MC1 and MC2 of the *S. miltiorrhiza* mitogenome, respectively. The green line of the outer circle is the plastome of *S. miltiorrhiza*. The purple and yellow links in the inner circle represent MTPT on MC1 and MC2 of the *S. miltiorrhiza* mitogenome, respectively. a1 and a2 denote the gene cluster of the MTPT fragment sami-mtpt-002 located on MC1 and plastome, respectively. b1 and b2 represent the gene cluster of the MTPT fragment of sami-mtpt-011 located on MC2 and plastome, respectively.

Mitochondrial plastid DNAs (MTPTs) are plastid-derived DNA fragments in mitochondrial genomes [41]. Sixteen fragments similar to the plastome were identified in the mitogenome of *S. miltiorrhiza*. Figure 6 shows their locations on the plastome and mitogenome. Table S6 provides detailed information for these MTPT fragments in terms of the percentage of match, start and end positions, gene contents, etc.

To determine whether these MTPTs were present in the mitogenome rather than the assembly artifact, we searched for PacBio long reads that can be mapped to these MTPT fragments. We identified numerous PacBio long reads that can cover the 16 MTPT fragments and their flanking of 2,000 bp regions in the mitogenome (Figure S8a–p). We obtained screenshots in Tablet to visualize the alignment of long PacBio reads to sami-mtpt-001 to sami-mtpt-016 (Figure S8a–p, respectively). These long reads confirmed the occurrence of MTPT events in the mitogenome of *S. miltiorrhiza*.

These MTPTs had a total size of 12,583 bp, representing approximately 3.04% of the total length of the *S. miltiorrhiza* mitogenome and 8.31% of the plastome. The longest MTPT fragment (sami-mtpt-012) was observed from positions 73,481 to 78,467 on MC2, and it contained the full coding sequences of rbcL, atpB, atpE, and trnM-CAU. The second-longest MTPT (sami-mtpt-001) was from base 70,229 to base 72,166 of MC1 and contained a fragment of the chloroplast gene psbB. The third-longest MTPT (sami-mtpt-002) was from base 308,153 to base 309,889 and included the full coding sequences of the chloroplast genes petG, petL, and trnW-CCA, which were identified in the MC1 region (Figure 6 and Table S6).

### 2.6. Phylogenetic Analysis

**Figure 7:**
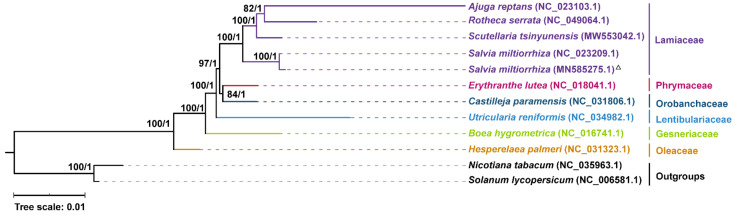
Molecular phylogenetic analysis of mitogenomes in Lamiales. The tree was constructed using concatenated conserved protein sequences from the mitogenomes of nine species via the ML and BI methods. The bootstrap score was obtained using 1000 replicates. The ML bootstrap support values and BI posterior probabilities were labeled at the corresponding nodes. Two species from Solanaceae (*Nicotiana tabacum* and *Solanum lycopersicum*) were used as outgroups. The mitogenome assembled in this study was labeled with a triangle. The species belonging to *Lamiaceae*, *Phrymaceae*, *Lentibulariaceae*, *Gesneriaceae*, *Oleaceae* were labeled with the colors of purple, peach, dark green, blue, green, and orange, respectively.

To determine the phylogenetic relationship of ten Lamiales mitogenomes, we constructed a phylogenetic tree using 24 core mitochondrial PCGs (*atp1*, *atp4*, *atp6*, *atp8*, *atp9*, *ccmB*, *ccmC*, *ccmFc*, *ccmFn*, *cob*, *cox1*, *cox2*, *cox3*, *matR*, *mttB*, *nad1*, *nad2*, *nad3*, *nad4*, *nad4L*, *nad5*, *nad6*, *nad7,* and *nad*9) and 2 variable genes (*rps12* and *rps13*) from these 10 mitogenome sequences. The mitogenomes of *Solanum lycopersicum* and *Nicotiana tabacum* were used as outgroups. The ten mitogenome sequences of Lamiales were from the following families: Lamiaceae, Phrymaceae, Orobanchaceae, Lentibulariaceae, Gesneriaceae, and Oleaceae. Phylogenetic trees were constructed using the maximum likelihood (ML) and Bayesian inference (BI) methods. Most nodes on the phylogenetic tree had bootstrap support values >90 and posterior probabilities = 1, indicating the strong reliability of the phylogenetic relationship of the nine Lamiales species (Figure 7). The topological structure of the tree is identical to the phylogeny of Lamiales species in the APG IV system [42] and the results from a previous study [34]. In particular, although the organization of our genome assembly and the previously reported one (NC_023209.1) differed significantly (Figure S4), their protein sequences are highly conserved.

### 2.7. Identification of Genes under Selection

**Figure 8:**
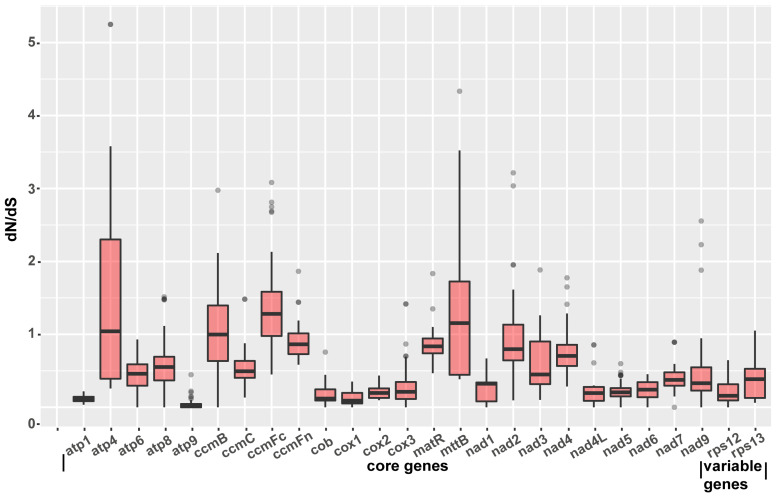
Molecular phylogenetic analysis of mitogenomes in Lamiales. The highest and lowest dot represented the maximum and minimum of the pairwise dN/dS values of each gene. The upper, medium, and lower lines in the box represented the upper quartile, median, and lower quartile of the pairwise dN/dS values of each gene, repsectively.

To determine variations in nucleotide substitution rates in the mitogenome of *S. miltiorrhiza* and the other nine mitogenome sequences of Lamiales, we calculated the pairwise non-synonymous substitution rate (dN), the synonymous substitution rate (dS), and the ratio of dN to dS of the 26 shared mitochondrial genes using the yn00 module of PAML (v4.9) [43]. The genes *atp*4, *ccm*B, *ccm*Fc, and *mtt*B showed that the dN/dS ratios were over 1.0 in most species, indicating a possible positive selection (Figure 8 and Figures S9 and S10, Supplementary File S3). Most of the genes, such as *atp1*, *atp9*, *cob*, *cox1*, *cox2*, *cox3*, *nad1*, *nad4L*, *nad5*, *nad6*, and *rps12*, showed low dN/dS ratios, implying a possible negative selection. The *atp1* and *atp9* genes had a prominently low dN/dS ratio compared with those of other PCGs, suggesting that they may be functionally highly conserved.

## 3. Discussion

### 3.1. Overview of the S. miltiorrhiza Mitogenome

In this study, we achieved the following: (1) obtained a high-quality mitogenome of *S. miltiorrhiza* using a hybrid assembly strategy; (2) annotated the *S. miltiorrhiza* mitogenome and predicted its gene contents; (3) analyzed repeat elements; (4) predicted and validated the homologous recombination mediated by repeats; (5) identified the MTPTs between the plastome and mitogenome; (6) constructed phylogenetic trees with the PCG sequences; (7) calculated the substitution rates of mitochondrial PCGs. The detailed characterization of the high-quality assembly of the *S. miltiorrhiza* mitogenome may serve as the foundation for future studies on the genomic evolution of this important medicinal plant.

We compared the sequences of the *S. miltiorrhiza* mitogenome obtained in this study with the published one (NC_023209) [9] and observed that the two assemblies significantly differed in terms of structure with a number of rearrangements. The mapping of long reads strongly supported the assembly of this study. The structural differences between these assemblies may be due to the following reasons. First, large intra-specific variations may exist in mitogenome structures. Second, the use of different sequencing technologies and assembly algorithms may generate various mitogenome structures.

### 3.2. Repeat Mediated Homologous Recombination in Lamiaceae

Plant mitogenomes are a complex and dynamic mixture of forms rather than a single circle [44]. Previously, a single circular *S. miltiorrhiza* mitogenome was released. In the present study, we used Illumina and PacBio reads to investigate the diverse mitogenome structures. We observed that the *S. miltiorrhiza* mitogenome consists of two major conformations, Mac1 and Mac2, and multiple minor conformations, which resulted from the recombination mediated by nine repeats.

These findings were similar to those found in the mitogenomes of *Silene* [15], cucumber [17], sugarcane [45], *Lactuca* [10], rice [46], onion [27], and *Solanum tuberosum* [47]. In a previous report, the mitogenome of *Scutellaria tsinyunensis* of the Lamiaceae family consisted of a 175 bp direct repeat shared by two minor circular conformations. Similar to *S. tsinyunensis*, the *S. miltiorrhiza* mitogenome is divided into two direct circular structures by a 127 bp-long forward repeat. In *S. tsinyunensis*, the major confirmation of the mitogenome is a single circle. However, two circular molecules form the major confirmation in the *S. miltiorrhiza* mitogenome. By comparing the two repeat sequences mediating recombination in *S. tsinyunensis* and *S. miltiorrhiza*, we found no sequence similarity between them. The mechanism of repeats mediating recombination in other Lamiaceae species requires support from further experimental evidence.

### 3.3. Current Limitation of the Plant Mitogenome Assembly Method

Several technical limitations may affect the quality of mitogenome assembly. This study assembled mitogenome from total DNAs with a hybrid assembly strategy, combining the unitig graph assembled from short reads and contigs assembled from long reads. This strategy can avoid the false positive caused by the Polish strategy. In addition, the presence of MTPTs and nuclear mitochondrial sequences (NUMTs) in total DNAs may affect genome assembly. We have carefully checked the 16 MTPTs in this study based on the mapping results of MTPTs and regions flanking the MTPTs.

In addition, given their complex structure, mitogenomes may have multiple configurations as a combination of linear, circular, and branched molecules [23]. Several studies of plant mitogenomes were accustomed to using a separate ring molecule to represent the plant mitogenome. Such a representation is inadequate to describe the dynamic and complex structure of mitogenomes. Through cryo-electron microscopy, complex physical structures (circular, linear, and branched) of mtDNA molecules were observed in *Lactuca sativa* [10]. In addition, researchers of plant mitogenomes should identify as many of the Mac of the genome as possible and explore possible forms of recombination based on sequencing results. Bioinformatic predictions must be further validated by quantitative PCR experiments, Sanger sequencing, Southern blot, and electronic microscopy.

### 3.4. Future Studies of the S. miltiorrhiza Mitogenome

Several directions can be pursued to further analyze the structure of the *S. miltiorrhiza* mitogenome. We can analyze the mitogenome structure by isolating mitochondria and DNAs for subsequent analyses with long-read DNA sequencing technology, the diversity and dynamics of *S. miltiorrhiza* mitogenomes at the population level, and the various levels of recombination among mitogenomes from different plants, particularly those that may affect the physical properties of *S. miltiorrhiza*. Finally, the structure of mitogenome DNAs can be examined via electron microscopy to visualize the actual mitogenome structures and confirm their structural diversity. The results obtained from these studies will lay a solid foundation for understanding mitogenome evolution and facilitate mitogenome-based breeding.

## 4. Materials and Methods

### 4.1. Plant Materials and DNA Extraction

Fresh leaves were collected from an inbred line (named sami-il01) of *S. miltiorrhiza* from the Research Center of Medicinal Plants, Shandong Academy of Agricultural Sciences, Shandong, China. *S. miltiorrhiza* is not an endangered or protected species, and, thus, no specific permissions were required for its collection. A voucher specimen was deposited with the accession number sami-001 at the institute. The leaves were stored at -80 °C until the total DNA was extracted using a plant genomic DNA kit (Tiangen Biotech, Beijing, Co., Ltd., Bejing, China). DNA purity was evaluated with electrophoresis using a 1.0% agarose gel, and the DNA concentration was measured using a Nanodrop spectrophotometer 2000 (Thermo Fisher Scientific, Waltham, MA, USA).

### 4.2. DNA Sequencing and Mitogenome Assembly

The DNA samples from sami-il01 were subjected to library construction using the SMRTbell Template kit 1.0 (Pacific Biosciences, Menlo Pask, CA, USA). In particular, 10 µg DNA was sheared with a Covaris gTube at 4500 rpm for 2 min. DNA fragments (12–50 kb) were selected using the BluePippin cassette 0.75% DF Marker S1 high pass (15–20 Kb). A total of 15 cells were sequenced using the PacBio RSII sequencer with the DNA Sequencing Kit 2.0 (Pacific Biosciences, Menlo Park, CA, USA).

The same DNA sample used above was fragmented to 300–500 bp in length, barcoded, and subjected to library construction using the NEBNext^®^ library construction kit [48] (NEB, Ipswich, MA, USA), following the manufacturer’s instructions. The library was sequenced in the PE100 setting using an Illumina HiSeq 2000 (Illumina, San Diego, CA, USA ) sequencer.

The plastome of *S. miltiorrhiza* (sami-il01) was de novo assembled from Illumina reads using GetOrganelle (v1.6.4) with the parameter “-R 15 -k 21,45,65,85,105 -F embplant_pt “. The mitogenome of *S. miltiorrhiza* (sami-il01) was assembled with a hybrid assembly strategy. First, the mitochondrial short reads were extended and achieved by GetOrganelle (v1.6.4). Then, we de novo assembled the GetOrganelle-extended reads into a unitig graph by the SPAdes software embedded in Unicycler (Pacific Biosciences, Menlo Park, CA, USA). Finally, we resolved the DBSs in the unitig graph with PacBio long reads using Unicycler.

To examine the accuracy of the unicycler in resolving the DBSs, we extracted the sequences containing the repeats in DBSs and 1000 bp sequences upstream and downstream of the repeats. We then switched the flanking regions, and these created sequences that corresponded to the four conformations. We mapped the long reads to the sequences using BWA (v0.7.12-r1039) [49] with default parameters.

To further examine the mitogenome assembly, we mapped the PacBio long reads to the plastome and mitogenome sequences using minimap2 (2.17-r941) [50] with the parameter “minimap2 -ax map-pb.” The Illumina short reads were mapped to the mitogenome by BWA (v0.7.12-r1039) [49] with default parameters. The coverage depth was calculated using samtools (v1.3.1) [51]. The collinearities of the *S. miltiorrhiza* mitogenome obtained in this study and the one published before (NC_023209) [9] were examined using Gepard (v1.40). For further examination, we mapped the PacBio long reads to the mitochondrial genome available in GenBank (NC_023209).

### 4.3. Mitogenome Annotation

The Geseq web server [52] and the custom program MGA (http://www.1kmpg.cn/mga (accessed on 18 June 2022)) were used to annotate the mitogenome. The tRNA genes were identified using tRNAscan-SE [53]. The positions of the start and stop codons and intron/exon boundaries were manually corrected using the Apollo program [54]. The circular mitogenome map was visualized using PMGView (http://www.1kmpg.cn/pmgview (accessed on 22 June 2022)). The mitogenome sequences were submitted to GenBank with the accession numbers MN585275.1 and MN585276.1 and were also available at Figshare (https://doi.org/10.6084/m9.figshare.21195841 (accessed on 28 June 2022)).

### 4.4. Repeat Sequence Analysis

SSRs were detected using the MISA web service [55], with the following thresholds: 10 for the number of mononucleotide repeat units, 5 for the number of dinucleotide repeat units, 4 for the number of trinucleotide repeat units, and 3 for the number of tetra-, penta-, and hexanucleotide repeat units. Tandem repeats were analyzed using the Tandem Repeats Finder [56] with parameter settings of 2 for matches and 7 for mismatches and indels. The minimum alignment score and maximum period size were set at 50 and 500, respectively.

### 4.5. Analysis of the Recombination Products

To identify the inter-and intra-molecular recombination mediated by the repeat sequences, we searched for the repeat sequences of MC1 and MC2 with BLASTN using the following parameters: E-value: 1E-6 and word size: 7 [40]. To examine the presence of possible recombination products around the repeats, we first extracted the 500 bp-long sequence segments around the repeats based on the expected sequences before and after recombination. We then mapped PacBio long reads to the extracted sequence segments of the four conformations and counted the repeat-spanning reads.

For the hypothetic recombination products identified by mapping the PacBio long reads, we designed PCR primers using the Primer 3 web service [57]. PCR reactions were performed in 50 µL volumes with 23 µL water, 25 µL 2 × Taq PCR Master Mix, 1 µL of each primer, and 1 µL DNA. PCR reactions were carried out on a Pro-Flex PCR system (Applied Biosystems, Waltham, MA, USA) under the following conditions: denaturation at 94 °C for 2 min, followed by 35 cycles of 94 °C for 30 s, 57 °C for 30 s, 72 °C for 60 s, and 72 °C for 2 min as the final extension. We separated and visualized the PCR products on 1.0% agarose gels. Finally, the PCR amplicons were sequenced using the Sanger method.

### 4.6. Identification of Mitochondrial Plastid Sequences (MTPTs)

MTPTs were identified by a reciprocal comparison strategy using BLASTN (v 2.2.30+), as described previously [58]. The plastome was assembled from Illumina reads using GetOrganelle [59]. The plastome was compared with the mitogenome using BLASTn through the following parameters: e-value: 1e-6 and word size: 7 [40]. BLASTn hits that were shorter than 100 bp were ignored. The MTPT gene cluster was identified in accordance with the shared boundary of continuous genes, as described by Wang et al. [41].

Then, the MTPT gene clusters on the mitogenome were identified, which were defined as a cluster of continuous genes on the plastome without the insertion of any mitochondrial genes. MTPT gene clusters were depicted by a circular map obtained from TBtools (v1.076) [60]. Moreover, to confirm the MTPT events, we extracted the MTPT fragments including 2000 bp sequences on its 5′ and 3′ ends. The PacBio long reads were aligned to these long fragments using BWA-MEM [61]. Tablet software (Zebra Technologies, Lincolnshire, IL, USA) [62] was used to visualize the mapping results.

### 4.7. Phylogenetic Analysis of the Nine Lamiales Species

Nine complete mitochondrial DNA sequences belonging to the order Lamiales were obtained from GenBank. These sequences specifically belonged to the following species: *Ajuga reptans* (NC_023103.1), *Rotheca serrata* (NC_049064.1), *Scutellaria tsinyunensis* (MW553042.1), *Salvia miltiorrhiza* (NC_023209.1), *Erythranthe lutea* (NC_018041.1), *Castilleja paramensis* (NC_031806.1), *Utricularia reniformis* (NC_034982.1), *Boea hygrometrica* (NC_016741.1), and *Hesperelaea palmeri* (NC_031323.1). The mitogenomes of *Nicotiana tabacum* (NC_006581.1) and *Solanum lycopersicum* (NC_035963.1) were used as outgroup taxa.

For phylogenetic analysis, the DNA sequences of the 26 PCGs shared among these ten mitogenomes (two for *S. miltiorrhiza*) were extracted using PhyloSuite (v1.2.1) [63]. The 26 conserved PCGs were *atp1*, *atp4*, *atp6*, *atp8*, *atp9*, *ccmB*, *ccmC*, *ccmFc*, *ccmFn*, *cob*, *cox1*, *cox2*, *cox3*, *matR*, *mttB*, *nad1*, *nad2*, *nad3*, *nad4*, *nad4L*, *nad5*, *nad6*, *nad7*, *nad*9, *rps12*, and *rps13*. These sequences were aligned using MAFFT (v7.450) [64], and a phylogenetic tree was constructed using the alignment and ML method implemented in RAxML (v8.2.4) [65]. The detailed parameters were “raxmlHPC-PTHREADS-SSE3 -f a -N 1000 -m PROTGAMMACPREV -x 551314260 -p 551314260 -o Nicotiana_tabacum, Solanum lycopersicum -T 20.” The significance level for the phylogenetic tree was assessed using bootstrap testing with 1000 replications. We also performed the BI analysis using MrBayes (v3.2.7a) [66] on CIPRES Science Gateway (v3.3) [67]. The best model for the BI analysis was obtained by the jMdoleTest (v2.1.0) [68]. The resulting tree was visualized by iTOL [69].

### 4.8. Estimation of Nucleotide Substitution Rates

We estimated the pairwise non-synonymous substitution rate (dN), the synonymous substitution rate (dS), and the ratio of dN to dS of sequences of the 26 mitogenome protein-coding genes used in the previous analysis. The yn00 module in PAML (v4.9) [43] was used to conduct the estimation with the following parameters: verbose, icode, weighting, and common 3 × 4 = 0, and ndata = 1. The pairwise dN, dS, and dN/dS values were shown by the boxplot, which was plotted by R-package (ggplot2) [70].

## 5. Conclusions

We showed that the *S. miltiorrhiza* mitogenome consists of two circular chromosomes. Recombination mediated by nine repeats can result in a large number of various conformations. The results obtained from this study suggest that multiple chromosomal structures may be more prevalent than previously thought. They can be present in plants whose primary form of mitogenome is a “master circle”. In the future, obtaining complete mitogenome sequences of additional *Salvia* plants will provide insights into the role that homologous recombination plays in the diversification and evolution of mitogenomes.

## Figures and Tables

**Table 1:** Results for mapping PacBio long reads to the four possible conformations associated with eleven HSPs (r01–r11) and their corresponding seven DBSs (bs01–bs07). “MC1/2”: mitogenome chromosome 1/2. ^a^: Validated successfully with PCR; ^b^: all positions are based on MC1, except those marked with MC2. The recombination frequency was calculated as the number of reads mapped to Mic1 and Mic2 divided by that mapped to all found conformations. NA: not applicable.

ID of the HSP	ID of the DBS	Identity (%)	Alignment Length	Numbers of Mismatches	Numbers of Gap Openings	Positions of Repeat Copy 1 ^b^		Positions of Repeat Copy 2		E-Value	Type	Numbers of Long Reads Mapped to Each Conformation				Recombination Frequency (%)
						Start	End	Start	End			Mac1	Mac2	Mic1	Mic2	
r01 ^a^	bs02	100	127	1	1	328,831	328,915	85,115 (MC2)	85199 (MC2)	3.51 × 10^−37^	direct	70	46	5	3	6.45%
r02 ^a^	bs05	99.853	682	1	0	279,954	280,635	235,468	234,787	0	inverted	31	28	5	11	21.33%
r03 ^a^	bs04	100	369	0	0	223,308	223,676	27,513	27,145	0	inverted	28	22	5	3	13.79%
r04 ^a^	bs06	95.312	192	5	4	232,877	233,068	78,659	78,846	1.53 × 10^−80^	direct	24	17	1	1	4.65%
r05 ^a^	bs03	100	87	0	0	185,367	185,453	1433	1519	2.72 × 10^−38^	direct	65	60	2	1	2.34%
r06 ^a^	NA	95.139	144	5	2	191,145	191,287	141,672	141,814	9.49 × 10^−58^	direct	46	59	6	5	9.48%
r07 ^a^	NA	91.176	136	11	1	175,216	175,351	209,605	209,471	5.80 × 10^−45^	inverted	30	37	3	2	6.94%
r08 ^a^	NA	98.649	74	1	0	300,772	300,845	19,641	19714	2.13 × 10^−29^	direct	33	29	11	3	18.42%
r09 ^a^	NA	98.305	59	0	1	311,788	311,846	252,783	252,840	1.67 × 10^−20^	direct	41	48	9	3	11.88%
r10	bs07	99.966	5835	1	1	269,937	275,770	42,128	36,294	0	inverted	15	10	15	12	51.92%
r11	bs01	94.828	116	5	1	27,842	27,957	326,567 (MC2)	326,681 (MC2)	1.54 × 10^−44^	direct	61	65	0	1	0.79%

**Table 2:** Genes predicted in the mitogenome of *S. miltiorrhiza*.

Group of Genes		Name of Genes
Core genes	ATP synthase	*atp1*, *atp4*, *atp6*, *atp8*, *atp9*
	Cytochrome c biogenesis	*ccmB*, *ccmC*, *ccmFc*^a^, *ccmFn*
	Ubichinol cytochrome c reductase	*cob*
	Cytochrome c oxidase	*cox1*^a^, *cox2*^a^, *cox3*
	Maturases	*matR*
	Transport membrane protein	*mttB*
	NADH dehydrogenase	*nad1*^c^, *nad2*^c^, *nad3*, *nad4*^b^, *nad4L*, *nad5*^c^, *nad6*, *nad7*^b^, *nad9*
Variable genes	Ribosomal protein large subunit	*rpl5, rpl10*, *rpl16*
	Ribosomal protein small subunit	*rps3*^a^, *rps4*, *rps10*^a^, *rps12*, *rps13*, *rps14*
rRNA genes	Ribosomal RNAs	*rrn5*, *rrn18, rrn26*
tRNA genes	Transfer RNA	*trnC-GCA*, *trnD-GUC*, *trnE-UUC*, *trnF-GAA*, *trnG-GCC*, *trnH-GUG*, *trnK-UUU*, *trnM-CAU*, *trnM-CAU*, *trnN-GUU*, *trnP-UGG*, *trnS-GCU*, *trnS-GGA*, *trnS-UGA*, *trnW-CCA*, *trnY-GUA*, *trnQ-UUG*, *trnM-CAU*, *trnM-CAU*

Note: “^a^”, “^b^”, and “^c^”: genes with two, four, and five exons, respectively.

## Data Availability

The raw sequencing data from the Illumina and PacBio platforms generated during the current study are available in GenBank. The associated BioProject, BioSample, and SRA numbers and the associated link are PRJNA782861, SAMN23402358, and SRR17041866 for Illumina sequencing reads and SRR17042314 for PacBio sequencing reads. The mitogenome sequences have been released in GenBank (https://www.ncbi.nlm.nih.gov/, (accessed on 28 September 2022)) with the following accession numbers: MN585275.1 and MN585276.1. The DNA sequences of two chromosomes of the mitogenome of *S. miltiorrhiza* are provided as fasta files, along with the annotation information, which is provided as Genbank files and is also available at Figshare (https://doi.org/10.6084/m9.figshare.21195841, (accessed on 28 September 2022)). The plant sample has been stored at the Herbarium of the Institute of Medicinal Plant Development, Beijing, China (voucher numbers: Implad20181026).

## Supplementary Materials

The following supporting information can be downloaded at: https://www.mdpi.com/article/10.3390/ijms232214267/s1, Figure S1: Comparison of the sequences of the plastome assembled in this study and NC_020341.1 of *S. miltiorrhiza*. The X-axis shows the nucleotide sequence of plastome assembled in this study, and the Y-axis displays the nucleotide sequence of NC_020341.1. Figure S2: Alignments of long PacBio reads to the four conformations of the seven DBSs (bs01–bs07) found in the unitig graph. The unitig graph was generated using Unicycler from Illumina reads, which were filtered with GetOrganelle for mitochondrial reads. Mac corresponds to the connection of shared contigs and their flanking contigs found in MC1 and MC2 (Figure 1B). Mic corresponds to the alternative connection of the shared contigs and their flanking contigs that are not found in MC1 and MC2. Panels a–g show Mac1 and Mac2 and Mic1 and Mic2 of bs01–bs07, respectively. The figure in each panel can be divided into the top and bottom parts. The top part shows a bird’s eye view, whereas the bottom part reveals a base-level view. At the bottom part of the figure, the DBS ID, the length of the shared contig, and the conformation name are shown. The boundaries of the shared contig are indicated with red vertical lines. The contigs in the DBS are shown as the red line with arrows at each end. It was labeled as the “repeat region.” Figure S3: Mapping results of PacBio and Illumina reads to the MC1 and MC2 of *S. miltiorrhiza.* Panels a and b show the mapping results of PacBio reads to MC1 and MC2, respectively. Panels c and d show the mapping results of all Illumina reads to MC1 and MC2, respectively. The X- and Y-axis show the nucleotide position and corresponding coverage depth, respectively. Figure S4: Comparison of the two assemblies of the mitogenome of *S. miltiorrhiza*. Comparison of the sequences of MC1 (A), MC2 (B), and NC_023209.1. The X-axis shows the nucleotide sequence of MC1, and the Y-axis presents the nucleotide sequence of NC_023209.1. The largest collinear block between them was 66,778 bp in length and is highlighted with a red circle. Figure S5: Mapping results of PacBio long reads to the mitogenome of *S. miltiorrhiza* in GenBank (NC_023209.1). The X-axis shows the nucleotide position, and the Y-axis reveals the corresponding coverage depth. Three large regions not supported by the long reads in this study are highlighted with red squares. Figure S6: Alignments of long PacBio reads to the Mac and Mic resulted from nine pairs of HSPs (sami-r01 to sami-r09). The nine HSPs were identified using BLASTn with the E-value = 1E-6. The alignment of two HSPs generated a DBS similar to the ones observed in the unitig graph (Figure 1). In the presence of homologous recombination, the DBSs formed by the alignment of HSPs and their flanking sequences will also have four conformations, similar to those shown in Figure 1B. We constructed the sequences corresponding to the four conformations of each DBS for each HSP. We then mapped the long PacBio reads to these sequences. The results are shown in panels a–i for sami-r01 to sami-r09, respectively. We named the conformations with the most supporting reads as Mac1 and Mac2. The reference sequences of Mac1 and Mac2 are reverse-complementary to each other. The alternative conformations were named as Mic1 and Mic2. The figure in each panel can be divided into the top and bottom parts. The top part shows a bird’s eye view, whereas the bottom part reveals a base-level view. At the bottom part of the figure, the HSP ID, the length of HSP, and the conformation name are shown. The boundaries of HSPs are indicated with red vertical lines. The HSPs are shown as the red line with arrows at each end. They were labeled as “repeat region.” Figure S7: Validation of four conformations corresponding to the recombination products mediated by the nine repeats. PCR primers were designed based on the four conformations. The genomic DNAs were then amplified by PCR, and PCR products were subjected to Sanger sequencing. The sequencing chromatogram (top), Sanger sequencing results (labeled with “PCR” and conformation number), expected sequences (labeled with repeat ID and conformation number), and consensus sequences are shown from top to bottom. Panels a–i correspond to repeats of the r01 to r09 results, respectively. The sequences of r01–r09 are highlighted in yellow. Figure S8: Alignments of the long PacBio reads to the MTPT fragments in the mitogenome of *S. miltiorrhiza*. Panels a–p show the alignments of long PacBio reads to the ten MTPT fragments in MC1 (sami-mtpt-001 to sami-mtpt-010) and six MTPT fragments in MC2 (sami-mtpt-011 to sami-mtpt-016). The MTPT regions are indicated with the red lines with arrow heads at each end. The 2000 bp-long flanking sequences are indicated with a red line without arrow heads. Figure S9: Boxplots of pairwise dN values for mitochondrial genes among the ten Lamiales plants. Figure S10: Boxplots of pairwise dS values for mitochondrial genes among the ten Lamiales plants. Table S1: Summary of sequence data generated by the PacBio RS and Illumina platforms. Table S2: SSRs in the *S. miltiorrhiza* mitogenome. “MC1/2”: mitogenome chromosome 1/2. Table S3: Summary of SSRs in the mitogenome of Lamiales. Table S4: Tandem repeats in the mitogenome of Lamiales. Table S5: PCR primers used to validate the recombination products of nine repeats in the *S. miltiorrhiza* mitogenome. Table S6: Chloroplast DNA insertions in the *S. miltiorrhiza* mitogenome. The three longest regions (sami-mtpt-001, sami-mtpt-002, and sami-mtpt-012) similar to the mitogenome sequence are indicated with “*”. “MC1/2”: mitogenome chromosome 1/2, frag: fragment. File S1: DNA sequence of the plastome of *S. miltiorrhiza* assembled using the GetOrganelle toolkit. File S2: DNA sequences of the four possible conformations associated with eleven HSPs (r01–r11) and their corresponding seven DBSs (bs01–bs07) in the mitogenome of *S. miltiorrhiza*. File S3: Alignment of the sequences of 26 PCGs of ten Lamiales mitogenomes and the results of pairwise dN, dS, and dN/dS values of the two species.

## Abbreviations

mitogenome	Mitochondrial genome
NCBI	National Center for Biotechnology Information
PCG	Protein-coding genes
DNA	Deoxyribonucleic acid
RNA	Ribonucleic acid
C-to-U	Cytidine-to-Uridine
PacBio	Pacific Biosciences
Gb	Giga base pair
DBS	Double bifurcating structures
MC1/2	Mitochondrial genome chromosomes
SSR	Simple sequence repeat
HSP	High-scoring pair
Mac	Major conformation
Mic	Minor conformation
MTPT	Mitochondrial plastid DNA
PCR	Polymerase chain reaction
sami	Salvia miltiorrhiza
NUMT	Nuclear mitochondrial sequence
