# Supplementary material for: De Novo Hybrid Assembly of the Salvia miltiorrhiza Mitochondrial Genome Provides the First Evidence of the Multi-Chromosomal Mitochondrial DNA Structure of Salvia Species
Source: Int J Mol Sci. 2022 Nov 17;23(22):14267. doi: 10.3390/ijms232214267 (PMC9694629; doi:10.3390/ijms232214267)
Supplement: Supplementary file 1 [file ijms-23-14267-s001.zip › ijms-1970424-supplementary.pdf]

SUPPLEMENTARY MATERIALS

Article

De novo assembly of the *Salvia miltiorrhiza* mitochondrial genome using Pacbio long reads provides the first evidence of multi-chromosomal mitochondrial DNA structure of *Salvia* species

Heyu Yang[1,2], Haimei Chen[2], Yang Ni[2], Jingling Li[2], Yisha Cai[2], Binxin Ma[2], Jing Yu[2], Jiehua Wang[1*], Chang Liu[2*]

1 School of Environmental Science and Engineering, Tianjin University, Tianjin 300072, China
2 Institute of Medicinal Plant Development, Chinese Academy of Medical Sciences, Peking Union Medical College, Beijing 100193, China
* Correspondence: cliu6688@yahoo.com; Tel: +86-10-57833111; Fax: +86-10- 62899715; jiehuawang@tju.edu.cn; Tel: +86-022-87402072; Fax: +86-022-27407956

Table S1 Summary of sequence data generated by PacBio RS and Illumina platform.

Sequencing platform	PacBio RS	Illumina
Total number of nucleotides (raw data, bp)	19,434,039,998	16,380,908,800
Total Number of Reads	1,408,786	163,809,088
N50 Read Length	19,289	NA
Mean Read Length (bp)	13,794	100
Mean Read Quality Score	0.82	NA
Total Number of Mapped Reads	23,959 (MC1), 13,431(MC2)	3,291,507 (MC1), 1,021,009(MC2)
Average Coverage Depth	327.77 (MC1), 396.13 (MC2)	969.556 (MC1), 903.15 (MC2)

Table S2 SSRs in the *S. miltiorrhiza* mitogenome. “MC1/2”: mitogenome chromosome 1/2.

Circular Molecule	Repeat unit	3	4	5	6	7	8	9	10	11	12	13	14	Total
MC1	A/T	-	-	-	-	-	-	-	6	3	2	-	2	13
MC1	AG/CT	-	-	14	2	1	-	-	-	-	-	-	-	17
MC1	AT/AT	-	-	8	2	-	-	-	-	-	-	-	-	10
MC1	AAC/GTT	-	3		-	-	-	-	-	-	-	-	-	3
MC1	AAG/CTT	-	3		-	-	-	-	-	-	-	-	-	3
MC1	AAT/ATT	-	5	1	-	-	-	-	-	-	-	-	-	6
MC1	ACG/CGT	-	1	-	-	-	-	-	-	-	-	-	-	1
MC1	ACT/AGT	-	2	-	-	-	-	-	-	-	-	-	-	2
MC1	ATC/ATG	-	1	-	-	-	-	-	-	-	-	-	-	1
MC1	AAAC/GTTT		2	-	-	-	-	-	-	-	-	-	-	2
MC1	AAAG/CTTT	15	-	-	-	-	-	-	-	-	-	-	-	15
MC1	AAAT/ATTT	1	-	-	-	-	-	-	-	-	-	-	-	1
MC1	AACC/GGTT	1	-	-	-	-	-	-	-	-	-	-	-	1
MC1	AAGG/CCTT	4	-	-	-	-	-	-	-	-	-	-	-	4
MC1	AATG/ATTC	3	-	-	-	-	-	-	-	-	-	-	-	3
MC1	ACAT/ATGT	1	-	-	-	-	-	-	-	-	-	-	-	1
MC1	ACCG/CGGT	1	-	-	-	-	-	-	-	-	-	-	-	1
MC1	ACTC/AGTG	1	-	-	-	-	-	-	-	-	-	-	-	1
MC1	AGAT/ATCT	3	-	-	-	-	-	-	-	-	-	-	-	3
MC1	AGCC/CTGG	1	-	-	-	-	-	-	-	-	-	-	-	1
MC1	AGGG/CCCT	1	-	-	-	-	-	-	-	-	-	-	-	1
MC1	CCGG/CCGG	1	-	-	-	-	-	-	-	-	-	-	-	1
MC1	AAAAT/ATTTT	1	-	-	-	-	-	-	-	-	-	-	-	1
MC1	AATAC/ATTGT	1	-	-	-	-	-	-	-	-	-	-	-	1
MC1	ACTAG/AGTCT	1	-	-	-	-	-	-	-	-	-	-	-	1
MC1	AGGAT/ATCCT	1	-	-	-	-	-	-	-	-	-	-	-	1
MC1	AAAGAG/CTCTTT	1	-	-	-	-	-	-	-	-	-	-	-	1
MC1	AGAGAT/ATCTCT	1	-	-	-	-	-	-	-	-	-	-	-	1
MC1	AAAGAG/CTCTTT	-	1	-	-	-	-	-	-	-	-	-	-	1
MC1	AGAGAT/ATCTCT	-	1	-	-	-	-	-	-	-	-	-	-	1
MC2	A/T	-	-	-	-	-	-	-	1	1	-	-	-	2
MC2	AG/CT	-	-	2	-	-	-	-	-	-	-	-	-	2
MC2	AAG/CTT	-	1	1	-	-	-	-	-	-	-	-	-	2
MC2	AAT/ATT	-	2	-	-	-	-	-	-	-	-	-	-	2
MC2	AAAC/GTTT	1	-	-	-	-	-	-	-	-	-	-	-	1
MC2	AAAG/CTTT	2	-	-	-	-	-	-	-	-	-	-	-	2
MC2	AGCG/CGCT	1	-	-	-	-	-	-	-	-	-	-	-	1
MC2	AAAAG/CTTTT	1	-	-	-	-	-	-	-	-	-	-	-	1

Table S3 Summary of SSRs in the mitogenome of Lamiales.

accession number	species	Genome size	mononucleotides	dinucleotides	trinucleotides	tetranucleotides	pentanucleotides	hexanucleotides	Total
NC_023103	*Ajuga reptans*	352069	7	15	16	36	6	0	80
MW553042	*Scutellaria tsinyunensis*	354073	15	15	16	41	3	0	90
MN585275.1/6.1	*Salvia miltiorrhiza*	414114	15	29	20	39	5	4	112
NC_049064	*Rotheca serrata*	482114	14	19	17	34	8	3	95
NC_031806	*Castilleja paramensis*	495499	21	29	15	46	6	0	117
NC_023209	*Salvia miltiorrhiza*	499236	17	27	22	43	6	3	118
NC_016741	*Dorcoceras hygrometricum*	510519	22	34	9	33	5	1	104
NC_018041	*Erythranthe guttata*	525671	36	17	14	44	8	1	120
NC_031323	*Hesperelaea palmeri*	658522	50	40	28	64	8	0	190
NC_034982	*Utricularia reniformis*	857234	36	33	26	63	4	2	164

Table S4 Tandem repeats in the mitogenome of the Lamiales.

accession_number	Indices	Period size(bp)	Copy Number	Consensus Size (bp)	Percent Matches	Percent Indels	Score	Bases number				Entropy
								A	G	C	T	(0–2)
MN585276.1	194570-194609	15	2.5	16	88	8	55	70	17	2	10	1.27
MN585275.1	358379-358424	21	2.1	22	88	11	69	50	0	0	50	1
MN585275.1	402285-402332	16	2.9	16	76	14	53	33	20	12	33	1.9
NC_016741	5295-5330	17	2.1	17	89	0	54	41	11	5	41	1.64
NC_016741	208537-208578	19	2.2	20	87	12	61	52	7	26	14	1.67
NC_016741	237481-237523	21	2	21	95	0	77	27	11	32	27	1.92
NC_016741	283955-283988	16	2	16	88	11	50	58	0	0	41	0.98
NC_016741	364263-364297	18	2	17	88	5	52	5	11	11	71	1.3
NC_018041	9641-9681	20	2	20	100	0	82	24	12	19	43	1.85
NC_018041	31116-31183	18	3.8	18	98	0	127	38	22	16	23	1.93
NC_018041	65710-65739	16	1.9	15	93	6	51	43	13	30	13	1.82
NC_018041	94432-94509	40	1.9	42	81	5	97	17	20	21	39	1.92
NC_018041	104253-104302	24	2.1	24	96	0	91	40	18	20	22	1.92
NC_018041	120892-120935	14	3.1	14	90	6	63	72	6	2	18	1.17
NC_018041	163482-163508	13	2.1	13	100	0	54	14	22	0	62	1.31
NC_018041	180754-180796	21	2	21	100	0	86	32	37	18	11	1.87
NC_018041	222539-222579	20	2	20	100	0	82	43	19	12	24	1.85
NC_018041	234667-234691	12	2.1	12	100	0	50	44	8	32	16	1.76
NC_018041	241974-242009	18	1.9	19	88	5	56	36	33	2	27	1.72
NC_018041	270916-271069	69	2.2	69	84	2	202	25	18	18	38	1.93
NC_018041	270941-271089	69	2.2	69	86	4	203	24	21	15	38	1.92

NC_018041	327462-327529	34	2	34	94	0	118	20	32	17	29	1.96
NC_018041	331088-331149	31	2	31	90	0	97	11	22	16	50	1.76
NC_018041	332369-332410	21	2	21	95	0	75	9	40	19	30	1.83
NC_018041	334170-334225	30	2	28	86	13	80	26	12	44	16	1.83
NC_018041	341742-341805	21	3	21	100	0	128	28	29	9	32	1.88
NC_018041	346877-346912	18	2	18	100	0	72	38	0	44	16	1.48
NC_018041	351394-351431	18	2.1	18	95	0	67	34	18	18	28	1.95
NC_018041	376089-376127	20	2	20	84	0	51	0	28	10	61	1.28
NC_018041	406948-406991	20	2.2	20	100	0	88	31	13	22	31	1.93
NC_018041	472313-472338	13	2	13	100	0	52	38	0	15	46	1.46
NC_018041	479695-479737	21	2	21	90	0	68	39	20	23	16	1.92
NC_018041	481207-481232	12	2.2	12	100	0	52	26	34	15	23	1.94
NC_031323	168064-168145	36	2.3	36	100	0	164	23	21	30	24	1.99
NC_031323	180046-180072	14	1.9	14	100	0	54	66	11	22	0	1.22
NC_031323	182710-182752	21	2	21	86	8	61	34	25	20	18	1.96
NC_031323	298153-298182	11	2.7	11	94	0	51	40	0	0	60	0.97
NC_031323	333953-333989	18	2.1	18	100	0	74	62	0	27	10	1.28
NC_031323	379363-379401	18	2.2	18	95	0	69	30	7	25	35	1.84
NC_031323	405342-405383	2	22.5	2	81	13	54	47	2	0	50	1.14
NC_031323	407129-407168	19	2.1	19	90	0	62	35	7	12	45	1.7
NC_031323	452885-452923	20	2	20	89	0	60	28	15	30	25	1.96
NC_031323	479717-479747	15	2.1	15	93	0	53	45	16	6	32	1.72
NC_031323	491285-491325	21	2	21	95	0	73	34	9	26	29	1.88
NC_031323	541075-541103	14	2.1	14	100	0	58	58	6	6	27	1.5
NC_031323	552386-552442	24	2.4	24	100	0	114	8	29	22	38	1.85
NC_031323	552386-552452	24	2.8	24	93	0	98	8	26	20	43	1.82

NC_031806	110400-110425	13	2	13	100	0	52	46	15	0	38	1.46
NC_031806	237194-237259	24	2.8	24	92	0	114	48	7	18	25	1.74
NC_031806	312354-312413	18	3.3	18	97	0	111	33	26	10	30	1.89
NC_031806	312354-312423	18	4.1	17	92	5	81	35	25	8	30	1.86
NC_031806	330781-330817	18	2.1	18	94	0	65	29	24	18	27	1.98
NC_031806	331981-332011	15	2.1	15	100	0	62	35	25	0	38	1.56
NC_031806	332124-332170	24	2	24	82	0	58	23	14	31	29	1.95
NC_031806	350079-350144	24	2.8	24	95	0	114	22	21	15	40	1.9
NC_031806	350132-350195	18	3.6	18	89	0	92	20	34	17	28	1.95
NC_031806	402577-402606	15	2	15	100	0	60	40	6	13	40	1.71
NC_031806	440298-440340	19	2.6	15	75	25	50	25	6	11	55	1.6
NC_031806	440304-440341	19	2	19	100	0	76	26	5	10	57	1.53
NC_031806	457267-457310	22	2	22	95	0	79	31	29	27	11	1.91
NC_031806	481432-481456	12	2.1	12	100	0	50	8	24	28	40	1.83
NC_034982	263418-263464	24	2	24	86	0	67	29	21	25	23	1.99
NC_034982	264919-264970	28	2	25	85	14	70	17	42	11	28	1.84
NC_034982	337073-337105	16	2.1	16	94	0	57	36	18	27	18	1.94
NC_034982	427450-427497	25	2	23	84	15	62	37	12	33	16	1.86
NC_034982	681553-681610	21	2.8	21	80	10	57	44	15	27	12	1.82
NC_023103	110693-110742	25	2	25	100	0	100	28	28	4	40	1.74
NC_023103	208003-208043	21	2	21	100	0	82	46	4	14	34	1.66
NC_023103	246827-246860	16	2.1	17	88	5	52	47	8	5	38	1.59
NC_023103	246807-246864	19	2.9	21	71	23	52	41	8	8	41	1.66
NC_023103	342398-342426	14	2.1	14	100	0	58	27	6	13	51	1.66
NC_023209	21035-21082	16	2.9	16	76	14	53	33	12	20	33	1.9
NC_023209	147782-147836	24	2.5	22	85	8	67	14	23	12	49	1.78

NC_023209	147804-147871	21	3.2	21	77	8	75	11	22	10	55	1.65
NC_023209	150188-150242	18	3.1	18	91	0	83	29	9	25	36	1.87
NC_023209	172593-172632	15	2.5	17	88	8	57	10	17	2	70	1.27
NC_023209	295019-295071	21	2.5	22	87	9	74	49	0	0	50	1
NC_023209	371146-371177	16	2	16	93	0	55	25	21	15	37	1.93
NC_049064	81772-81806	16	2.3	16	90	5	54	2	37	14	45	1.59
NC_049064	132247-132300	15	3.6	15	94	0	90	40	11	29	18	1.85
NC_049064	160514-160561	6	8	6	100	0	96	33	33	0	33	1.58
NC_049064	185885-185910	13	2	13	100	0	52	46	15	0	38	1.46
NC_049064	269642-269742	24	4.2	24	96	0	175	35	3	35	24	1.74
NC_049064	283791-283836	23	2	23	91	8	76	13	26	13	47	1.78
NC_049064	334317-334395	26	3	26	100	0	158	26	18	15	39	1.91
NC_049064	340274-340318	23	1.9	24	90	4	74	51	15	26	6	1.68
NC_049064	422418-422487	6	11.7	6	100	0	140	17	31	0	51	1.45
NC_049064	470684-470718	18	1.9	18	88	0	52	54	8	8	28	1.6
MW553042	24043-24082	15	2.5	16	88	8	55	70	2	17	10	1.27
MW553042	33394-33438	22	2	22	82	0	54	20	22	15	42	1.89
MW553042	100394-100439	23	2	23	91	8	76	47	13	26	13	1.78
MW553042	162197-162267	37	1.9	37	94	0	124	14	14	50	21	1.77
MW553042	216408-216447	20	2	20	95	0	71	0	32	10	57	1.32
MW553042	320051-320098	24	2	24	91	0	78	33	20	39	6	1.78

Table S5 PCR primers used to validate recombination products of the nine repeats in the *S. miltiorrhiza* mitogenome.

Primer Name	Primer Sequence
sami-r01-F1	CAGTCTATTTCGCCCTGTCC
sami-r01-R1	TAATCGACGGCTGCTAACTCG
sami-r01-F2	AGGTTTACATTCCTCCCTGCA
sami-r01-R2	ACCCTTATTCCGGTGTCCTT
sami-r02-F1	ATGGGGTGGAAGTGGAGATG
sami-r02-R1	TCCCATCTTGATTTGCCCTCT
sami-r02-F2	TTGACCGGAACTGGAGTCTC
sami-r02-R2	CGCTGATCTGATCCTCCCTT
sami-r03-F1	CACCAGTAGCCAAGGAAGGA
sami-r03-R1	GAAAACACGCCCTCTTTGGT
sami-r03-F2	TCCTCAACCTCATCTGCAGC
sami-r03-R2	GGAAGAGCACGAACAAGAGC
sami-r04-F1	CAACGTCGCTTGGATCAGAG
sami-r04-R1	AGCGCCGAGAGATCTGTTAA
sami-r04-F2	GCTTTATGGTCCCTGTCCCT
sami-r04-R2	TGGTGGTTGAGTAAGGGCAT
sami-r05-F1	GTCTTCGAGCCAGTACCCTT
sami-r05-R1	TTGGGCATGAATGGAGCTTG
sami-r05-F2	TAACCATCACAGGACCCGAC
sami-r05-R2	CCCCAGAGTGTGCCTTTCTT
sami-r06-F1	TGAACCGAGGACTCTTGCTT
sami-r06-R1	CAAGTGAGTGAGTGGCAGTG
sami-r06-F2	TCTTTCCCCTCACAAGCCAT
sami-r06-R2	CCAGGCCCATCAGACCATAA
sami-r07-F1	CTACGCGCGTATACTGGTTG
sami-r07-R1	CGTAGTTGATGATTCCGCCG
sami-r07-F2	CGCCCGGATACAAAGGAATC
sami-r07-R2	CTCCCCATATTCCCCACTCA
sami-r08-F1	CTGAGGGTAGGGTTAAGGGC
sami-r08-R1	GCCCCAAGAAGCATCAAACA
sami-r08-F2	CATCTCCGCCTACTCCACTC
sami-r08-R2	ACCTATCATCGCCGGCATAA
sami-r09-F1	ACACCTCAGGCCCCTTTATC
sami-r09-R1	CCCGGGTTACACTCTTCCAT
sami-r09-F2	GCTCTGAAAACCACCTCAAATC
sami-r09-R2	AGAAAGTATGAGGCCCGTCC

Table S6 Chloroplast DNA insertions in the *S. miltiorrhiza* mitogenome. The three longest regions (sami-mtpt-001, sami-mtpt-002, and sami-mtpt-012) similar to the mitogenome sequence are indicated with "*". "MC1/2": mitogenome chromosome 1/2, frag: fragment.

MTPT ID	Name of Circular molecule	Identity (%)	Alignment Length (bp)	Number of Mismatches	Number of Gap openings	Positions on the Chloroplast Genome		Positions on the Mitochondrial Genome		Genes Located in the MTPT Fragments	E value	Score
						Start	End	Start	End			
sami-mtpt01	MC1	98.10	1949*	23	6	70841	72786	72166	70229	*psb*B-frag	0	3382
sami-mtpt-002	MC1	99.71	1737*	2	1	63951	65684	308153	309889	*pet*L,*pet*G,*trn*W-CCA,*trn*P-UGG-frag	0	3177
sami-mtpt-003	MC1	100	623	0	0	32783	33405	230470	229848	*psb*C-frag	0	1151
sami-mtpt-004	MC1	100	135	0	0	32376	32510	264504	264638	*psb*D-frag	2.06E-65	250
sami-mtpt-005	MC1	75.22	452	102	9	10441	10887	119207	118761	*atp*A-frag	4.52E-52	206
sami-mtpt-006	MC1	94.03	134	7	1	40981	41113	264689	264822	*psa*A-frag	5.85E-51	202
sami-mtpt-007	MC1	87.64	178	11	6	45376	45553	298972	299138	*rps*4-frag	2.72E-49	196
sami-mtpt-008	MC1	94.78	115	6	0	44704	44818	298852	298966	t*rn*S-GGA	2.74E-44	180

sami-mtpt-009	MC1	91.04	134	2	3	132622	132754	164757	164881	*trn*I-GAU-frag	4.59E-42	172
sami-mtpt-010	MC1	90.07	131	12	1	29796	29925	298382	298252	*trn*D-GUC	5.93E-41	169
sami-mtpt-011	MC2	99.92	4987*	3	1	51184	56169	78467	73481	*trn*M-CAU,*atp*E,*atp*B,*rbc*L	0.00E+00	9186
sami-mtpt-012	MC2	99.52	632	3	0	37959	38590	23985	24616		0.00E+00	1151
sami-mtpt-013	MC2	98.79	332	4	0	23320	23651	30454	30785		7.69E-169	592
sami-mtpt-014	MC2	88.73	355	33	2	717	1070	21738	22086		2.30E-119	427
sami-mtpt-015	MC2	100	214	0	0	83019	83232	53309	53522		6.47E-110	396
sami-mtpt-016	MC2	76.37	127	30	0	40230	40356	24032	24158		1.60E-11	69.4

Figure S1. Comparison of the sequences of the plastome assembled in this study and NC_020341.1 of *S. miltiorrhiza.* The X-axis shows the nucleotide sequence of plastome assembled in this study, and the Y-axis displays the nucleotide sequence of NC_020341.1.

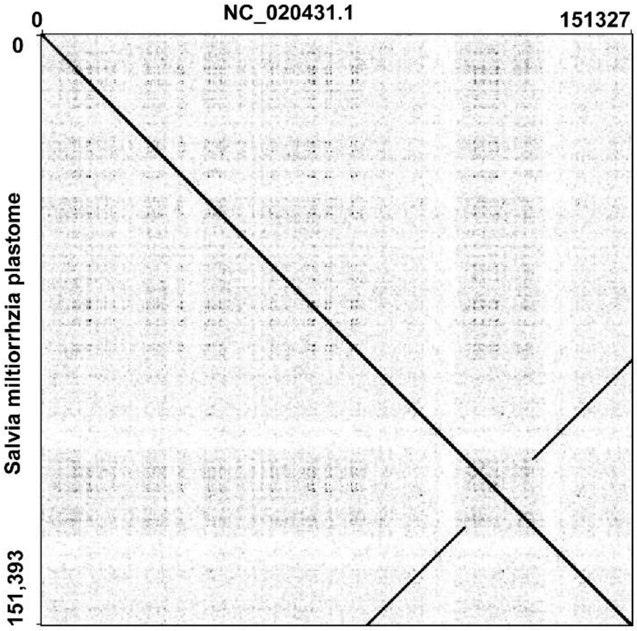

Figure S2. Alignments of long PacBio reads to the four conformations of the seven DBS (bs01–bs07) found in the unitig graph. The unitig graph was generated using Unicycler from Illumina reads, which were filtered with GetOrganelle for mitochondrial reads. Mac corresponds to the connection of shared contigs and their flanking contigs found in MC1 and MC2 (Fig. 1B). Mic corresponds to the alternative connection of the shared contigs and their flanking contigs that are not found in MC1 and MC2. Panels a–g show Mac1 and Mac2 and Mic1 and Mic2 of bs01–bs07, respectively. The figure in each panel can be divided into the top and bottom parts. The top part shows a bird's eye view, whereas the bottom part reveals a base-level view. At the bottom part of the figure, the DBS ID, length of shared contig, and conformation name are shown. The boundaries of shared contig are indicated with red vertical lines. The contigs in the DBS are shown as the red line having arrows at each end. It was labeled as the "repeat region".

a

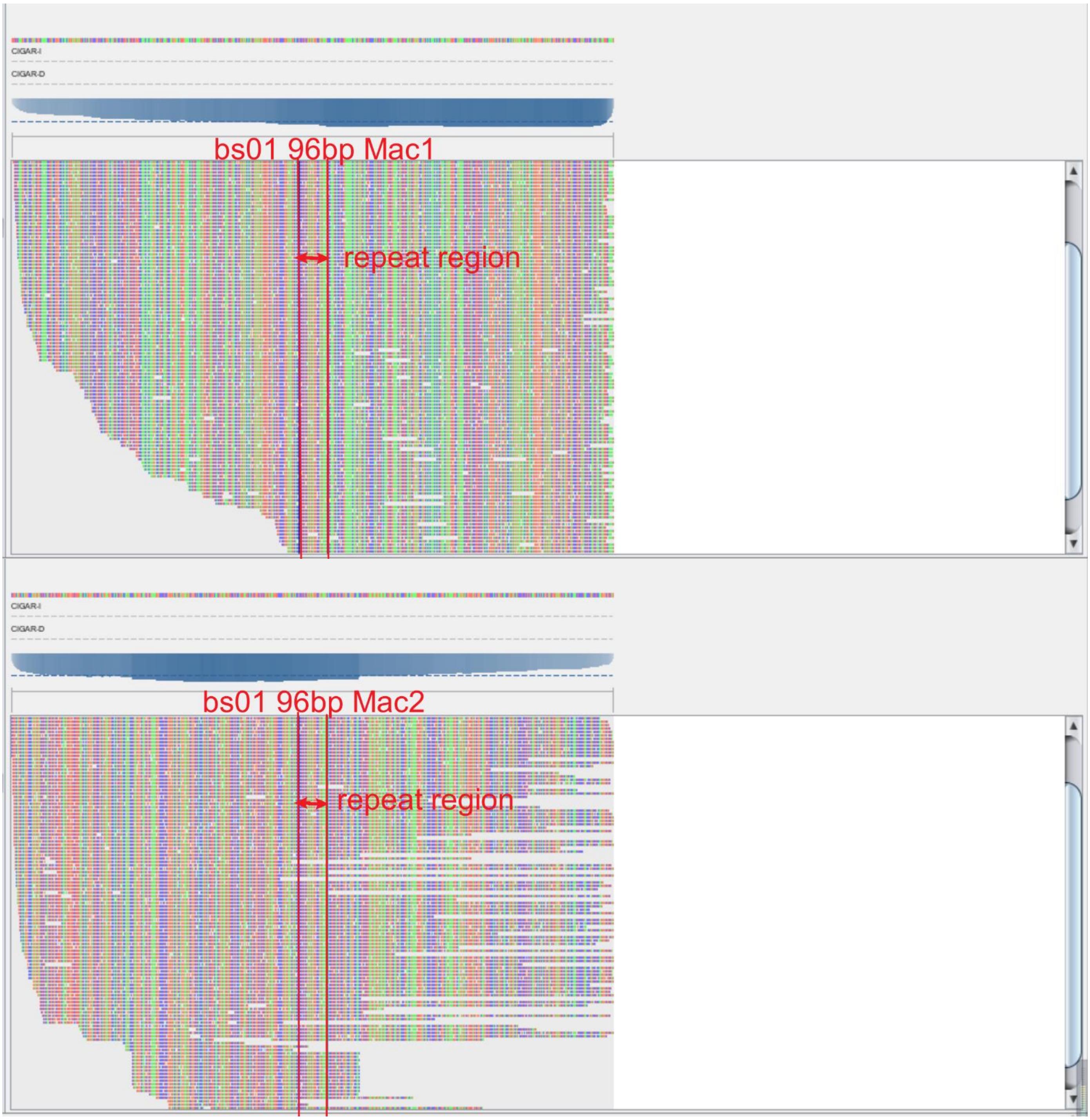

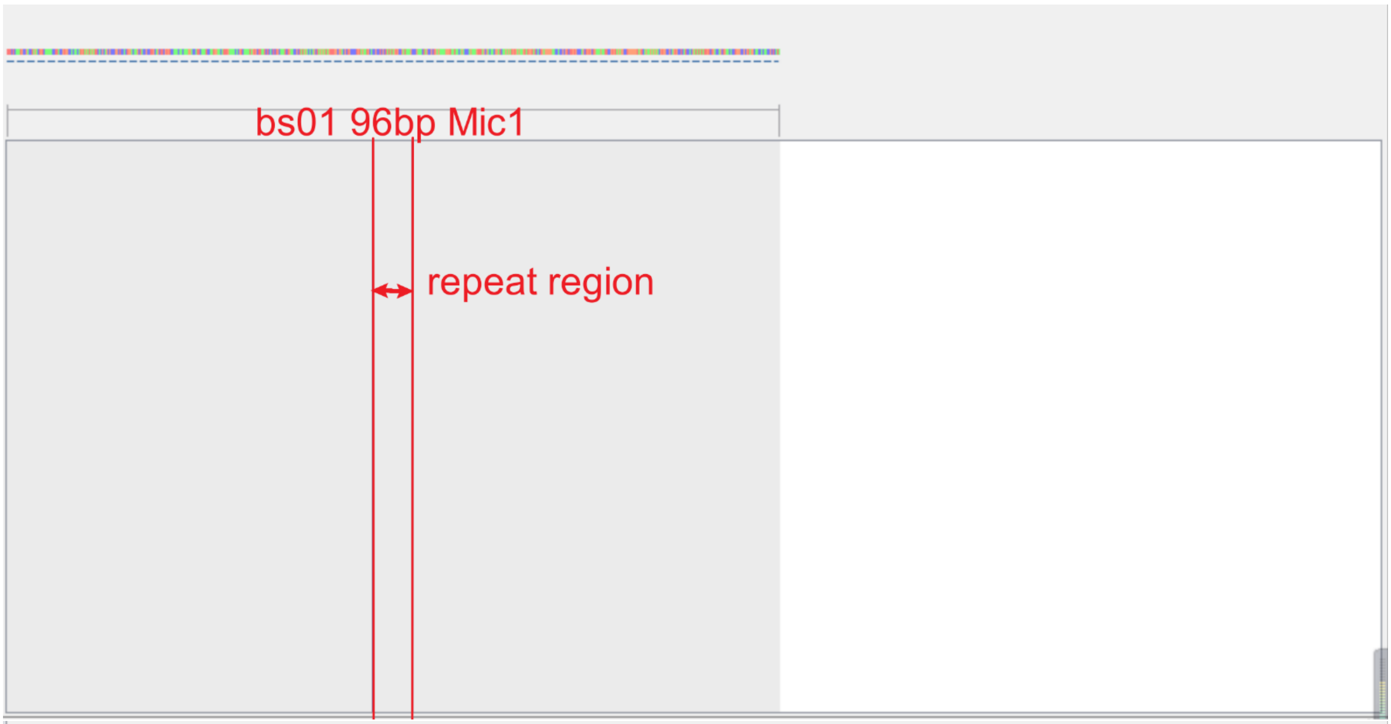
bs01 96bp Mic1
repeat region

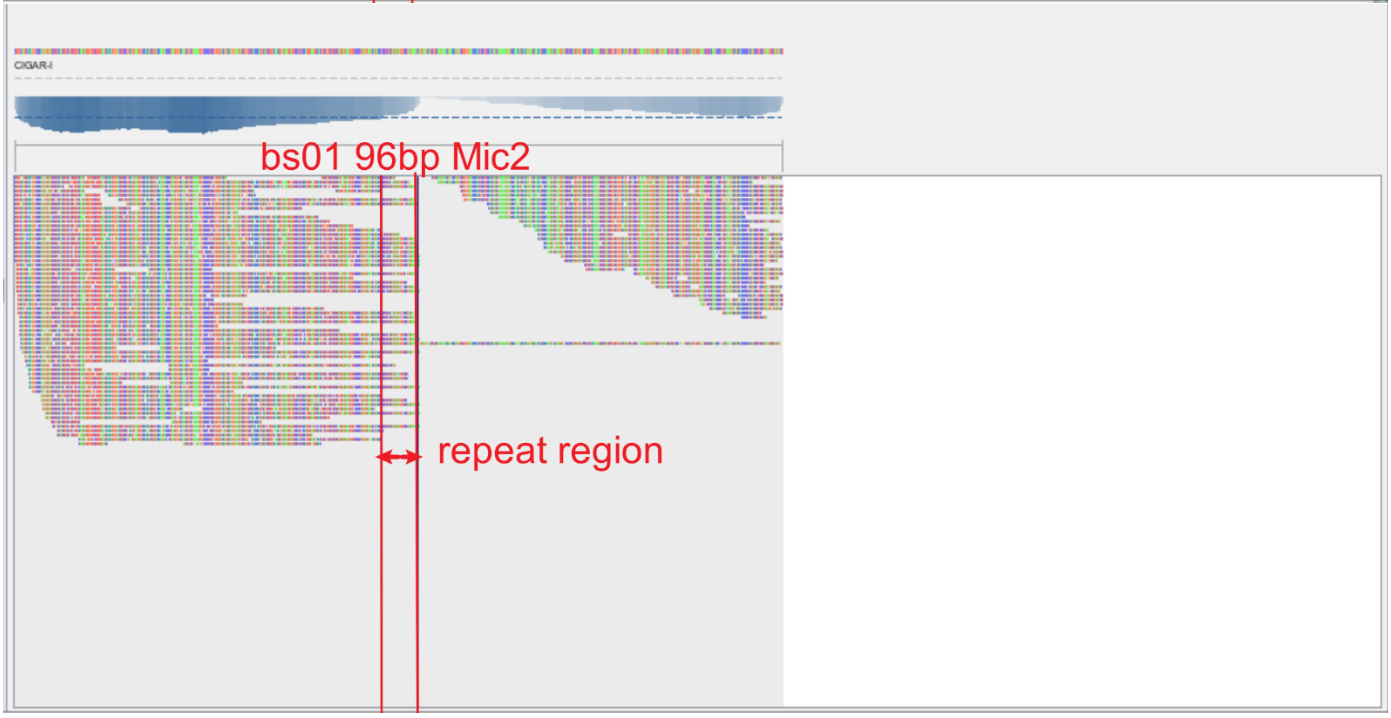
CIGAR-I
bs01 96bp Mic2
repeat region

b

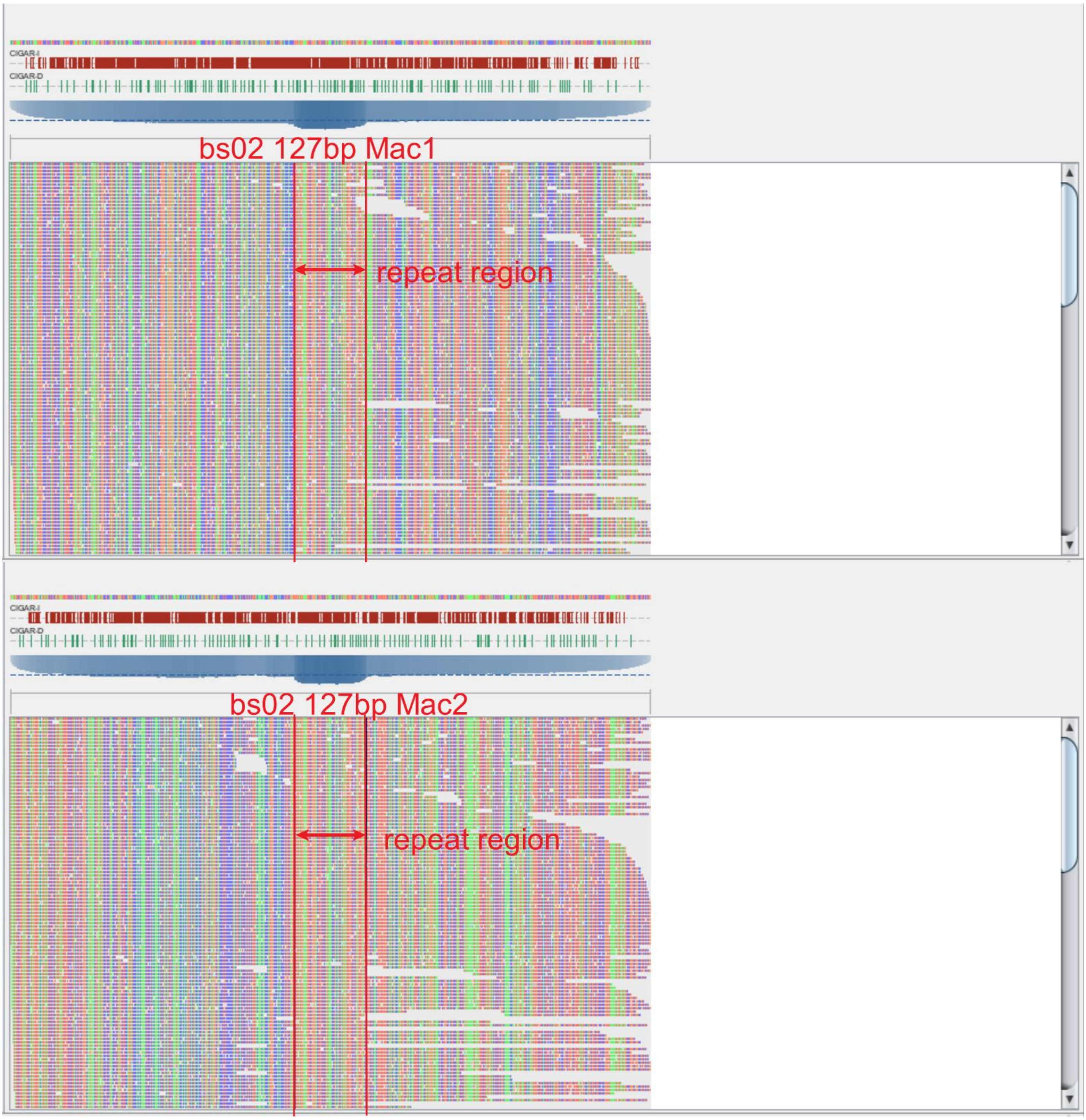
CIGAR-I
CIGAR-D
bs02 127bp Mac1
repeat region
CIGAR-I
CIGAR-D
bs02 127bp Mac2
repeat region

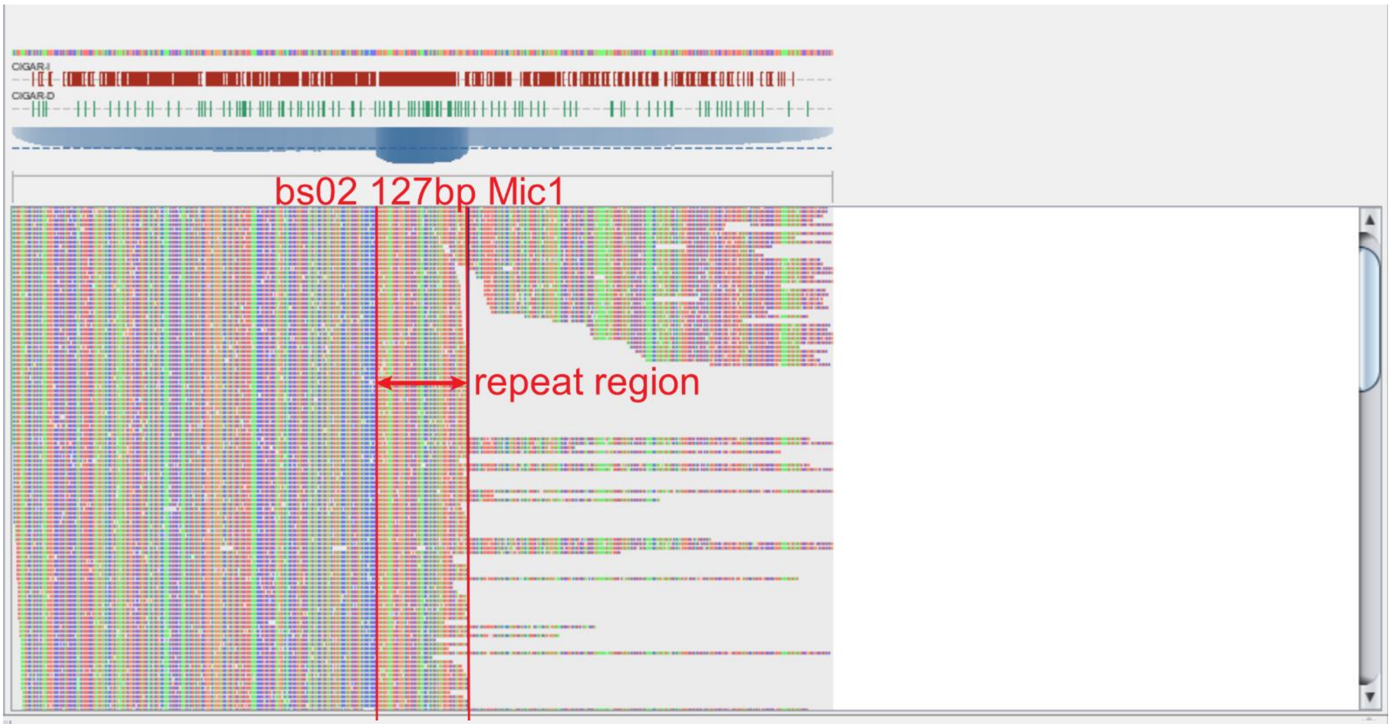
CIGAR-I
CIGAR-D
bs02 127bp Mic1
repeat region

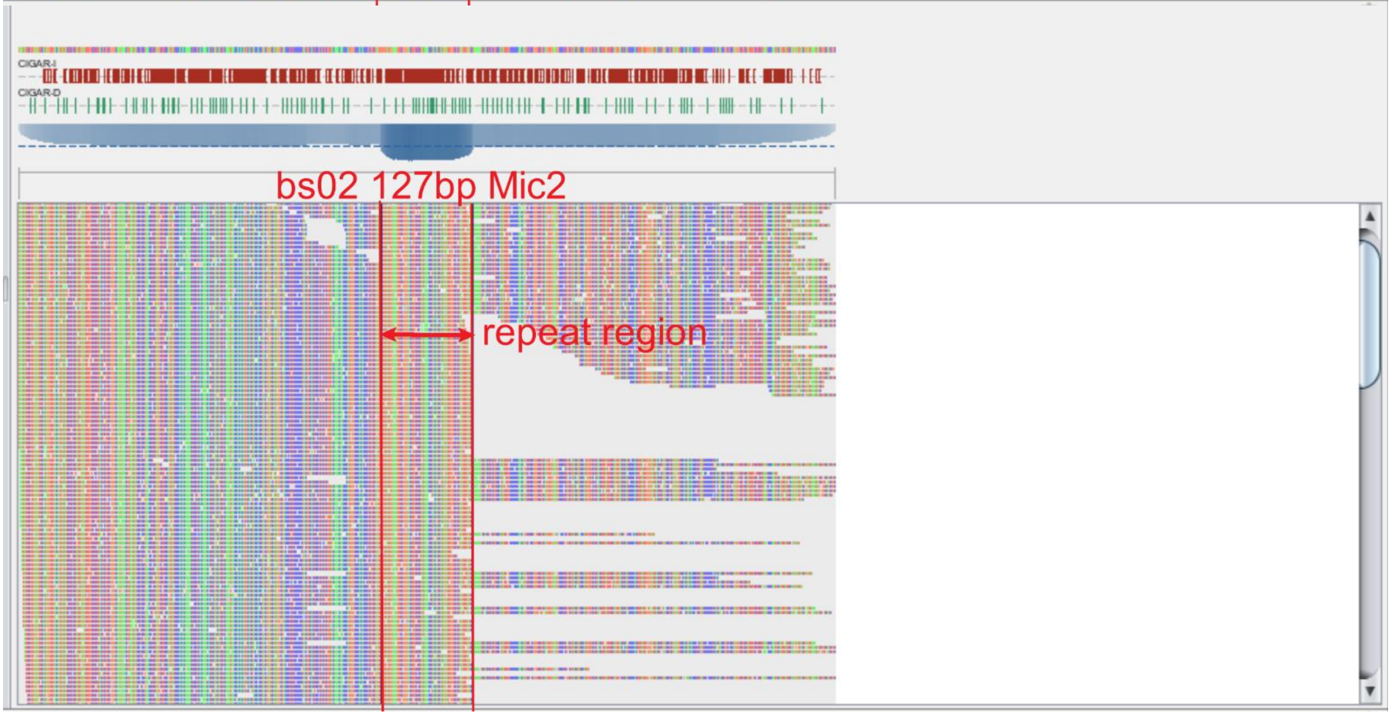
CIGAR-I
CIGAR-D
bs02 127bp Mic2
repeat region

c

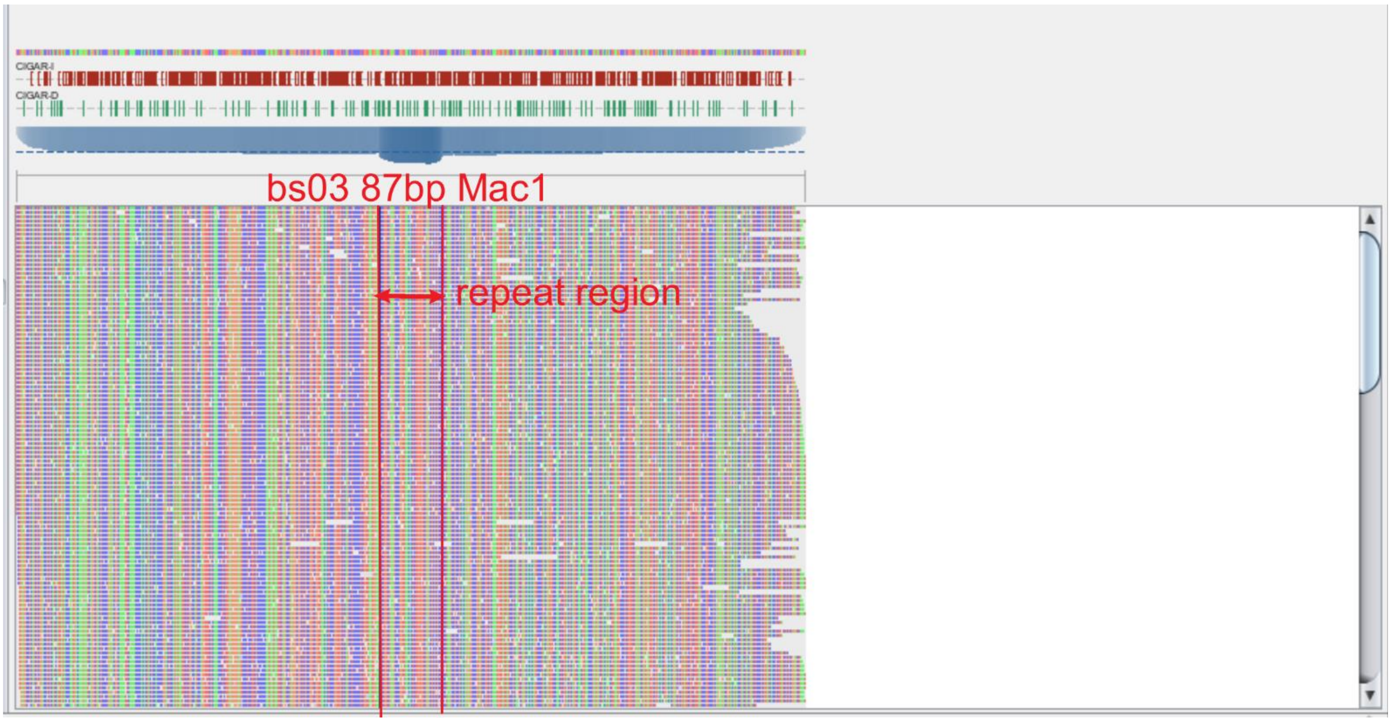

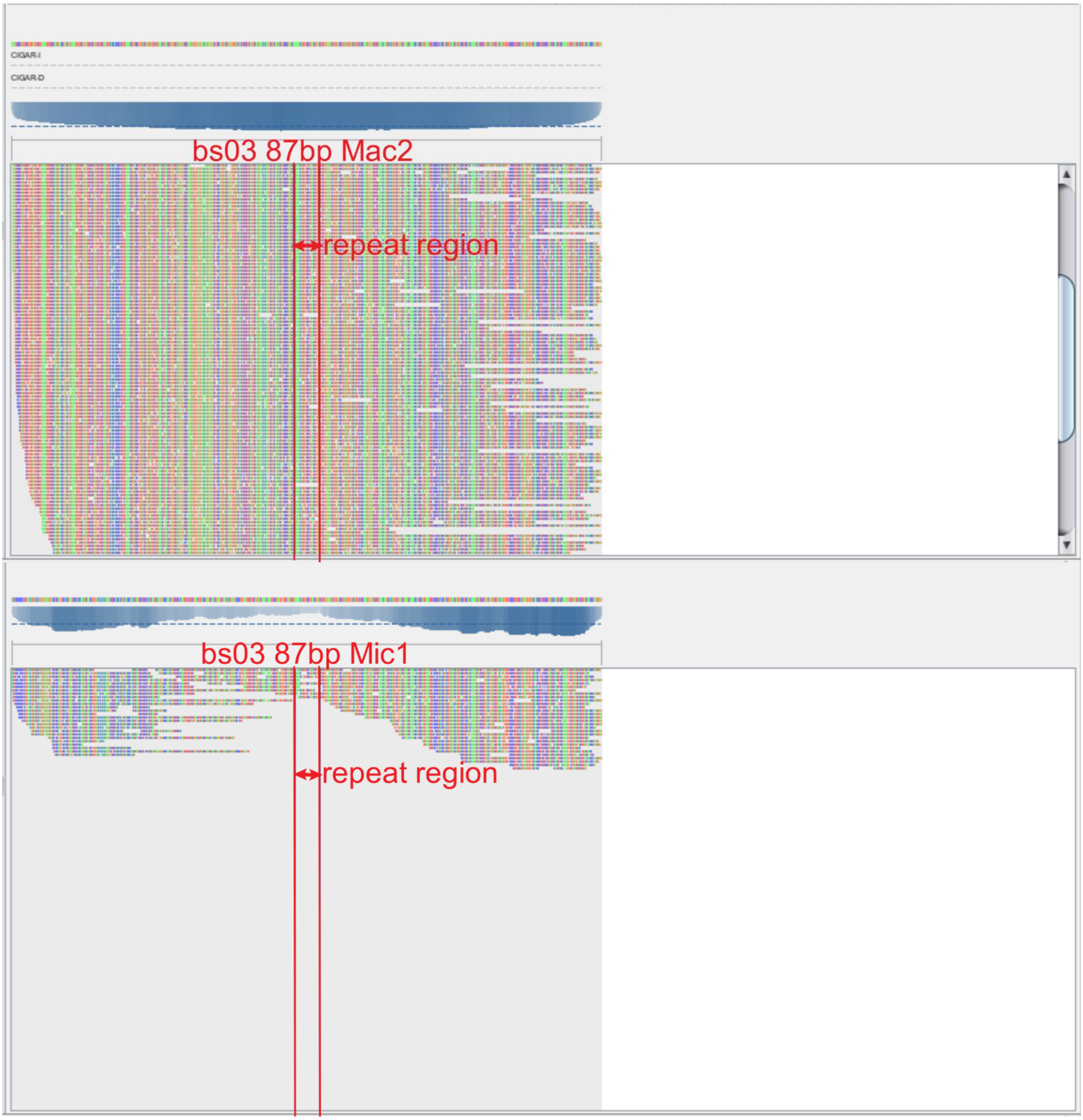
CIGAR-I
CIGAR-D
bs03 87bp Mac2
repeat region
bs03 87bp Mic1
repeat region

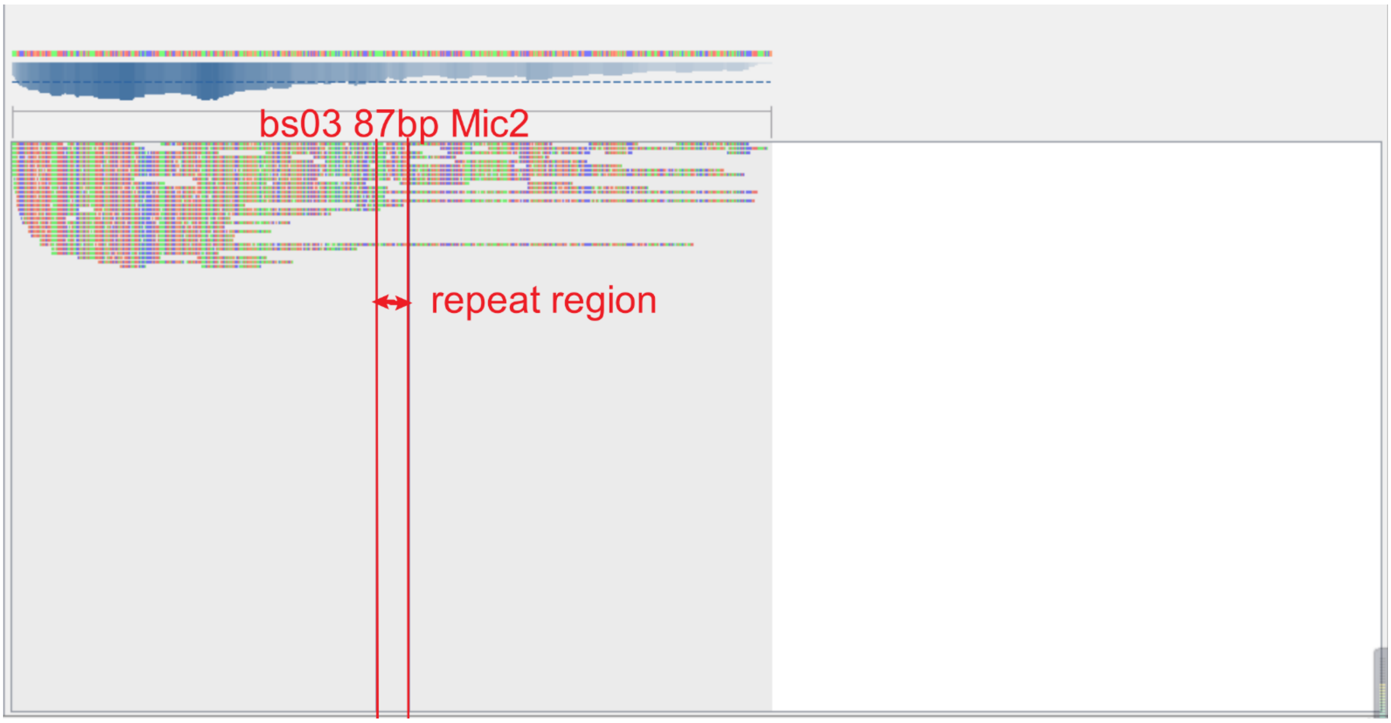
bs03 87bp Mic2
repeat region

d

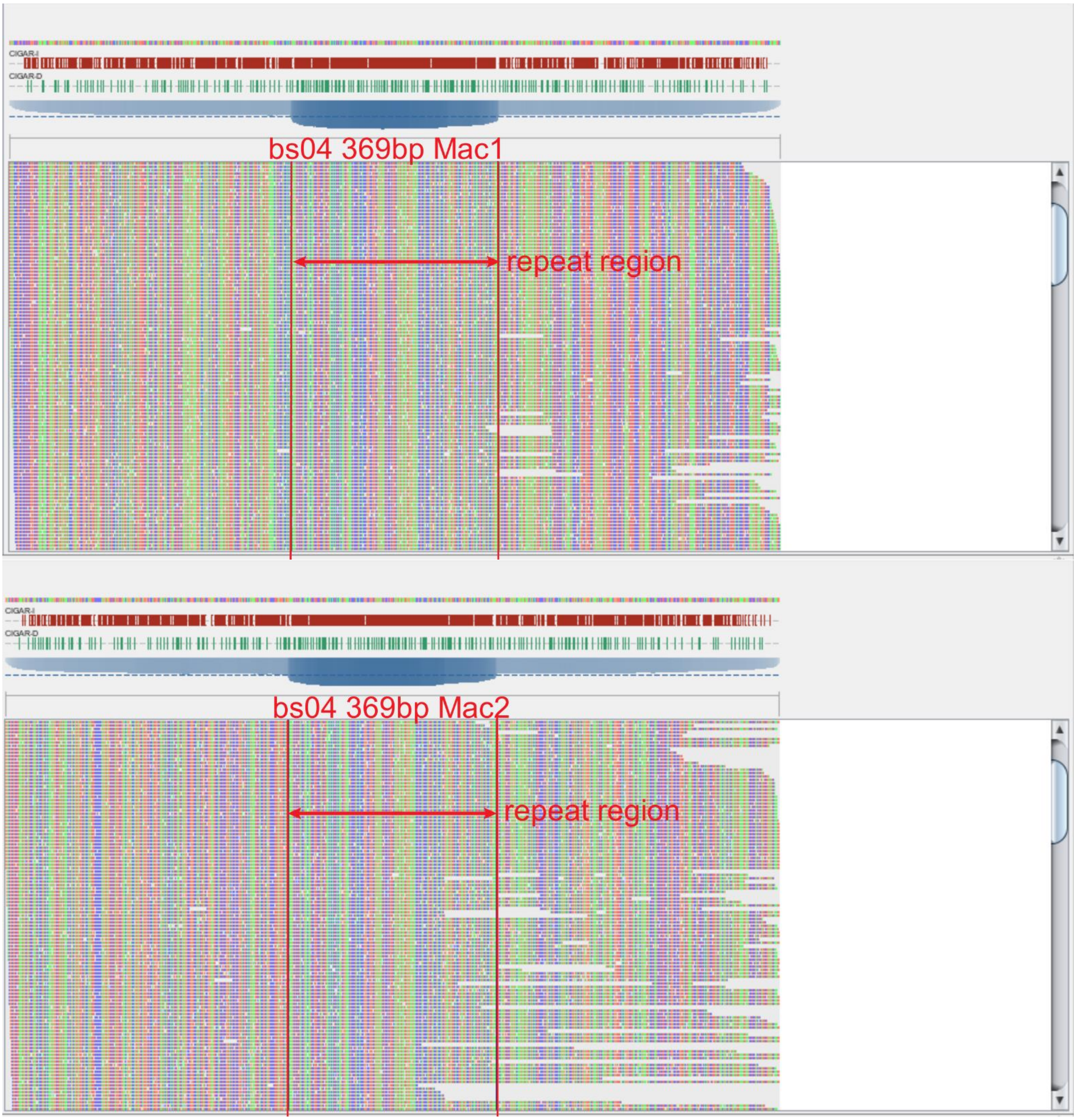
CIGAR-I
CIGAR-D
bs04 369bp Mac1
repeat region
CIGAR-I
CIGAR-D
bs04 369bp Mac2
repeat region

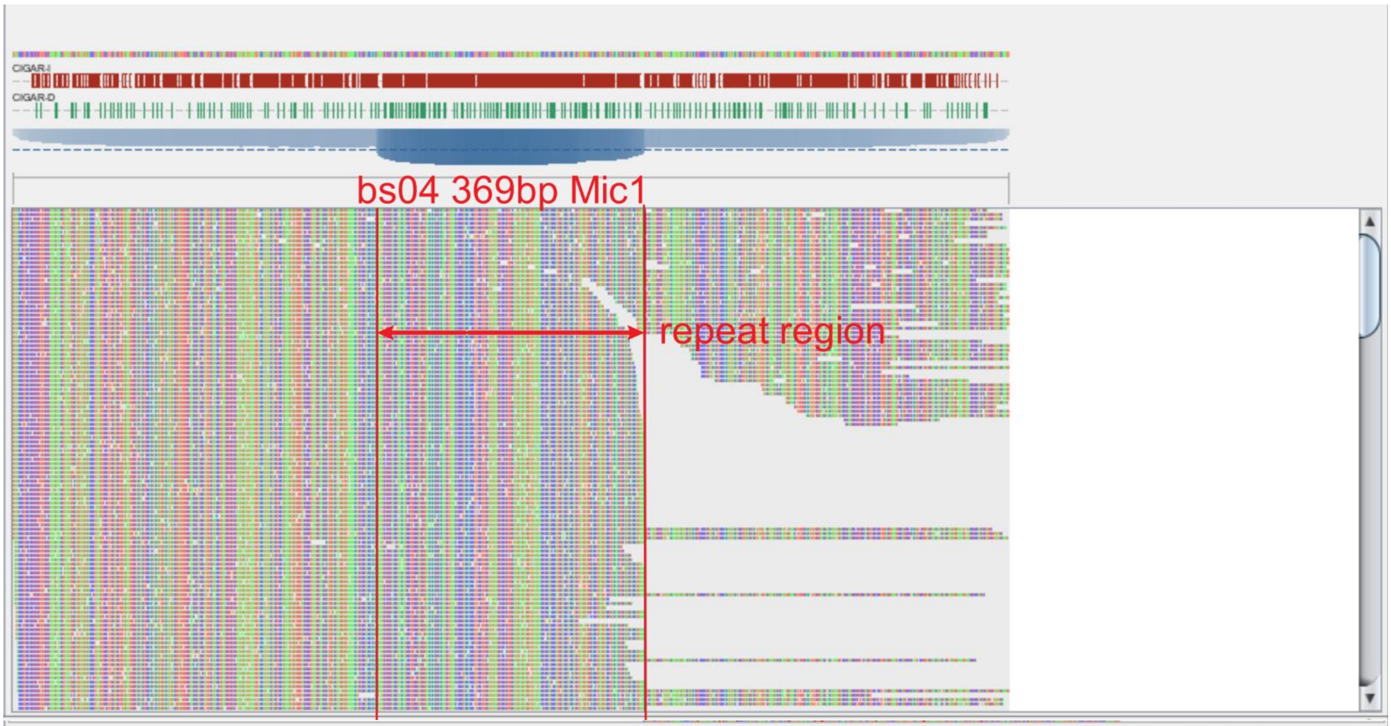
CIGAR-I
CIGAR-D
bs04 369bp Mic1
repeat region

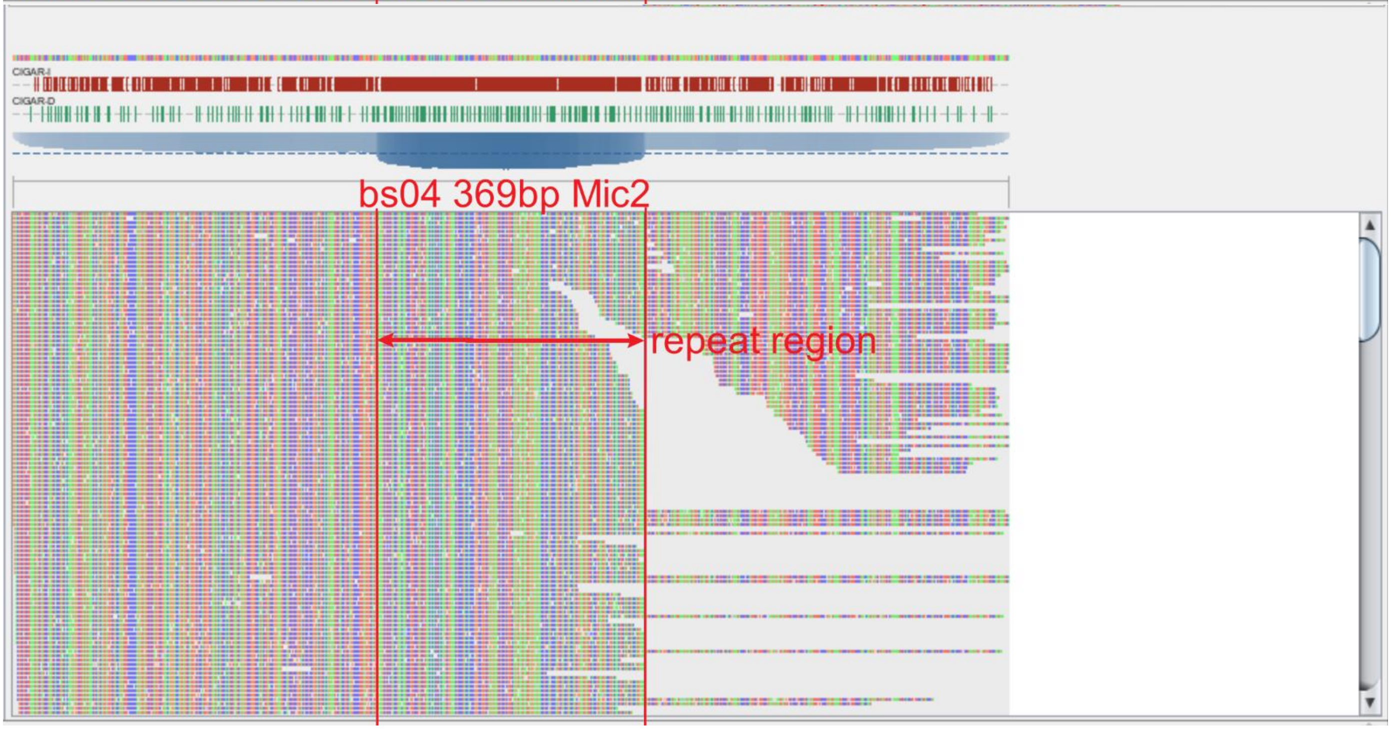
CIGAR-I
CIGAR-D
bs04 369bp Mic2
repeat region

e

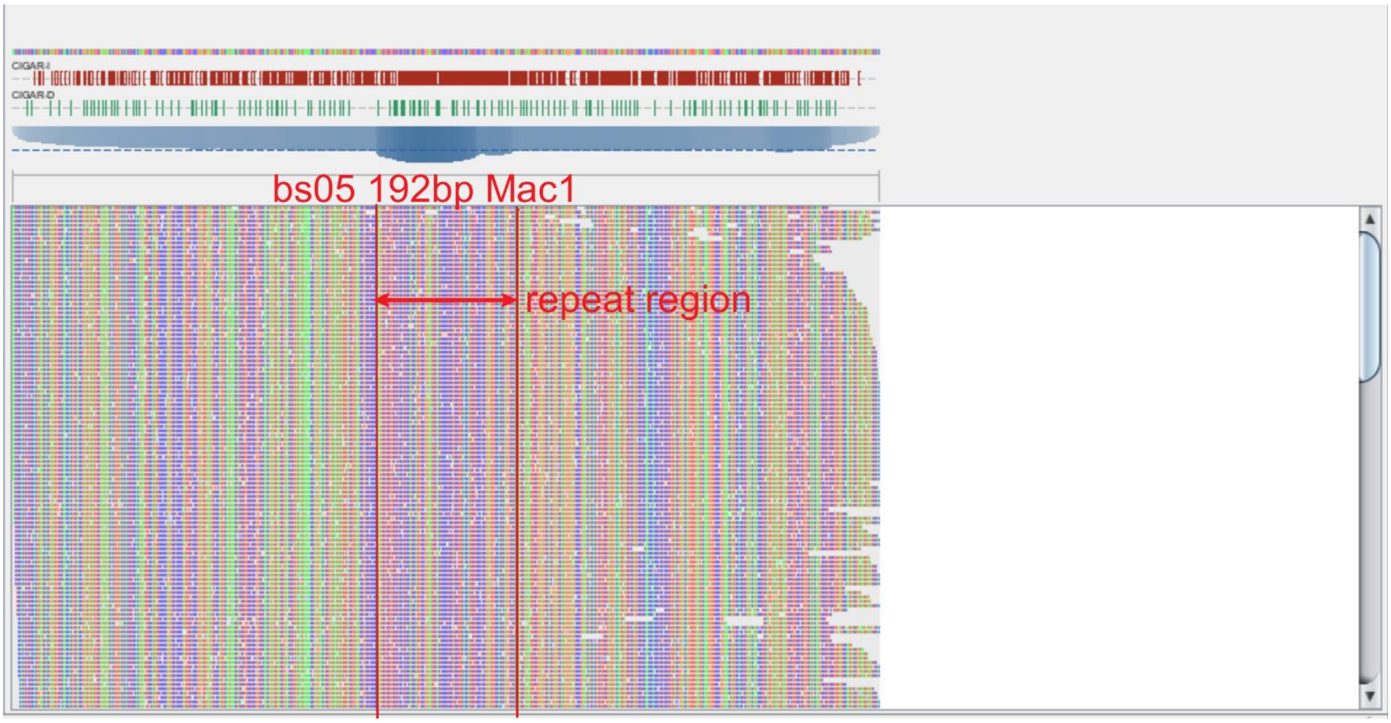

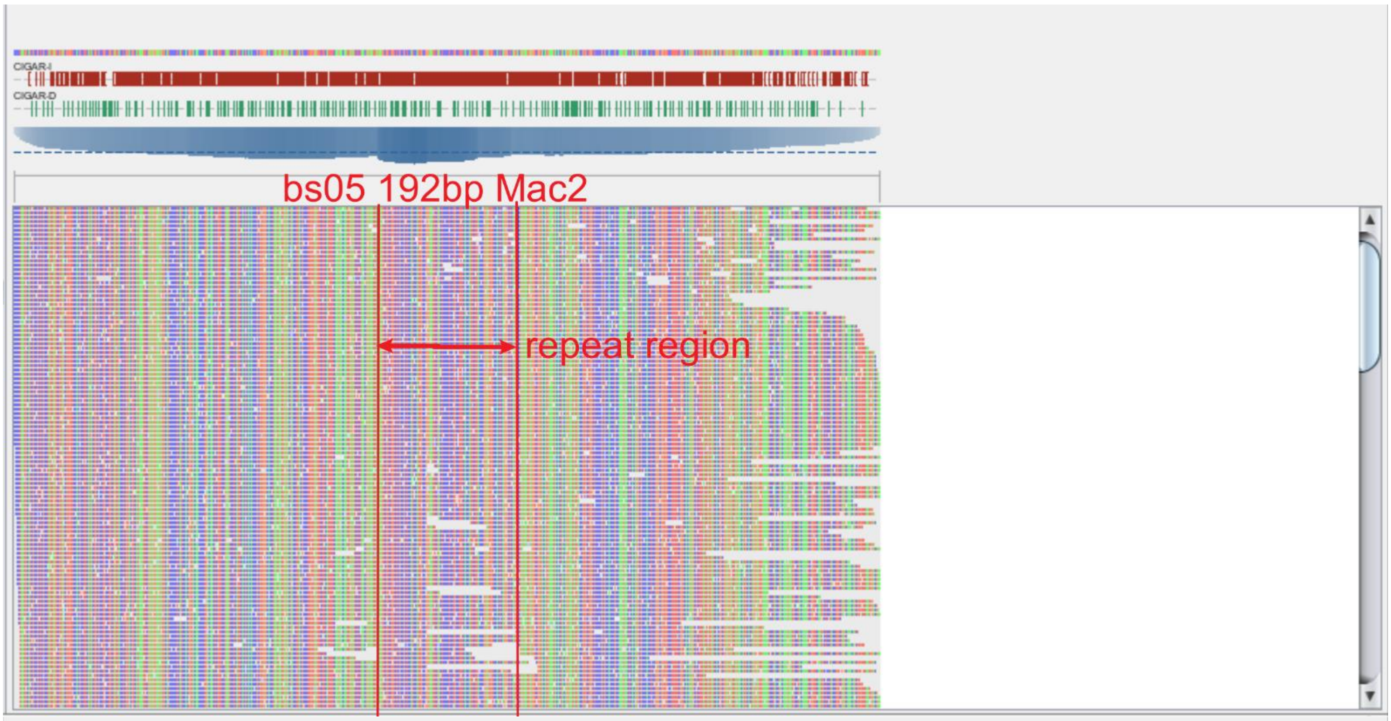
CIGAR-I
CIGAR-D
bs05 192bp Mac2
repeat region

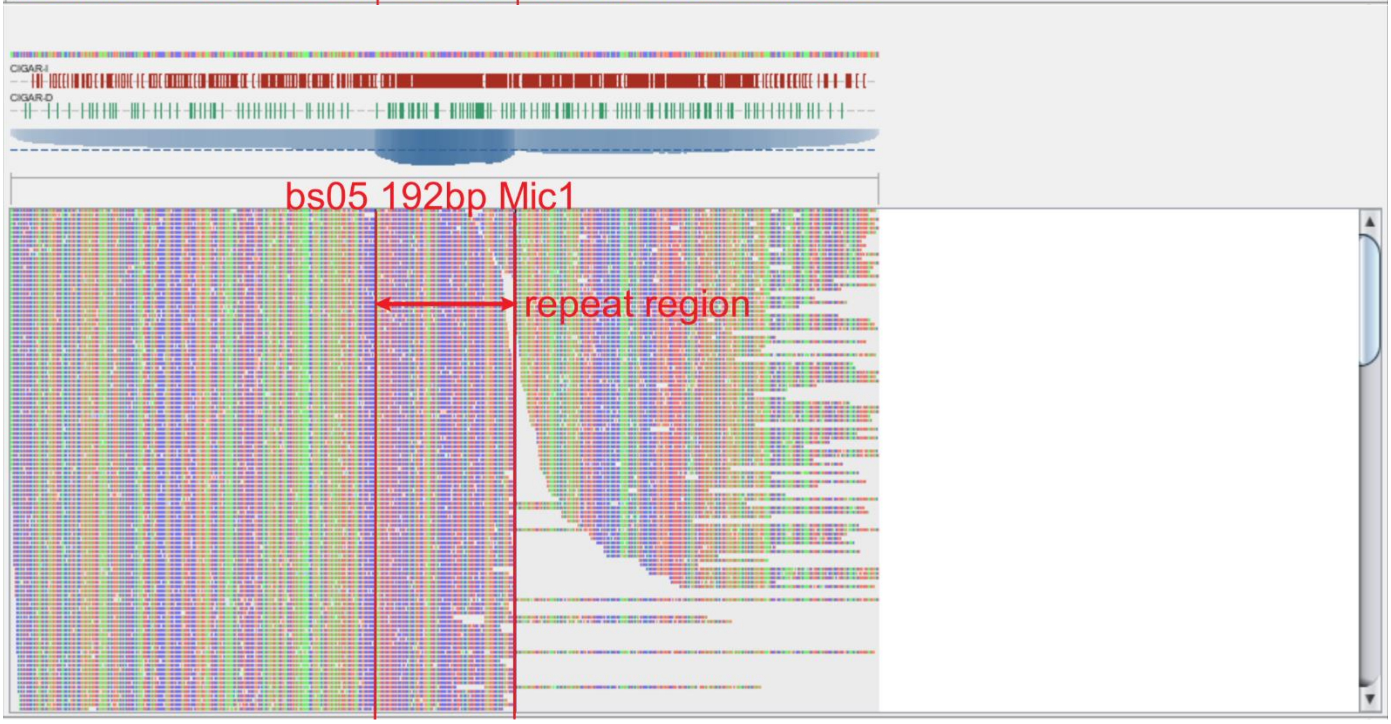
CIGAR-I
CIGAR-D
bs05 192bp Mic1
repeat region

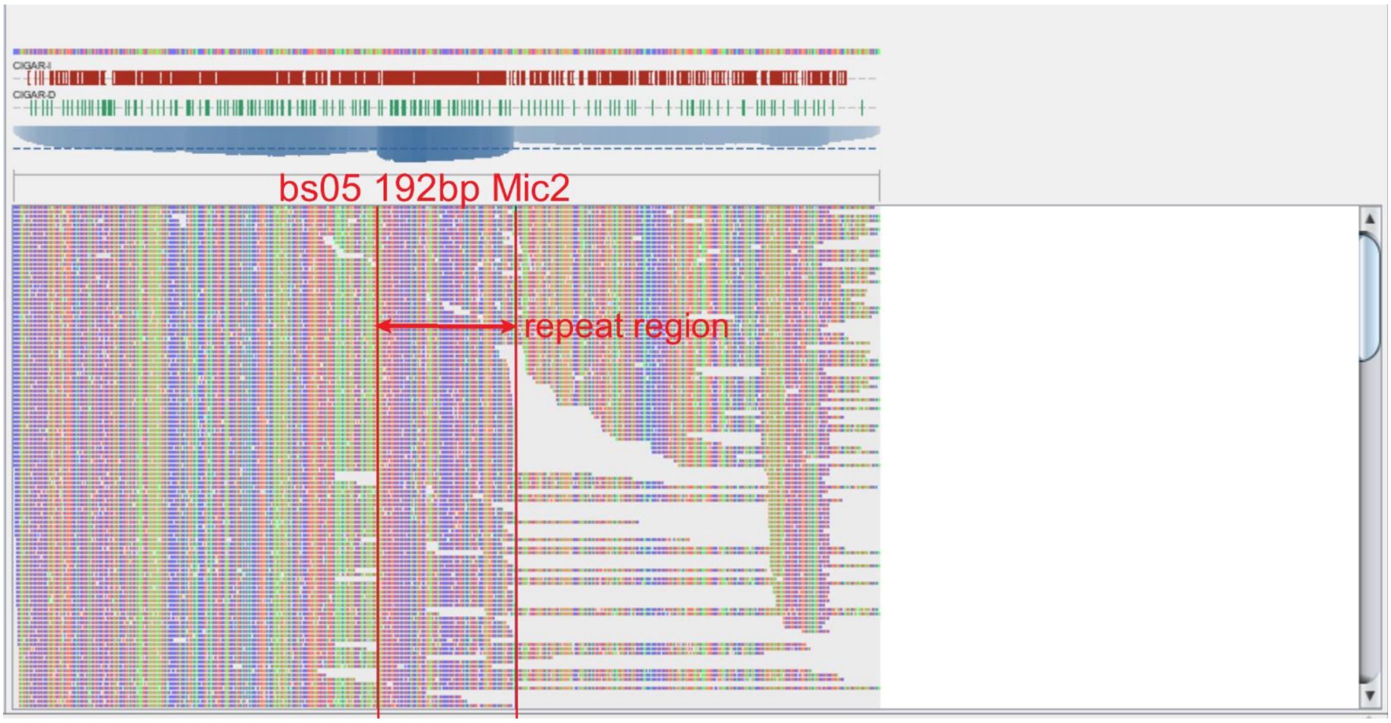
CIGAR-I
CIGAR-D
bs05 192bp Mic2
repeat region

f

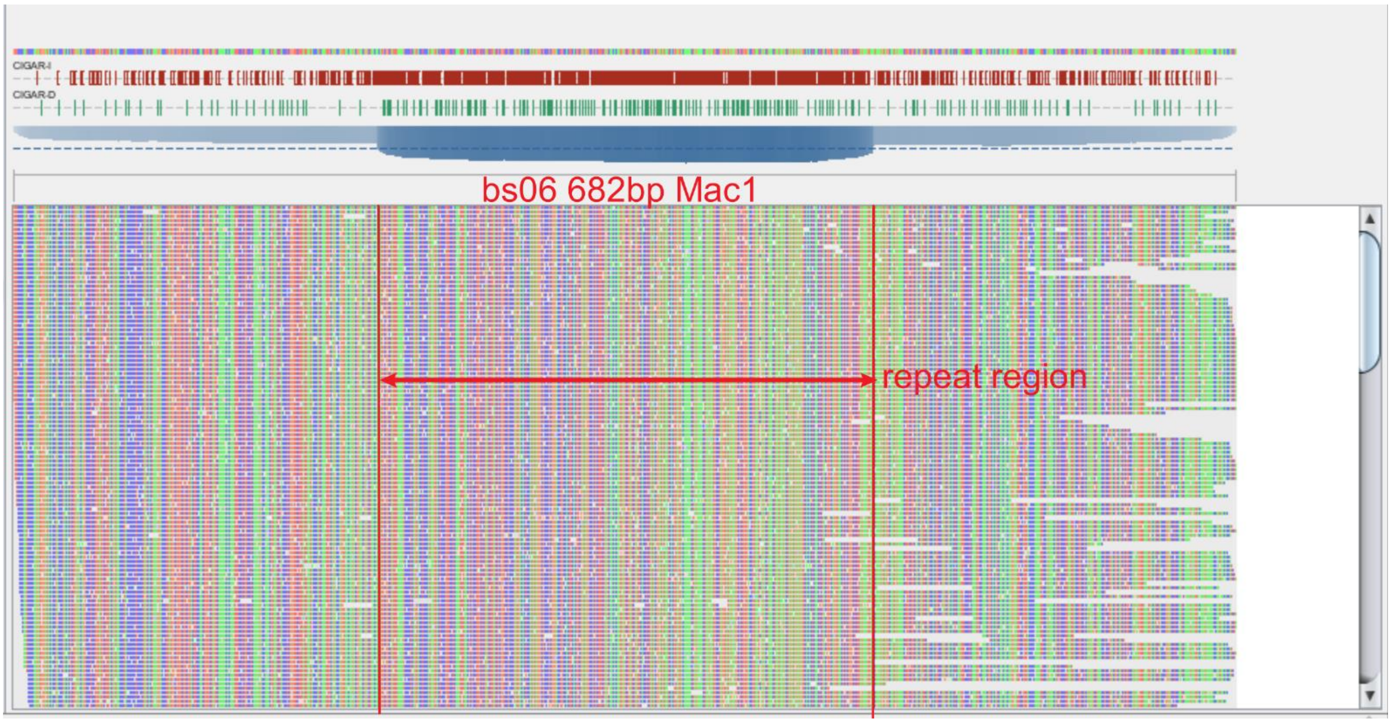

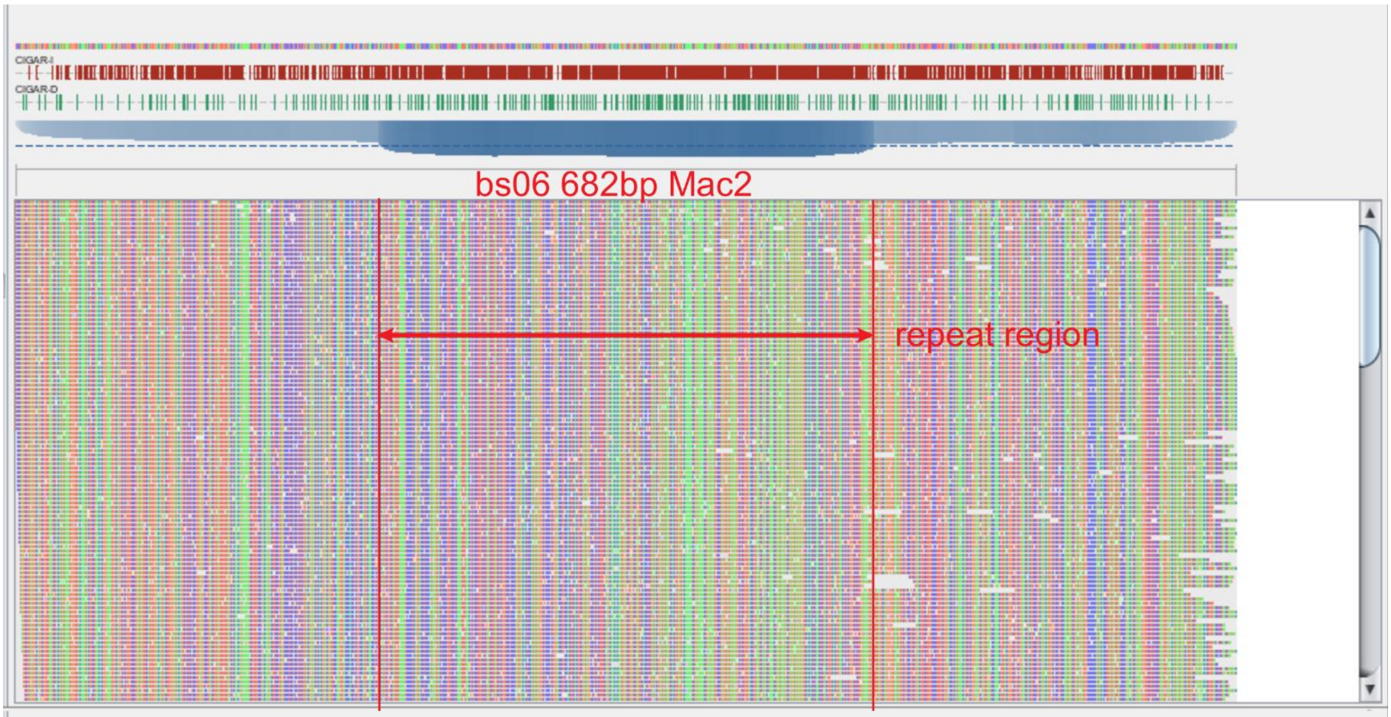
CIGAR-I
CIGAR-D
bs06 682bp Mac2
repeat region

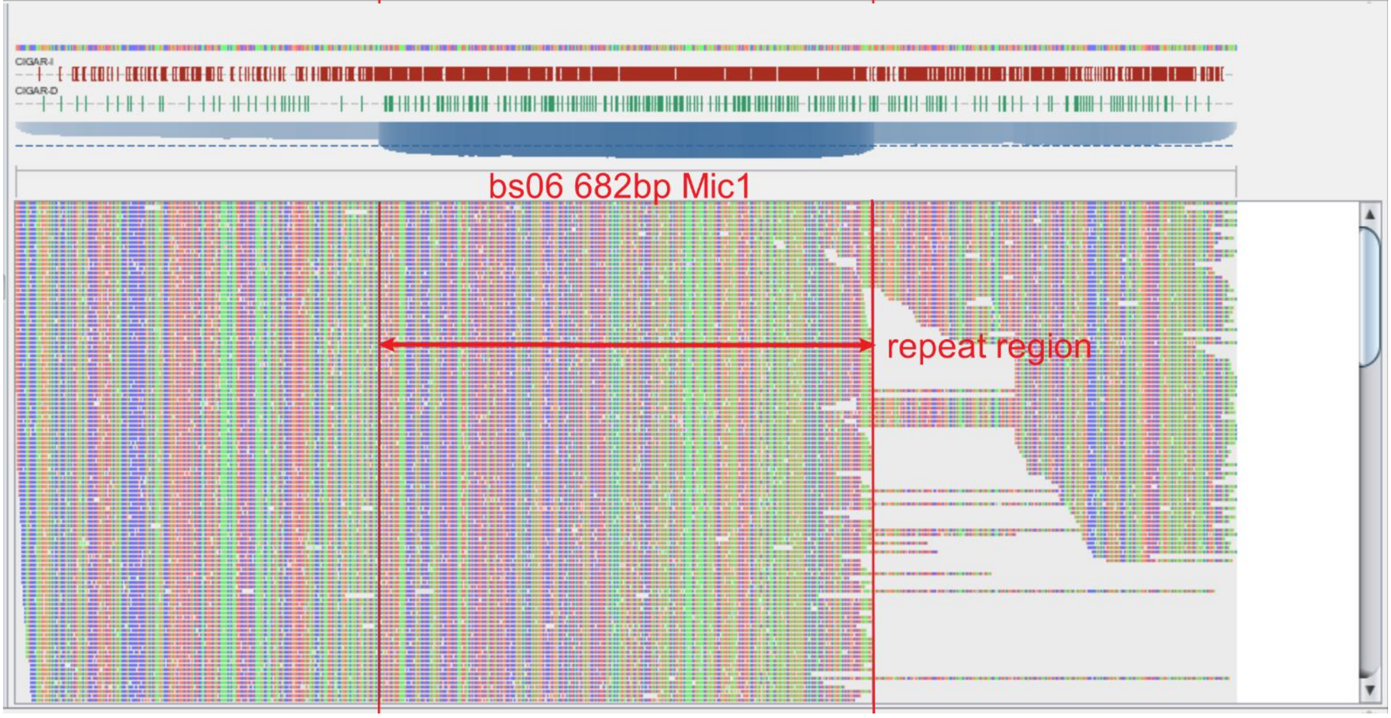
CIGAR-I
CIGAR-D
bs06 682bp Mic1
repeat region

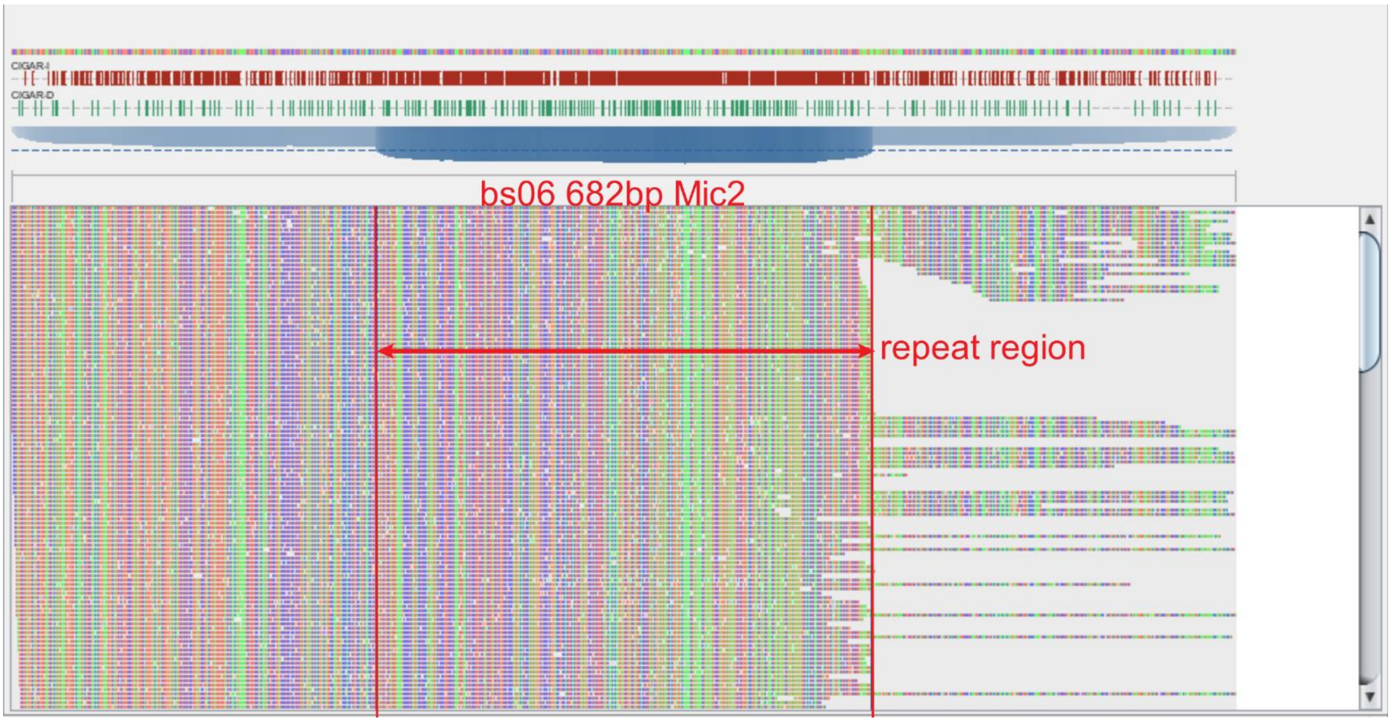
CIGAR-I
CIGAR-D
bs06 682bp Mic2
repeat region

g

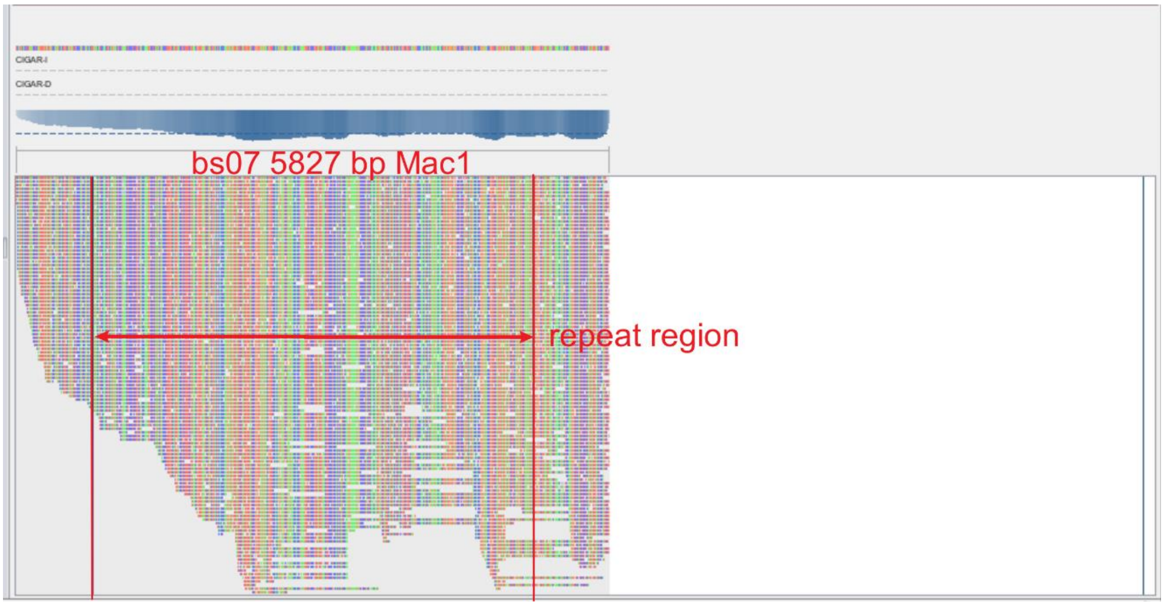

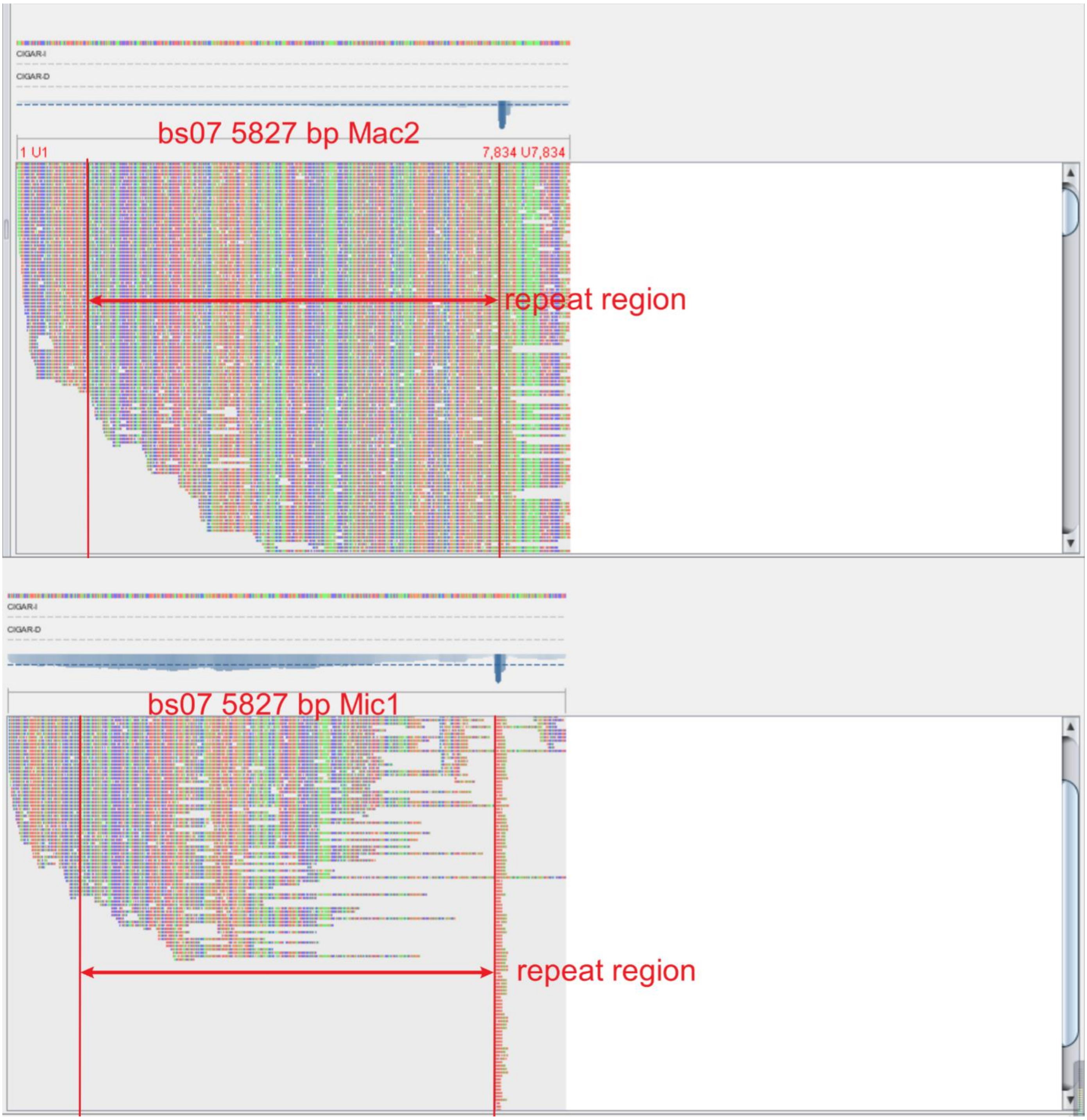
CIGAR-I
CIGAR-D
bs07 5827 bp Mac2
1 U1
7,834 U7,834
repeat region
CIGAR-I
CIGAR-D
bs07 5827 bp Mic1
repeat region

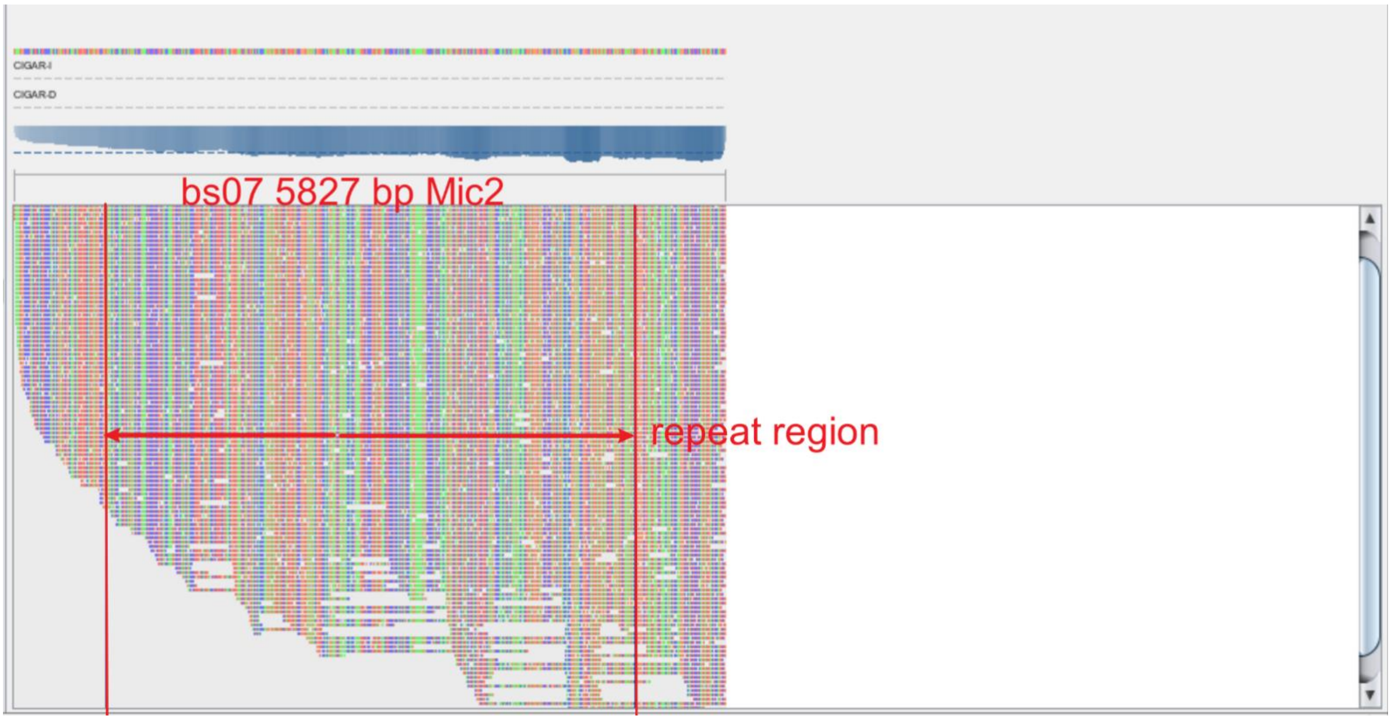
CIGAR-I
CIGAR-D
bs07 5827 bp Mic2
repeat region

Figure S3. Mapping results of PacBio and Illumina reads to the MC1 and MC2 of S. miltiorrhiza. Panels a and b show the mapping results of PacBio reads to MC1 and MC2, respectively. Panels c and d show the mapping results of all Illumina reads to MC1 and MC2, respectively. The X- and Y-axis show the nucleotide position and corresponding coverage depth, respectively.

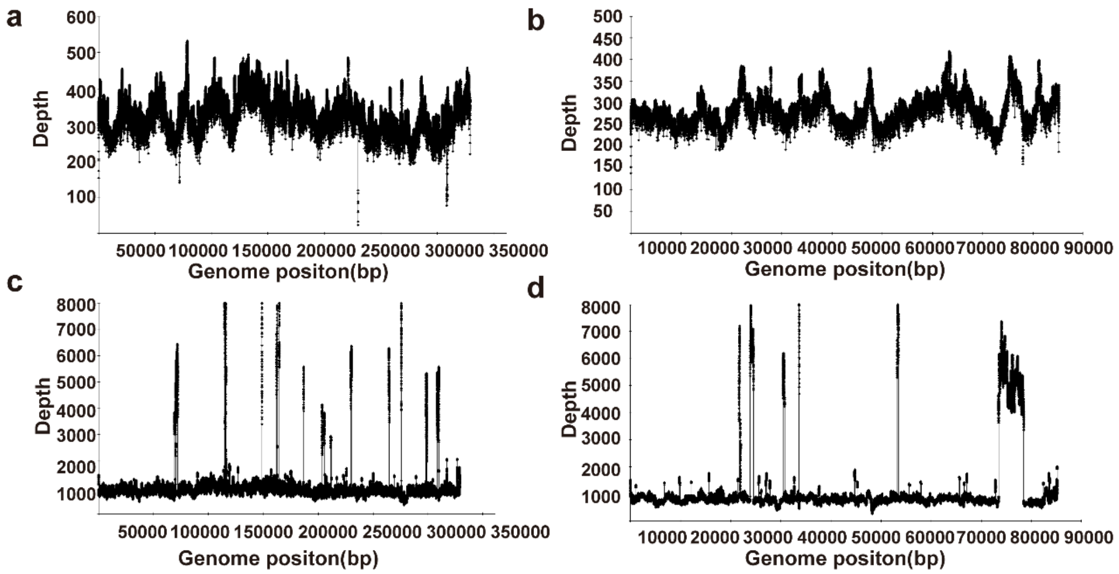

Figure S4. Comparison of the two assemblies of the mitogenome of S. miltiorrhiza. Comparison of the sequences of MC1 (A), MC2 (B), and NC_023209.1. The X-axis shows the nucleotide sequence of MC1, and the Y-axis presents the nucleotide sequence of NC_023209.1. The largest collinear block between them was 66,778 bp in length and is highlighted with a red circle.

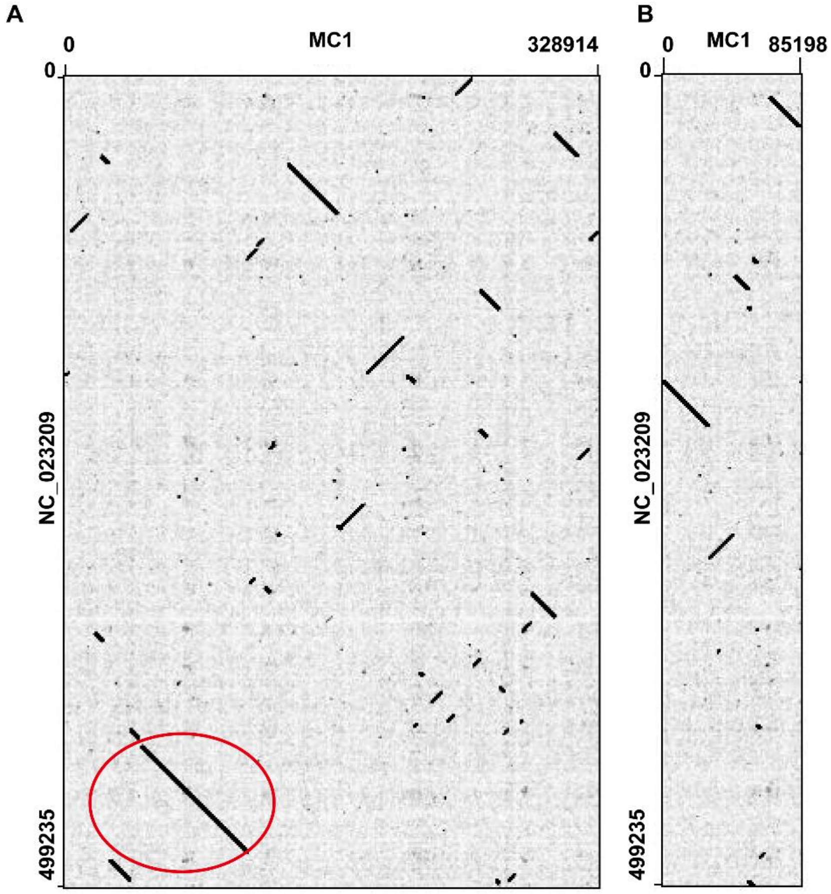

Figure S5. Mapping results of PacBio long reads to the mitogenome of S. miltiorrhiza in GenBank (NC_023209.1). The X-axis shows the nucleotide position, and the Y-axis reveals the corresponding coverage depth. Three large regions not supported by the long reads in this study are highlighted with red squares.

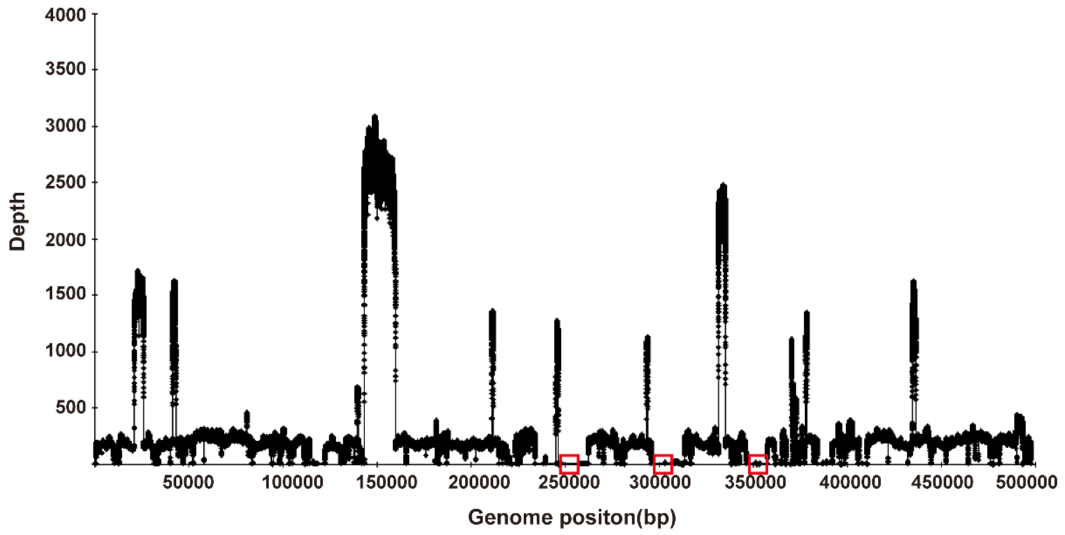

Figure S6. Alignments of long PacBio reads to the Mac and Mic resulted from nine pairs of HSPs (sami-r01 to sami-r09). The nine HSPs were identified using BLASTn with the e-value = 1e-6. The alignment of two HSPs generated a DBS, similar to the ones observed in the unitig graph (Fig. 1). In the presence of homologous recombination, the DBSs formed by the alignment of HSPs and their flanking sequences will also have four conformations, similar to those shown in Fig. 1B. We constructed the sequences corresponding to the four conformations of each DBS for each HSP. We then mapped the long PacBio reads to these sequences. The results are shown in panels a – i for sami-r01 to sami-r09, respectively. We named the conformations having the most supporting reads as Mac1 and Mac2. The reference sequences of Mac1 and Mac2 are reverse-complementary to each other. The alternative conformations were named as Mic1 and Mic2. The figure in each panel can be divided into the top and bottom parts. The top part shows a bird's eye view, whereas the bottom part reveals a base-level view. At the bottom part of the figure, the HSP ID, length of HSP, and conformation name are shown. The boundaries of HSPs are indicated with red vertical lines. The HSPs are shown as the red line having arrows at each end. They were labeled as "repeat region".

a

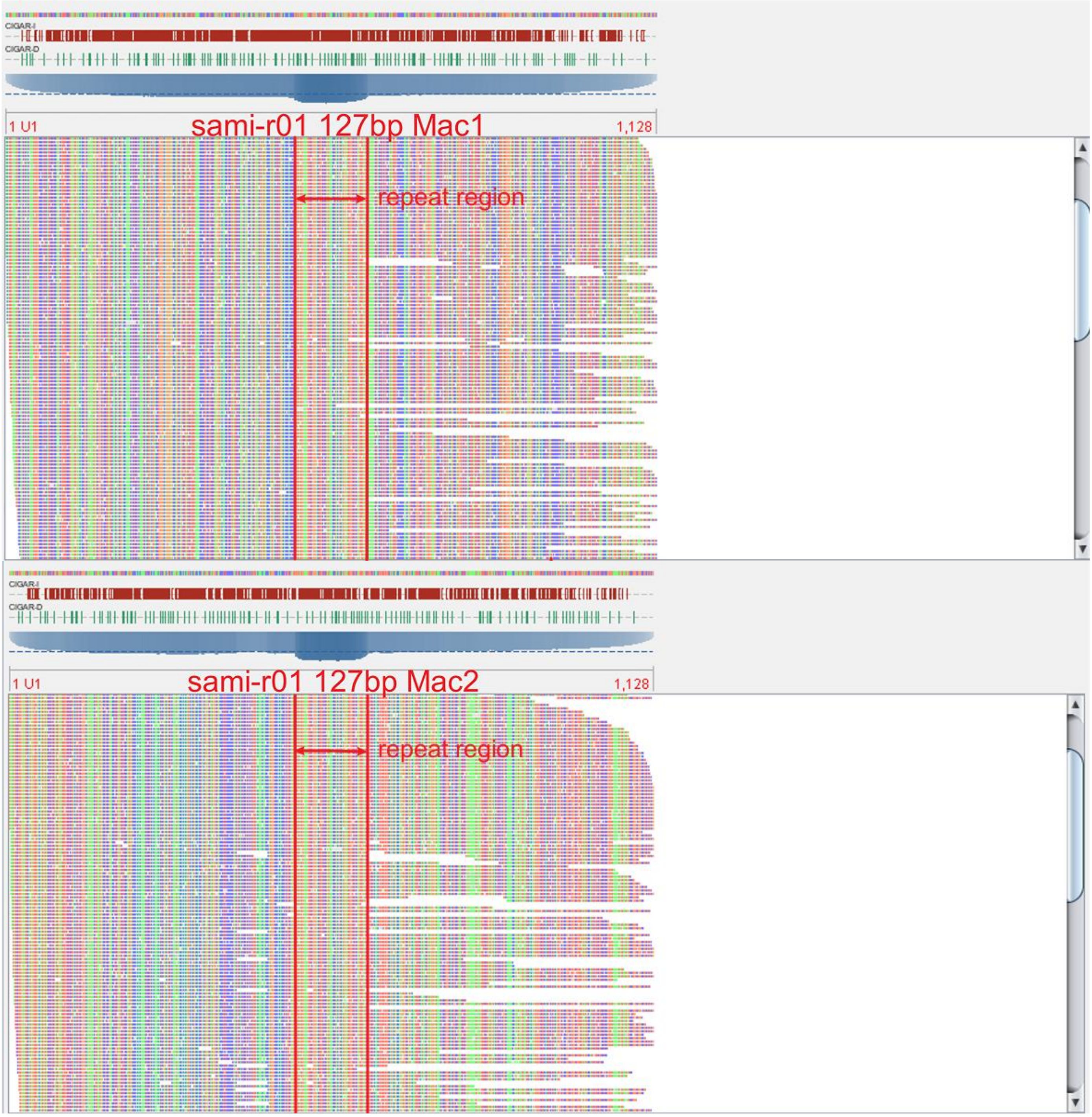

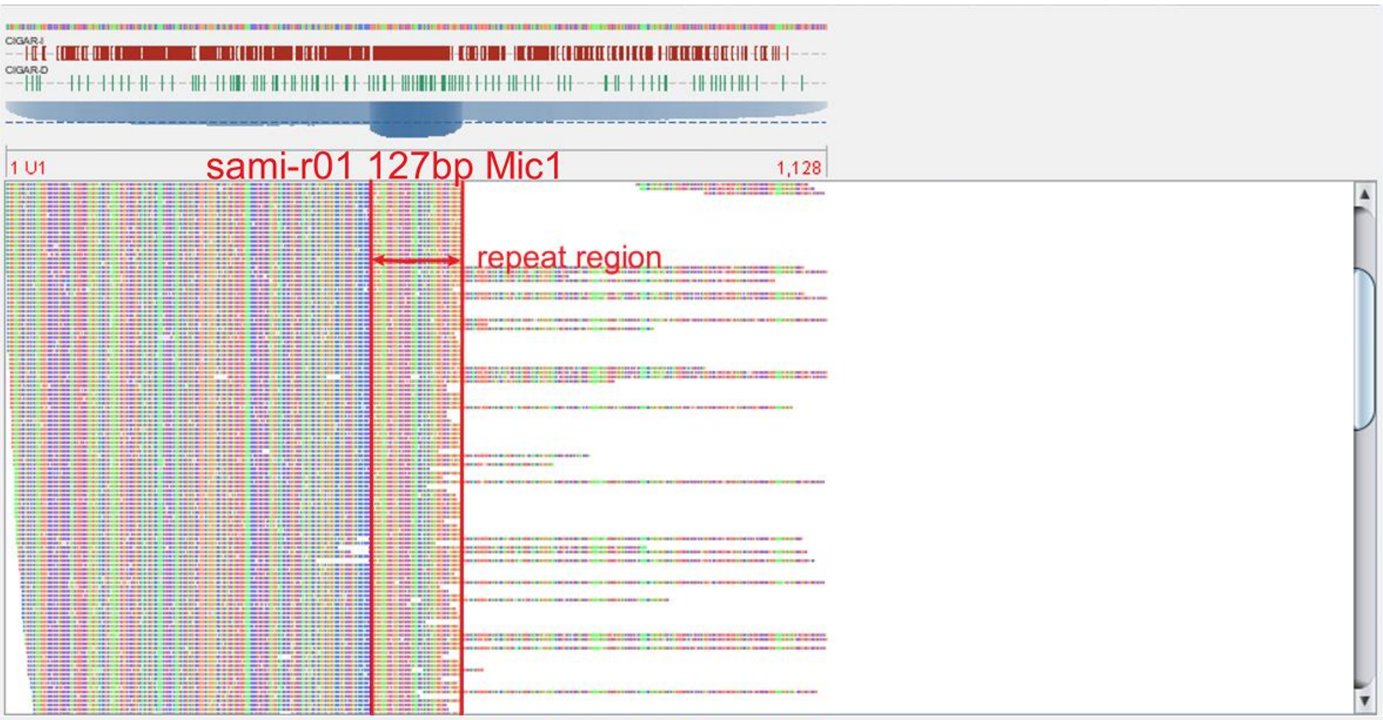
CIGAR-I
CIGAR-D
1 U1
sami-r01 127bp Mic1
1,128
repeat region

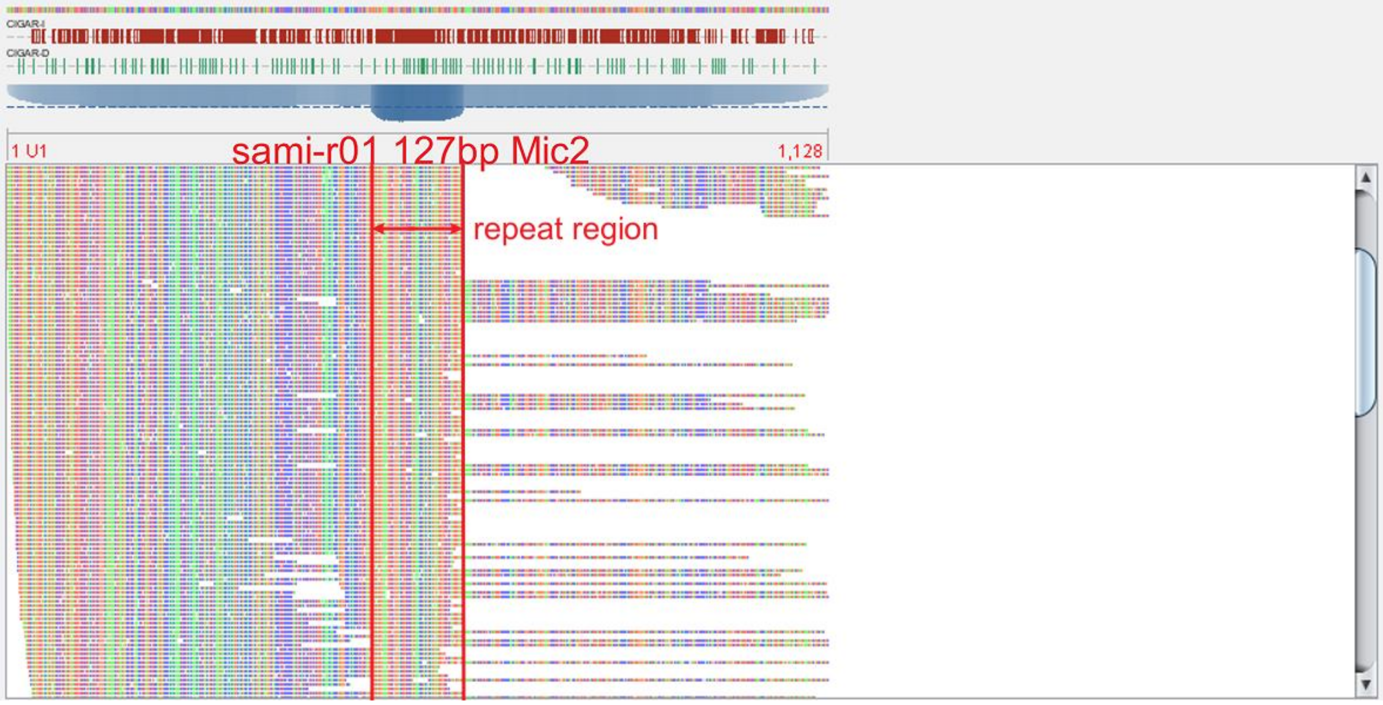
CIGAR-I
CIGAR-D
1 U1
sami-r01 127bp Mic2
1,128
repeat region

b

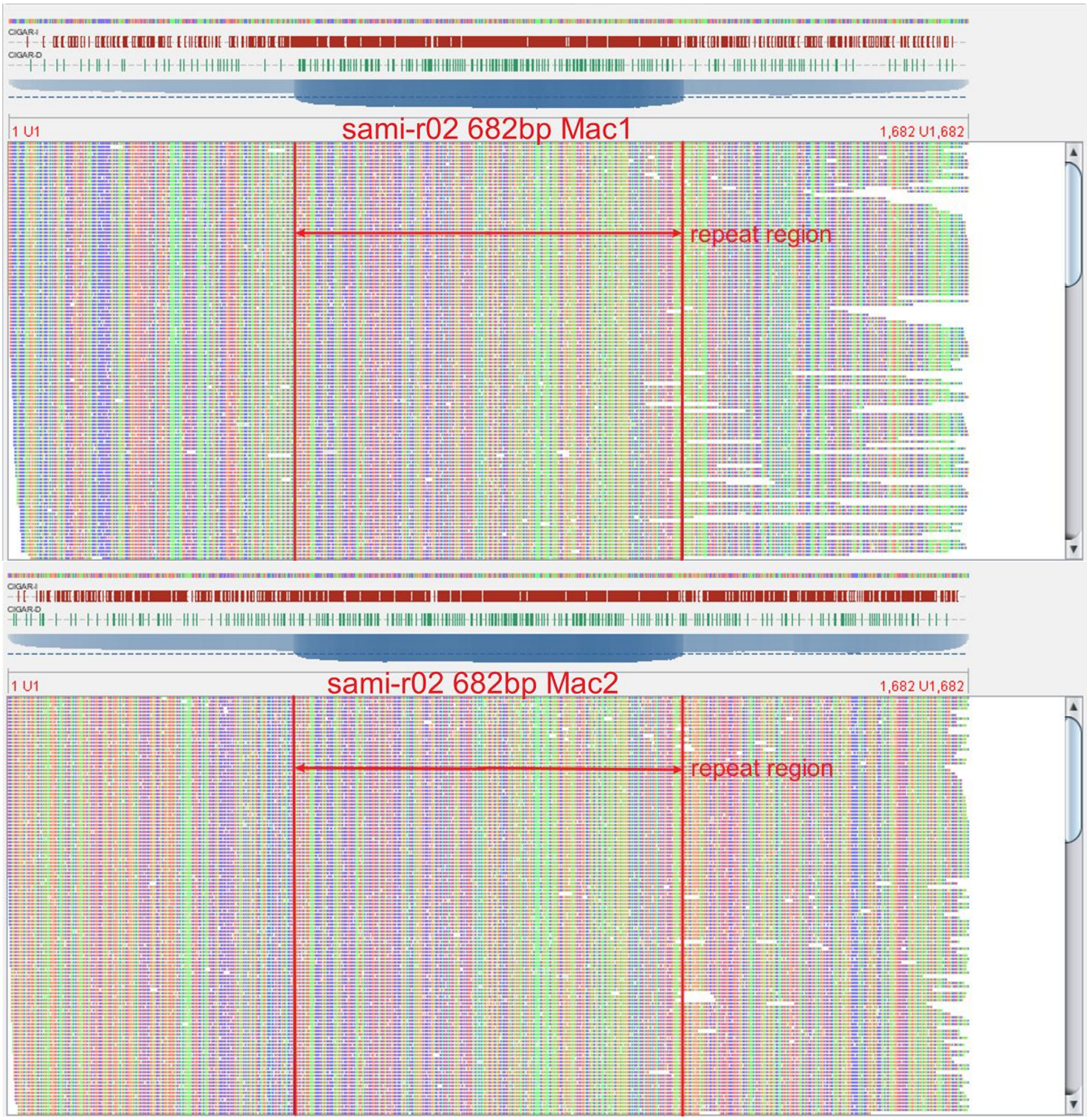

CIGAR-I
CIGAR-D
1 U1
sami-r02 682bp Mac1
1,682 U1,682
repeat region
CIGAR-I
CIGAR-D
1 U1
sami-r02 682bp Mac2
1,682 U1,682
repeat region

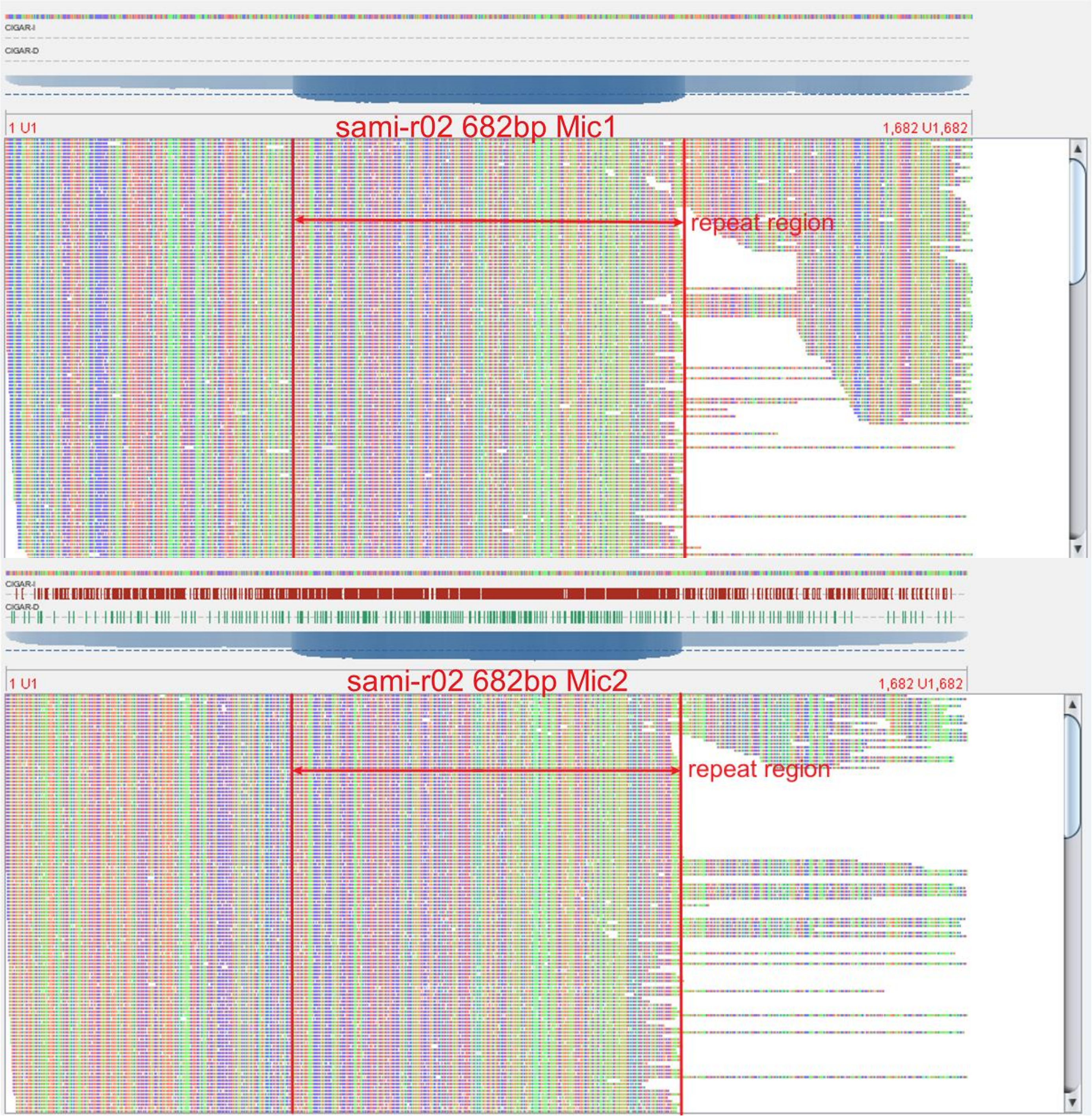
CIGAR-I
CIGAR-D
1 U1
sami-r02 682bp Mic1
1,682 U1,682
repeat region
CIGAR-I
CIGAR-D
1 U1
sami-r02 682bp Mic2
1,682 U1,682
repeat region

c

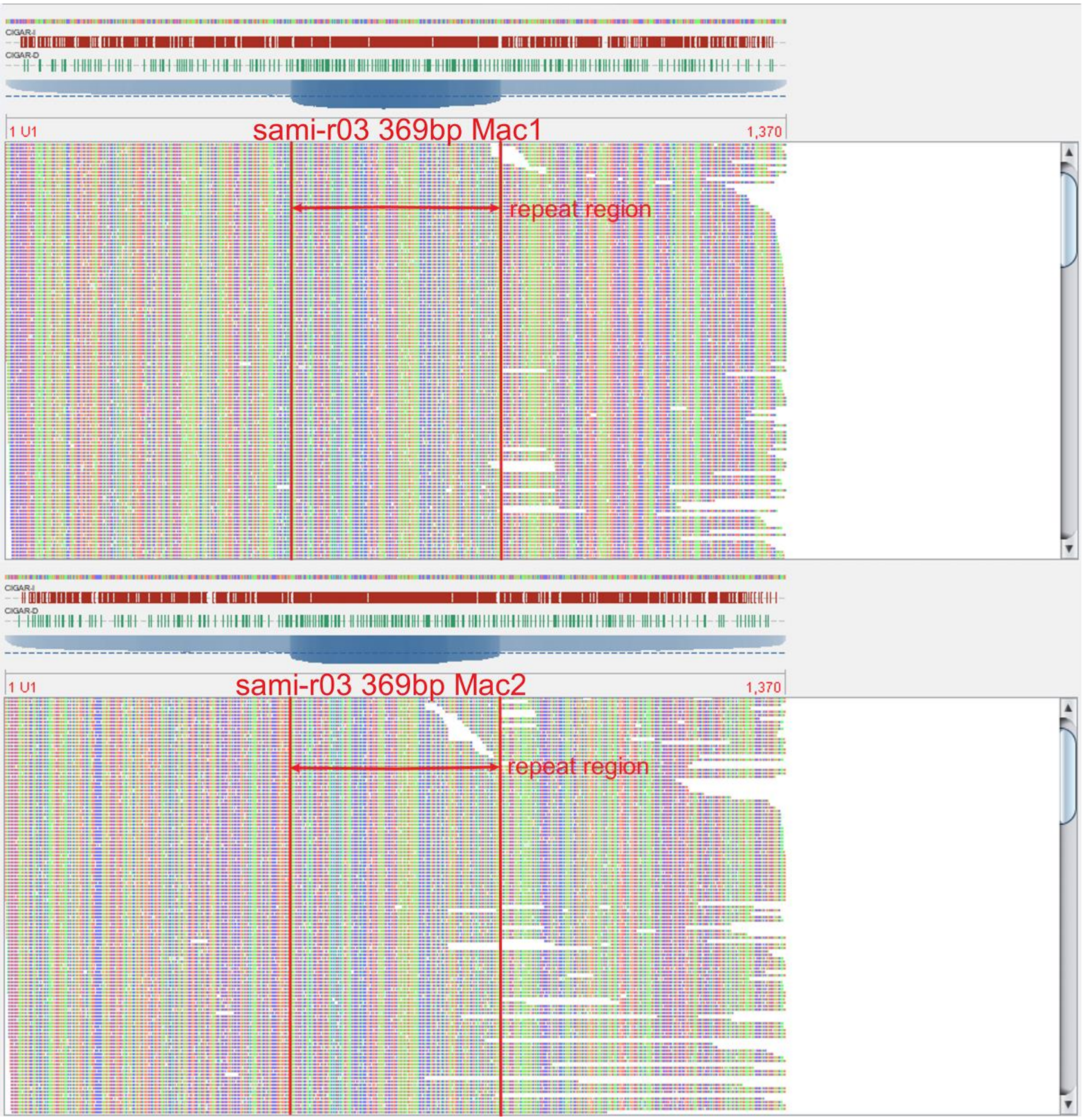
CIGAR-I
CIGAR-D
1 U1
sami-r03 369bp Mac1
1,370
repeat region
CIGAR-I
CIGAR-D
1 U1
sami-r03 369bp Mac2
1,370
repeat region

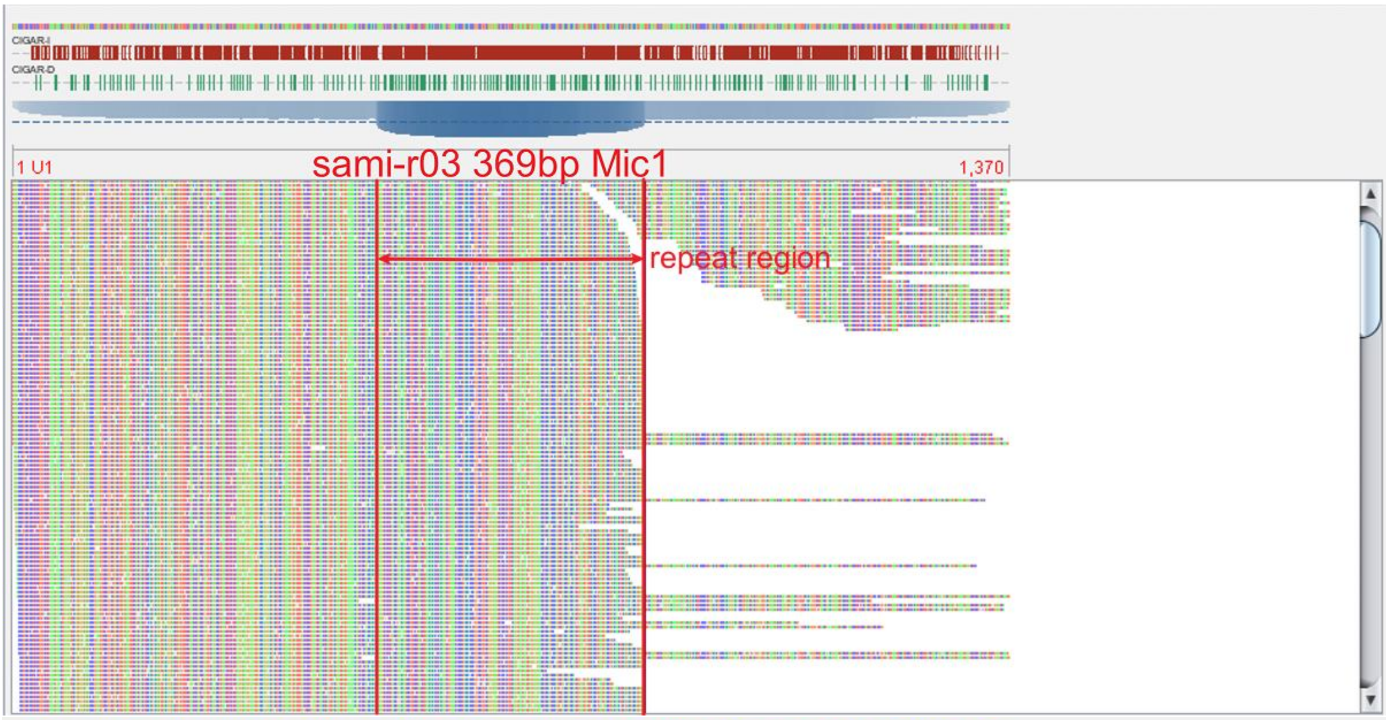
CIGAR-I
CIGAR-D
1 U1
sami-r03 369bp Mic1
1,370
repeat region

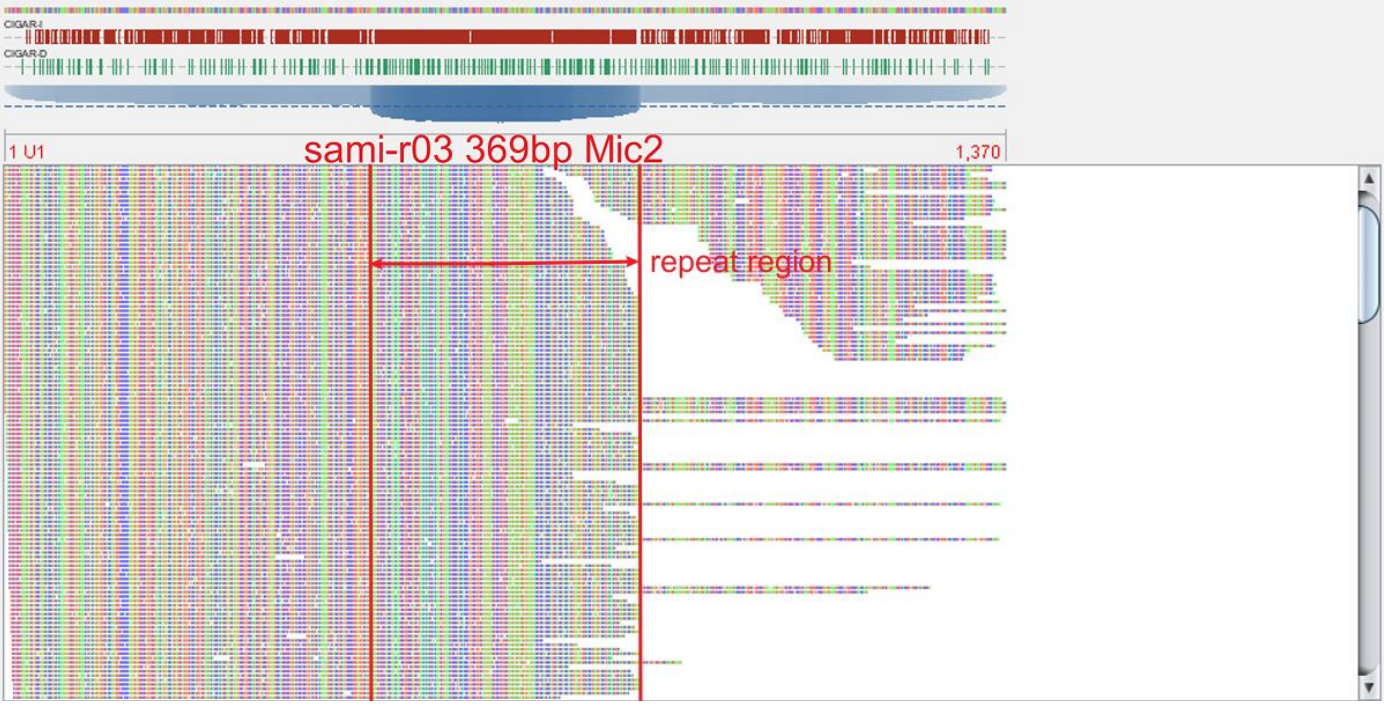
CIGAR-I
CIGAR-D
1 U1
sami-r03 369bp Mic2
1,370
repeat region

d

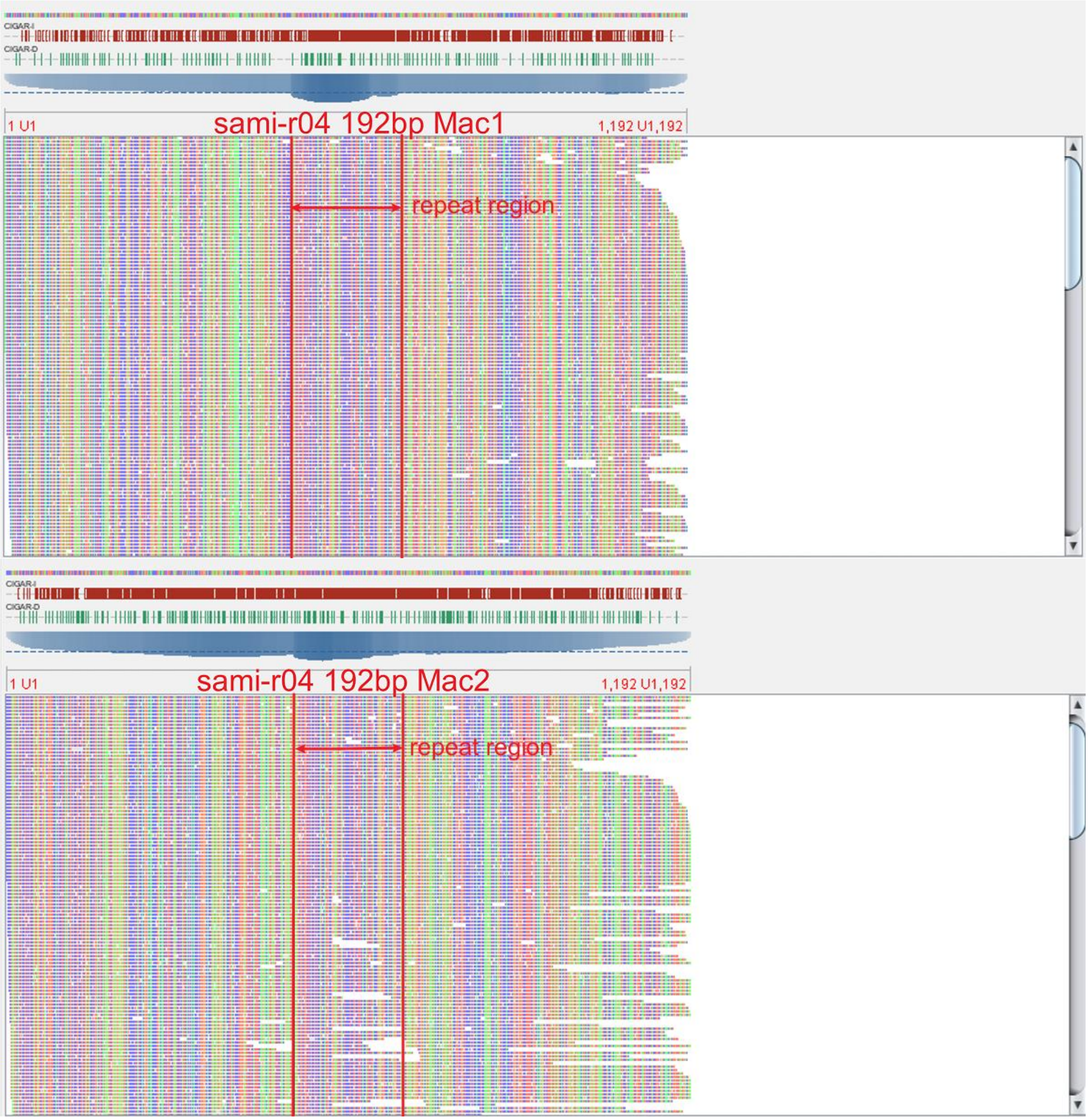
CIGAR-I
CIGAR-D
1 U1
sami-r04 192bp Mac1
1,192 U1,192
repeat region
CIGAR-I
CIGAR-D
1 U1
sami-r04 192bp Mac2
1,192 U1,192
repeat region

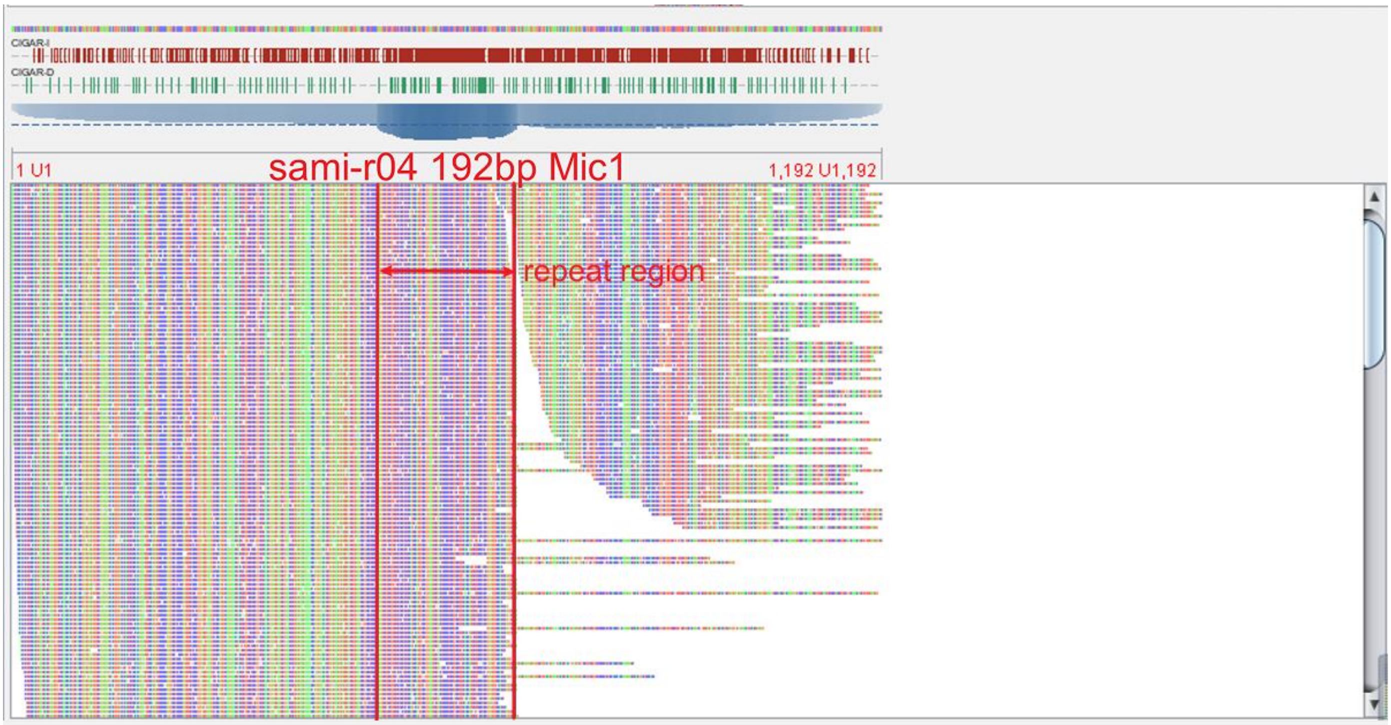
CIGAR-I
CIGAR-D
1 U1
sami-r04 192bp Mic1
1,192 U1,192
repeat region

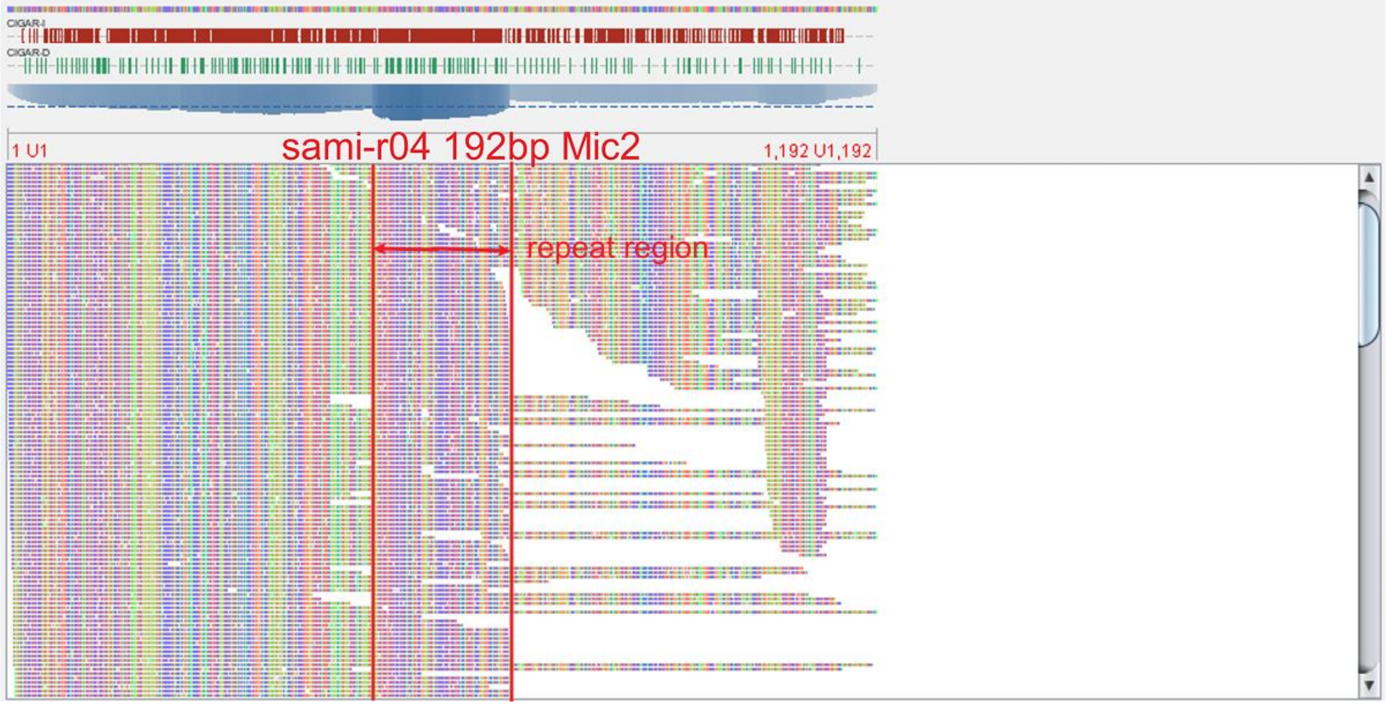
CIGAR-I
CIGAR-D
1 U1
sami-r04 192bp Mic2
1,192 U1,192
repeat region

e

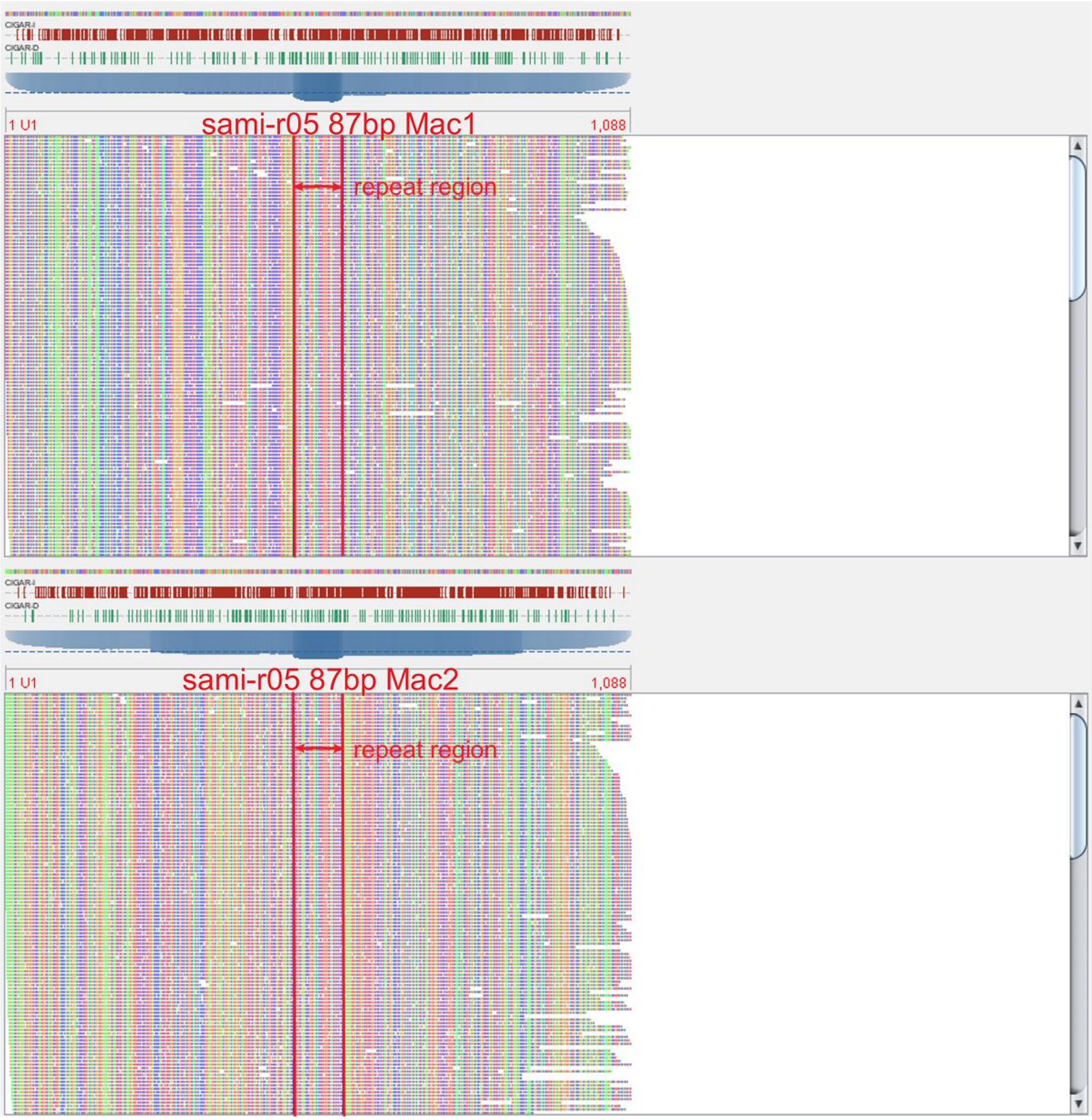
CIGAR-I
CIGAR-D
1 U1
sami-r05 87bp Mac1
1,088
repeat region
CIGAR-I
CIGAR-D
1 U1
sami-r05 87bp Mac2
1,088
repeat region

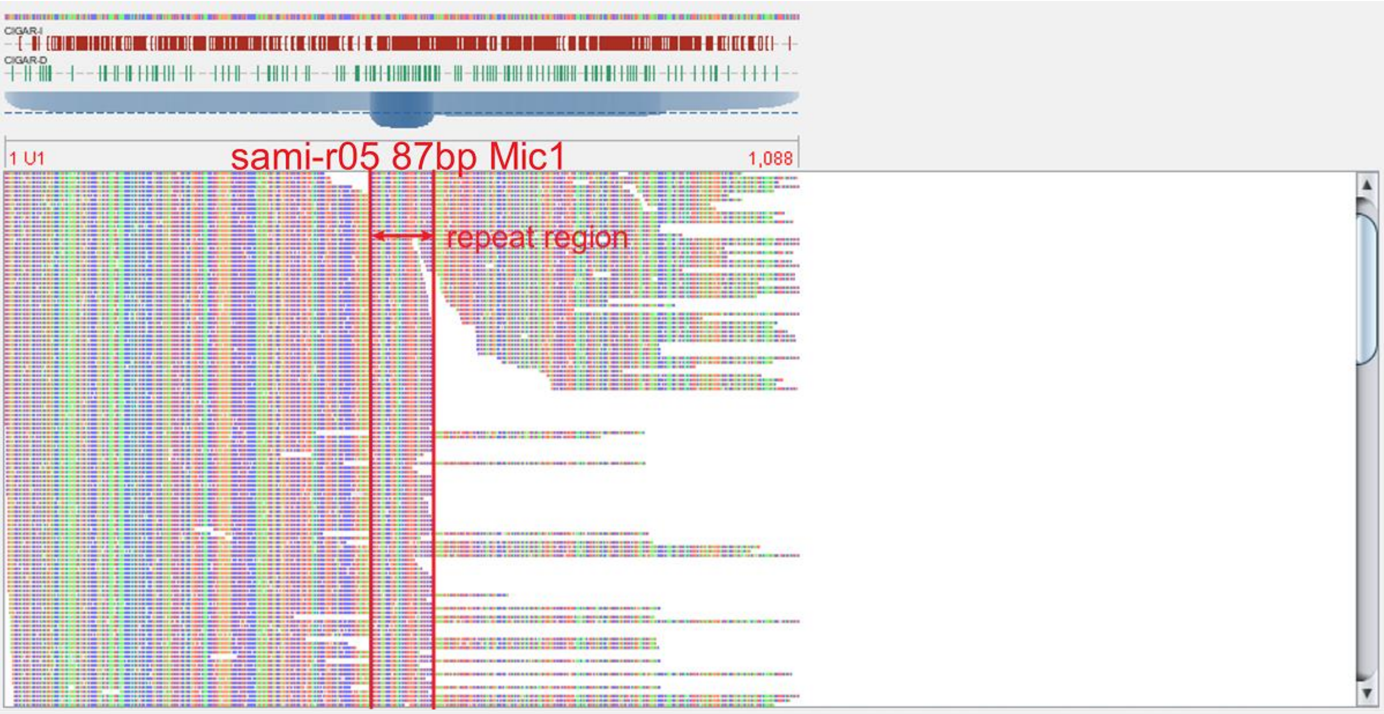
CIGAR-I
CIGAR-D
1 U1
sami-r05 87bp Mic1
1,088
repeat region

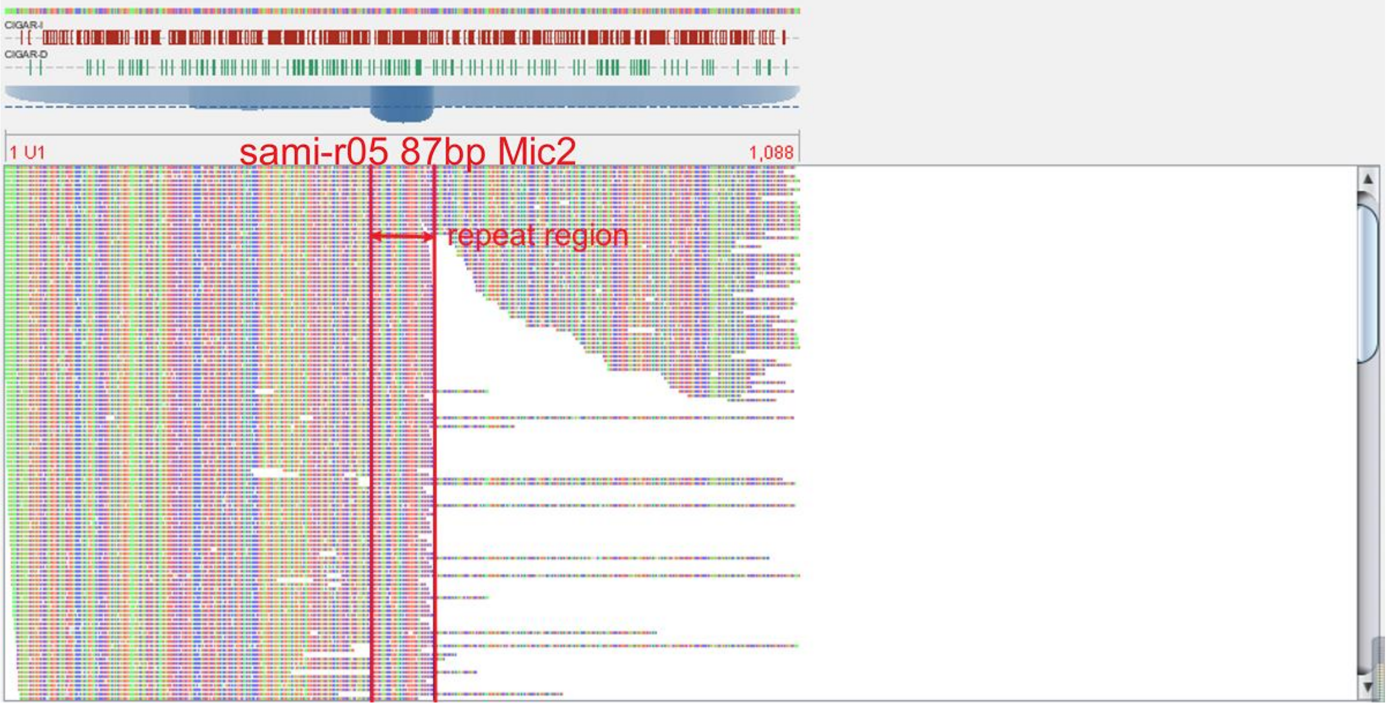
CIGAR-I
CIGAR-D
1 U1
sami-r05 87bp Mic2
1,088
repeat region

f

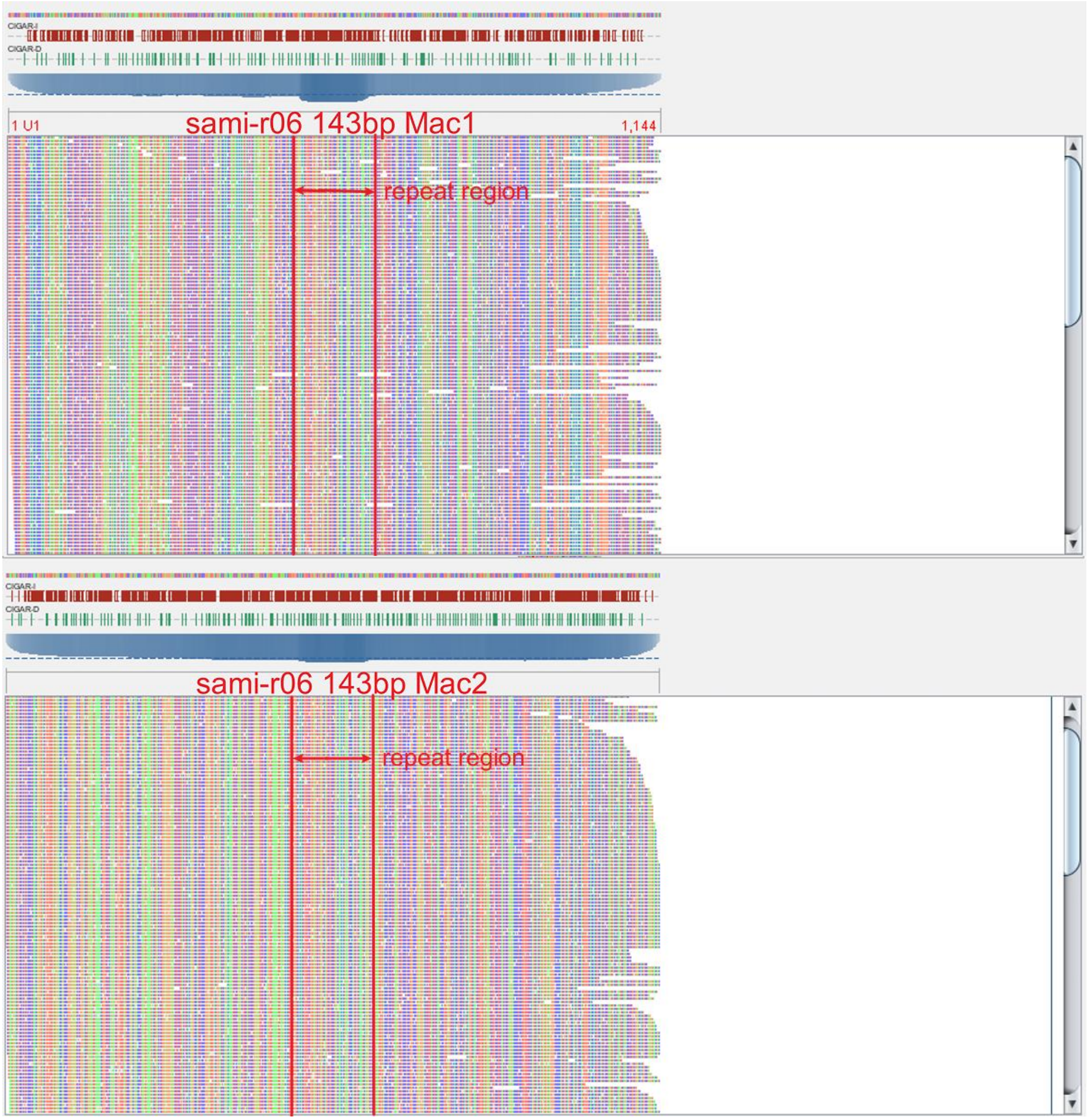
CIGAR-I
CIGAR-D
1 U1
sami-r06 143bp Mac1
1,144
repeat region
CIGAR-I
CIGAR-D
sami-r06 143bp Mac2
repeat region

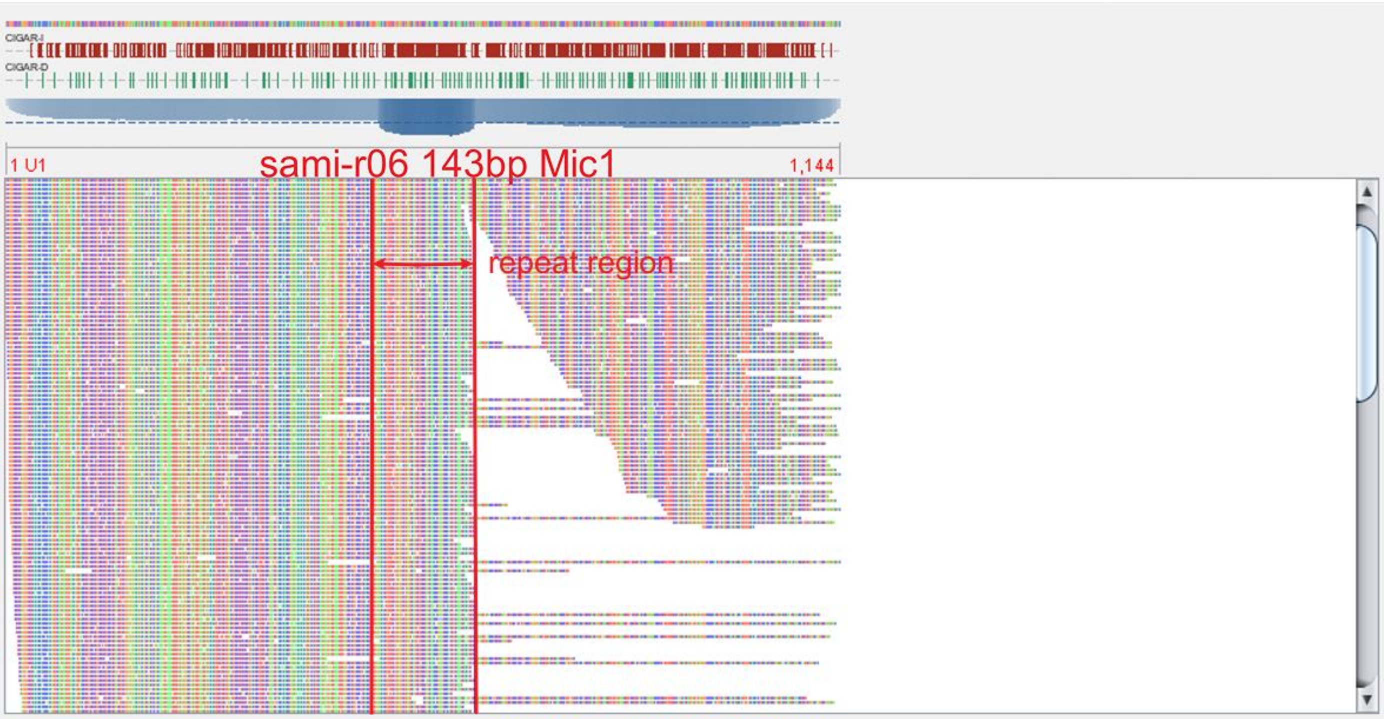
CIGAR-I
CIGAR-D
1 U1
sami-r06 143bp Mic1
1,144
repeat region

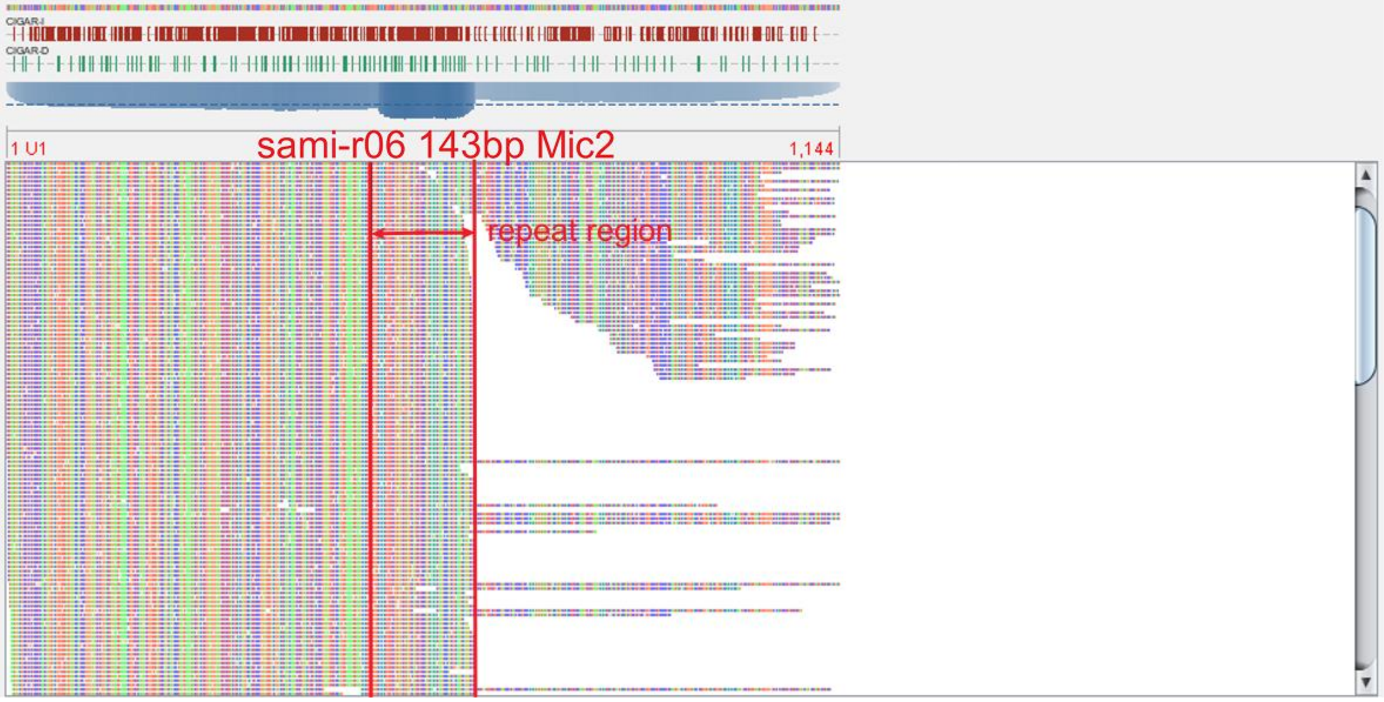
CIGAR-I
CIGAR-D
1 U1
sami-r06 143bp Mic2
1,144
repeat region

g

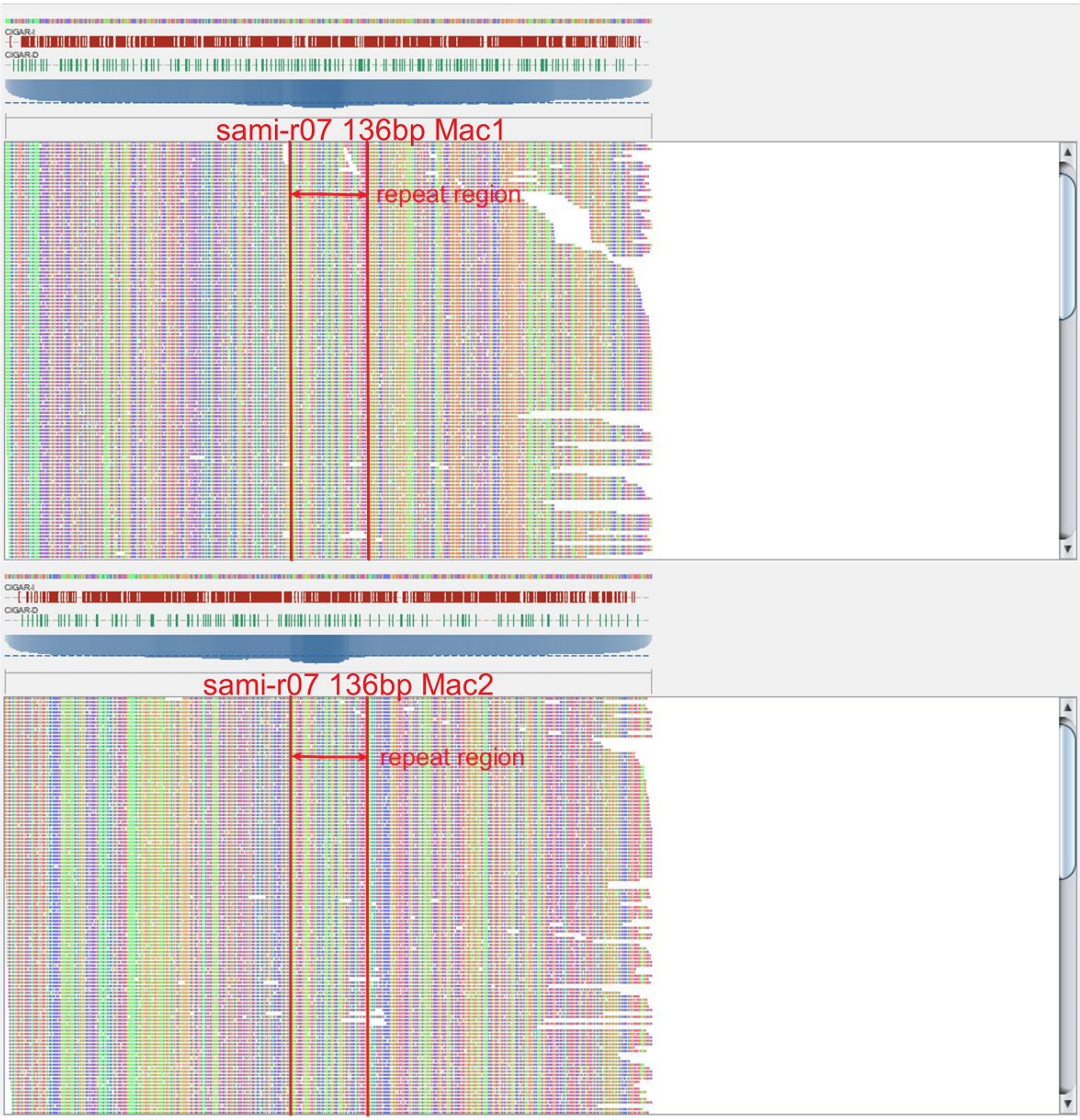
CIGAR-I
CIGAR-D
sami-r07 136bp Mac1
repeat region
CIGAR-I
CIGAR-D
sami-r07 136bp Mac2
repeat region

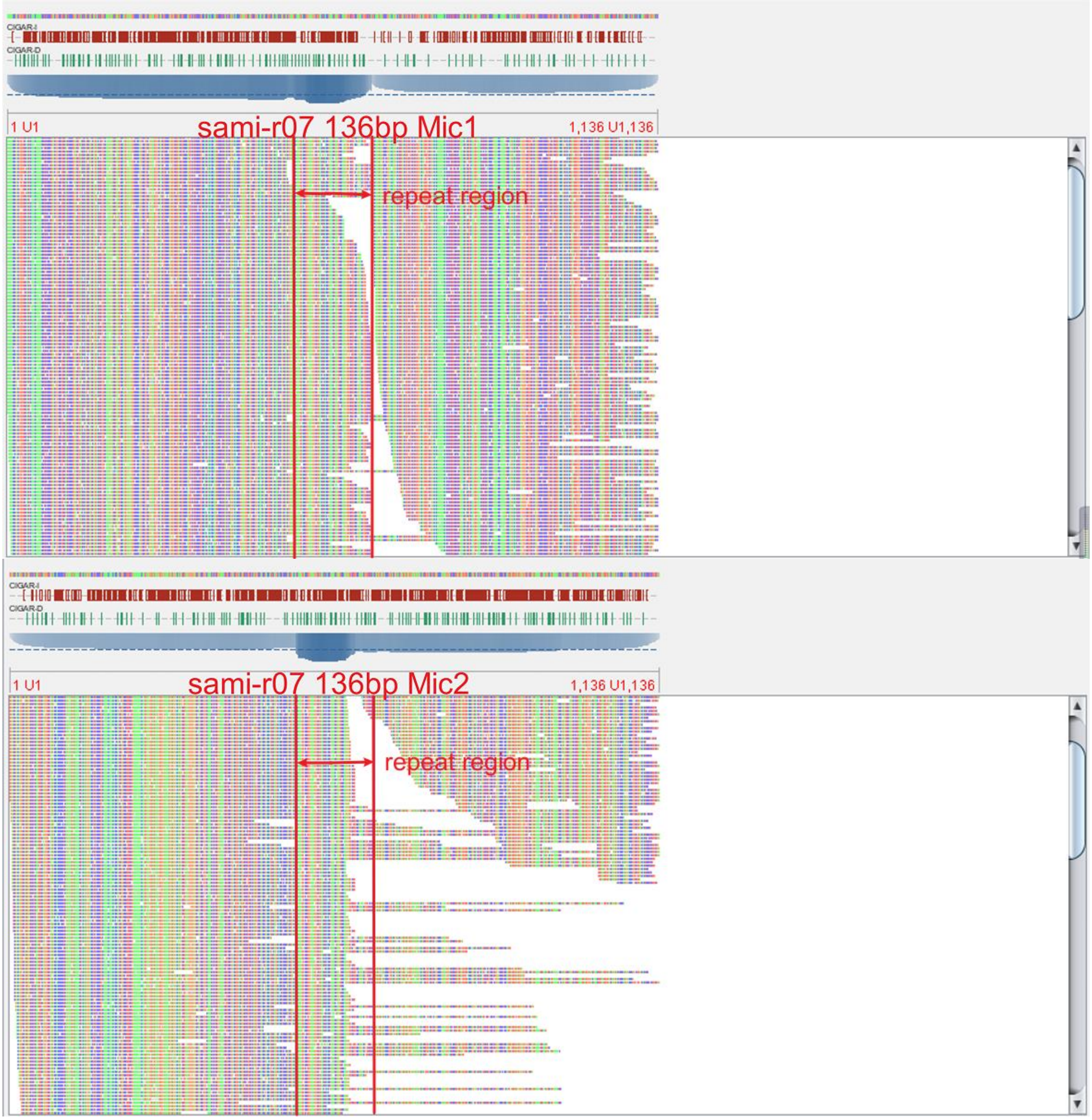
CIGAR-I
CIGAR-D
1 U1
sami-r07 136bp Mic1
1,136 U1,136
repeat region
CIGAR-I
CIGAR-D
1 U1
sami-r07 136bp Mic2
1,136 U1,136
repeat region

h

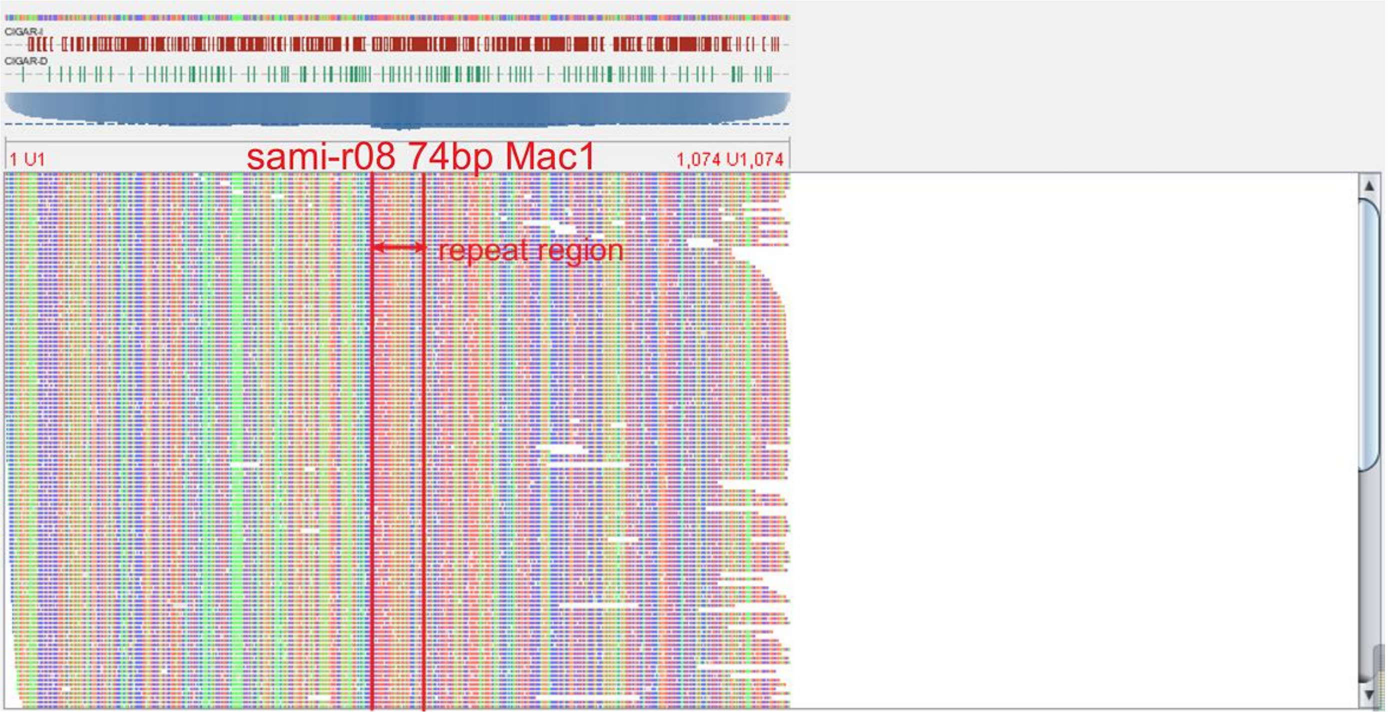

CIGAR-I
CIGAR-D
1 U1
sami-r08 74bp Mac1
1,074 U1,074
repeat region

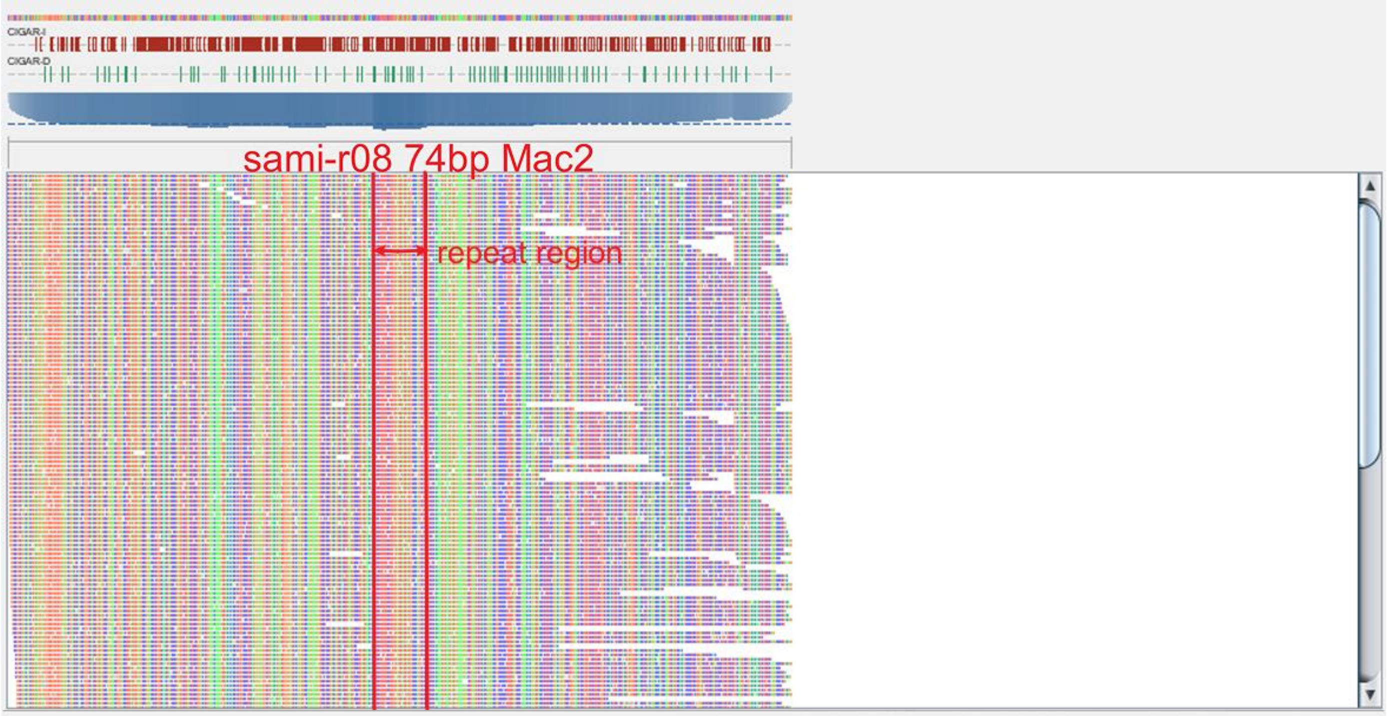
CIGAR-I
CIGAR-D
sami-r08 74bp Mac2
repeat region

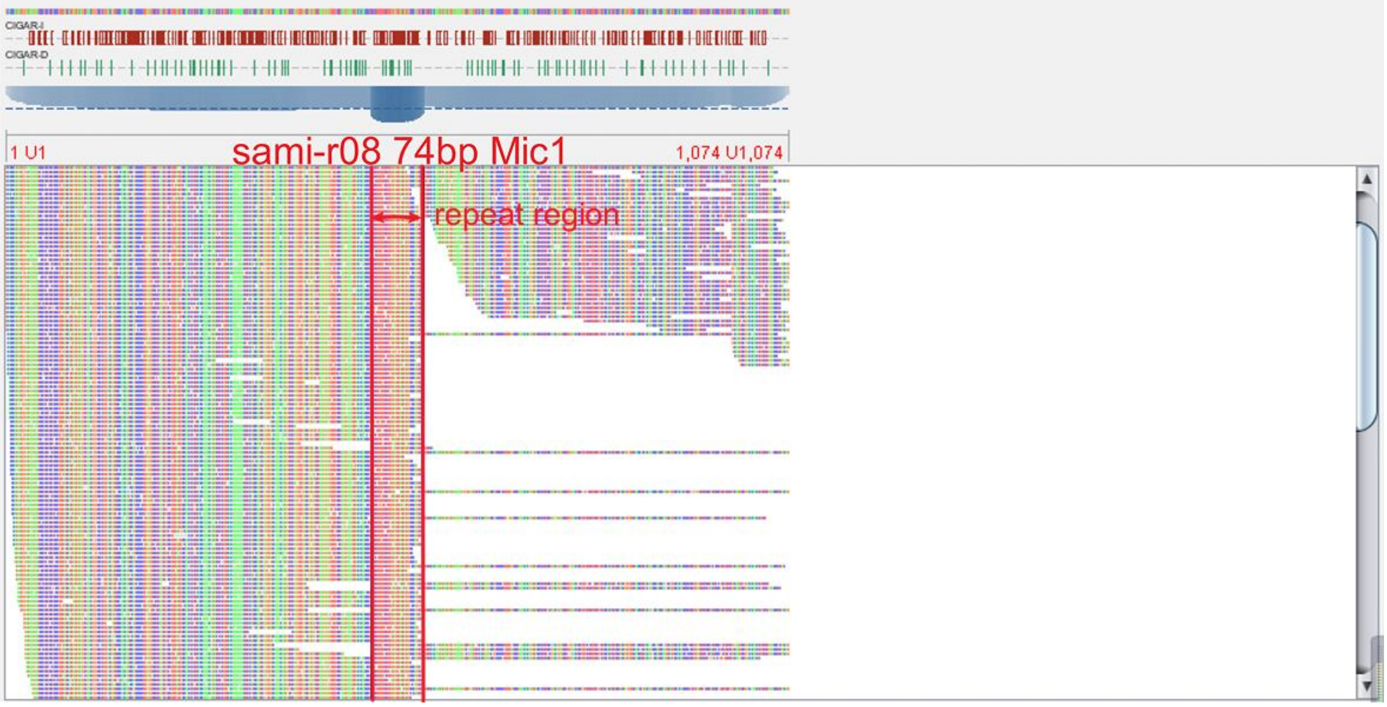
CIGAR-I
CIGAR-D
1 U1
sami-r08 74bp Mic1
1,074 U1,074
repeat region

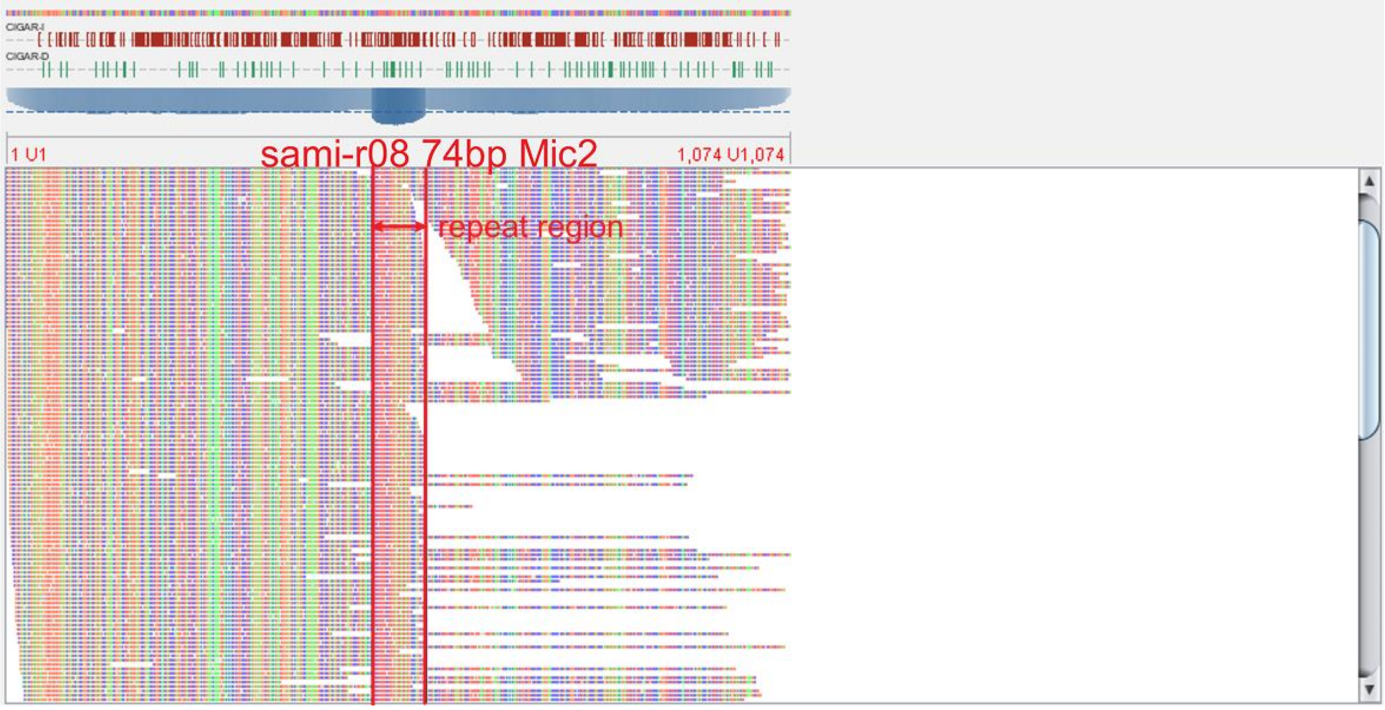

CIGAR-I
CIGAR-D
1 U1
sami-r08 74bp Mic2
1,074 U1,074
repeat region

i

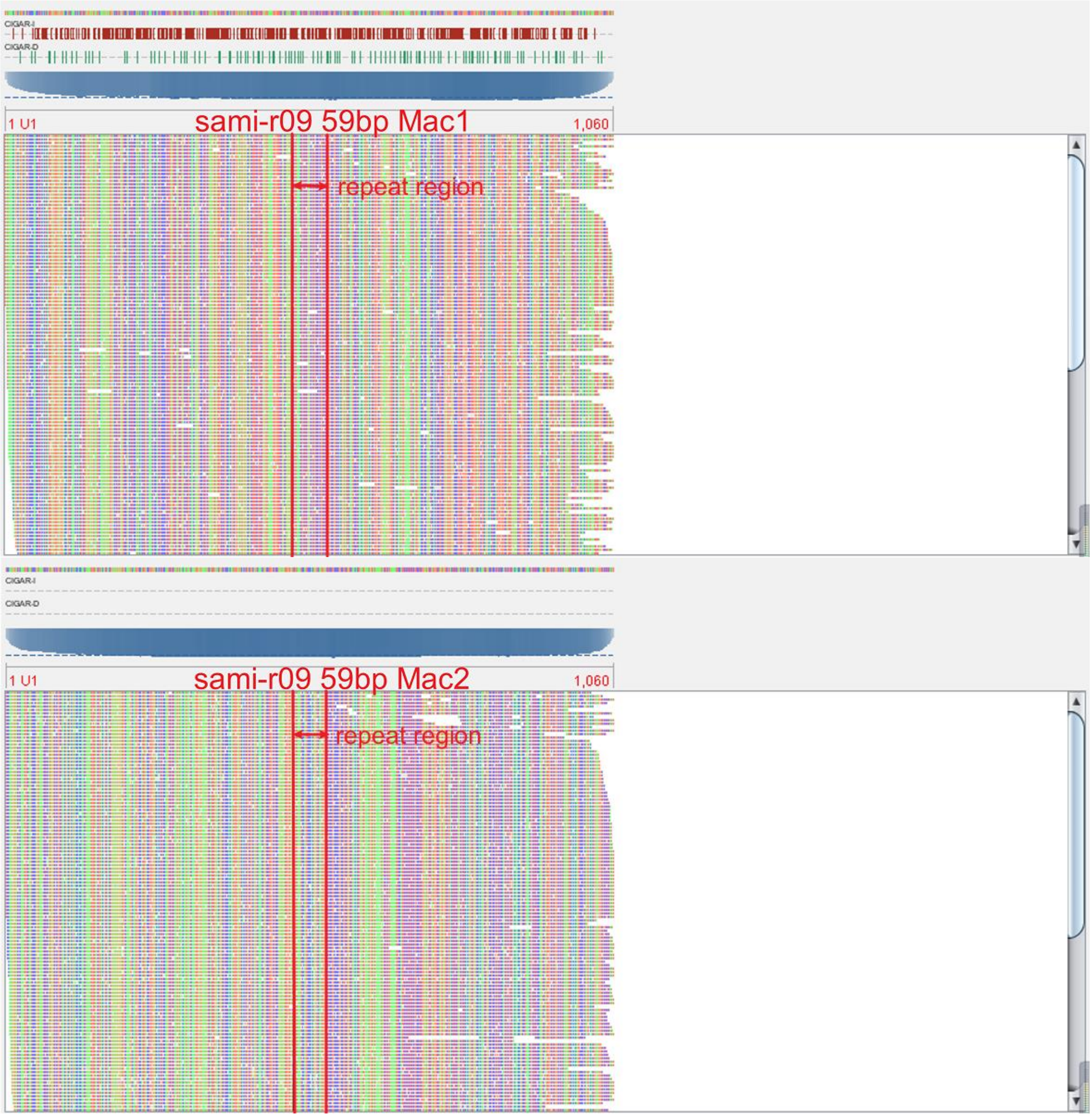
CIGAR-I
CIGAR-D
1 U1
sami-r09 59bp Mac1
1,060
repeat region
CIGAR-I
CIGAR-D
1 U1
sami-r09 59bp Mac2
1,060
repeat region

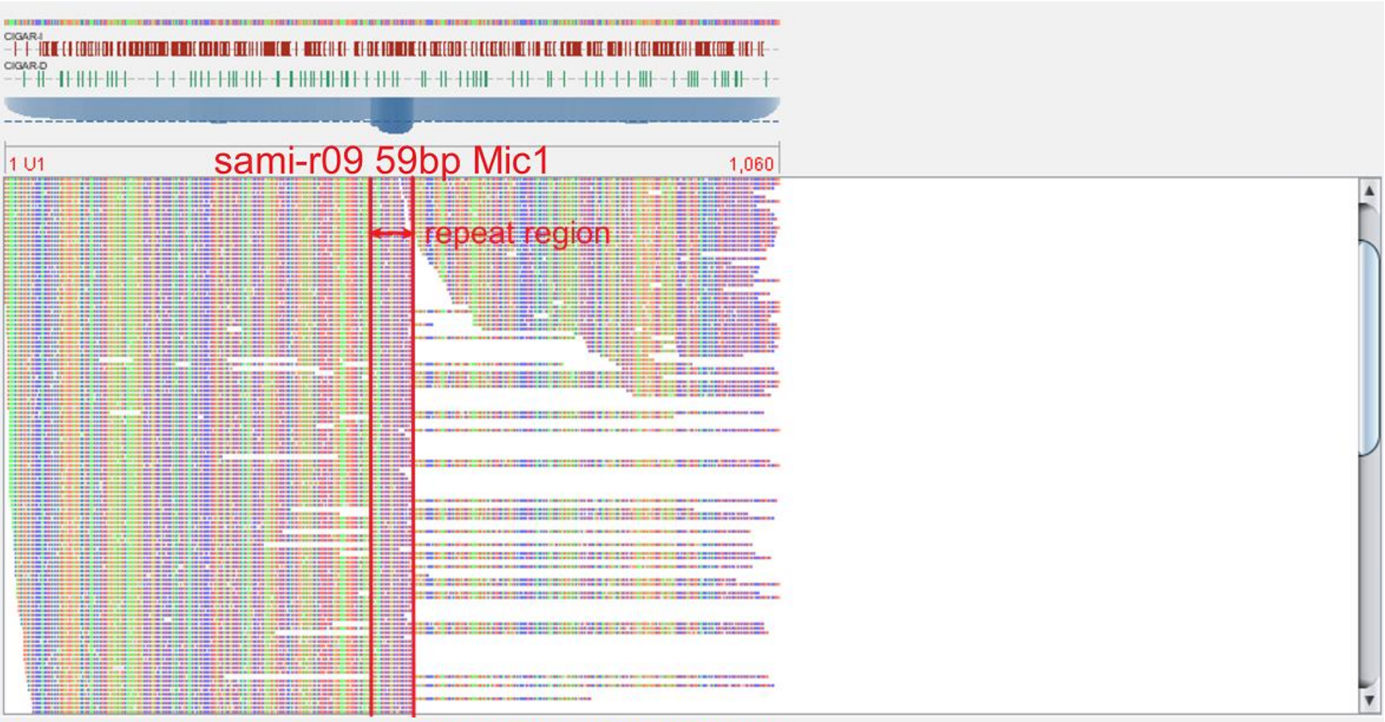
CIGAR-I
CIGAR-D
1 U1
sami-r09 59bp Mic1
1,060
repeat region

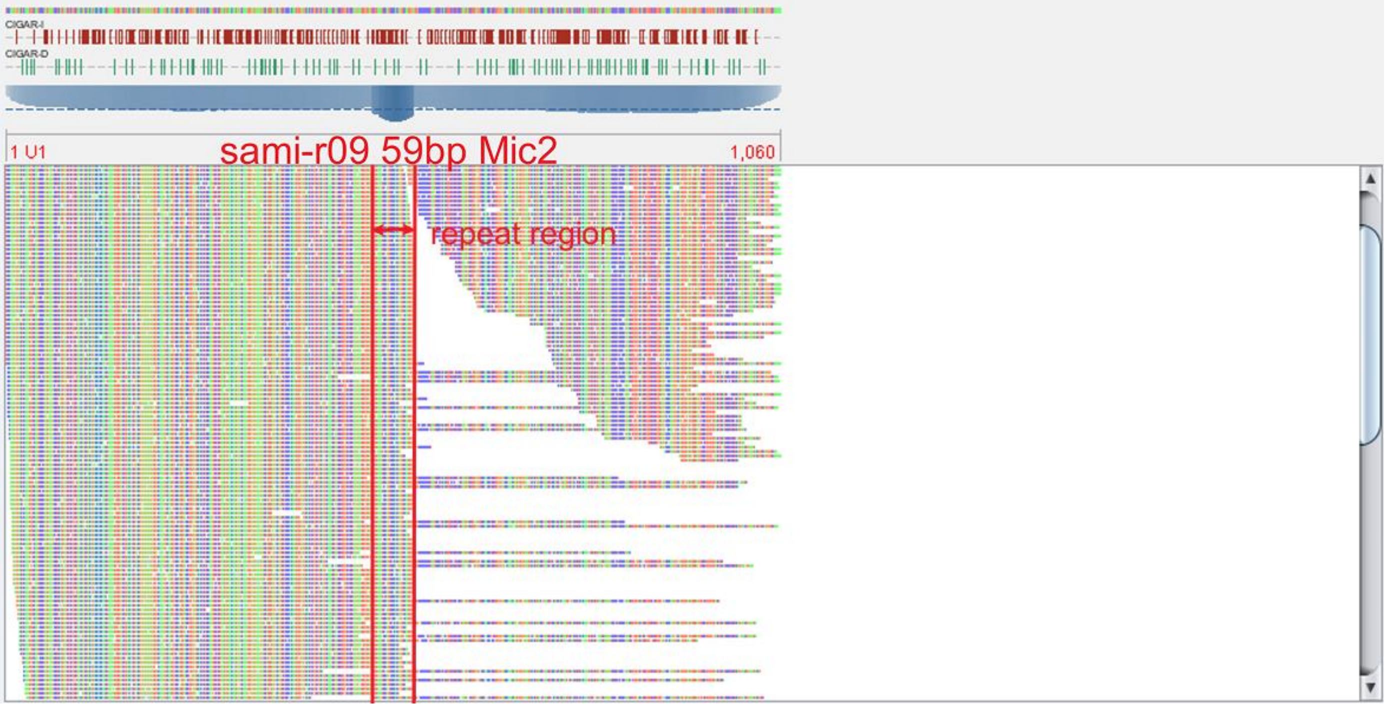
CIGAR-I
CIGAR-D
1 U1
sami-r09 59bp Mic2
1,060
repeat region

Figure S7. Validation of four conformations corresponding to the recombination products mediated by the nine repeats. PCR primers were designed based on the four conformations. The genomic DNAs were then amplified by PCR, and PCR products were subjected to Sanger sequencing. The sequencing chromatogram (top), Sanger sequencing results (labeled with "PCR" and conformation number), expected sequences (labeled with repeat ID and conformation number), and consensus sequences are shown from top to bottom. Panels a–i correspond to repeats of the r01 to r09 results, respectively. The sequences of r01–r09 are highlighted in yellow.

a

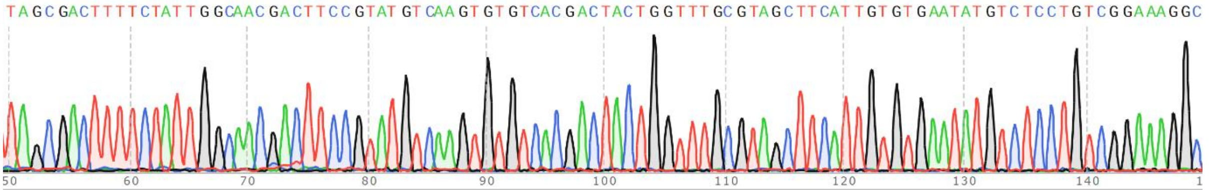

```
PCR_C1 : tagcgacttttctattggcaacgacttccgtatgtcaagtgtgtcacgactactggtttgcgtagcttcattgtgtgaatatgtctcctgtcggaaaggc : 100
r01_C1 : tagcgacttttctattggcaacgacttccgtatgtcaagtgtgtcacgactactggtttgcgtagcttcattgtgtgaatatgtctcctgtcggaaaggc : 100
         TAGCGACTTTTCTATTGGCAACGACTTCCGTATGTCAAGTGTGTCACGACTACTGGTTTGCGTAGCTTCATTGTGTGAATATGTCTCCTGTCGGAAAGGC
```

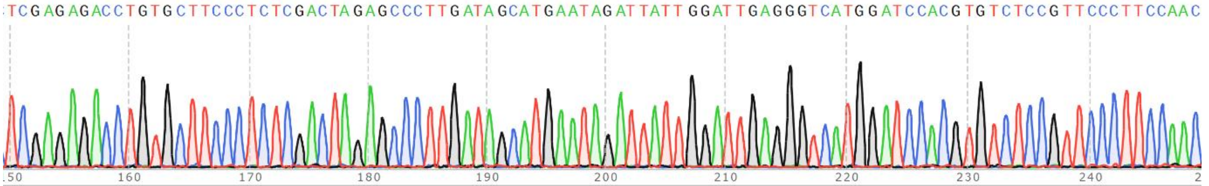

```
PCR_C1 : tcgagagacctgtgcttccctctcgactagagcccttgatagcatgaatagattattggattgagggtcatggatccacgtgtctccgttcccttccaac : 200
r01_C1 : tcgagagacctgtgcttccctctcgactagagcccttgatagcatgaatagattattggattgagggtcatggatccacgtgtctccgttcccttccaac : 200
         TCGAGAGACCTGTGCTTCCCTCTCGACTAGAGCCCTTGATAGCATGAATAGATTATTGGATTGAGGGTCATGGATCCACGTGTCTCCGTTCCCTTCCAAC
```

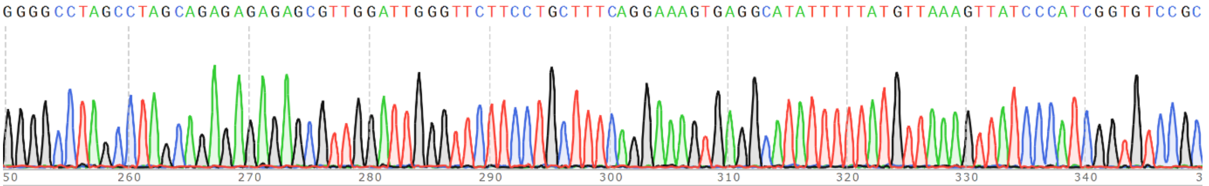

```
PCR_C1 : ggggcctagcctagcagagagagagagcgttggattgggttcttcctgctttcaggaaagtgaggcatatttttatgttaaagttatcccatcggtgtccgc : 300
r01_C1 : ggggcctagcctagcagagagagagagcgttggattgggttcttcctgctttcaggaaagtgaggcatatttttatgttaaagttatcccatcggtgtccgc : 300
         GGGGCCTAGCCTAGCAGAGAGAGAGAGCGTTGGATTGGGTTCTTCCTGCTTTCAGGAAAGTGAGGCATATTTTTATGTTAAAGTTATCCCATCGGTGTCCGC
```

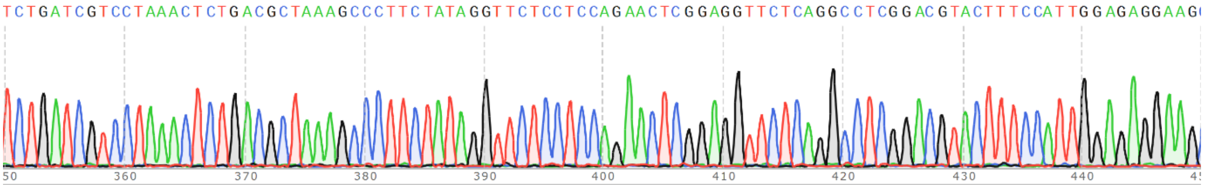

```
PCR_C1 : tctgatcgtcctaaactctgacgctaaagcccttctataggttctcctccagaactcggaggttctcaggcctcggacgtactttccattggagaggaag : 400
r01_C1 : tctgatcgtcctaaactctgacgctaaagcccttctataggttctcctccagaactcggaggttctcaggcctcggacgtactttccattggagaggaag : 400
         TCTGATCGTCCTAAACTCTGACGCTAAAGCCCTTCTATAGGTTCTCCTCCAGAACTCGGAGGTTCTCAGGCCTCGGACGTACTTTCCATTGGAGAGGAAG
```

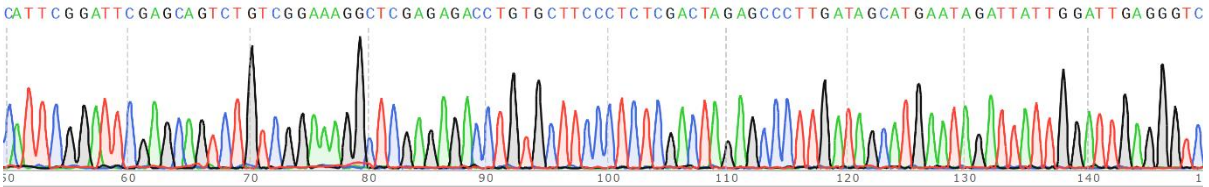

```
PCR_C2 : cattcggattcgagcagtctgtcggaaaggctcgagagacctgtgcttccctctcgactagagcccttgatagcatgaatagattattggattgagggtc : 100
r01_C2 : cattcggattcgagcagtctgtcggaaaggctcgagagacctgtgcttccctctcgactagagcccttgatagcatgaatagattattggattgagggtc : 100
         CATTCGGATTCGAGCAGTCTGTCGGAAAGGCTCGAGAGACCTGTGCTTCCCTCTCGACTAGAGCCCTTGATAGCATGAATAGATTATTGGATTGAGGGTC
```

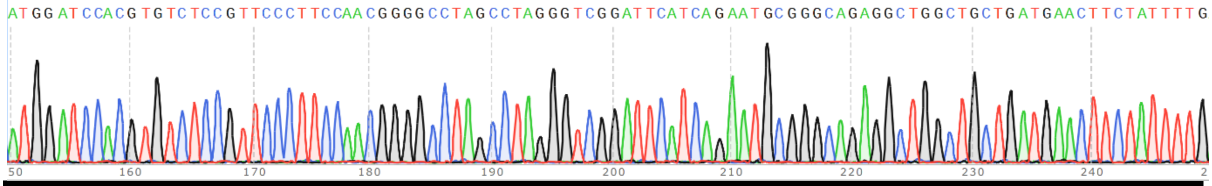

```
PCR_C2 : atggatccacgtgtctccgttcccttccaacggggcctagcctagggtcggattcatcagaatgcgggcagaggctggctgctgatgaacttctattttg : 200
r01_C2 : atggatccacgtgtctccgttcccttccaacggggcctagcctagggtcggattcatcagaatgcgggcagaggctggctgctgatgaacttctattttg : 200
         ATGGATCCACGTGTCTCCGTTCCCTTCCAACGGGGCCTAGCCTAGGGTCGGATTCATCAGAATGCGGGCAGAGGCTGGCTGCTGATGAACTTCTATTTTG
```

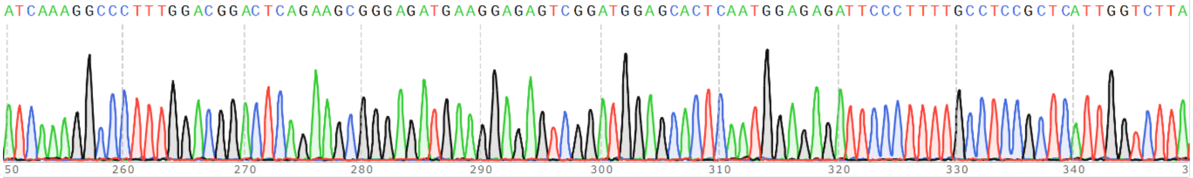

```
PCR_C2 : atcaaaggccctttggacggactcagaagcgggagatgaaggagagtcggatggagcactcaatggagagattcccttttgcctccgctcattggtctta : 300
r01_C2 : atcaaaggccctttggacggactcagaagcgggagatgaaggagagtcggatggagcactcaatggagagattcccttttgcctccgctcattggtctta : 300
         ATCAAAGGCCCTTTGGACGGACTCAGAAGCGGGAGATGAAGGAGAGTCGGATGGAGCACTCAATGGAGAGATTCCCTTTTGCCTCCGCTCATTGGTCTTA
```

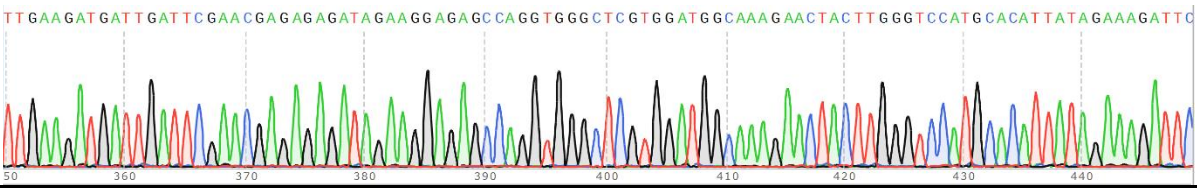

```
PCR_C2 : ttgaagatgattgattcgaacgagagagatagaaggagagccaggtgggctcgtggatggcaaagaactacttgggtccatgcacattatagaaagattc : 400
r01_C2 : ttgaagatgattgattcgaacgagagagatagaaggagagccaggtgggctcgtggatggcaaagaactacttgggtccatgcacattatagaaagattc : 400
         TTGAAGATGATTGATTCGAACGAGAGAGATAGAAGGAGAGCCAGGTGGGCTCGTGGATGGCAAAGAACTACTTGGGTCCATGCACATTATAGAAAGATTC
```

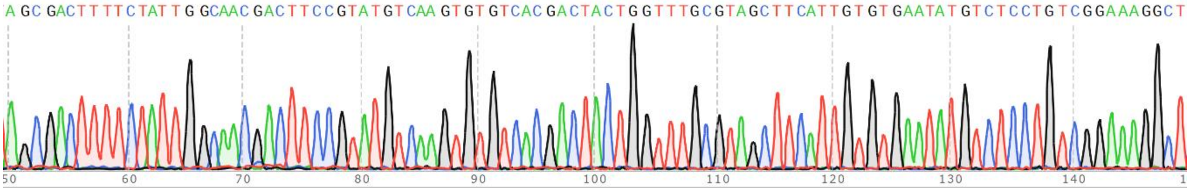

```
PCR_C3 : agcgacttttctattggcaacgacttccgtatgtcaagtgtgtcacgactactggtttgcgtagcttcattgtgtgaatatgtctcctgtcggaaaggct : 100
r01_C3 : agcgacttttctattggcaacgacttccgtatgtcaagtgtgtcacgactactggtttgcgtagcttcattgtgtgaatatgtctcctgtcggaaaggct : 100
         AGCGACTTTTCTATTGGCAACGACTTCCGTATGTCAAGTGTGTCACGACTACTGGTTTGCGTAGCTTCATTGTGTGAATATGTCTCCTGTCGGAAAGGCT
```

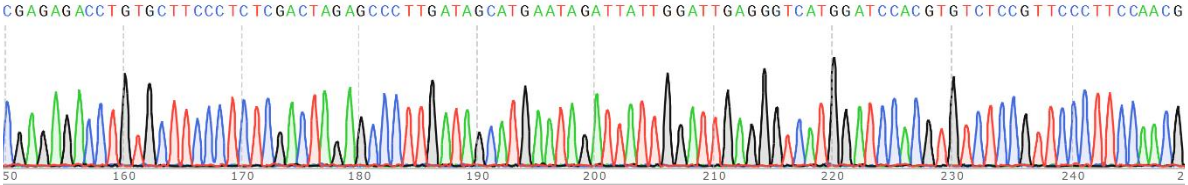

```
PCR_C3 : cgagagacctgtgcttccctctcgactagagcccttgatagcatgaatagattattggattgagggtcatggatccacgtgtctccgttcccttccaacg : 200
r01_C3 : cgagagacctgtgcttccctctcgactagagcccttgatagcatgaatagattattggattgagggtcatggatccacgtgtctccgttcccttccaacg : 200
         CGAGAGACCTGTGCTTCCCTCTCGACTAGAGCCCTTGATAGCATGAATAGATTATTGGATTGAGGGTCATGGATCCACGTGTCTCCGTTCCCTTCCAACG
```

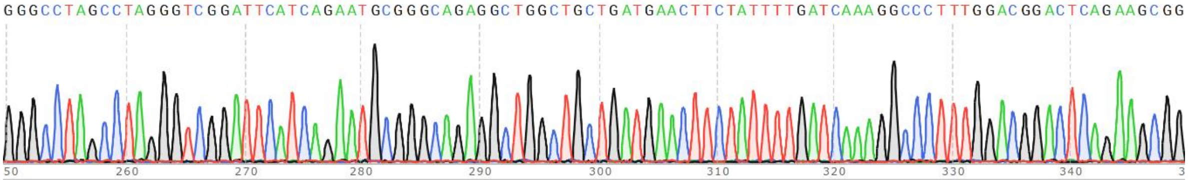

```
PCR_C3 : gggcctagcctagggtcggattcatcagaatgcgggcagaggctggctgctgatgaacttctattttgatcaaaggccctttggacggactcagaagcgg : 300
r01_C3 : gggcctagcctagggtcggattcatcagaatgcgggcagaggctggctgctgatgaacttctattttgatcaaaggccctttggacggactcagaagcgg : 300
         GGGCCTAGCCTAGGGTCGGATTCATCAGAATGCGGGCAGAGGCTGGCTGCTGATGAACTTCTATTTTGATCAAAGGCCCTTTGGACGGACTCAGAAGCGG
```

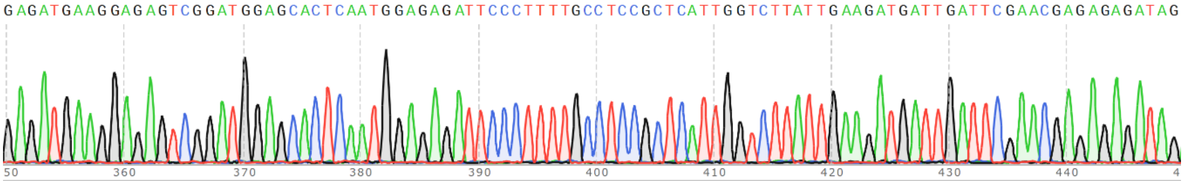

```
PCR_C3 : gagatgaaggagagtcggatggagcactcaatggagagattcccttttgcctccgctcattggtcttattgaagatgattgattcgaacgagagagatag : 400
r01_C3 : gagatgaaggagagtcggatggagcactcaatggagagattcccttttgcctccgctcattggtcttattgaagatgattgattcgaacgagagagatag : 400
         GAGATGAAGGAGAGTCGGATGGAGCACTCAATGGAGAGATTCCCTTTTGCCTCCGCTCATTGGTCTTATTGAAGATGATTGATTCGAACGAGAGAGATAG
```

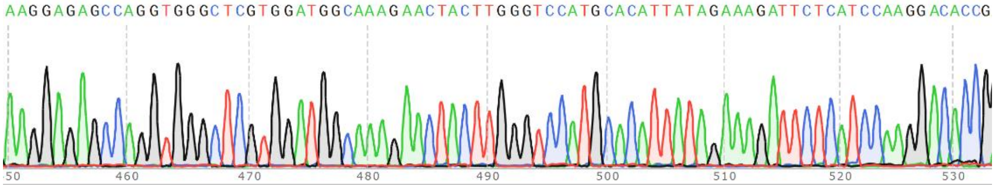

```
PCR_C3 : aaggagagccaggtgggctcgtggatggcaaagaactacttgggtccatgcacattatagaaagattctcatccaaggacaccg : 484
r01_C3 : aaggagagccaggtgggctcgtggatggcaaagaactacttgggtccatgcacattatagaaagattctcatccaaggacaccg : 484
         AAGGAGAGCCAGGTGGGCTCGTGGATGGCAAAGAACTACTTGGGTCCATGCACATTATAGAAAGATTCTCATCCAAGGACACCG
```

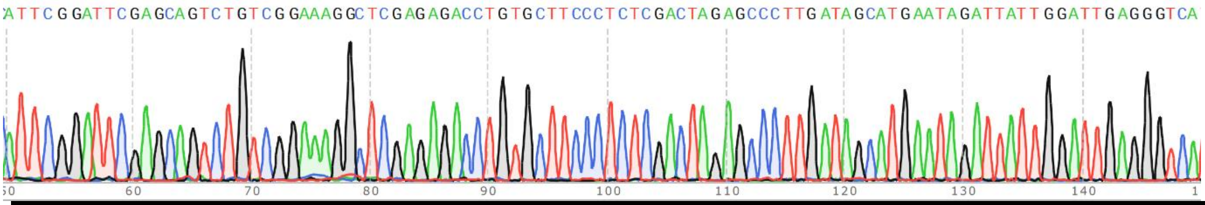

```
PCR_C4 : attcggattcgagcagtctgtcggaaaggctcgagagacctgtgcttccctctcgactagagcccttgatagcatgaatagattattggattgagggtca : 100
r01_C4 : attcggattcgagcagtctgtcggaaaggctcgagagacctgtgcttccctctcgactagagcccttgatagcatgaatagattattggattgagggtca : 100
         ATTCGGATTCGAGCAGTCTGTCGGAAAGGCTCGAGAGACCTGTGCTTCCCTCTCGACTAGAGCCCTTGATAGCATGAATAGATTATTGGATTGAGGGTCA
```

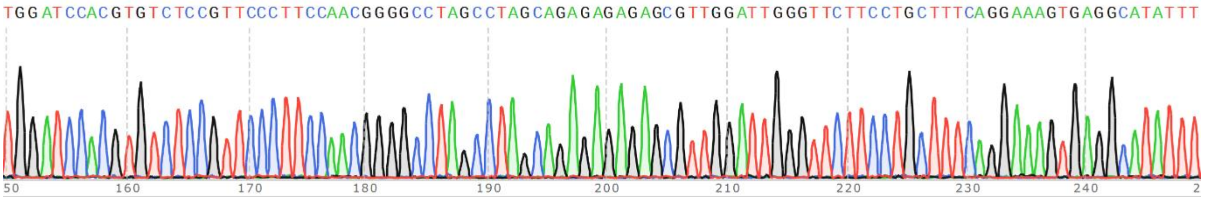

```
PCR_C4 : tggatccacgtgtctccgttcccttccaacggggcctagcctagcagagagagagcgttggattgggttcttcctgctttcaggaaagtgaggcatattt : 200
r01_C4 : tggatccacgtgtctccgttcccttccaacggggcctagcctagcagagagagagcgttggattgggttcttcctgctttcaggaaagtgaggcatattt : 200
         TGGATCCACGTGTCTCCGTTCCCTTCCAACGGGGCCTAGCCTAGCAGAGAGAGAGCGTTGGATTGGGTTCTTCCTGCTTTCAGGAAAGTGAGGCATATTT
```

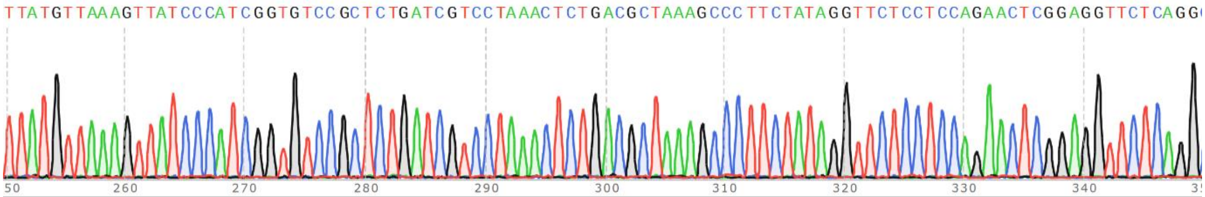

```
PCR_C4 : ttatgttaaagttatcccatcggtgtccgctctgatcgtcctaaactctgacgctaaagcccttctataggttctcctccagaactcggaggttctcagg : 300
r01_C4 : ttatgttaaagttatcccatcggtgtccgctctgatcgtcctaaactctgacgctaaagcccttctataggttctcctccagaactcggaggttctcagg : 300
         TTATGTTAAAGTTATCCCATCGGTGTCCGCTCTGATCGTCCTAAACTCTGACGCTAAAGCCCTTCTATAGGTTCTCCTCCAGAACTCGGAGGTTCTCAGG
```

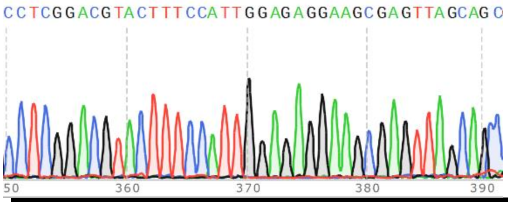

```
PCR_C4 : cctcggacgtactttccattggagaggaagcgagttagcagc : 342
r01_C4 : cctcggacgtactttccattggagaggaagcgagttagcagc : 342
         CCTCGGACGTACTTTCCATTGGAGAGGAAGCGAGTTAGCAGC
```

b

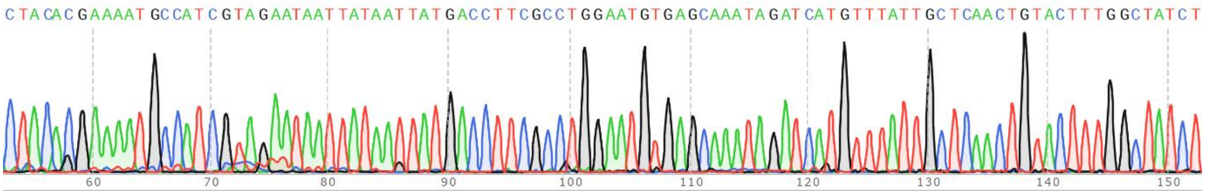

```
PCR_C1 : ctacacgaaaatgccatcgtagaataattataattatgaccttcgcctggaatgtgagcaaatagatcatgtttattgctcaactgtactttggctatct :
100
r02_C1 : ctacacgaaaatgccatcgtagaataattataattatgaccttcgcctggaatgtgagcaaatagatcatgtttattgctcaactgtactttggctatct :
100
         CTACACGAAAATGCCATCGTAGAATAATTATAATTATGACCTTCGCCTGGAATGTGAGCAAATAGATCATGTTTATTGCTCAACTGTACTTTGGCTATCT
```

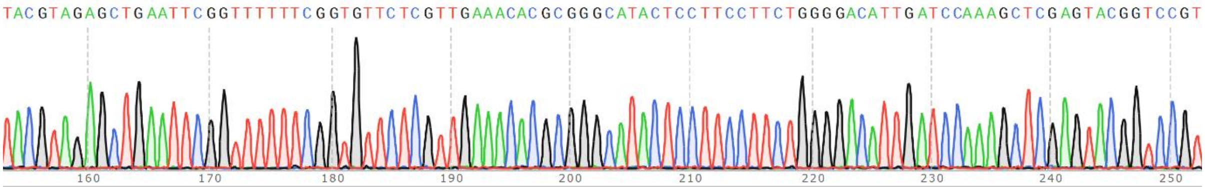

```
PCR_C1 : tacgtagagctgaattcggttttttcggtgttctcgttgaaacacgcgggcatactccttccttctggggacattgatccaaagctcgagtacggtccgt :
200
r02_C1 : tacgtagagctgaattcggttttttcggtgttctcgttgaaacacgcgggcatactccttccttctggggacattgatccaaagctcgagtacggtccgt :
200
         TACGTAGAGCTGAATTCGGTTTTTTCGGTGTTCTCGTTGAAACACGCGGGCATACTCCTTCCTTCTGGGGACATTGATCCAAAGCTCGAGTACGGTCCGT
```

GCGCCGTTTTTCTTCTCTACCATGACGAATCAATTGATTTAATGTAGGCATCGCTCTTTCCTTTGTCCTTCCCCCCTATTTTTGCCCTATCACTAGTGGT

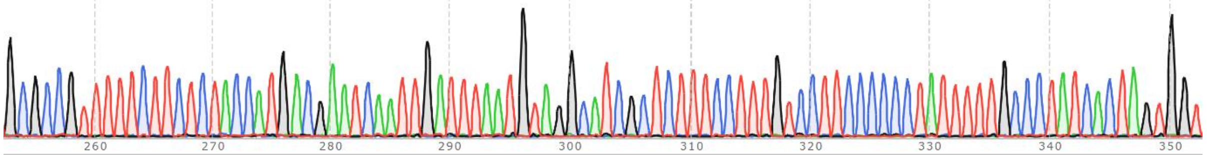

PCR_C1 : gcgccgtttttcttctctaccatgacgaatcaattgatttaatgtaggcatcgctctttcctttgtccttcccccctatttttgccctatcactagtggt : 300

r02_C1 : gcgccgtttttcttctctaccatgacgaatcaattgatttaatgtaggcatcgctctttcctttgtccttcccccctatttttgccctatcactagtggt : 300

GCGCCGTTTTTCTTCTCTACCATGACGAATCAATTGATTTAATGTAGGCATCGCTCTTTCCTTTGTCCTTCCCCCCTATTTTTGCCCTATCACTAGTGGT

TACTCCCGATCCGAAGCACCCCTTTTCCATTCATAGAGAGATCCAATCGTCAAAATCAATAAAAAGGCCATCATGGACCAAGATCCAAAGGGATCAATCT

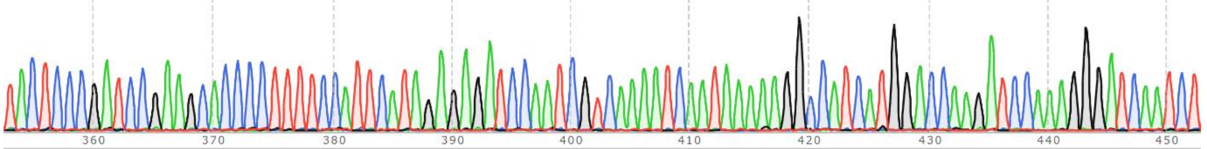

PCR_C1 : tactcccgatccgaagcaccccttttccattcatagagagatccaatcgtcaaaatcaataaaaaggccatcatggaccaagatccaaagggatcaatct : 400

r02_C1 : tactcccgatccgaagcaccccttttccattcatagagagatccaatcgtcaaaatcaataaaaaggccatcatggaccaagatccaaagggatcaatct : 400

TACTCCCGATCCGAAGCACCCCTTTTCCATTCATAGAGAGATCCAATCGTCAAAATCAATAAAAAGGCCATCATGGACCAAGATCCAAAGGGATCAATCT

TGTTGGGAGGTACTGCCCAAGGAAAGGAAAAGGTGACTTCCGGATCAGGAATAATAAATAAAATTGAAACAAGATAAAATCGTATATCAAAACGACTTCT

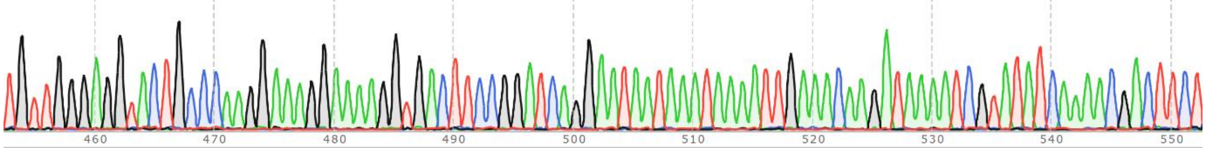

PCR_C1 : tgttgggaggtactgcccaaggaaaggaaaaggtgacttccggatcaggaataataaataaaattgaaacaagataaaatcgtatatcaaaacgacttct : 500

r02_C1 : tgttgggaggtactgcccaaggaaaggaaaaggtgacttccggatcaggaataataaataaaattgaaacaagataaaatcgtatatcaaaacgacttct : 500

TGTTGGGAGGTACTGCCCAAGGAAAGGAAAAGGTGACTTCCGGATCAGGAATAATAAATAAAATTGAAACAAGATAAAATCGTATATCAAAACGACTTCT

GGCATCACCGGAAGGATCGAAACCACATTCGTGGGCCGACAATTTTTCTGGATAGGTCGAACTATTGGAAGAAAATGGAAAAGGAAGACCGAGTGGAATC.

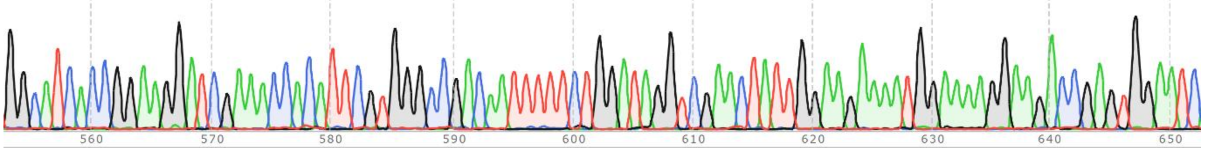

PCR_C1 : ggcatcaccggaaggatcgaaaccacattcgtgggccgacaatttttctggataggtcgaactattggaagaaaatggaaaaggaagaccgagtggaatc : 600

r02_C1 : ggcatcaccggaaggatcgaaaccacattcgtgggccgacaatttttctggataggttgaactattggaagaaaatggaaaaggaagaccgagtggaatc : 600

GGCATCACCGGAAGGATCGAAACCACATTCGTGGGCCGACAATTTTTCTGGATAGGTTGAACTATTGGAAGAAAATGGAAAAGGAAGACCGAGTGGAATC

.AAGAAACTAGCGAACTGATCACTAAATAGATCAAAATAGGTTCAAATTCGGACATCACCACAGCACACTTCGTTCTTTCGCTCCTTGCTCGCGGAGCTA

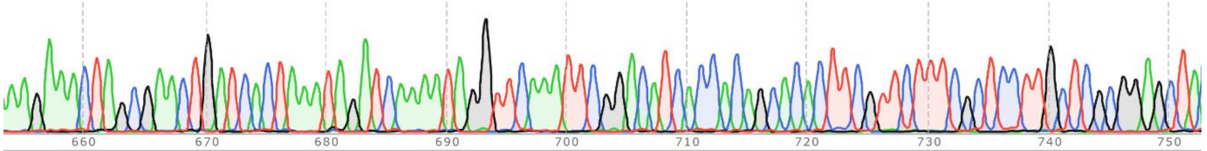

PCR_C1 : aaagaaactagcgaactgatcactaaatagatcaaaataggttcaaattcggacatcaccacagcacacttcgttctttcgctccttgctcgcggagcta : 700

r02_C1 : aaagaaactagcgaactgatcactaaatagatcaaaataggttcaaattcggacatcaccacagcacacttcgttctttcgctccttgctcgcggagcta : 700

AAAGAAACTAGCGAACTGATCACTAAATAGATCAAAATAGGTTCAAATTCGGACATCACCACAGCACACTTCGTTCTTTCGCTCCTTGCTCGCGGAGCTA

* 720 * 740 * 760 * 780 * 800

CATACCGGAAAAAAAGAAAACAACAAGGTAACTCCTAACAAGAAGCAACAAAAACTAGGGACTTCGACTTCCGATTGCTATGTGTTAAGCATCTAAGTAC

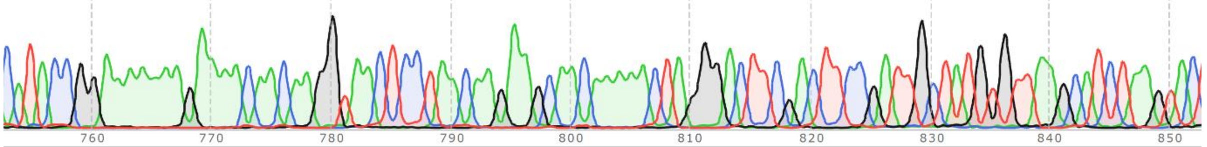

PCR_C1 : cataccggaaaaaaagaaaacaacaaggtaactcctaacaagaagcaacaaaaactagggacttcgacttccgattgctatgtgttaagcatctaagtac : 800

r02_C1 : cataccggaaaaaaagaaaacaacaaggtaactcctaacaagaagcaacaaaaactagggacttcgacttccgattgctatgtgttaagcatctaagtac : 800

CATACCGGAAAAAAAGAAAACAACAAGGTAACTCCTAACAAGAAGCAACAAAAACTAGGGACTTCGACTTCCGATTGCTATGTGTTAAGCATCTAAGTAC

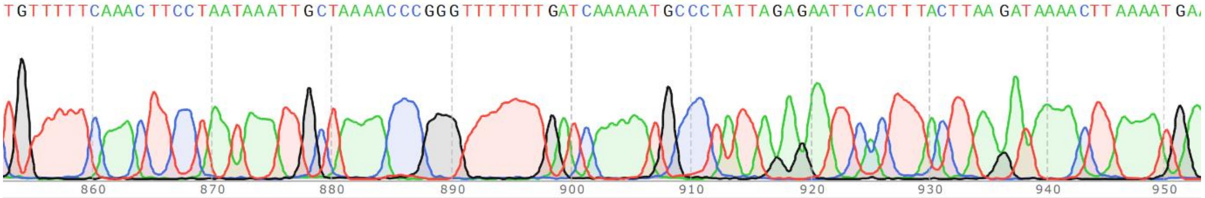


```
PCR_C1 : tgtttttcaaacttcctaataaattgctaaaacccgggtttttttgatcaaaaatgccctattagagaattcactttacttaagataaaacttaaaatga :
900
r02_C1 : tgtttttcaaacttcctaataaattgctaaaacccgggtttttttgatcaaaaatgccctattagagaattcactttacttaagataaaacttaaaatga :
900
         TGTTTTTCAAACTTCCTAATAAATTGCTAAAACCCGGGTTTTTTTGATCAAAAATGCCCTATTAGAGAATTCACTTTACTTAAGATAAAACTTAAAATGA
```

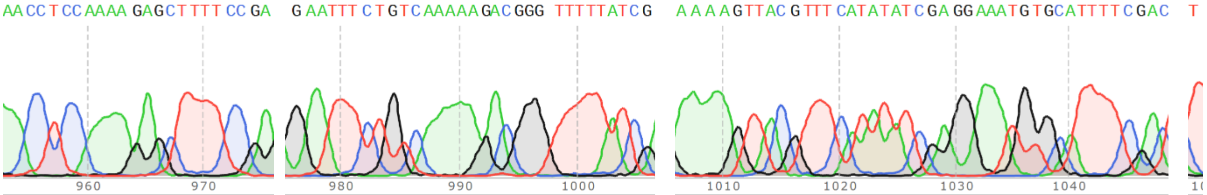


```
PCR_C1 : aacctccaaaagagcttttccga-gaatttctgtcaaaaagacgggtttttatcg-aaaagttacgtttcatatatcgaggaaatgtgcattttcgac-t :
997
r02_C1 : aacctccaaaagagcttttccgaggaatttctgtcaaaaagacgggtttttatcgaaaaagttacgtttcatatatcgaggaaatgtgcattttcgactt :
1000
         AACCTCCAAAAGAGCTTTTCCGA GAATTTCTGTCAAAAAGACGGGTTTTTATCG AAAAGTTACGTTTCATATATCGAGGAAATGTGCATTTTCGAC T
```

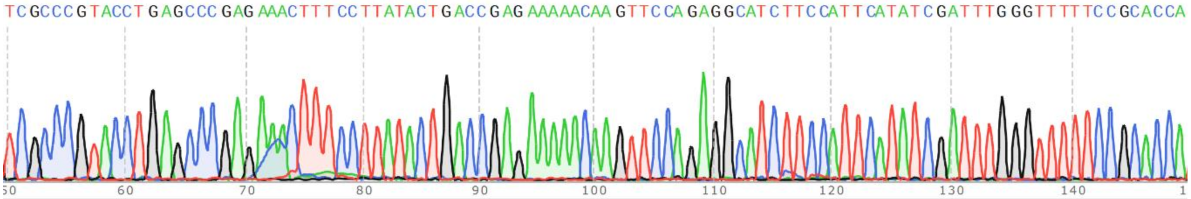


```
PCR_C2 : tcgcccgtacctgagcccgagaaactttccttatactgaccgagaaaaacaagttccagaggcatcttccattcatatcgatttgggtttttccgcacca : 100
r02_C2 : tcgcccgtacctgagcccgagaaactttccttatactgaccgagaaaaacaagttccagaggcatcttccattcatatcgatttgggtttttccgcacca : 100
          TCGCCCGTACCTGAGCCCGAGAAACTTTCCTTATACTGACCGAGAAAAACAAGTTCCAGAGGCATCTTCCATTCATATCGATTTGGGTTTTTCCGCACCA
```

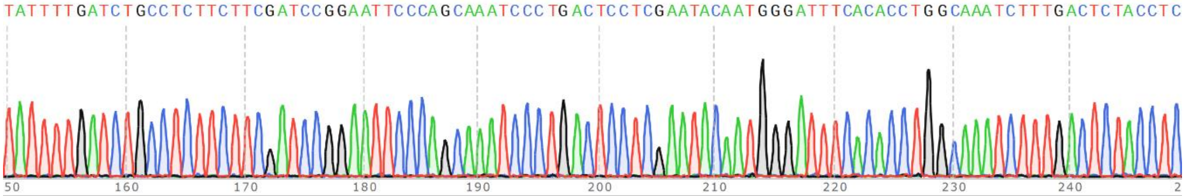


```
PCR_C2 : tattttgatctgcctcttcttcgatccggaattcccagcaaatccctgactcctcgaatacaatgggatttcacacctggcaaatctttgactctacctc : 200
r02_C2 : tattttgatctgcctcttcttcgatccggaattcccagcaaatccctgactcctcgaatacaatgggatttcacacctggcaaatctttgactctacctc : 200
          TATTTTGATCTGCCTCTTCTTCGATCCGGAATTCCCAGCAAATCCCTGACTCCTCGAATACAATGGGATTTCACACCTGGCAAATCTTTGACTCTACCTC
```

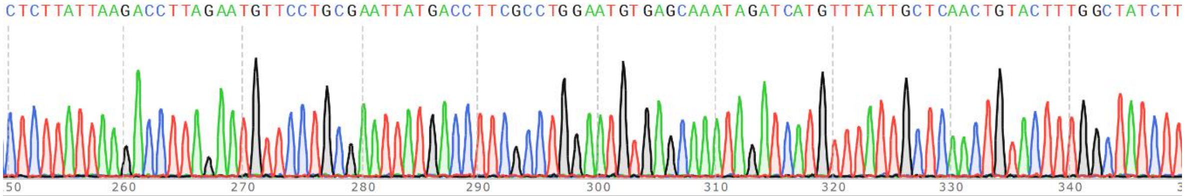


```
PCR_C2 : ctcttattaagaccttagaatgttcctgcgaattatgaccttcgcctggaatgtgagcaaatagatcatgtttattgctcaactgtactttggctatctt : 300
r02_C2 : ctcttattaagaccttagaatgttcctgcgaattatgaccttcgcctggaatgtgagcaaatagatcatgtttattgctcaactgtactttggctatctt : 300
          CTCTTATTAAGACCTTAGAATGTTCCTGCGAATTATGACCTTCGCCTGGAATGTGAGCAAATAGATCATGTTTATTGCTCAACTGTACTTTGGCTATCTT
```

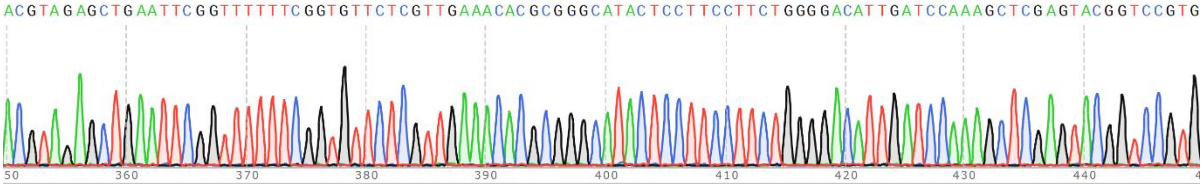


```
PCR_C2 : acgtagagctgaattcggttttttcggtgttctcgttgaaacacgcgggcatactccttccttctggggacattgatccaaagctcgagtacggtccgtg : 400
r02_C2 : acgtagagctgaattcggttttttcggtgttctcgttgaaacacgcgggcatactccttccttctggggacattgatccaaagctcgagtacggtccgtg : 400
          ACGTAGAGCTGAATTCGGTTTTTTCGGTGTTCTCGTTGAAACACGCGGGCATACTCCTTCCTTCTGGGGACATTGATCCAAAGCTCGAGTACGGTCCGTG
```

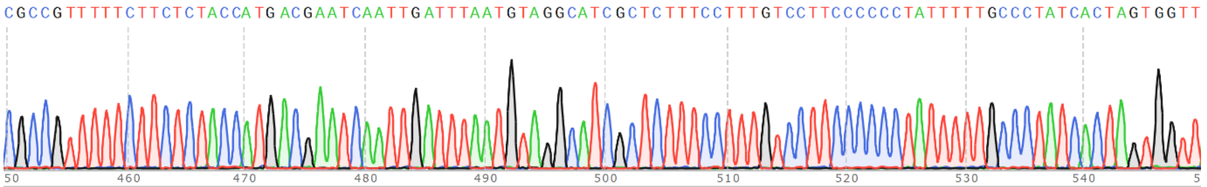

```
PCR_C2 : cgccgttttcttctctaccatgacgaatcaattgatttaatgtaggcatcgctctttcctttgtccttccccccctatttttgccctatcactagtggtt : 500
r02_C2 : cgccgttttcttctctaccatgacgaatcaattgatttaatgtaggcatcgctctttcctttgtccttccccccctatttttgccctatcactagtggtt : 500
         CGCCGTTTTCTTCTCTACCATGACGAATCAATTGATTTAATGTAGGCATCGCTCTTTCCTTTGTCCTTCCCCCCCTATTTTTGCCCTATCACTAGTGGTT
```

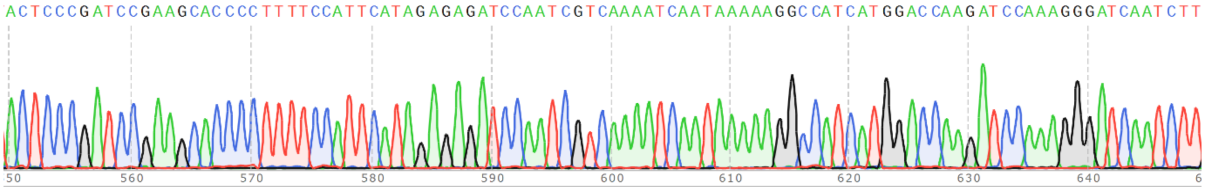

```
PCR_C2 : actcccgatccgaagcaccccttttccattcatagagagatccaatcgtcaaaatcaataaaaaggccatcatggaccaagatccaaagggatcaatctt : 600
r02_C2 : actcccgatccgaagcaccccttttccattcatagagagatccaatcgtcaaaatcaataaaaaggccatcatggaccaagatccaaagggatcaatctt : 600
         ACTCCCGATCCGAAGCACCCCTTTTCCATTCATAGAGAGATCCAATCGTCAAAATCAATAAAAAGGCCATCATGGACCAAGATCCAAAGGGATCAATCTT
```

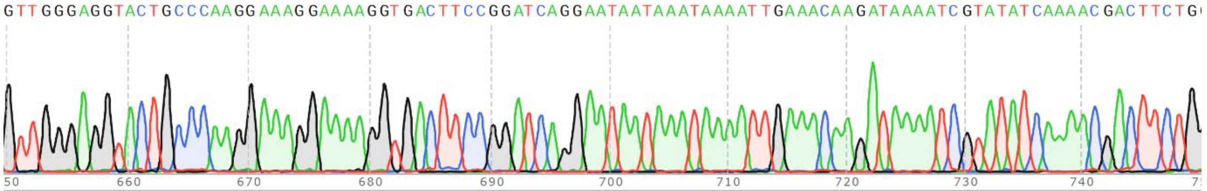

```
PCR_C2 : gttgggaggtactgcccaaggaaaggaaaaggtgacttccggatcaggaataataaataaaattgaaacaagataaaatcgtatatcaaaacgacttctg : 700
r02_C2 : gttgggaggtactgcccaaggaaaggaaaaggtgacttccggatcaggaataataaataaaattgaaacaagataaaatcgtatatcaaaacgacttctg : 700
         GTTGGGAGGTACTGCCCAAGGAAAGGAAAAGGTGACTTCCGGATCAGGAATAATAAATAAAATTGAAACAAGATAAAATCGTATATCAAAACGACTTCTG
```

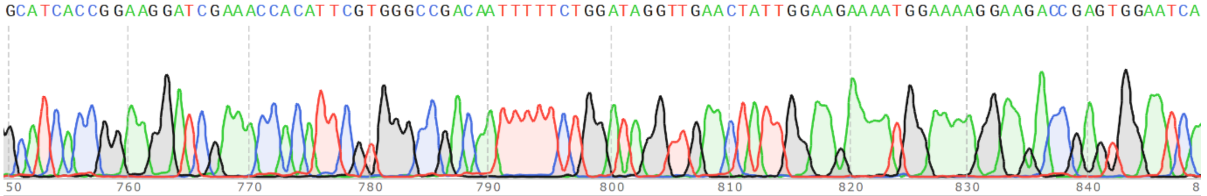

```
PCR_C2 : gcatcaccggaaggatcgaaaccacattcgtgggccgacaatttttctggataggttgaactattggaagaaaatggaaaaggaagaccgagtggaatca : 800
r02_C2 : gcatcaccggaaggatcgaaaccacattcgtgggccgacaatttttctggataggttgaactattggaagaaaatggaaaaggaagaccgagtggaatca : 800
         GCATCACCGGAAGGATCGAAACCACATTCGTGGGCCGACAATTTTTCTGGATAGGTTGAACTATTGGAAGAAAATGGAAAAGGAAGACCGAGTGGAATCA
```

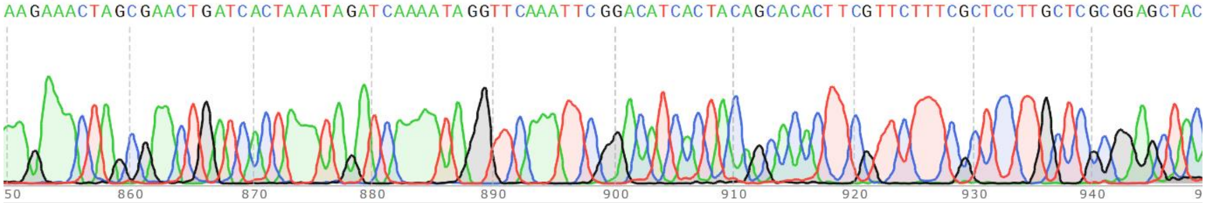

```
PCR_C2 : aagaaactagcgaactgatcactaaatagatcaaaataggttcaaattcggacatcactacagcacacttcgttctttcgctccttgctcgcggagctac : 900
r02_C2 : aagaaactagcgaactgatcactaaatagatcaaaataggttcaaattcggacatcactacagcacacttcgttctttcgctccttgctcgcggagctac : 900
         AAGAAACTAGCGAACTGATCACTAAATAGATCAAAATAGGTTCAAATTCGGACATCACTACAGCACACTTCGTTCTTTCGCTCCTTGCTCGCGGAGCTAC
```

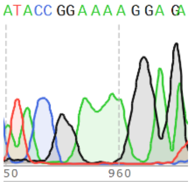

```
PCR_C2 : ataccggaaaaggaga : 916
r02_C2 : ataccggaaaaaggga : 916
         ATACCGGAAAA  G GA
```

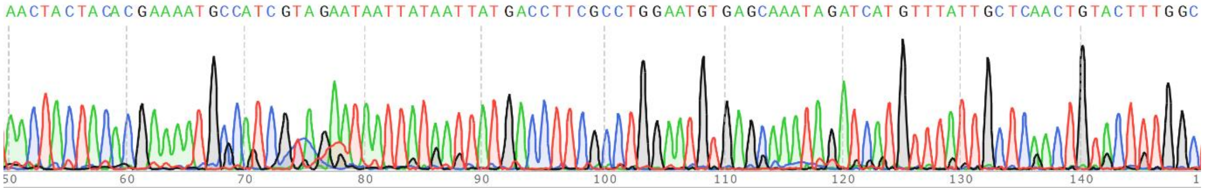

```
PCR_C3 : aactactacacgaaaatgccatcgtagaataattataattatgaccttcgcctggaatgtgagcaaatagatcatgtttattgctcaactgtactttggc : 100
r02_C3 : aactactacacgaaaatgccatcgtagaataattataattatgaccttcgcctggaatgtgagcaaatagatcatgtttattgctcaactgtactttggc : 100
         AACTACTACACGAAAATGCCATCGTAGAATAATTATAATTATGACCTTCGCCTGGAATGTGAGCAAATAGATCATGTTTATTGCTCAACTGTACTTTGGC
```

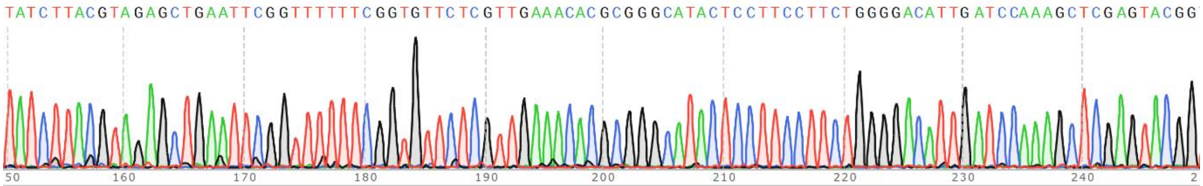

```
PCR_C3 : tatcttacgtagagctgaattcggttttttcggtgttctcgttgaaacacgcgggcatactccttccttctggggacattgatccaaagctcgagtacgg : 200
r02_C3 : tatcttacgtagagctgaattcggttttttcggtgttctcgttgaaacacgcgggcatactccttccttctggggacattgatccaaagctcgagtacgg : 200
         TATCTTACGTAGAGCTGAATTCGGTTTTTTCGGTGTTCTCGTTGAAACACGCGGGCATACTCCTTCCTTCTGGGGACATTGATCCAAAGCTCGAGTACGG
```

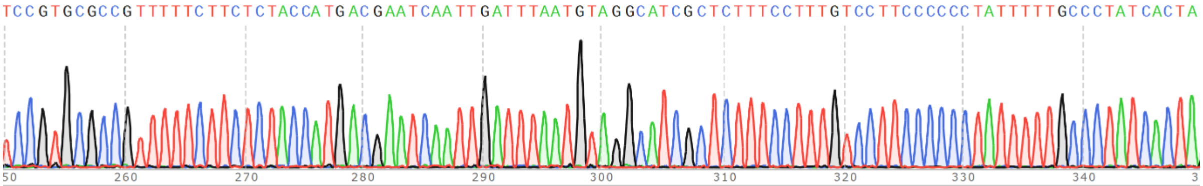

```
PCR_C3 : tccgtgcgccgtttttcttctctaccatgacgaatcaattgatttaatgtaggcatcgctctttcctttgtccttcccccctatttttgccctatcacta : 300
r02_C3 : tccgtgcgccgtttttcttctctaccatgacgaatcaattgatttaatgtaggcatcgctctttcctttgtccttcccccctatttttgccctatcacta : 300
         TCCGTGCGCCGTTTTTCTTCTCTACCATGACGAATCAATTGATTTAATGTAGGCATCGCTCTTTCCTTTGTCCTTCCCCCCTATTTTTGCCCTATCACTA
```

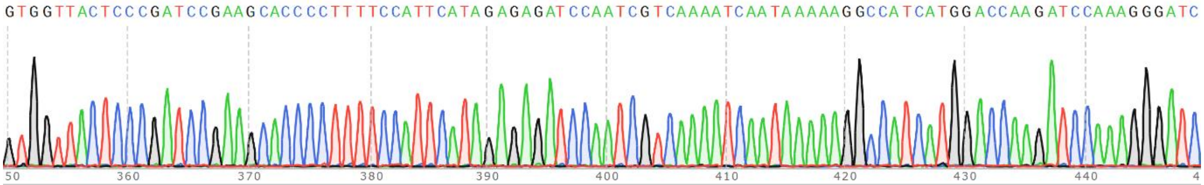

```
PCR_C3 : gtggttactcccgatccgaagcaccccttttccattcatagagagatccaatcgtcaaaatcaataaaaaggccatcatggaccaagatccaaagggatc : 400
r02_C3 : gtggttactcccgatccgaagcaccccttttccattcatagagagatccaatcgtcaaaatcaataaaaaggccatcatggaccaagatccaaagggatc : 400
         GTGGTTACTCCCGATCCGAAGCACCCCTTTTCCATTCATAGAGAGATCCAATCGTCAAAATCAATAAAAAGGCCATCATGGACCAAGATCCAAAGGGATC
```

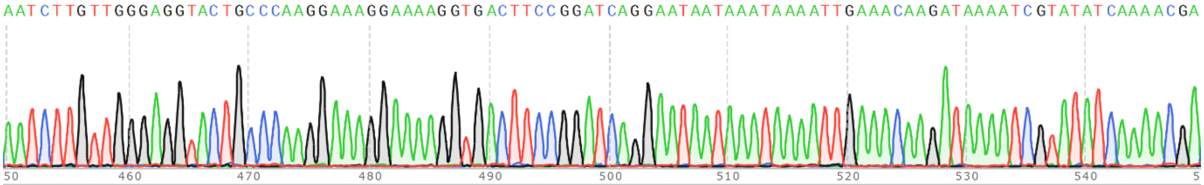

```
PCR_C3 : aatcttgttgggaggtactgcccaaggaaaggaaaaggtgacttccggatcaggaataataaataaaattgaaacaagataaaatcgtatatcaaaacga : 500
r02_C3 : aatcttgttgggaggtactgcccaaggaaaggaaaaggtgacttccggatcaggaataataaataaaattgaaacaagataaaatcgtatatcaaaacga : 500
         AATCTTGTTGGGAGGTACTGCCCAAGGAAAGGAAAAGGTGACTTCCGGATCAGGAATAATAAATAAAATTGAAACAAGATAAAATCGTATATCAAAACGA
```

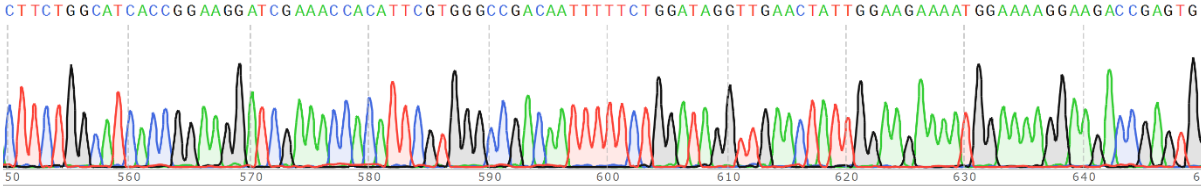

```
PCR_C3 : cttctggcatcaccggaaggatcgaaaccacattcgtgggccgacaatttttctggataggttgaactattggaagaaaatggaaaaggaagaccgagtg : 600
r02_C3 : cttctggcatcaccggaaggatcgaaaccacattcgtgggccgacaatttttctggataggttgaactattggaagaaaatggaaaaggaagaccgagtg : 600
         CTTCTGGCATCACCGGAAGGATCGAAACCACATTCGTGGGCCGACAATTTTTCTGGATAGGTTGAACTATTGGAAGAAAATGGAAAAGGAAGACCGAGTG
```

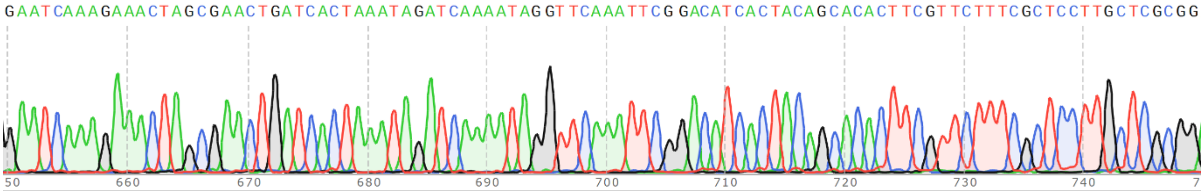

```
PCR_C3 : gaatcaaagaaactagcgaactgatcactaaatagatcaaaataggttcaaattcggacatcactacagcacacttcgttctttcgctccttgctcgcgg : 700
r02_C3 : gaatcaaagaaactagcgaactgatcactaaatagatcaaaataggttcaaattcggacatcaccacagcacacttcgttctttcgctccttgctcgcgg : 700
         GAATCAAAGAAACTAGCGAACTGATCACTAAATAGATCAAAATAGGTTCAAATTCGGACATCAC ACAGCACACTTCGTTCTTTCGCTCCTTGCTCGCGG
```

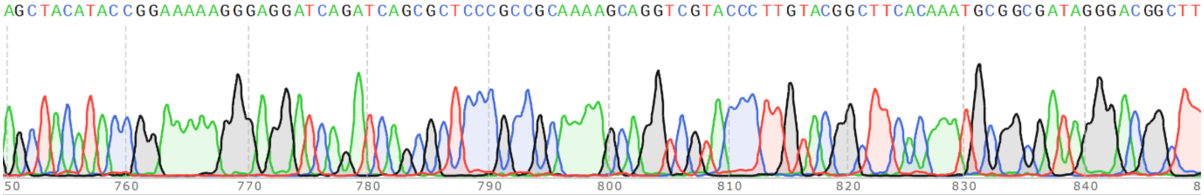

```
PCR_C3 : agctacataccggaaaaagggaggatcagatcagcgctcccgccgcaaaagcaggtcgtacccttgtacggcttcacaaatgcggcgatagggacggctt : 800
r02_C3 : agctacataccggaaaaagggaggatcagatcagcgctcccgccgcaaaagcaggtcgtacccttgtacggcttcacaaatgcggcgatagggacggctt : 800
         AGCTACATACCGGAAAAAGGGAGGATCAGATCAGCGCTCCCGCCGCAAAAGCAGGTCGTACCCTTGTACGGCTTCACAAATGCGGCGATAGGGACGGCTT
```

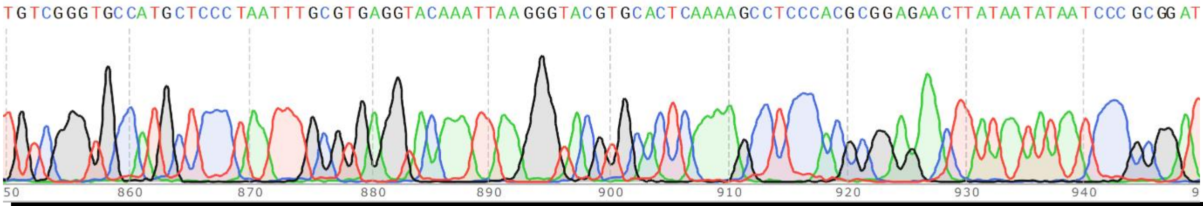

PCR_C3 : tgtcgggtgccatgctccctaatttgcgtgaggtacaaattaagggtacgtgcactcaaaagcctcccacgcggagaacttataatataatcccgcggat : 900
r02_C3 : tgtcgggtgccatgctccctaatttgcgtgaggtacaaattaagggtacgtgcactcaaaagcctcccacgcggagaacttataatataatcccgcggat : 900
TGTCGGGTGCCATGCTCCCTAATTTGCGTGAGGTACAAATTAAGGGTACGTGCACTCAAAAGCCTCCCACGCGGAGAACTTATAATATAATCCCGCGGAT

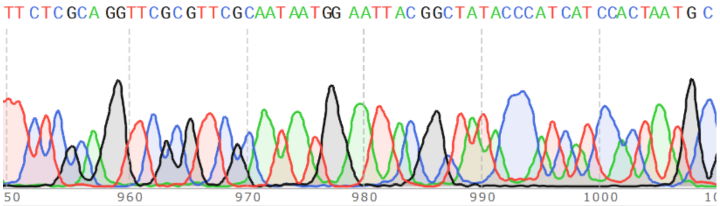

PCR_C3 : ttctcgcaggttcgcgttcgcaataatggaattacggctatacccatcatccactaatgc : 960
r02_C3 : ttctcgcaggttcgcgttcgcaataatggaattacggctatacccatcatccactaatgc : 960
TTCTCGCAGGTTCGCGTTCGCAATAATGGAATTACGGCTATACCCATCATCCACTAATGC

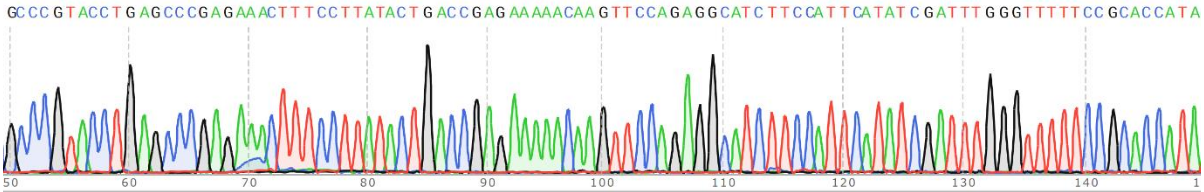

PCR_C4 : gcccgtacctgagcccgagaaactttccttatactgaccgagaaaaacaagttccagaggcatcttccattcatatcgatttgggtttttccgcaccata : 100
r02_C4 : gcccgtacctgagcccgagaaactttccttatactgaccgagaaaaacaagttccagaggcatcttccattcatatcgatttgggtttttccgcaccata : 100

GCCCGTACCTGAGCCCGAGAAACTTTCCTTATACTGACCGAGAAAAACAAGTTCCAGAGGCATCTTCCATTCATATCGATTTGGGTTTTTCCGCACCATA

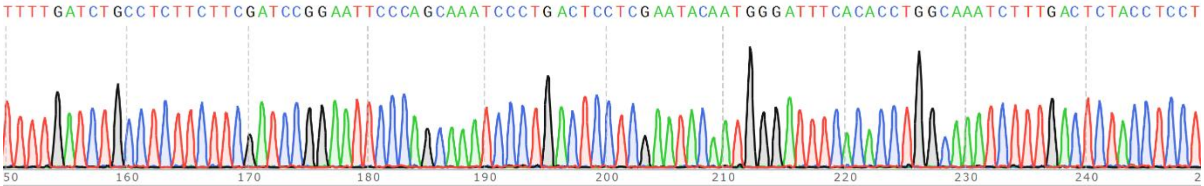

PCR_C4 : tttgatctgcctcttcttcgatccggaattcccagcaaatccctgactcctcgaatacaatgggatttcacacctggcaaatctttgactctacctcct : 200
r02_C4 : tttgatctgcctcttcttcgatccggaattcccagcaaatccctgactcctcgaatacaatgggatttcacacctggcaaatctttgactctacctcct : 200

TTTTGATCTGCCTCTTCTTCGATCCGGAATTCCCAGCAAATCCCTGACTCCTCGAATACAATGGGATTTCACACCTGGCAAATCTTTGACTCTACCTCCT

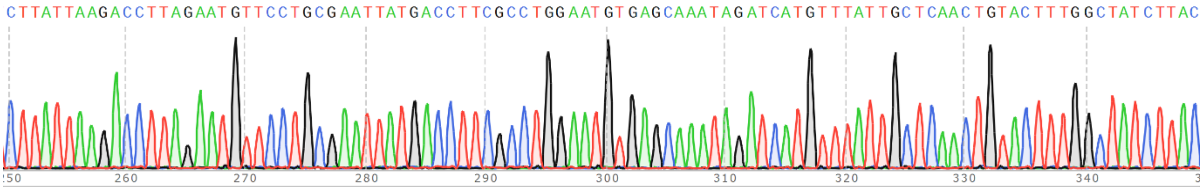

PCR_C4 : cttattaagaccttagaatgttcctgcgaattatgaccttcgcctggaatgtgagcaaatagatcatgtttattgctcaactgtactttggctatcttac : 300
r02_C4 : cttattaagaccttagaatgttcctgcgaattatgaccttcgcctggaatgtgagcaaatagatcatgtttattgctcaactgtactttggctatcttac : 300

CTTATTAAGACCTTAGAATGTTCCTGCGAATTATGACCTTCGCCTGGAATGTGAGCAAATAGATCATGTTTATTGCTCAACTGTACTTTGGCTATCTTAC

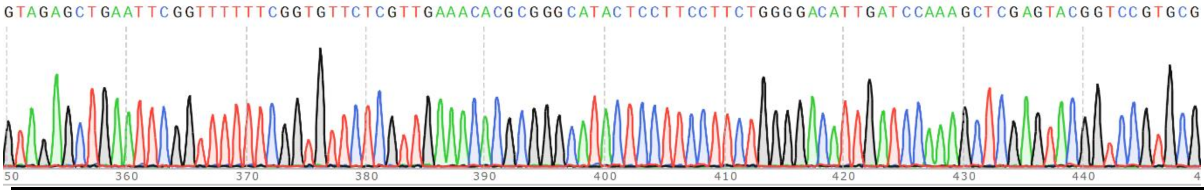

PCR_C4 : gtagagctgaattcggtttttttcggtgttctcgttgaaacacgcgggcatactccttccttctggggacattgatccaaagctcgagtacggtccgtgcg : 400
r02_C4 : gtagagctgaattcggtttttttcggtgttctcgttgaaacacgcgggcatactccttccttctggggacattgatccaaagctcgagtacggtccgtgcg : 400

GTAGAGCTGAATTCGGTTTTTTTCGGTGTTCTCGTTGAAACACGCGGGCATACTCCTTCCTTCTGGGGACATTGATCCAAAGCTCGAGTACGGTCCGTGCG

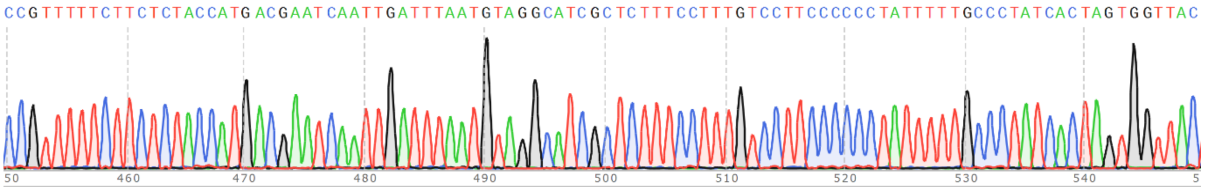

PCR_C4 : ccgtttttcttctctaccatgacgaatcaattgatttaatgtaggcatcgctctttcctttgtccttccccccctatttttgccctatcactagtggttac :
500
r02_C4 : ccgtttttcttctctaccatgacgaatcaattgatttaatgtaggcatcgctctttcctttgtccttccccccctatttttgccctatcactagtggttac :
500

CCGTTTTTCTTCTCTACCATGACGAATCAATTGATTTAATGTAGGCATCGCTCTTTCCTTTGTCCTTCCCCCCTATTTTTGCCCTATCACTAGTGGTTAC

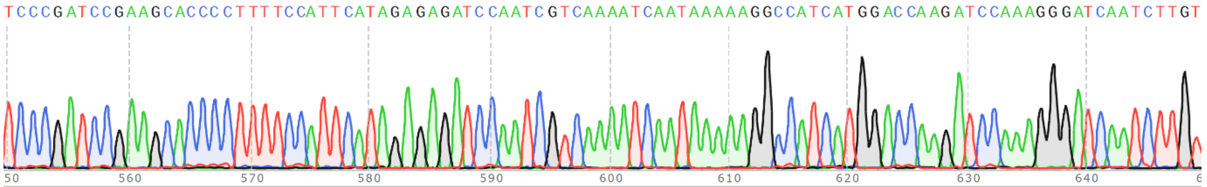

PCR_C4 : tcccgatccgaagcacccctttttccattcatagagagatccaatcgtcaaaatcaataaaaaggccatcatggaccaagatccaaagggatcaatcttgt :
600
r02_C4 : tcccgatccgaagcacccctttttccattcatagagagatccaatcgtcaaaatcaataaaaaggccatcatggaccaagatccaaagggatcaatcttgt :
600

TCCCGATCCGAAGCACCCCTTTTCCATTCATAGAGAGATCCAATCGTCAAAATCAATAAAAAGGCCATCATGGACCAAGATCCAAAGGGATCAATCTTGT

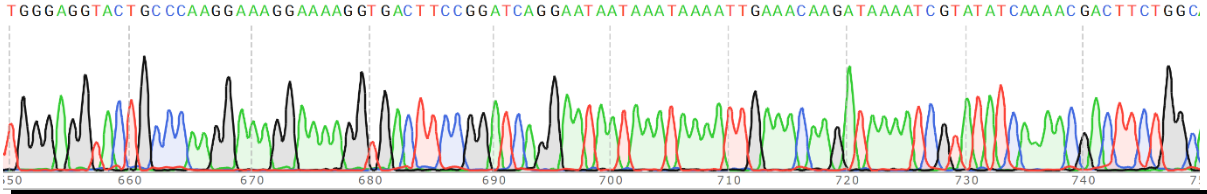

PCR_C4 : tgggaggtactgcccaaggaaaggaaaaggtgacttccggatcaggaataataaataaaattgaaacaagataaaatcgtatatcaaaacgacttctggc :
700
r02_C4 : tgggaggtactgcccaaggaaaggaaaaggtgacttccggatcaggaataataaataaaattgaaacaagataaaatcgtatatcaaaacgacttctggc :
700

TGGGAGGTACTGCCCAAGGAAAGGAAAAGGTGACTTCCGGATCAGGAATAATAAATAAAATTGAAACAAGATAAAATCGTATATCAAAACGACTTCTGGC

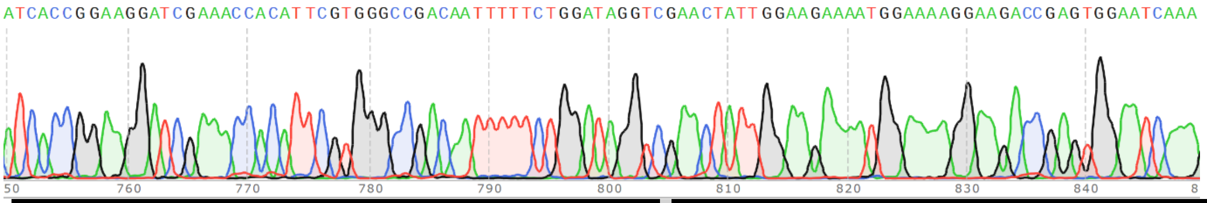

PCR_C4 : atcaccggaaggatcgaaaccacattcgtgggccgacaatttttctggataggtcgaactattggaagaaaatggaaaaggaagaccgagtggaatcaaa :
800
r02_C4 : atcaccggaaggatcgaaaccacattcgtgggccgacaatttttctggataggttgaactattggaagaaaatggaaaaggaagaccgagtggaatcaaa :
800

ATCACCGGAAGGATCGAAACCACATTCGTGGGCCGACAATTTTTCTGGATAGGT GAACTATTGGAAGAAAATGGAAAAGGAAGACCGAGTGGAATCAAA

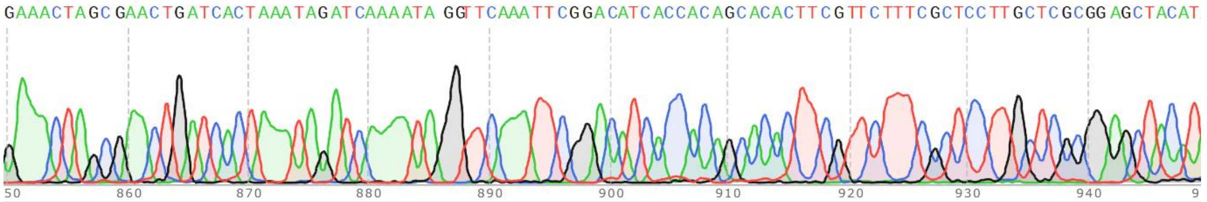

PCR_C4 : gaaactagcgaactgatcactaaatagatcaaaataggttcaaattcggacatcaccacagcacacttcgttctttcgctccttgctcgcggagctacat :
900
r02_C4 : gaaactagcgaactgatcactaaatagatcaaaataggttcaaattcggacatcaccacagcacacttcgttctttcgctccttgctcgcggagctacat :
900

GAAACTAGCGAACTGATCACTAAATAGATCAAAATAGGTTCAAATTCGGACATCACCACAGCACACTTCGTTCTTTCGCTCCTTGCTCGCGGAGCTACAT

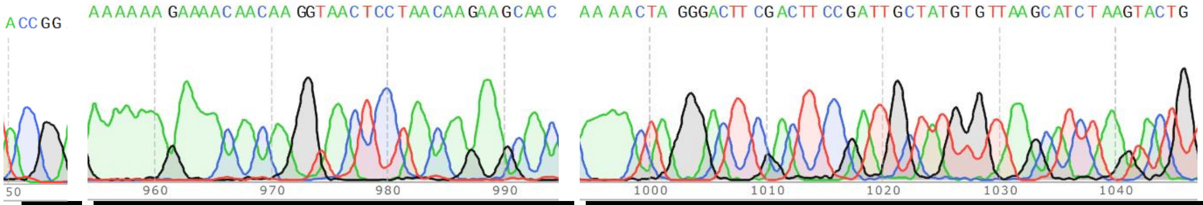

PCR_C4 : accgg-aaaaaagaaaacaacaaggtaactcctaacaagaagcaac-aaaactagggacttcgacttccgattgctatgtgttaagcatctaagtactg : 997
r02_C4 : accggaaaaaaagaaaacaacaaggtaactcctaacaagaagcaacaaaaactagggacttcgacttccgattgctatgtgttaagcatctaagtactg : 999
ACCGG AAAAAAGAAAACAACAAGGTAACTCCTAACAAGAAGCAAC AAAACTAGGGACTTCGACTTCCGATTGCTATGTGTTAAGCATCTAAGTACTG

c

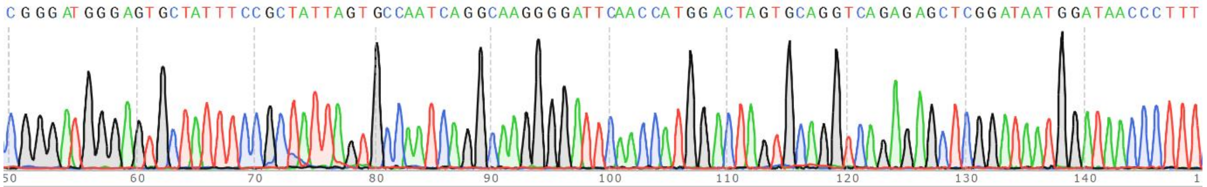

```
PCR_C1 : cgggatgggagtgctatttccgctattagtgccaatcaggcaaggggattcaaccatggactagtgcaggtcagagagctcggataatggataaccccttt : 100
r03_C1 : cgggatgggagtgctatttccgctattagtgccaatcaggcaaggggattcaaccatggactagtgcaggtcagagagctcggataatggataaccccttt : 100
         CGGGATGGGAGTGCTATTTCCGCTATTAGTGCCAATCAGGCAAGGGGATTCAACCATGGACTAGTGCAGGTCAGAGAGCTCGGATAATGGATAACCCCTTT
```

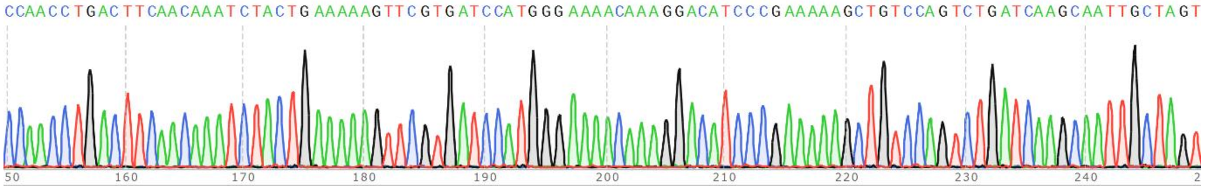

```
PCR_C1 : ccaacctgacttcaacaaatctactgaaaaagttcgtgatccatgggaaaacaaaggacatcccgaaaaagctgtccagtctgatcaagcaattgctagt : 200
r03_C1 : ccaacctgacttcaacaaatctactgaaaaagttcgtgatccatgggaaaacaaaggacatcccgaaaaagctgtccagtctgatcaagcaattgctagt : 200
         CCAACCTGACTTCAACAAATCTACTGAAAAAGTTCGTGATCCATGGGAAAACAAAGGACATCCCGAAAAAGCTGTCCAGTCTGATCAAGCAATTGCTAGT
```

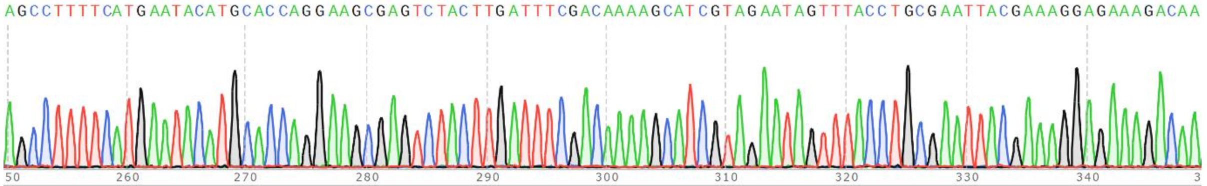

```
PCR_C1 : agccttttcatgaatacatgcaccaggaagcgagtctacttgatttcgacaaaagcatcgtagaatagtttacctgcgaattacgaaaggagaaagacaa : 300
r03_C1 : agccttttcatgaatacatgcaccaggaagcgagtctacttgatttcgacaaaagcatcgtagaatagtttacctgcgaattacgaaaggagaaagacaa : 300
         AGCCTTTTCATGAATACATGCACCAGGAAGCGAGTCTACTTGATTTCGACAAAAGCATCGTAGAATAGTTTACCTGCGAATTACGAAAGGAGAAAGACAA
```

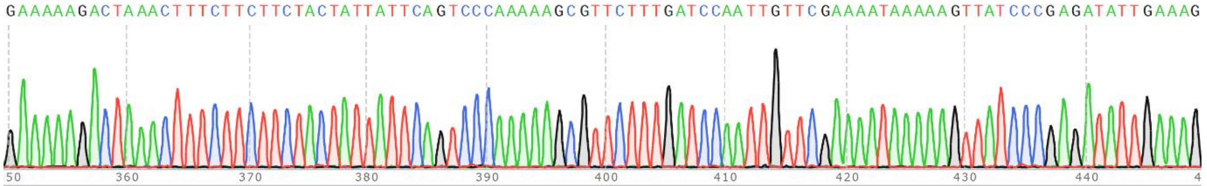

```
PCR_C1 : gaaaaagactaaactttcttcttctactattattcagtcccaaaaagcgttctttgatccaattgttcgaaaataaaaagttatcccgagatattgaaag : 400
r03_C1 : gaaaaagactaaactttcttcttctactattattcagtcccaaaaagcgttctttgatccaattgttcgaaaataaaaagttatcccgagatattgaaag : 400
         GAAAAAGACTAAACTTTCTTCTTCTACTATTATTCAGTCCCAAAAAGCGTTCTTTGATCCAATTGTTCGAAAATAAAAAGTTATCCCGAGATATTGAAAG
```

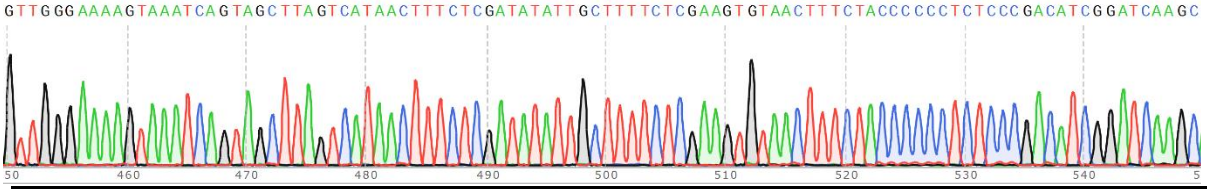

```
PCR_C1 : gttgggaaaagtaaatcagtagcttagtcataactttctcgatatattgcttttctcgaagtgtaactttctacccccctctcccgacatcggatcaagc : 500
r03_C1 : gttgggaaaagtaaatcagtagcttagtcataactttctcgatatattgcttttctcgaagtgtaactttctacccccctctcccgacatcggatcaagc : 500
         GTTGGGAAAAGTAAATCAGTAGCTTAGTCATAACTTTCTCGATATATTGCTTTTCTCGAAGTGTAACTTTCTACCCCCCTCTCCCGACATCGGATCAAGC
```

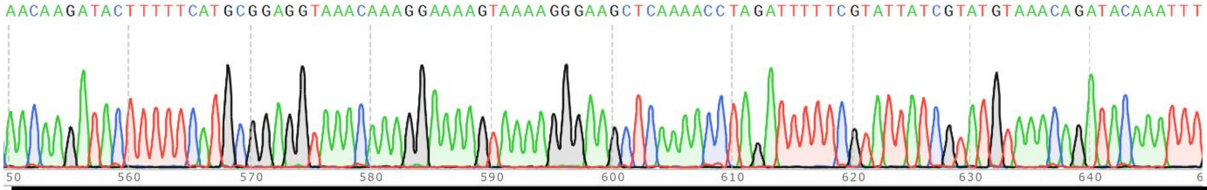

```
PCR_C1 : aacaagatactttttcatgcggaggtaaacaaaggaaaagtaaaagggaagctcaaaacctagatttttcgtattatcgtatgtaaacagatacaaattt : 600
r03_C1 : aacaagatactttttcatgcggaggtaaacaaaggaaaagtaaaagggaagctcaaaacctagatttttcgtattatcgtatgtaaacagatacaaattt : 600
         AACAAGATACTTTTTCATGCGGAGGTAAACAAAGGAAAAGTAAAAGGGAAGCTCAAAACCTAGATTTTTCGTATTATCGTATGTAAACAGATACAAATTT
```

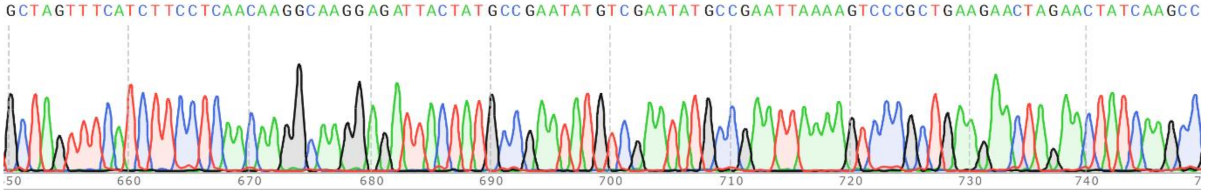

```
PCR_C1 : gctagtttcatcttcctcaacaaggcaaggagattactatgccgaatatgtcgaatatgccgaattaaaagtcccgctgaagaactagaactatcaagcc : 700
```

r03_C1 : gctagtttcatcttcctcaacaaggcaaggagattactatgccgaatatgtcgaatatgccgaattaaaagtcccgctgaagaactagaactatcaagcc : 700
GCTAGTTTCATCTTCCTCAACAAGGCAAGGAGATTACTATGCCGAATATGTCGAATATGCCGAATTAAAAGTCCCGCTGAAGAACTAGAACTATCAAGCC

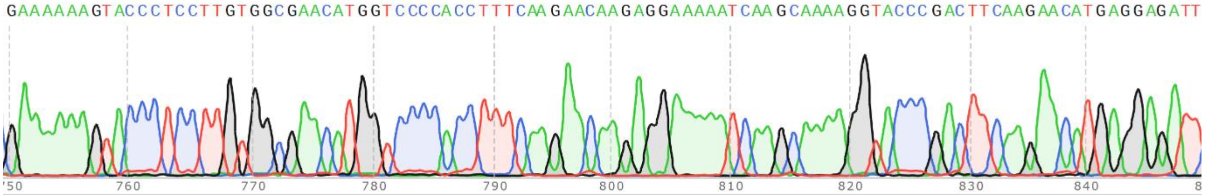

PCR_C1 : gaaaaaagtaccctccttgtggcgaacatggtccccacctttcaagaacaagaggaaaaatcaagcaaaaggtacccgacttcaagaacatgaggagatt : 800
r03_C1 : gaaaaaagtaccctccttgtggcgaacatggtccccacctttcaagaacaagaggaaaaatcaagcaaaaggtacccgacttcaagaacatgaggagatt : 800
GAAAAAAGTACCCTCCTTGTGGCGAACATGGTCCCCACCTTTCAAGAACAAGAGGAAAAATCAAGCAAAAGGTACCCGACTTCAAGAACATGAGGAGATT

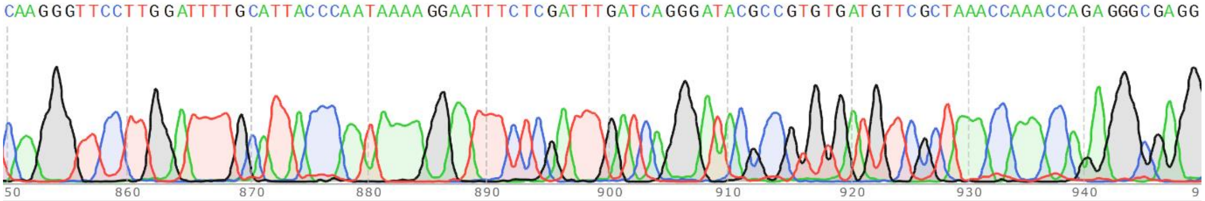

PCR_C1 : caagggttccttggattttgcattacccaataaaaggaatttctcgatttgatcagggatacgccgtgtgatgttcgctaaaccaaaccagagggcgagg : 900
r03_C1 : caagggttccttggattttgcattacccaataaaaggaatttctcgatttgatcagggatacgccgtgtgatgttcgctaaaccaaaccagagggcgagg : 900
CAAGGGTTCCTTGGATTTTGCATTACCCAATAAAAGGAATTTCTCGATTTGATCAGGGATACGCCGTGTGATGTTCGCTAAACCAAACCAGAGGGCGAGG

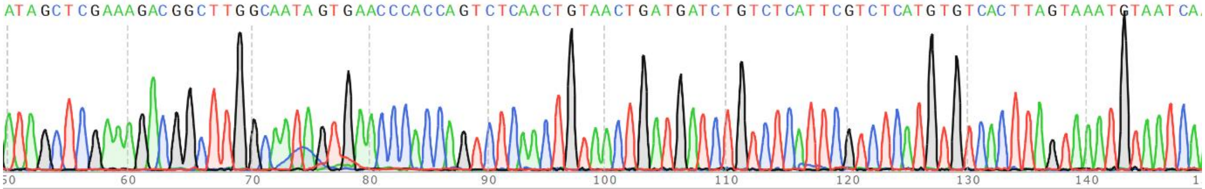

PCR_C1 : atagctcgaaagacggcttggcaatagtgaacccaccagtctcaactgtaactgatgatctgtctcattcgtctcatgtgtcacttagtaaatgtaatca : 100
r03_C1 : atagctcgaaagacggcttggcaatagtgaacccaccagtctcaactgtaactgatgatctgtctcattcgtctcatgtgtcacttagtaaatgtaatca : 100
ATAGCTCGAAAGACGGCTTGGCAATAGTGAACCCACCAGTCTCAACTGTAACTGATGATCTGTCTCATTCGTCTCATGTGTCACTTAGTAAATGTAATCA

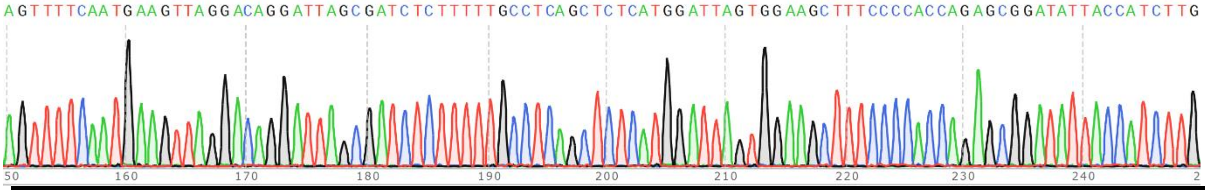

PCR_C1 : agttttcaatgaagttaggacaggattagcgatctctttttgcctcagctctcatggattagtggaagctttccccaccagagcggatattaccatcttg : 200
r03_C1 : agttttcaatgaagttaggacaggattagcgatctctttttgcctcagctctcatggattagtggaagctttccccaccagagcggatattaccatcttg : 200
AGTTTTCAATGAAGTTAGGACAGGATTAGCGATCTCTTTTTGCCTCAGCTCTCATGGATTAGTGGAAGCTTTCCCCACCAGAGCGGATATTACCATCTTG

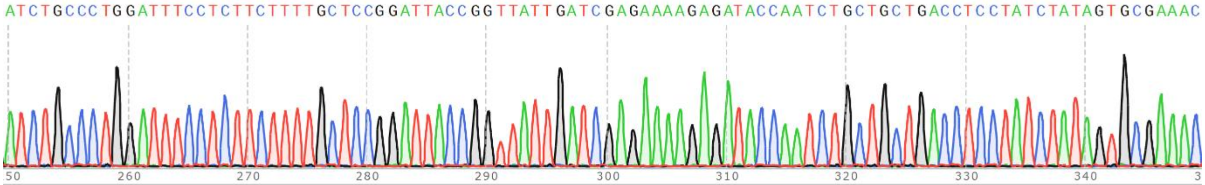

PCR_C1 : atctgccctggatttcctcttcttttgctccggattaccggttattgatcgagaaaagagataccaatctgctgctgacctcctatctatagtgcgaaac : 300
r03_C1 : atctgccctggatttcctcttcttttgctccggattaccggttattgatcgagaaaagagataccaatctgctgctgacctcctatctatagtgcgaaac : 300
ATCTGCCCTGGATTTCCTCTTCTTTTGCTCCGGATTACCGGTTATTGATCGAGAAAAGAGATACCAATCTGCTGCTGACCTCCTATCTATAGTGCGAAAC

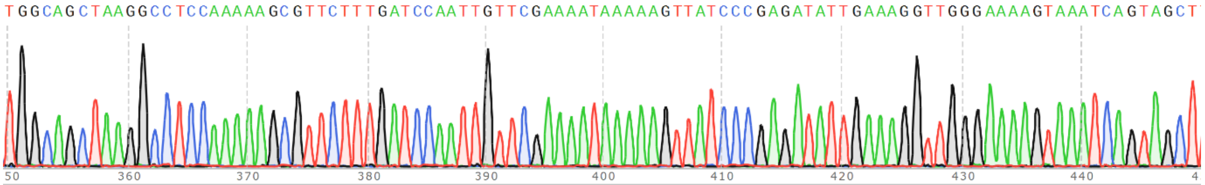

PCR_C1 : tggcagctaaggcctccaaaaagcgttctttgatccaattgttcgaaaataaaaagttatcccgagatattgaaaggttgggaaaagtaaatcagtagct : 400
r03_C1 : tggcagctaaggcctccaaaaagcgttctttgatccaattgttcgaaaataaaaagttatcccgagatattgaaaggttgggaaaagtaaatcagtagct : 400
TGGCAGCTAAGGCCTCCAAAAAGCGTTCTTTGATCCAATTGTTCGAAAATAAAAAGTTATCCCGAGATATTGAAAGGTTGGGAAAAGTAAATCAGTAGCT

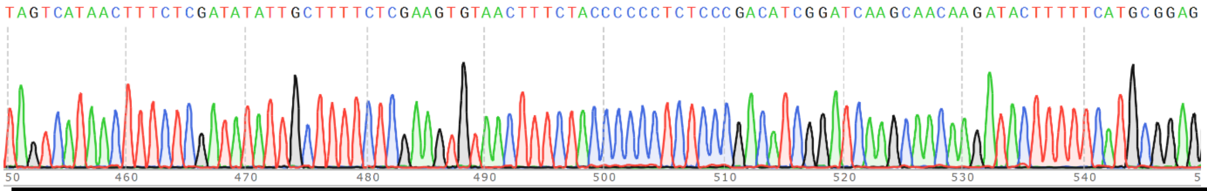


```
PCR_C2 : tagtcataactttctcgatatattgcttttctcgaagtgtaactttctacccccctctcccgacatcggatcaagcaacaagatactttttcatgcggag : 500
r03_C2 : tagtcataactttctcgatatattgcttttctcgaagtgtaactttctacccccctctcccgacatcggatcaagcaacaagatactttttcatgcggag : 500
         TAGTCATAACTTTCTCGATATATTGCTTTTCTCGAAGTGTAACTTTCTACCCCCCTCTCCCGACATCGGATCAAGCAACAAGATACTTTTTCATGCGGAG
```

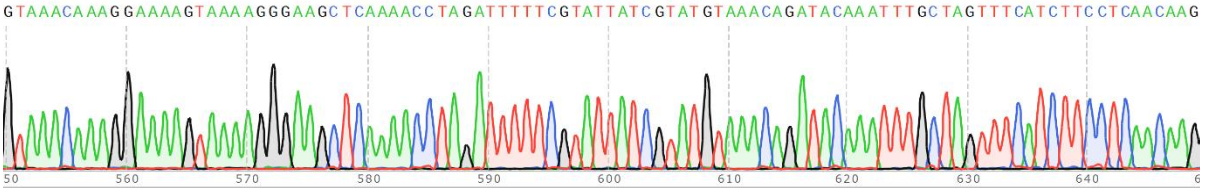


```
PCR_C2 : gtaaacaaaggaaaagtaaaagggaagctcaaaacctagatttttcgtattatcgtatgtaaacagatacaaatttgctagtttcatcttcctcaacaag : 600
r03_C2 : gtaaacaaaggaaaagtaaaagggaagctcaaaacctagatttttcgtattatcgtatgtaaacagatacaaatttgctagtttcatcttcctcaacaag : 600
         GTAAACAAAGGAAAAGTAAAAGGGAAGCTCAAAACCTAGATTTTTCGTATTATCGTATGTAAACAGATACAAATTTGCTAGTTTCATCTTCCTCAACAAG
```

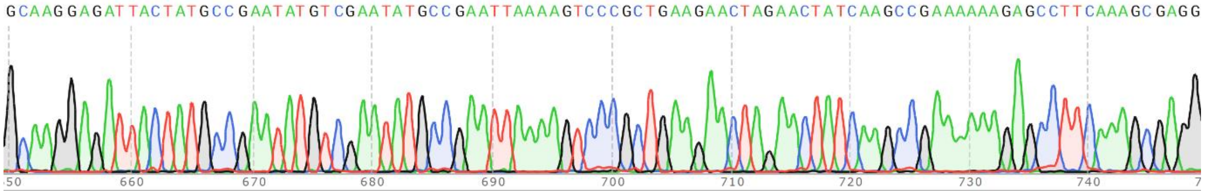


```
PCR_C2 : gcaaggagattactatgccgaatatgtcgaatatgccgaattaaaagtcccgctgaagaactagaactatcaagccgaaaaaagagccttcaaagcgagg : 700
r03_C2 : gcaaggagattactatgccgaatatgtcgaatatgccgaattaaaagtcccgctgaagaactagaactatcaagccgaaaaaagagccttcaaagcgagg : 700
         GCAAGGAGATTACTATGCCGAATATGTCGAATATGCCGAATTAAAAGTCCCGCTGAAGAACTAGAACTATCAAGCCGAAAAAAGAGCCTTCAAAGCGAGG
```

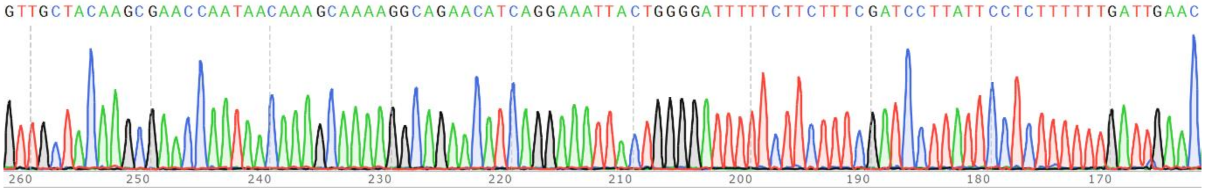


```
PCR_C2 : gttgctacaagcgaaccaataacaaagcaaaaggcagaacatcaggaaattactggggatttttcttctttcgatccttattcctcttttttgattgaac : 800
r03_C2 : gttgctacaagcgaaccaataacaaagcaaaaggcagaacatcaggaaattactggggatttttcttctttcgatccttattcctcttttttgattgaac : 800
         GTTGCTACAAGCGAACCAATAACAAAGCAAAAGGCAGAACATCAGGAAATTACTGGGGATTTTTCTTCTTTCGATCCTTATTCCTCTTTTTTGATTGAAC
```

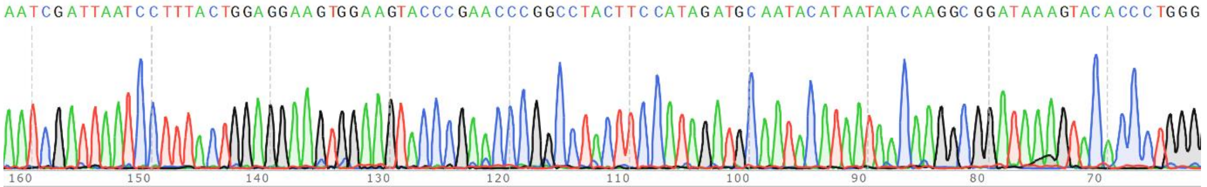


```
PCR_C2 : aatcgattaatcctttactggaggaagtggaagtacccgaacccggcctacttccatagatgcaatacataataacaaggcggataaagtacaccctggg : 900
r03_C2 : aatcgattaatcctttactggaggaagtggaagtacccgaacccggcctacttccatagatgcaatacataataacaaggcggataaagtacaccctggg : 900
         AATCGATTAATCCTTTACTGGAGGAAGTGGAAGTACCCGAACCCGGCCTACTTCCATAGATGCAATACATAATAACAAGGCGGATAAAGTACACCCTGGG
```

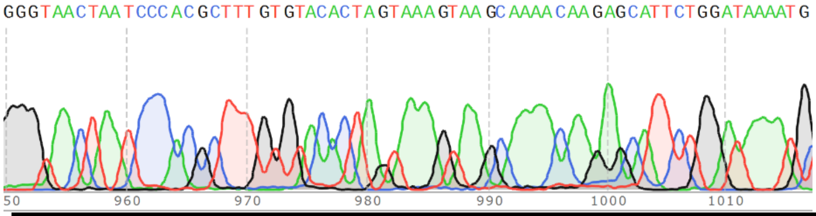


```
PCR_C2 : gggtaactaatcccacgctttgtgtacactagtaaagtaagcaaaacaagagcattctggataaaatg : 968
r03_C2 : gggtaactaatcccacgctttgtgtacactagtaaagtaagcaaaacaagagcattctggataaaatg : 968
         GGGTAACTAATCCCACGCTTTGTGTACACTAGTAAAGTAAGCAAAACAAGAGCATTCTGGATAAAATG
```

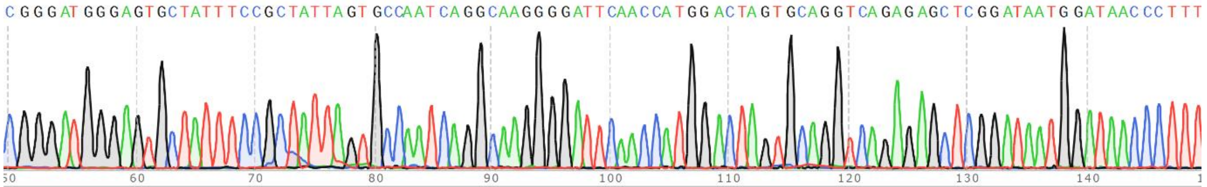


```
PCR_C3 : cgggatgggagtgctatttccgctattagtgccaatcaggcaaggggattcaaccatggactagtgcaggtcagagagctcggataatggataacccttt : 100
r03_C3 : cgggatgggagtgctatttccgctattagtgccaatcaggcaaggggattcaaccatggactagtgcaggtcagagagctcggataatggataacccttt : 100
         CGGGATGGGAGTGCTATTTCCGCTATTAGTGCCAATCAGGCAAGGGGATTCAACCATGGACTAGTGCAGGTCAGAGAGCTCGGATAATGGATAACCCTTT
```

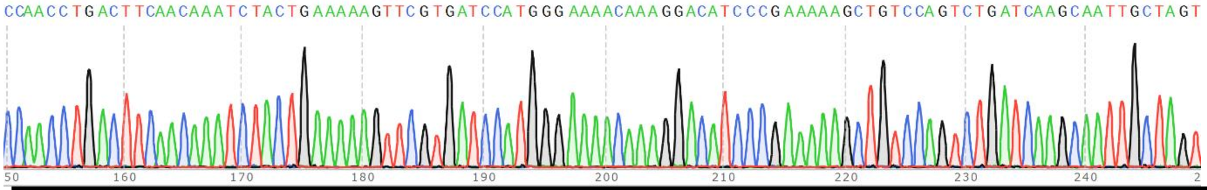


```
PCR_C3 : ccaacctgacttcaacaaatctactgaaaaagttcgtgatccatgggaaaacaaaggacatcccgaaaaagctgtccagtctgatcaagcaattgctagt : 200
r03_C3 : ccaacctgacttcaacaaatctactgaaaaagttcgtgatccatgggaaaacaaaggacatcccgaaaaagctgtccagtctgatcaagcaattgctagt : 200
         CCAACCTGACTTCAACAAATCTACTGAAAAAGTTCGTGATCCATGGGAAAACAAAGGACATCCCGAAAAAGCTGTCCAGTCTGATCAAGCAATTGCTAGT
```

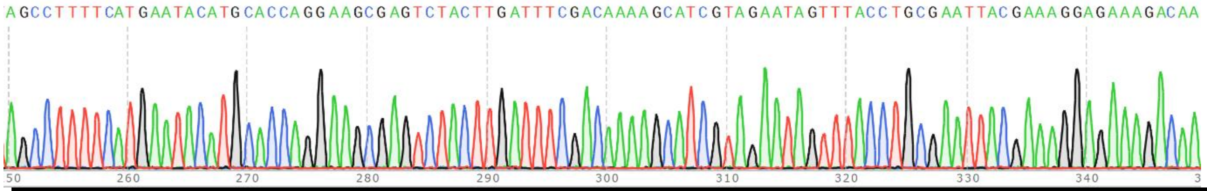


```
PCR_C3 : agccttttcatgaatacatgcaccaggaagcgagtctacttgatttcgacaaaagcatcgtagaatagtttacctgcgaattacgaaaggagaaagacaa : 300
r03_C3 : agccttttcatgaatacatgcaccaggaagcgagtctacttgatttcgacaaaagcatcgtagaatagtttacctgcgaattacgaaaggagaaagacaa : 300
         AGCCTTTTCATGAATACATGCACCAGGAAGCGAGTCTACTTGATTTCGACAAAAGCATCGTAGAATAGTTTACCTGCGAATTACGAAAGGAGAAAGACAA
```

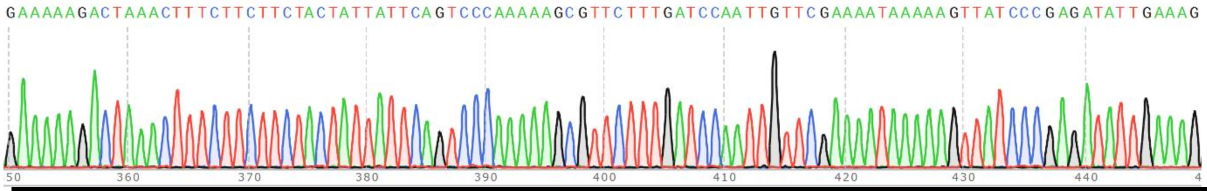


```
PCR_C3 : gaaaaagactaaactttcttcttctactattattcagtcccaaaaagcgttctttgatccaattgttcgaaaataaaaagttatcccgagatattgaaag : 400
r03_C3 : gaaaaagactaaactttcttcttctactattattcagtcccaaaaagcgttctttgatccaattgttcgaaaataaaaagttatcccgagatattgaaag : 400
         GAAAAAGACTAAACTTTCTTCTTCTACTATTATTCAGTCCCAAAAAGCGTTCTTTGATCCAATTGTTCGAAAATAAAAAGTTATCCCGAGATATTGAAAG
```

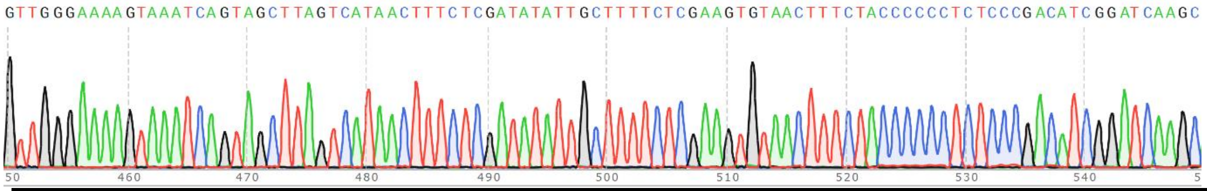


```
PCR_C3 : gttgggaaaagtaaatcagtagcttagtcataactttctcgatatattgcttttctcgaagtgtaactttctacccccctctcccgacatcggatcaagc : 500
r03_C3 : gttgggaaaagtaaatcagtagcttagtcataactttctcgatatattgcttttctcgaagtgtaactttctacccccctctcccgacatcggatcaagc : 500
         GTTGGGAAAAGTAAATCAGTAGCTTAGTCATAACTTTCTCGATATATTGCTTTTCTCGAAGTGTAACTTTCTACCCCCCTCTCCCGACATCGGATCAAGC
```

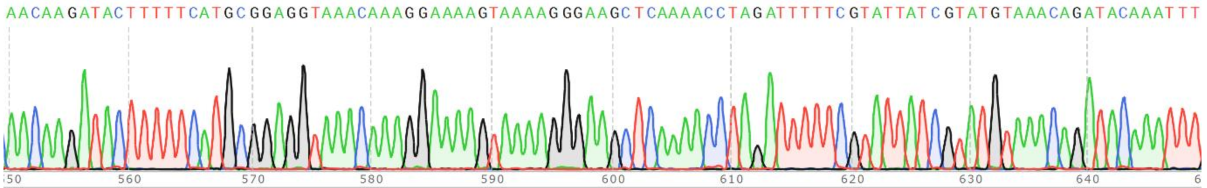


```
PCR_C3 : aacaagatacttttttcatgcggaggtaaacaaaggaaaagtaaaagggaagctcaaaacctagatttttcgtattatcgtatgtaaacagatacaaattt : 600
r03_C3 : aacaagatacttttttcatgcggaggtaaacaaaggaaaagtaaaagggaagctcaaaacctagatttttcgtattatcgtatgtaaacagatacaaattt : 600
         AACAAGATACTTTTTTCATGCGGAGGTAAACAAAGGAAAAGTAAAAGGGAAGCTCAAAACCTAGATTTTTCGTATTATCGTATGTAAACAGATACAAATTT
```

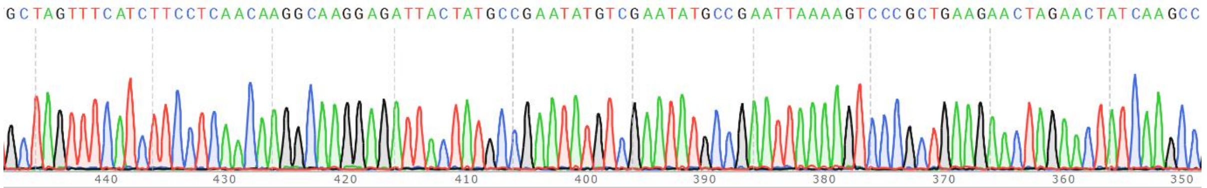


```
PCR_C3 : gctagtttcatcttcctcaacaaggcaaggagattactatgccgaatatgtcgaatatgccgaattaaaagtcccgctgaagaactagaactatcaagcc : 700
r03_C3 : gctagtttcatcttcctcaacaaggcaaggagattactatgccgaatatgtcgaatatgccgaattaaaagtcccgctgaagaactagaactatcaagcc : 700
         GCTAGTTTCATCTTCCTCAACAAGGCAAGGAGATTACTATGCCGAATATGTCGAATATGCCGAATTAAAAGTCCCGCTGAAGAACTAGAACTATCAAGCC
```

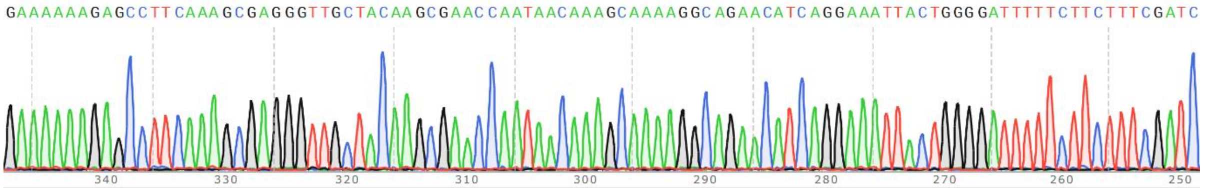


```
PCR_C3 : gaaaaaagagccttcaaagcgagggttgctacaagcgaaccaataacaaagcaaaaggcagaacatcaggaaattactggggatttttcttctttcgatc : 800
r03_C3 : gaaaaaagagccttcaaagcgagggttgctacaagcgaaccaataacaaagcaaaaggcagaacatcaggaaattactggggatttttcttctttcgatc : 800
         GAAAAAAGAGCCTTCAAAGCGAGGGTTGCTACAAGCGAACCAATAACAAAGCAAAAGGCAGAACATCAGGAAATTACTGGGGATTTTTCTTCTTTCGATC
```

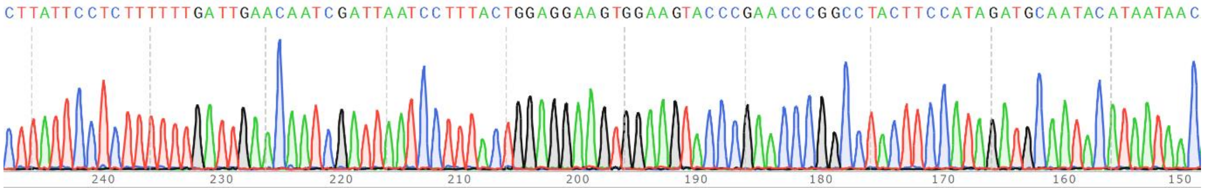


```
PCR_C3 : cttattcctctttttgattgaacaatcgattaatcctttactggaggaagtggaagtacccgaacccggcctacttccatagatgcaatacataataac : 900
r03_C3 : cttattcctctttttgattgaacaatcgattaatcctttactggaggaagtggaagtacccgaacccggcctacttccatagatgcaatacataataac : 900
         CTTATTCCTCTTTTTTGATTGAACAATCGATTAATCCTTTACTGGAGGAAGTGGAAGTACCCGAACCCGGCCTACTTCCATAGATGCAATACATAATAAC
```

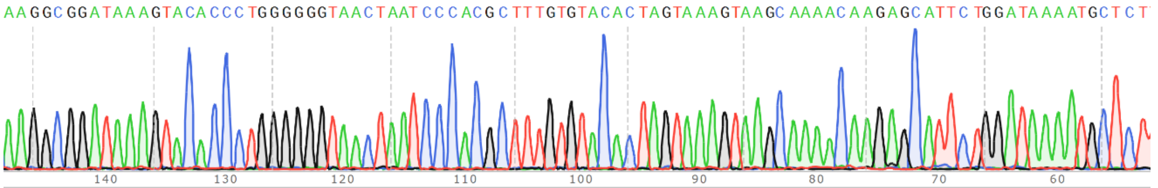


```
PCR_C3 : aaggcggataaagtacaccctggggggtaactaatcccacgctttgtgtacactagtaaagtaagcaaaacaagagcattctggataaaatgctct : 994
r03_C3 : aaggcggataaagtacaccctggggggtaactaatcccacgctttgtgtacactagtaaagtaagcaaaacaagagcattctggataaaatgctct : 996
         AAGGCGGATAAAGTACACCCTGGGGGGTAACTAATCCCACGCTTTGTGTACACTAGTAAAGTAAGCAAAACAAGAGCATTCTGGATAAAATGCTCT
```

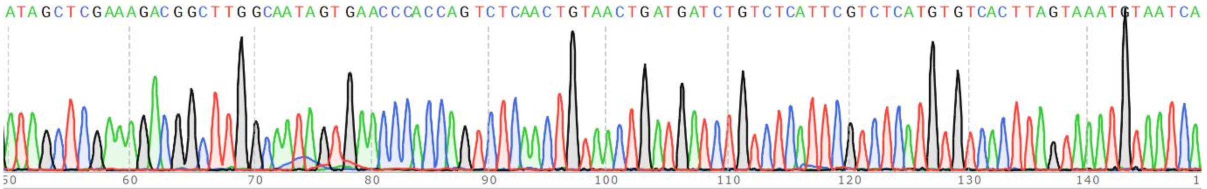


```
r03_C4 : atagctcgaaagacggcttggcaatagtgaacccaccagtctcaactgtaactgatgatctgtctcattcgtctcatgtgtcacttagtaaatgtaatca : 100
PCR_C4 : atagctcgaaagacggcttggcaatagtgaacccaccagtctcaactgtaactgatgatctgtctcattcgtctcatgtgtcacttagtaaatgtaatca : 100
         ATAGCTCGAAAGACGGCTTGGCAATAGTGAACCCACCAGTCTCAACTGTAACTGATGATCTGTCTCATTCGTCTCATGTGTCACTTAGTAAATGTAATCA
```

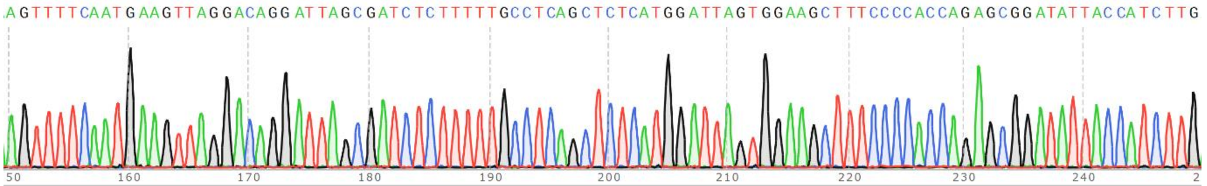


```
r03_C4 : agttttcaatgaagttaggacaggattagcgatctctttttgcctcagctctcatggattagtggaagctttccccaccagagcggatattaccatcttg : 200
PCR_C4 : agttttcaatgaagttaggacaggattagcgatctctttttgcctcagctctcatggattagtggaagctttccccaccagagcggatattaccatcttg : 200
         AGTTTTCAATGAAGTTAGGACAGGATTAGCGATCTCTTTTTGCCTCAGCTCTCATGGATTAGTGGAAGCTTTCCCCACCAGAGCGGATATTACCATCTTG
```

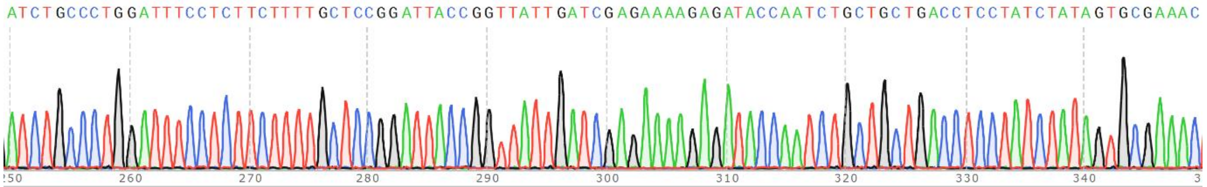


```
r03_C4 : atctgccctggatttcctcttcttttgctccggattaccggttattgatcgagaaaagagataccaatctgctgctgacctcctatctatagtgcgaaac : 300
PCR_C4 : atctgccctggatttcctcttcttttgctccggattaccggttattgatcgagaaaagagataccaatctgctgctgacctcctatctatagtgcgaaac : 300
         ATCTGCCCTGGATTTCCTCTTCTTTTGCTCCGGATTACCGGTTATTGATCGAGAAAAGAGATACCAATCTGCTGCTGACCTCCTATCTATAGTGCGAAAC
```

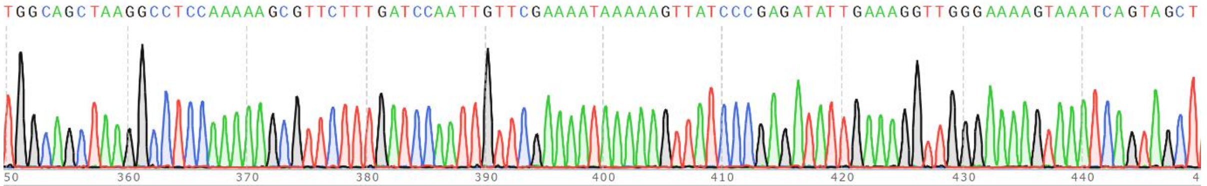


```
r03_C4 : tggcagctaaggcctccaaaaagcgttctttgatccaattgttcgaaaataaaaagttatcccgagatattgaaaggttgggaaaagtaaatcagtagct : 400
PCR_C4 : tggcagctaaggcctccaaaaagcgttctttgatccaattgttcgaaaataaaaagttatcccgagatattgaaaggttgggaaaagtaaatcagtagct : 400
         TGGCAGCTAAGGCCTCCAAAAAGCGTTCTTTGATCCAATTGTTCGAAAATAAAAAGTTATCCCGAGATATTGAAAGGTTGGGAAAAGTAAATCAGTAGCT
```

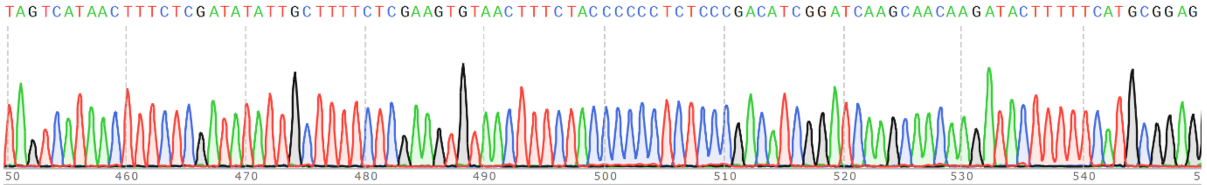


```
r03_C4 : tagtcataactttctcgatatattgcttttctcgaagtgtaactttctacccccctctcccgacatcggatcaagcaacaagatactttttcatgcggag : 500
PCR_C4 : tagtcataactttctcgatatattgcttttctcgaagtgtaactttctacccccctctcccgacatcggatcaagcaacaagatactttttcatgcggag : 500
         TAGTCATAACTTTCTCGATATATTGCTTTTCTCGAAGTGTAACTTTCTACCCCCCTCTCCCGACATCGGATCAAGCAACAAGATACTTTTTCATGCGGAG
```

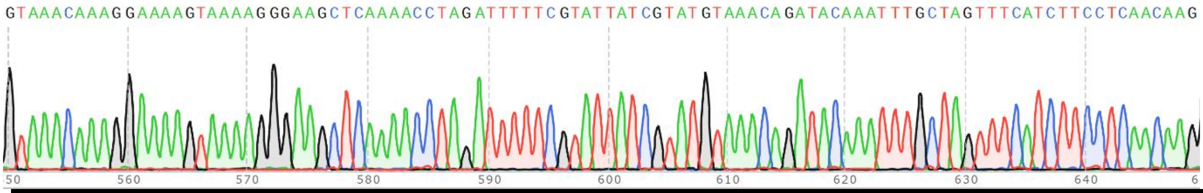

```
r03_C4 : gtaaacaaaggaaaagtaaaagggaagctcaaaacctagatttttcgtattatcgtatgtaaacagatacaaatttgctagtttcatcttcctcaacaag : 600
PCR_C4 : gtaaacaaaggaaaagtaaaagggaagctcaaaacctagatttttcgtattatcgtatgtaaacagatacaaatttgctagtttcatcttcctcaacaag : 600
         GTAAACAAAGGAAAAGTAAAAGGGAAGCTCAAAACCTAGATTTTTCGTATTATCGTATGTAAACAGATACAAATTTGCTAGTTTCATCTTCCTCAACAAG
```

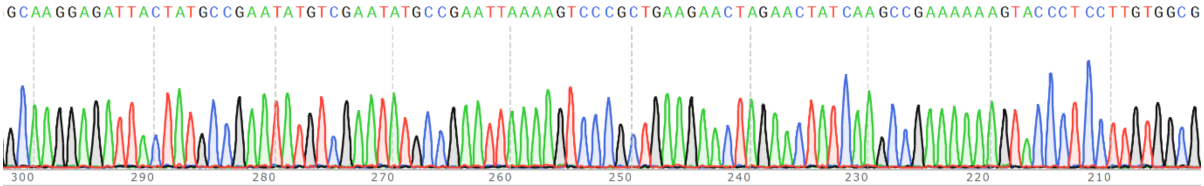

```
r03_C4 : gcaaggagattactatgccgaatatgtcgaatatgccgaattaaaagtcccgctgaagaactagaactatcaagccgaaaaaagtaccctccttgtggcg : 700
PCR_C4 : gcaaggagattactatgccgaatatgtcgaatatgccgaattaaaagtcccgctgaagaactagaactatcaagccgaaaaaagtaccctccttgtggcg : 700
         GCAAGGAGATTACTATGCCGAATATGTCGAATATGCCGAATTAAAAGTCCCGCTGAAGAACTAGAACTATCAAGCCGAAAAAAGTACCCTCCTTGTGGCG
```

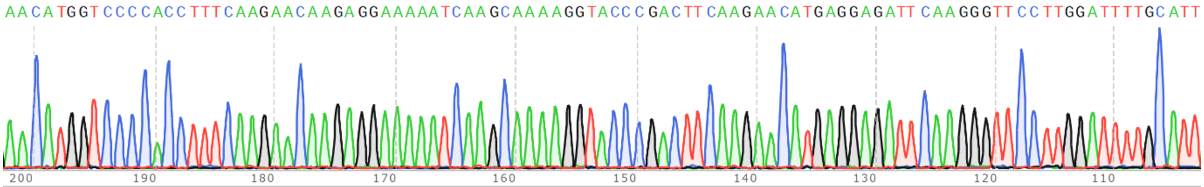

```
r03_C4 : aacatggtccccacctttcaagaacaagaggaaaaatcaagcaaaaggtacccgacttcaagaacatgaggagattcaagggttccttggattttgcatt : 800
PCR_C4 : aacatggtccccacctttcaagaacaagaggaaaaatcaagcaaaaggtacccgacttcaagaacatgaggagattcaagggttccttggattttgcatt : 800
         AACATGGTCCCCACCTTTCAAGAACAAGAGGAAAAATCAAGCAAAAGGTACCCGACTTCAAGAACATGAGGAGATTCAAGGGTTCCTTGGATTTTGCATT
```

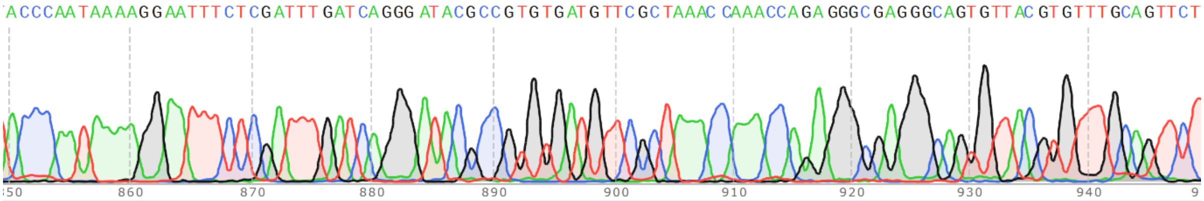

```
r03_C4 : acccaataaaaggaatttctcgatttgatcagggatacgccgtgtgatgttcgctaaaccaaaccagagggcgagggcagtgttacgtgtttgcagttct : 900
PCR_C4 : acccaataaaaggaatttctcgatttgatcagggatacgccgtgtgatgttcgctaaaccaaaccagagggcgagggcagtgttacgtgtttgcagttct : 900
         ACCCAATAAAAGGAATTTCTCGATTTGATCAGGGATACGCCGTGTGATGTTCGCTAAACCAAACCAGAGGGCGAGGGCAGTGTTACGTGTTTGCAGTTCT
```

d

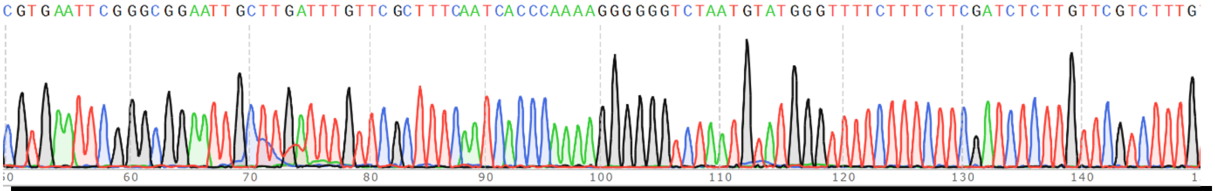

```
PCR_C1 : cgtgaattcgggcggaattgcttgatttgttcgctttcaatcacccaaaagggggggtctaatgtatgggttttctttcttcgatctcttgttcgtctttg : 100
r04_C1 : cgtgaattcgggcggaattgcttgatttgttcgctttcaatcacccaaaagggggggtctaatgtatgggttttctttcttcgatctcttgttcgtctttg : 100
         CGTGAATTCGGGCGGAATTGCTTGATTTGTTCGCTTTCAATCACCCAAAAGGGGGGGTCTAATGTATGGGTTTTCTTTCTTCGATCTCTTGTTCGTCTTTG
```

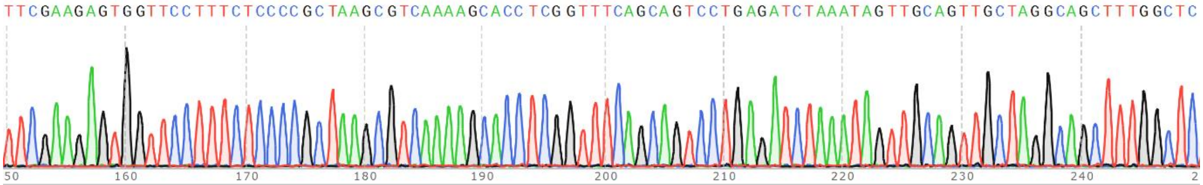

```
PCR_C1 : ttcgaagagtggttcctttctccccgctaagcgtcaaaagcacctcggtttcagcagtcctgagatctaaatagttgcagttgctaggcagctttggctc : 200
r04_C1 : ttcgaagagtggttcctttctccccgctaagcgtcaaaagcacctcggtttcagcagtcctgagatctaaatagttgcagttgctaggcagctttggctc : 200
         TTCGAAGAGTGGTTCCTTTCTCCCCGCTAAGCGTCAAAAGCACCTCGGTTTCAGCAGTCCTGAGATCTAAATAGTTGCAGTTGCTAGGCAGCTTTGGCTC
```

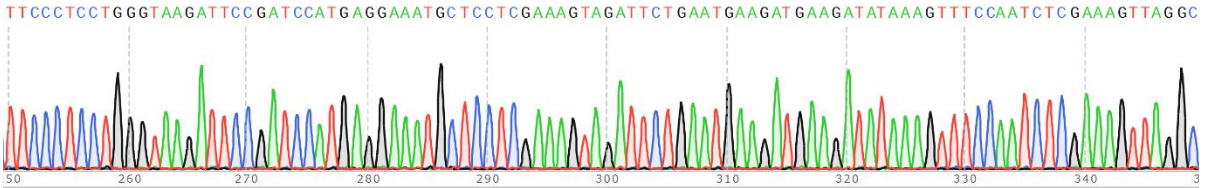


```
PCR_C1 : ttccctcctgggtaagattccgatccatgaggaaatgctcctcgaaagtagattctgaatgaagatgaagatataaagtttccaatctcgaaagttaggc : 300
r04_C1 : ttccctcctgggtaagattccgatccatgaggaaatgctcctcgaaagtagattctgaatgaagatgaagatataaagtttccaatctcgaaagttaggc : 300
         TTCCCTCCTGGGTAAGATTCCGATCCATGAGGAAATGCTCCTCGAAAGTAGATTCTGAATGAAGATGAAGATATAAAGTTTCCAATCTCGAAAGTTAGGC
```

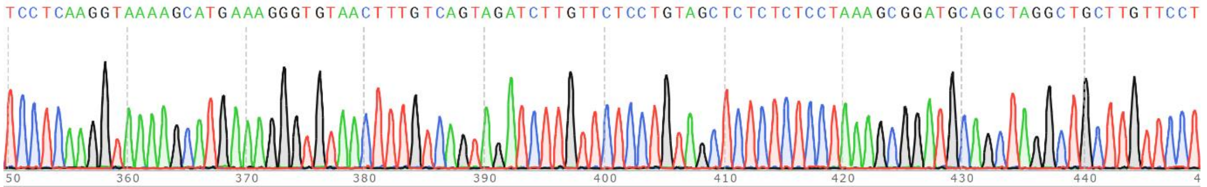


```
PCR_C1 : tcctcaaggtaaaagcatgaaagggtgtaactttgtcagtagatcttgttctcctgtagctctctctcctaaagcggatgcagctaggctgcttgttcct : 400
r04_C1 : tcctcaaggtaaaagcatgaaagggtgtaactttgtcagtagatcttgttctcctgtagctctctctcctaaagcggatgcagctaggctgcttgttcct : 400
         TCCTCAAGGTAAAAGCATGAAAGGGTGTAACTTTGTCAGTAGATCTTGTTCTCCTGTAGCTCTCTCTCCTAAAGCGGATGCAGCTAGGCTGCTTGTTCCT
```

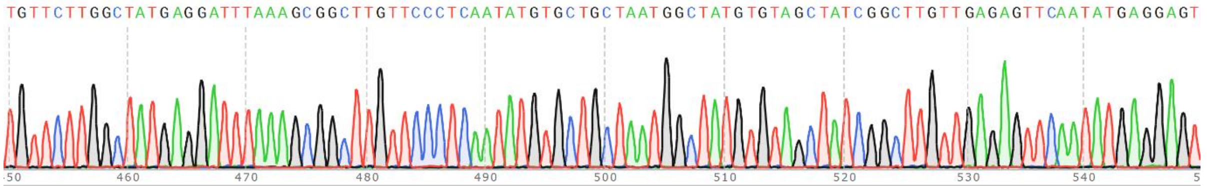


```
PCR_C1 : tgttcttggctatgaggatttaaagcggcttgttccctcaatatgtgctgctaatggctatgtgtagctatcggcttgttgagagttcaatatgaggagt : 500
r04_C1 : tgttcttggctatgaggatttaaagcggcttgttccctcaatatgtgctgctaatggctatgtgtagctatcggcttgttgagagttcaatatgaggagt : 500
         TGTTCTTGGCTATGAGGATTTAAAGCGGCTTGTTCCCTCAATATGTGCTGCTAATGGCTATGTGTAGCTATCGGCTTGTTGAGAGTTCAATATGAGGAGT
```

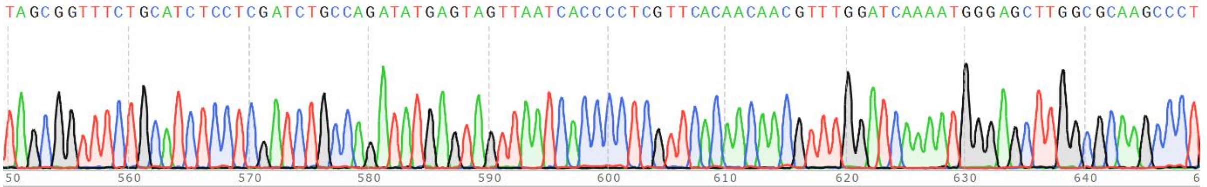


```
PCR_C1 : tagcggtttctgcatctcctcgatctgccagatatgagtagttaatcacccctcgttcacaacaacgtttggatcaaaatgggagcttggcgcaagccct : 600
r04_C1 : tagcggtttctgcatctcctcgatctgccagatatgagtagttaatcacccctcgttcacaacaacgtttggatcaaaatgggagcttggcgcaagccct : 600
         TAGCGGTTTCTGCATCTCCTCGATCTGCCAGATATGAGTAGTTAATCACCCCTCGTTCACAACAACGTTTGGATCAAAATGGGAGCTTGGCGCAAGCCCT
```

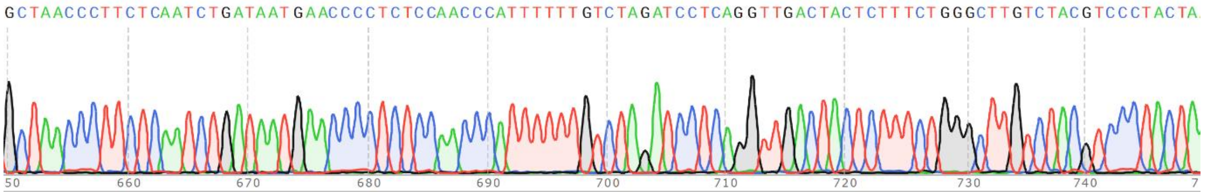


```
PCR_C1 : gctaacccttctcaatctgataatgaacccctctccaacccattttttgtctagatcctcaggttgactactctttctgggcttgtctacgtccctacta : 700
r04_C1 : gctaacccttctcaatctgataatgaacccctctccaacccattttttgtctagatcctcaggttgactactctttctgggcttgtctacgtccctacta : 700
         GCTAACCCTTCTCAATCTGATAATGAACCCCTCTCCAACCCATTTTTTGTCTAGATCCTCAGGTTGACTACTCTTTCTGGGCTTGTCTACGTCCCTACTA
```

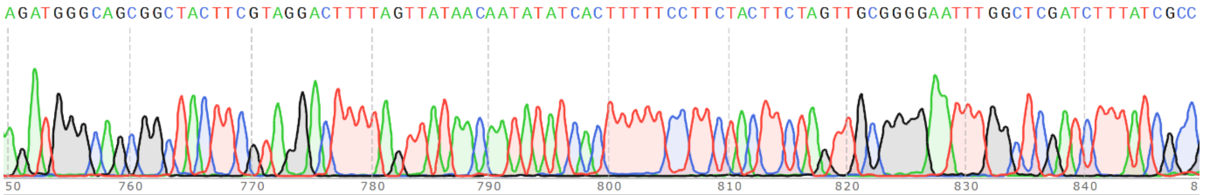


```
PCR_C1 : agatgggcagcggctacttcgtaggacttttagttataacaatatatcactttttccttctacttctagttgcggggaatttggctcgatctttatcgcc : 800
r04_C1 : agatgggcagcggctacttcgtaggacttttagttataacaatatatcactttttccttctacttctagttgcggggaatttggctcgatctttatcgcc : 800
         AGATGGGCAGCGGCTACTTCGTAGGACTTTTAGTTATAACAATATATCACTTTTTCCTTCTACTTCTAGTTGCGGGGAATTTGGCTCGATCTTTATCGCC
```

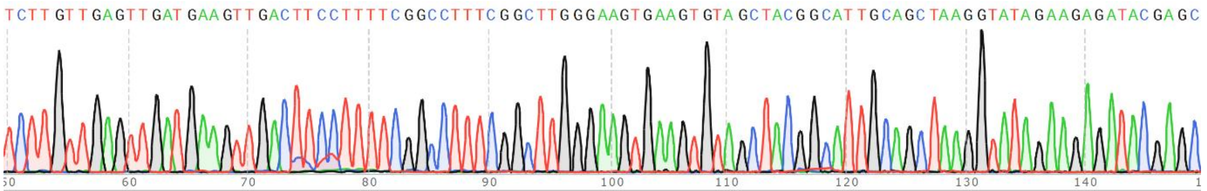


```
PCR_C2 : tcttgttgagttgatgaagttgacttccttttcggcctttcggcttgggaagtgaagtgtagctacggcattgcagctaaggtatagaagagatacgagc : 100
r04_C2 : tcttgttgagttgatgaagttgacttccttttcggcctttcggcttgggaagtgaagtgtagctacggcattgcagctaaggtatagaagagatacgagc : 100
         TCTTGTTGAGTTGATGAAGTTGACTTCCTTTTCGGCCTTTCGGCTTGGGAAGTGAAGTGTAGCTACGGCATTGCAGCTAAGGTATAGAAGAGATACGAGC
```

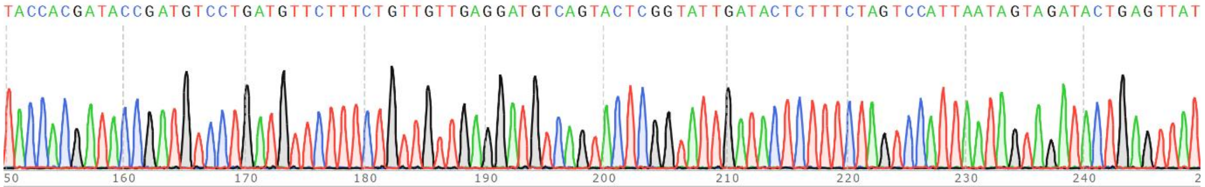


```
PCR_C2 : taccacgataccgatgtcctgatgttctttctgttgttgaggatgtcagtactcggtattgatactctttctagtccattaatagtagatactgagttat : 200
r04_C2 : taccacgataccgatgtcctgatgttctttctgttgttgaggatgtcagtactcggtattgatactctttctagtccattaatagtagatactgagttat : 200
         TACCACGATACCGATGTCCTGATGTTCTTTCTGTTGTTGAGGATGTCAGTACTCGGTATTGATACTCTTTCTAGTCCATTAATAGTAGATACTGAGTTAT
```

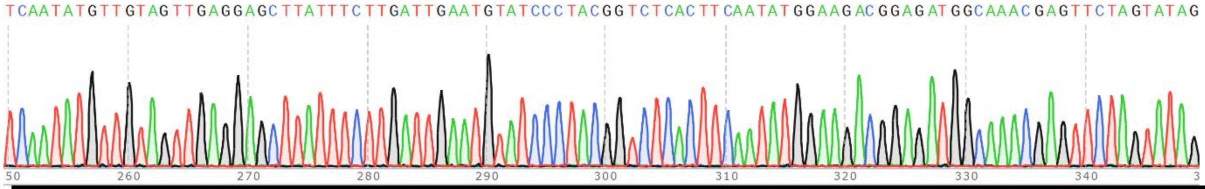


```
PCR_C2 : tcaatatgttgtagttgaggagcttatttcttgattgaatgtatccctacggtctcacttcaatatggaagacggagatggcaaacgagttctagtatag : 300
r04_C2 : tcaatatgttgtagttgaggagcttatttcttgattgaatgtatccctacggtctcacttcaatatggaagacggagatggcaaacgagttctagtatag : 300
         TCAATATGTTGTAGTTGAGGAGCTTATTTCTTGATTGAATGTATCCCTACGGTCTCACTTCAATATGGAAGACGGAGATGGCAAACGAGTTCTAGTATAG
```

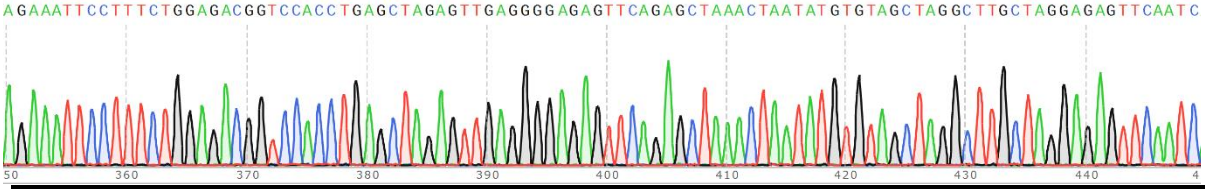


```
PCR_C2 : agaaattcctttctggagacggtccacctgagctagagttgaggggagagttcagagctaaactaatatgtgtagctaggcttgctaggagagttcaatc : 400
r04_C2 : agaaattcctttctggagacggtccacctgagctagagttgaggggagagttcagagctaaactaatatgtgtagctaggcttgctaggagagttcaatc : 400
         AGAAATTCCTTTCTGGAGACGGTCCACCTGAGCTAGAGTTGAGGGGAGAGTTCAGAGCTAAACTAATATGTGTAGCTAGGCTTGCTAGGAGAGTTCAATC
```

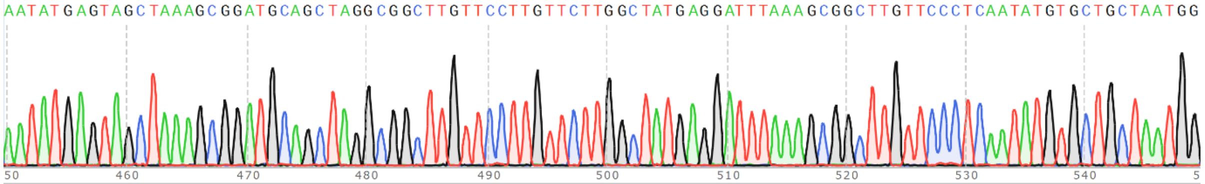


```
PCR_C2 : aatatgagtagctaaagcggatgcagctaggcggcttgttccttgttcttggctatgaggatttaaagcggcttgttccctcaatatgtgctgctaatgg : 500
r04_C2 : aatatgagtagctaaagcggatgcagctaggcggcttgttccttgttcttggctatgaggatttaaagcggcttgttccctcaatatgtgctgctaatgg : 500
         AATATGAGTAGCTAAAGCGGATGCAGCTAGGCGGCTTGTTCCTTGTTCTTGGCTATGAGGATTTAAAGCGGCTTGTTCCCTCAATATGTGCTGCTAATGG
```

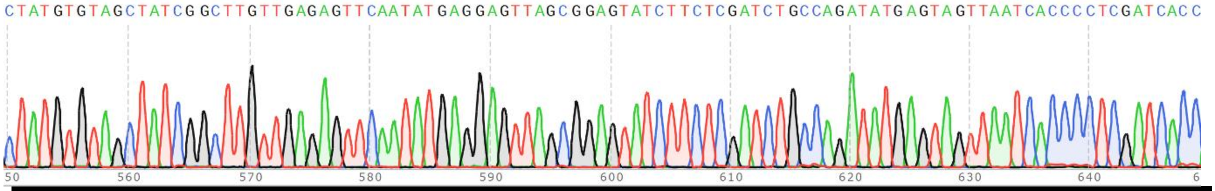


```
PCR_C2 : ctatgtgtagctatcggcttgttgagagttcaatatgaggagttagcggagtatcttctcgatctgccagatatgagtagttaatcacccctcgatcacc : 600
r04_C2 : ctatgtgtagctatcggcttgttgagagttcaatatgaggagttagcggagtatcttctcgatctgccagatatgagtagttaatcacccctcgatcacc : 600
         CTATGTGTAGCTATCGGCTTGTTGAGAGTTCAATATGAGGAGTTAGCGGAGTATCTTCTCGATCTGCCAGATATGAGTAGTTAATCACCCCTCGATCACC
```

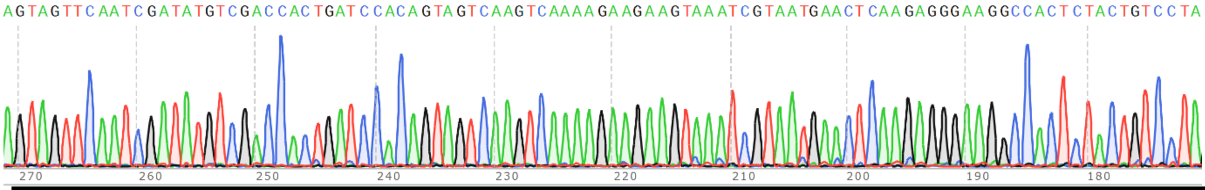


```
PCR_C2 : agtagttcaatcgatatgtcgaccactgatccacagtagtcaagtcaaaagaagaagtaaatcgtaatgaactcaagagggaaggccactctactgtcct : 700
r04_C2 : agtagttcaatcgatatgtcgaccactgatccacagtagtcaagtcaaaagaagaagtaaatcgtaatgaactcaagagggaaggccactctactgtcct : 700
         AGTAGTTCAATCGATATGTCGACCACTGATCCACAGTAGTCAAGTCAAAAGAAGAAGTAAATCGTAATGAACTCAAGAGGGAAGGCCACTCTACTGTCCT
```

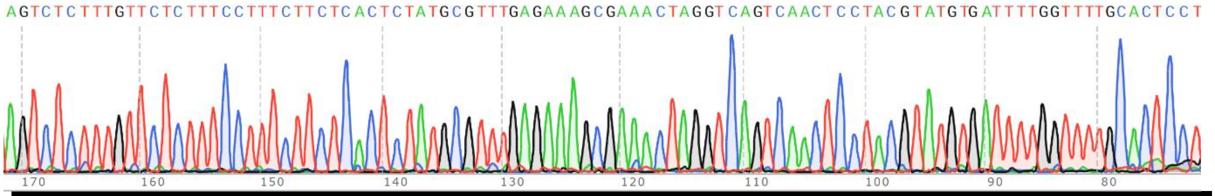


```
PCR_C2 : agtctctttgttctctttcctttcttctcactctatgcgtttgagaaagcgaaactaggtcagtcaactcctacgtatgtgattttggttttgcactcct : 800
r04_C2 : agtctctttgttctctttcctttcttctcactctatgcgtttgagaaagcgaaactaggtcagtcaactcctacgtatgtgattttggttttgcactcct : 800
```

AGTCTCTTTGTTCTCTTTCCTTTCTTCTCACTCTATGCGTTTGAGAAAGCGAAACTAGGTCAGTCAACTCCTACGTATGTGATTTTGGTTTTGCACTCCT

GCACTGCCGGCTCTGGAGAGAGTGATCCTCGGGTGACTGAACTACCCTACCTTGAGAGTA GGGGTAGGCCTAAGCTCAATGCCCT

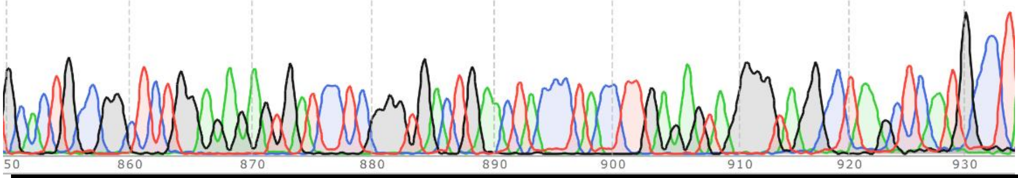

```
PCR_C2 : gcactgccggctctggagagagtgatcctcgggtgactgaactaccctaccttgagagtaggggtaggcctaagctcaatgccct : 885
r04_C2 : gcactgccggctctggagagagtgatcctcgggtgactgaactaccctaccttgagagtaggggtaggcctaagctcaatgccct : 885
         GCACTGCCGGCTCTGGAGAGAGTGATCCTCGGGTGACTGAACTACCCTACCTTGAGAGTAGGGGTAGGCCTAAGCTCAATGCCCT
```

CGTGAATTCGGGCGGAATTGCTTGATTTGTTCGCTTTCAATCACCCAAAAGGGGGGTCTAATGTATGGGTTTTCTTTCTTCGATCTCTTGTTCGTCTTTG

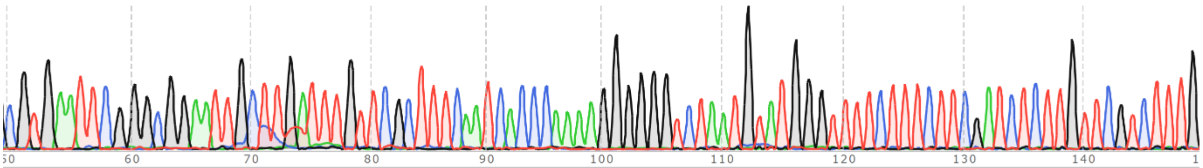

```
PCR_C3 : cgtgaattcgggcggaattgcttgatttgttcgctttcaatcacccaaaaggggggtctaatgtatgggttttctttcttcgatctcttgttcgtctttg : 100
r04_C3 : cgtgaattcgggcggaattgcttgatttgttcgctttcaatcacccaaaaggggggtctaatgtatgggttttctttcttcgatctcttgttcgtctttg : 100
         CGTGAATTCGGGCGGAATTGCTTGATTTGTTCGCTTTCAATCACCCAAAAGGGGGGTCTAATGTATGGGTTTTCTTTCTTCGATCTCTTGTTCGTCTTTG
```

TTCGAAGAGTGGTTCCTTTCTCCCCGCTAAGCGTCAAAAGCACCTCGGTTTCAGCAGTCCTGAGATCTAAATAGTTGCAGTTGCTAGGCAGCTTTGGCTC

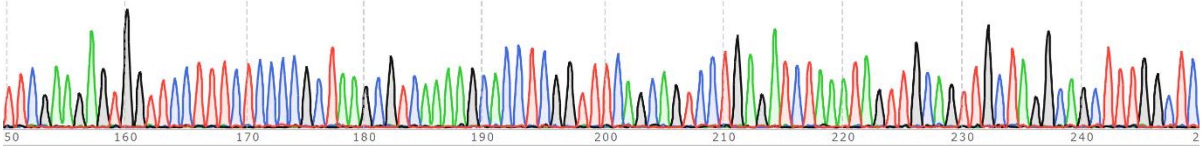

```
PCR_C3 : ttcgaagagtggttcctttctccccgctaagcgtcaaaagcacctcggtttcagcagtcctgagatctaaatagttgcagttgctaggcagctttggctc : 200
r04_C3 : ttcgaagagtggttcctttctccccgctaagcgtcaaaagcacctcggtttcagcagtcctgagatctaaatagttgcagttgctaggcagctttggctc : 200
         TTCGAAGAGTGGTTCCTTTCTCCCCGCTAAGCGTCAAAAGCACCTCGGTTTCAGCAGTCCTGAGATCTAAATAGTTGCAGTTGCTAGGCAGCTTTGGCTC
```

TTCCCTCCTGGGTAAGATTCCGATCCATGAGGAAATGCTCCTCGAAAGTAGATTCTGAATGAAGATGAAGATATAAAGTTTCCAATCTCGAAAGTTAGGC

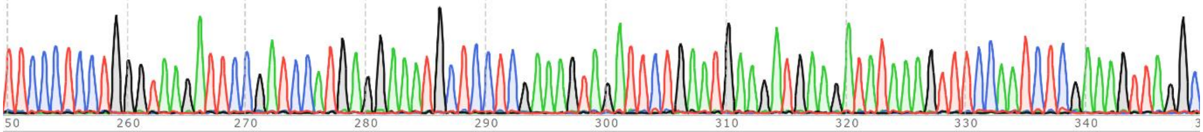

```
PCR_C3 : ttccctcctgggtaagattccgatccatgaggaaatgctcctcgaaagtagattctgaatgaagatgaagatataaagtttccaatctcgaaagttaggc : 300
r04_C3 : ttccctcctgggtaagattccgatccatgaggaaatgctcctcgaaagtagattctgaatgaagatgaagatataaagtttccaatctcgaaagttaggc : 300
         TTCCCTCCTGGGTAAGATTCCGATCCATGAGGAAATGCTCCTCGAAAGTAGATTCTGAATGAAGATGAAGATATAAAGTTTCCAATCTCGAAAGTTAGGC
```

TCCTCAAGGTAAAAGCATGAAAGGGTGTAACTTTGTCAGTAGATCTTGTTCTCCTGTAGCTCTCTCTCCTAAAGCGGATGCAGCTAGGCTGCTTGTTCCT

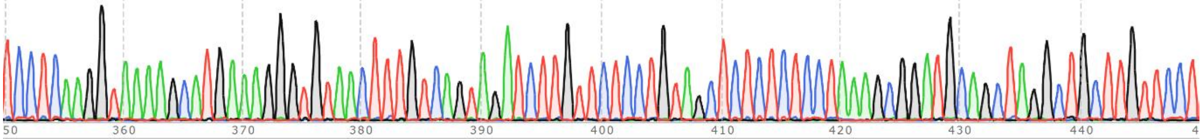

```
PCR_C3 : tcctcaaggtaaaagcatgaaagggtgtaactttgtcagtagatcttgttctcctgtagctctctctcctaaagcggatgcagctaggctgcttgttcct : 400
r04_C3 : tcctcaaggtaaaagcatgaaagggtgtaactttgtcagtagatcttgttctcctgtagctctctctcctaaagcggatgcagctaggctgcttgttcct : 400
         TCCTCAAGGTAAAAGCATGAAAGGGTGTAACTTTGTCAGTAGATCTTGTTCTCCTGTAGCTCTCTCTCCTAAAGCGGATGCAGCTAGGCTGCTTGTTCCT
```

TGTTCTTGGCTATGAGGATTTAAAGCGGCTTGTTCCCTCAATATGTGCTGCTAATGGCTATGTGTAGCTATCGGCTTGTTGAGAGTTCAATATGAGGAGT

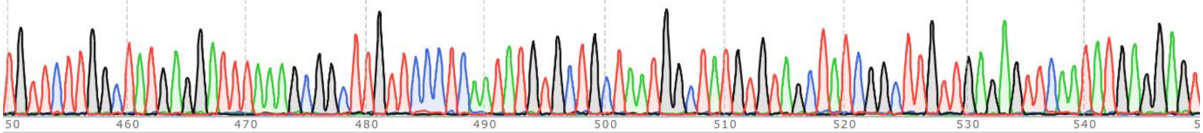

```
PCR_C3 : tgttcttggctatgaggatttaaagcggcttgttccctcaatatgtgctgctaatggctatgtgtagctatcggcttgttgagagttcaatatgaggagt : 500
r04_C3 : tgttcttggctatgaggatttaaagcggcttgttccctcaatatgtgctgctaatggctatgtgtagctatcggcttgttgagagttcaatatgaggagt : 500
         TGTTCTTGGCTATGAGGATTTAAAGCGGCTTGTTCCCTCAATATGTGCTGCTAATGGCTATGTGTAGCTATCGGCTTGTTGAGAGTTCAATATGAGGAGT
```

TAGCGG AGTATCTTCTCGATCTGCCAGATATGAGTAGTTAATCACCCCTCGATCACCAGTAGTTCAATCGATATGTCGACCACTGATCCACAGTAG

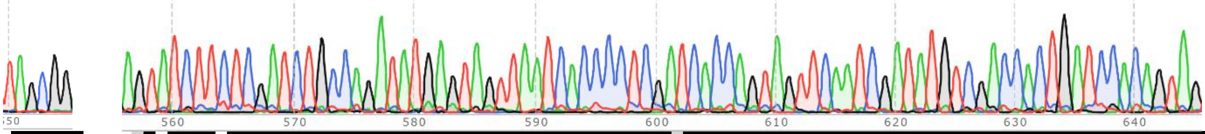

```
PCR_C3 : tagcgg----agtatcttctcgatctgccagatatgagtagttaatcacccctcgatcaccagtagttcaatcgatatgtcgaccactgatccacagtag : 596
r04_C3 : tagcggtttctgcatctccctcgatctgccagatatgagtagttaatcacccctcgttcaccagtagttcaatcgatatgtcgaccactgatccacagtag : 600
```

TAGCGG G ATCT CTCGATCTGCCAGATATGAGTAGTTAATCACCCCTCG TCACCAGTAGTTCAATCGATATGTCGACCACTGATCCACAGTAG

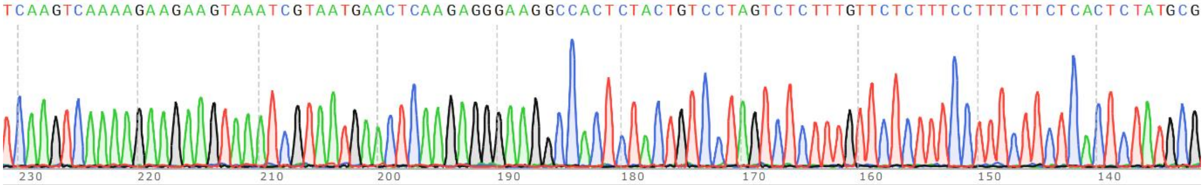

PCR_C3 : tcaagtcaaaagaagaagtaaatcgtaatgaactcaagagggaaggccactctactgtcctagtctctttgttctctttcctttcttctcactctatgcg : 696
r04_C3 : tcaagtcaaaagaagaagtaaatcgtaatgaactcaagagggaaggccactctactgtcctagtctctttgttctctttcctttcttctcactctatgcg : 700
TCAAGTCAAAAGAAGAAGTAAATCGTAATGAACTCAAGAGGGAAGGCCACTCTACTGTCCTAGTCTCTTTGTTCTCTTTCCTTTCTTCTCACTCTATGCG

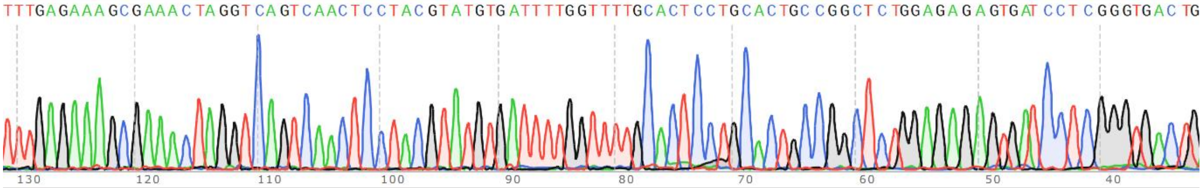

PCR_C3 : tttgagaaagcgaaactaggtcagtcaactcctacgtatgtgattttggttttgcactcctgcactgccggctctggagagagtgatcctcgggtgactg : 796
r04_C3 : tttgagaaagcgaaactaggtcagtcaactcctacgtatgtgattttggttttgcactcctgcactgccggctctggagagagtgatcctcgggtgactg : 800
TTTGAGAAAGCGAAACTAGGTCAGTCAACTCCTACGTATGTGATTTTGGTTTTGCACTCCTGCACTGCCGGCTCTGGAGAGAGTGATCCTCGGGTGACTG

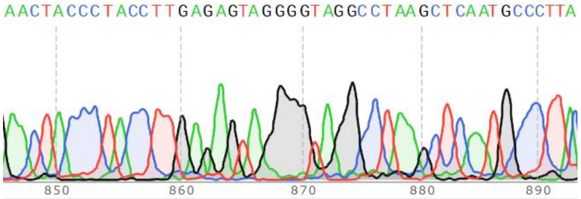

PCR_C3 : aactaccctaccttgagagtaggggtaggcctaagctcaatgccctta : 844
r04_C3 : aactaccctaccttgagagtaggggtaggcctaagctcaatgccctta : 848
AACTACCCTACCTTGAGAGTAGGGGTAGGCCTAAGCTCAATGCCCTTA

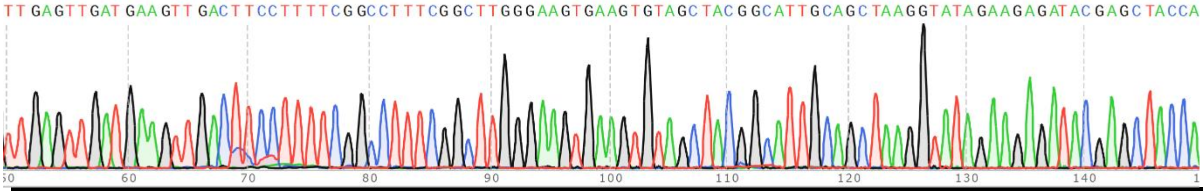

PCR_C4 : ttgagttgatgaagttgacttccttttcggcctttcggcttgggaagtgaagtgtagctacggcattgcagctaaggtatagaagagatacgagctacca : 100
r04_C4 : ttgagttgatgaagttgacttccttttcggcctttcggcttgggaagtgaagtgtagctacggcattgcagctaaggtatagaagagatacgagctacca : 100
TTGAGTTGATGAAGTTGACTTCCTTTTCGGCCTTTCGGCTTGGGAAGTGAAGTGTAGCTACGGCATTGCAGCTAAGGTATAGAAGAGATACGAGCTACCA

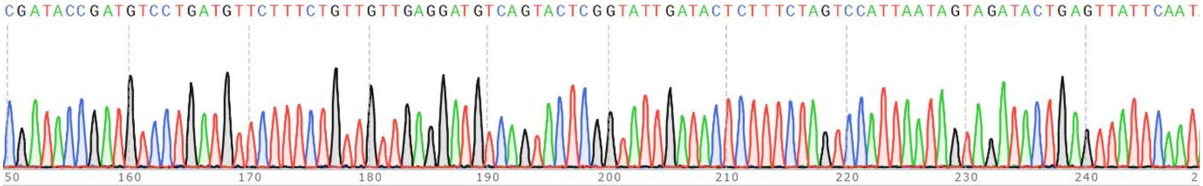

PCR_C4 : cgataccgatgtcctgatgttctttctgttgttgaggatgtcagtactcggtattgatactctttctagtccattaatagtagatactgagttattcaat : 200
r04_C4 : cgataccgatgtcctgatgttctttctgttgttgaggatgtcagtactcggtattgatactctttctagtccattaatagtagatactgagttattcaat : 200
CGATACCGATGTCCTGATGTTCTTTCTGTTGTTGAGGATGTCAGTACTCGGTATTGATACTCTTTCTAGTCCATTAATAGTAGATACTGAGTTATTCAAT

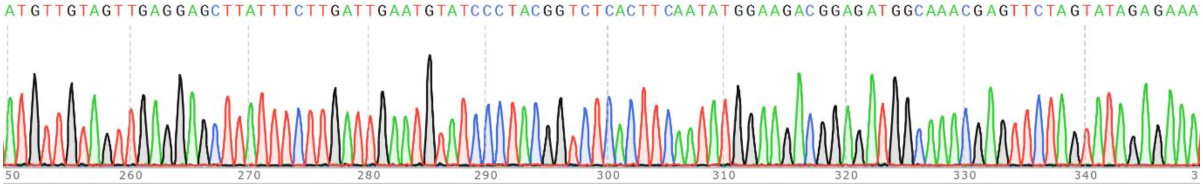

PCR_C4 : atgttgtagttgaggagcttatttcttgattgaatgtatccctacggtctcacttcaatatggaagacggagatggcaaacgagttctagtatagagaaa : 300
r04_C4 : atgttgtagttgaggagcttatttcttgattgaatgtatccctacggtctcacttcaatatggaagacggagatggcaaacgagttctagtatagagaaa : 300
ATGTTGTAGTTGAGGAGCTTATTTCTTGATTGAATGTATCCCTACGGTCTCACTTCAATATGGAAGACGGAGATGGCAAACGAGTTCTAGTATAGAGAAA

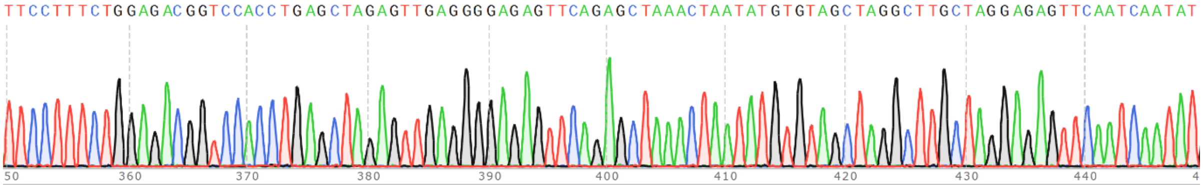

PCR_C4 : ttcctttctggagacggtccacctgagctagagttgaggggagagttcagagctaaactaatatgtgtagctaggcttgctaggagagttcaatcaatat : 400
r04_C4 : ttcctttctggagacggtccacctgagctagagttgaggggagagttcagagctaaactaatatgtgtagctaggcttgctaggagagttcaatcaatat : 400

```
TTCCTTTCTGGAGACGGTCCACCTGAGCTAGAGTTGAGGGGAGAGTTCAGAGCTAAACTAATATGTGTAGCTAGGCTTGCTAGGAGAGTTCAATCAATAT
```

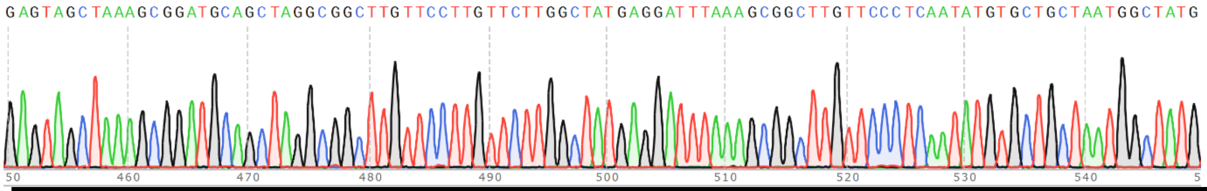


```
PCR_C4 : gagtagctaaagcggatgcagctaggcggcttgttccttgttcttggctatgaggatttaaagcggcttgttccctcaatatgtgctgctaatggctatg : 500
r04_C4 : gagtagctaaagcggatgcagctaggcggcttgttccttgttcttggctatgaggatttaaagcggcttgttccctcaatatgtgctgctaatggctatg : 500
         GAGTAGCTAAAGCGGATGCAGCTAGGCGGCTTGTTCCTTGTTCTTGGCTATGAGGATTTAAAGCGGCTTGTTCCCTCAATATGTGCTGCTAATGGCTATG
```

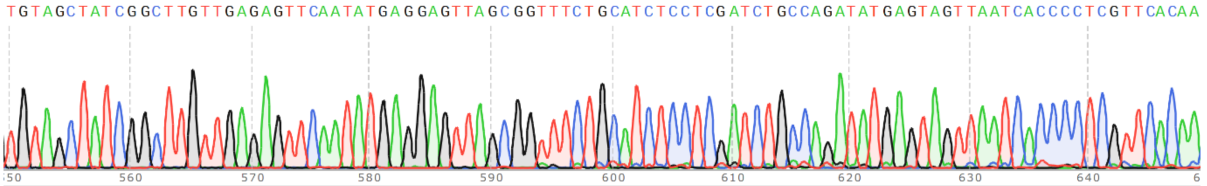


```
PCR_C4 : tgtagctatcggcttgttgagagttcaatatgaggagttagcggtttctgcatctcctcgatctgccagatatgagtagttaatcacccctcgttcacaa : 600
r04_C4 : tgtagctatcggcttgttgagagttcaatatgaggagttagcgg----agtatctttctcgatctgccagatatgagtagttaatcacccctcgatcacaa : 596
         TGTAGCTATCGGCTTGTTGAGAGTTCAATATGAGGAGTTAGCGG      G ATCT  CTCGATCTGCCAGATATGAGTAGTTAATCACCCCTCG TCACAA
```

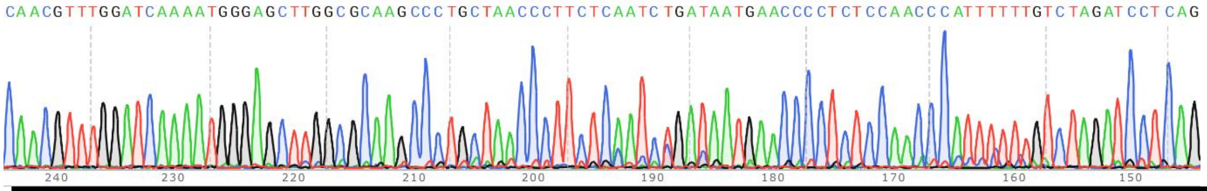


```
PCR_C4 : caacgtttggatcaaaatgggagcttggcgcaagccctgctaacccttctcaatctgataatgaacccctctccaacccattttttgtctagatcctcag : 700
r04_C4 : caacgtttggatcaaaatgggagcttggcgcaagccctgctaacccttctcaatctgataatgaacccctctccaacccattttttgtctagatcctcag : 696
         CAACGTTTGGATCAAAATGGGAGCTTGGCGCAAGCCCTGCTAACCCTTCTCAATCTGATAATGAACCCCTCTCCAACCCATTTTTTGTCTAGATCCTCAG
```

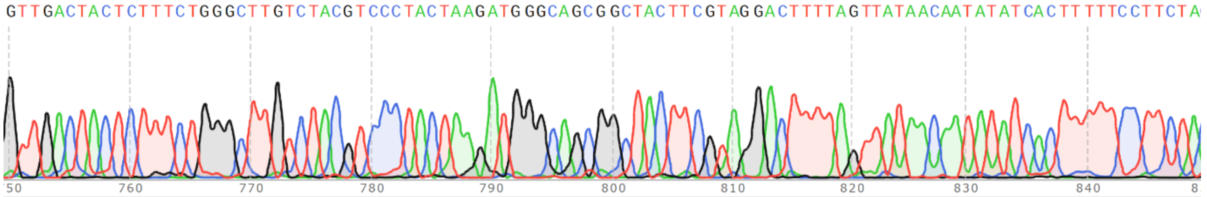


```
PCR_C4 : gttgactactctttctgggcttgtctacgtccctactaagatgggcagcggctacttcgtaggacttttagttataacaatatatcactttttccttcta : 800
r04_C4 : gttgactactctttctgggcttgtctacgtccctactaagatgggcagcggctacttcgtaggacttttagttataacaatatatcactttttccttcta : 796
         GTTGACTACTCTTTCTGGGCTTGTCTACGTCCCTACTAAGATGGGCAGCGGCTACTTCGTAGGACTTTTAGTTATAACAATATATCACTTTTTCCTTCTA
```

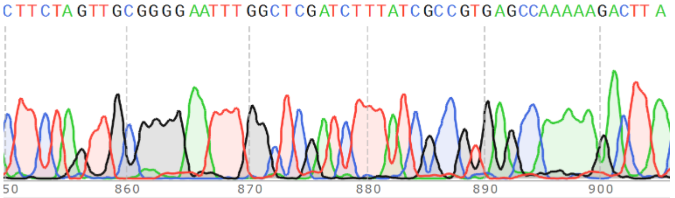


```
PCR_C4 : cttctagttgcggggaatttggctcgatctttatcgccgtgagccaaaaagactta : 856
r04_C4 : cttctagttgcggggaatttggctcgatctttatcgccgtgagccaaaaagactta : 852
         CTTCTAGTTGCGGGGAATTTGGCTCGATCTTTATCGCCGTGAGCCAAAAAGACTTA
```

e

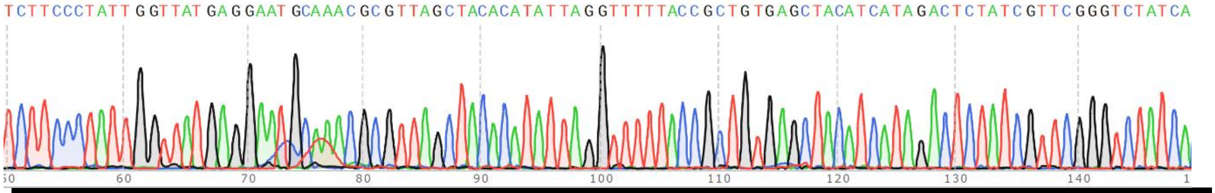


```
PCR_C1 : tcttccctattggttatgaggaatgcaaacgcgttagctacacatattaggtttttaccgctgtgagctacatcatagactctatcgttcgggtctatca : 100
r05_C1 : tcttccctattggttatgaggaatgcaaacgcgttagctacacatattaggtttttaccgctgtgagctacatcatagactctatcgttcgggtctatca : 100
         TCTTCCCTATTGGTTATGAGGAATGCAAACGCGTTAGCTACACATATTAGGTTTTTACCGCTGTGAGCTACATCATAGACTCTATCGTTCGGGTCTATCA
```

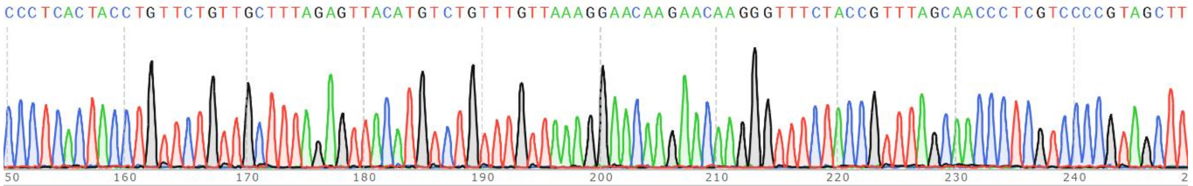

```
PCR_C1 : cccctcactacctgttctgttgctttagagttacatgtctgtttgttaaaggaacaagaacaagggtttctaccgtttagcaaccctcgtccccgtagctt : 200
r05_C1 : cccctcactacctgttctgttgctttagagttacatgtctgtttgttaaaggaacaagaacaagggtttctaccgtttagcaaccctcgtccccgtagctt : 200
         CCCCTCACTACCTGTTCTGTTGCTTTAGAGTTACATGTCTGTTTGTTAAAGGAACAAGAACAAGGGTTTCTACCGTTTAGCAACCCTCGTCCCCGTAGCTT
```

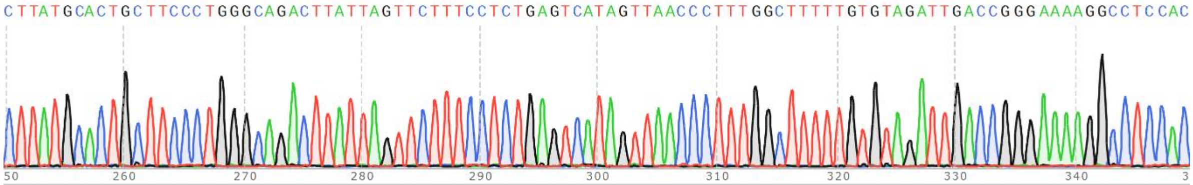

```
PCR_C1 : cttatgcactgcttccctgggcagacttattagttctttcctctgagtcatagttaaccctttggctttttgtgtagattgaccgggaaaaggcctccac : 300
r05_C1 : cttatgcactgcttccctgggcagacttattagttctttcctctgagtcatagttaaccctttggctttttgtgtagattgaccgggaaaaggcctccac : 300
         CTTATGCACTGCTTCCCTGGGCAGACTTATTAGTTCTTTCCTCTGAGTCATAGTTAACCCTTTGGCTTTTTGTGTAGATTGACCGGGAAAAGGCCTCCAC
```

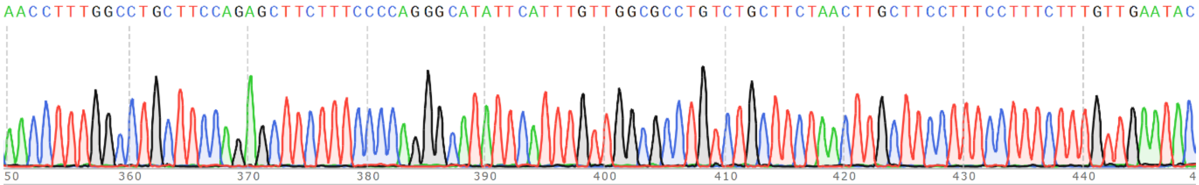

```
PCR_C1 : aacctttggcctgcttccagagcttctttccccagggcatattcatttgttggcgcctgtctgcttctaacttgcttcctttcctttctttgttgaatac : 400
r05_C1 : aacctttggcctgcttccagagcttctttccccagggcatattcatttgttggcgcctgtctgcttctaacttgcttcctttcctttctttgttgaatac : 400
         AACCTTTGGCCTGCTTCCAGAGCTTCTTTCCCCAGGGCATATTCATTTGTTGGCGCCTGTCTGCTTCTAACTTGCTTCCTTTCCTTTCTTTGTTGAATAC
```

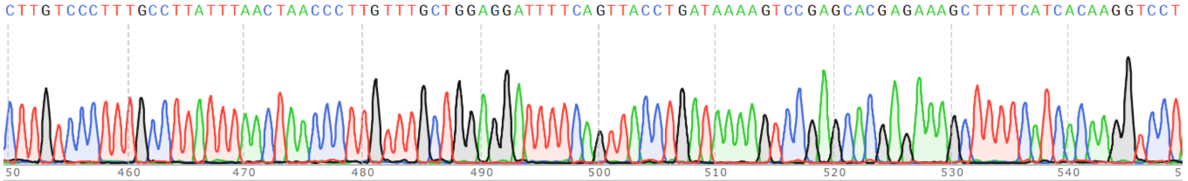

```
PCR_C1 : cttgtccctttgccttatttaactaaccccttgtttgctggaggattttcagttacctgataaaagtccgagcacgagaaagcttttcatcacaaggtcct : 500
r05_C1 : cttgtccctttgccttatttaactaaccccttgtttgctggaggattttcagttacctgataaaagtccgagcacgagaaagcttttcatcacaaggtcct : 500
         CTTGTCCCTTTGCCTTATTTAACTAACCCCTTGTTTGCTGGAGGATTTTCAGTTACCTGATAAAAGTCCGAGCACGAGAAAGCTTTTCATCACAAGGTCCT
```

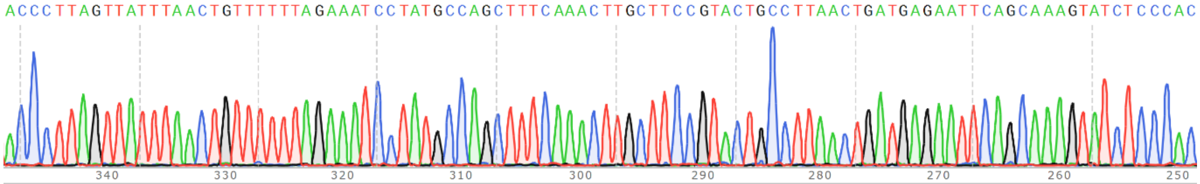

```
PCR_C1 : acccttagttatttaactgttttttagaaatcctatgccagctttcaaacttgcttccgtactgccttaactgatgagaattcagcaaagtatctcccac : 600
r05_C1 : acccttagttatttaactgttttttagaaatcctatgccagctttcaaacttgcttccgtactgccttaactgatgagaattcagcaaagtatctcccac : 600
         ACCCTTAGTTATTTAACTGTTTTTTAGAAATCCTATGCCAGCTTTCAAACTTGCTTCCGTACTGCCTTAACTGATGAGAATTCAGCAAAGTATCTCCCAC
```

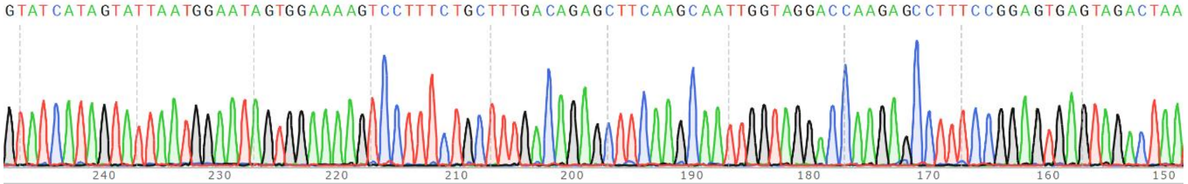

```
PCR_C1 : gtatcatagtattaatggaatagtggaaaagtcctttctgctttgacagagcttcaagcaattggtaggaccaagagcctttccggagtgagtagactaa : 700
r05_C1 : gtatcatagtattaatggaatagtggaaaagtcctttctgctttgacagagcttcaagcaattggtaggaccaagagcctttccggagtgagtagactaa : 700
         GTATCATAGTATTAATGGAATAGTGGAAAAGTCCTTTCTGCTTTGACAGAGCTTCAAGCAATTGGTAGGACCAAGAGCCTTTCCGGAGTGAGTAGACTAA
```

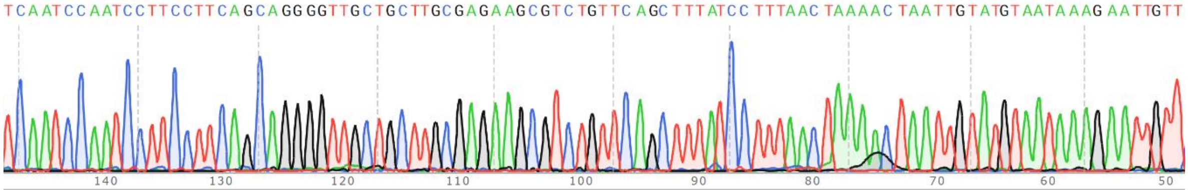

```
PCR_C1 : tcaatccaatccttccttcagcaggggttgctgcttgcgagaagcgtctgttcagctttatcctttaactaaaactaattgtatgtaataaagaattgtt : 800
r05_C1 : tcaatccaatccttccttcagcaggggttgctgcttgcgagaagcgtctgttcagctttatcctttaactaaaactaattgtatgtaataaagaattgtt : 800
         TCAATCCAATCCTTCCTTCAGCAGGGGTTGCTGCTTGCGAGAAGCGTCTGTTCAGCTTTATCCTTTAACTAAAACTAATTGTATGTAATAAAGAATTGTT
```

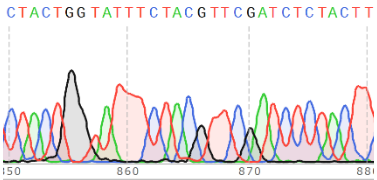

PCR_C1 : ctactggtatttctacgttcgatctctactt : 831
r05_C1 : ctactggtatttctacgttcgatctctactt : 831
CTACTGGTATTTCTACGTTCGATCTCTACTT

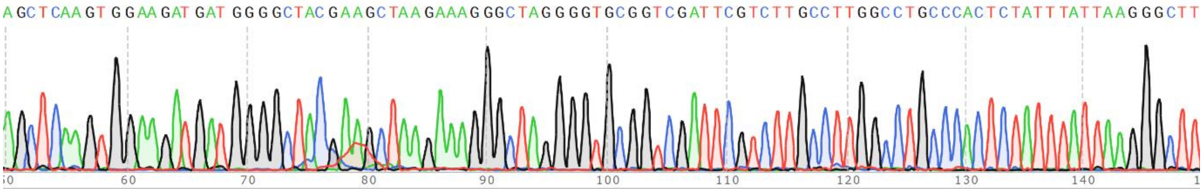

PCR_C2 : agctcaagtggaagatgatggggctacgaagctaagaaagggctaggggtgcggtcgattcgtcttgccttggcctgcccactctatttattaagggctt : 100
r05_C2 : agctcaagtggaagatgatggggctacgaagctaagaaagggctaggggtgcggtcgattcgtcttgccttggcctgcccactctatttattaagggctt : 100
AGCTCAAGTGGAAGATGATGGGGCTACGAAGCTAAGAAAGGGCTAGGGGTGCGGTCGATTCGTCTTGCCTTGGCCTGCCCACTCTATTTATTAAGGGCTT

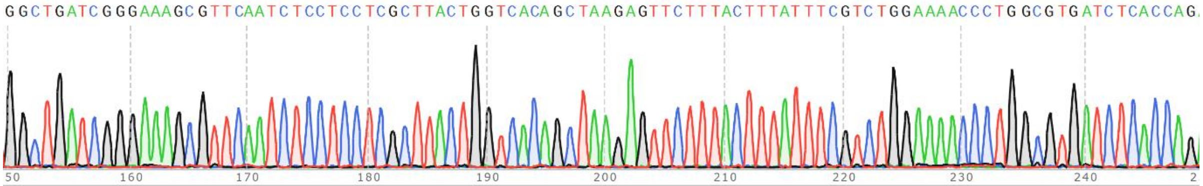

PCR_C2 : ggctgatcgggaaagcgttcaatctcctcctcgcttactggtcacagctaagagttctttactttatttcgtctggaaaaccctggcgtgatctcaccag : 200
r05_C2 : ggctgatcgggaaagcgttcaatctcctcctcgcttactggtcacagctaagagttctttactttatttcgtctggaaaaccctggcgtgatctcaccag : 200
GGCTGATCGGGAAAGCGTTCAATCTCCTCCTCGCTTACTGGTCACAGCTAAGAGTTCTTTACTTTATTTCGTCTGGAAAACCCTGGCGTGATCTCACCAG

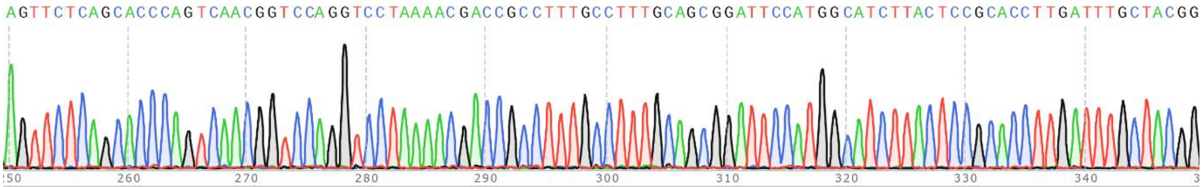

PCR_C2 : agttctcagcacccagtcaacggtccaggtcctaaaacgaccgcctttgcctttgcagcggattccatggcatcttactccgcaccttgatttgctacgg : 300
r05_C2 : agttctcagcacccagtcaacggtccaggtcctaaaacgaccgcctttgcctttgcagcggattccatggcatcttactccgcaccttgatttgctacgg : 300
AGTTCTCAGCACCCAGTCAACGGTCCAGGTCCTAAAACGACCGCCTTTGCCTTTGCAGCGGATTCCATGGCATCTTACTCCGCACCTTGATTTGCTACGG

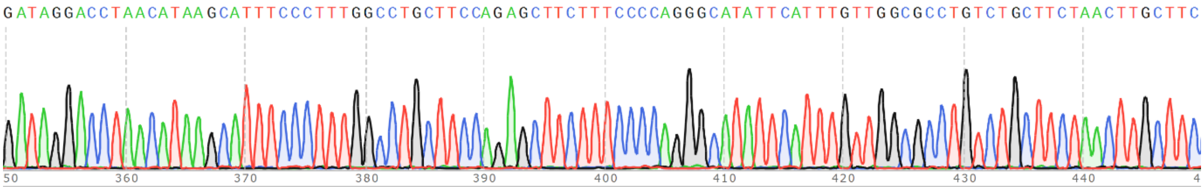

PCR_C2 : gataggacctaacataagcatttccctttggcctgcttccagagcttctttccccagggcatattcatttgttggcgcctgtctgcttctaacttgcttc : 400
r05_C2 : gataggacctaacataagcatttccctttggcctgcttccagagcttctttccccagggcatattcatttgttggcgcctgtctgcttctaacttgcttc : 400
GATAGGACCTAACATAAGCATTTCCCTTTGGCCTGCTTCCAGAGCTTCTTTCCCCAGGGCATATTCATTTGTTGGCGCCTGTCTGCTTCTAACTTGCTTC

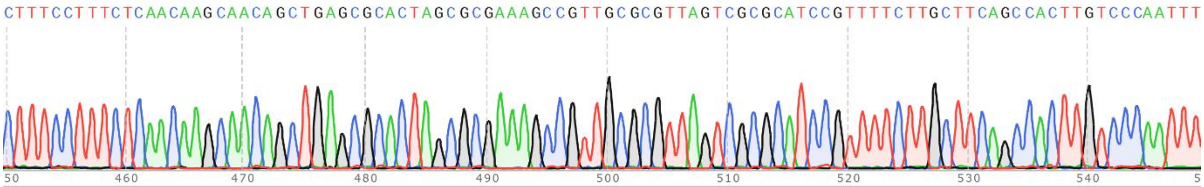

PCR_C2 : ctttcctttctcaacaagcaacagctgagcgcactagcgcgaaagccgttgcgcgttagtcgcgcatccgttttcttgcttcagccacttgtcccaattt : 500
r05_C2 : ctttcctttctcaacaagcaacagctgagcgcactagcgcgaaagccgttgcgcgttagtcgcgcatccgttttcttgcttcagccacttgtcccaattt : 500
CTTTCCTTTCTCAACAAGCAACAGCTGAGCGCACTAGCGCGAAAGCCGTTGCGCGTTAGTCGCGCATCCGTTTTCTTGCTTCAGCCACTTGTCCCAATTT

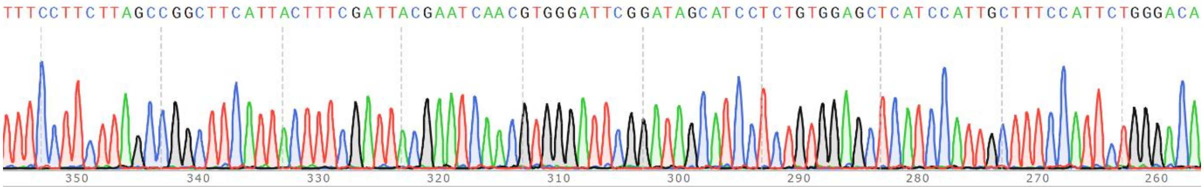

PCR_C2 : tttccttcttagccggcttcattactttcgattacgaatcaacgtgggattcggatagcatcctctgtggagctcatccattgctttccattctgggaca : 600
r05_C2 : tttccttcttagccggcttcattactttcgattacgaatcaacgtgggattcggatagcatcctctgtggagctcatccattgctttccattctgggaca : 600
TTTCCTTCTTAGCCGGCTTCATTACTTTCGATTACGAATCAACGTGGGATTCGGATAGCATCCTCTGTGGAGCTCATCCATTGCTTTCCATTCTGGGACA

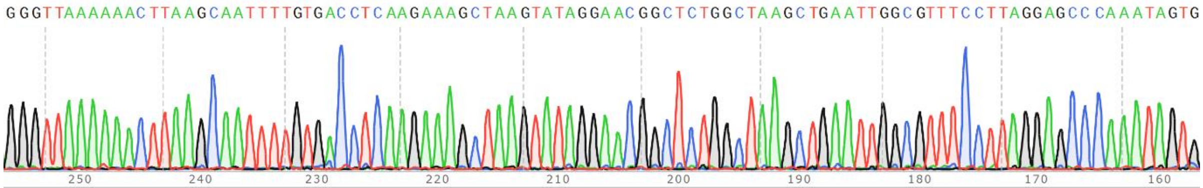

```
PCR_C2 : gggttaaaaaacttaagcaattttgtgacctcaagaaagctaagtataggaacggctctggctaagctgaattggcgtttccttaggagcccaaatagtg : 700
r05_C2 : gggttaaaaaacttaagcaattttgtgacctcaagaaagctaagtataggaacggctctggctaagctgaattggcgtttccttaggagcccaaatagtg : 700
         GGGTTAAAAAACTTAAGCAATTTTGTGACCTCAAGAAAGCTAAGTATAGGAACGGCTCTGGCTAAGCTGAATTGGCGTTTCCTTAGGAGCCCAAATAGTG
```

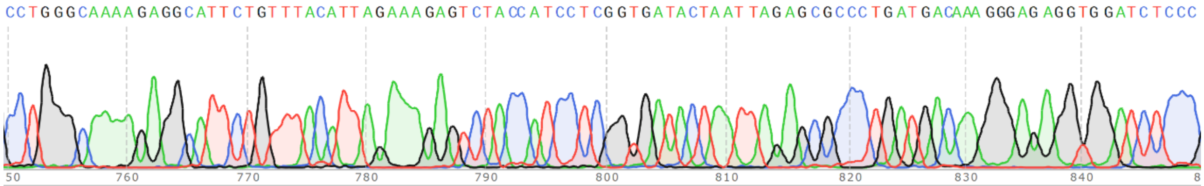

```
PCR_C2 : cctgggcaaaagaggcattctgtttacattagaaagagtctaccatcctcggtgatactaattagagcgccctgatgacaaagggagaggtggatctccc : 800
r05_C2 : cctgggcaaaagaggcattctgtttacattagaaagagtctaccatcctcggtgatactaattagagcgccctgatgac-aagggagaggtggatctccc : 799
         CCTGGGCAAAAGAGGCATTCTGTTTACATTAGAAAGAGTCTACCATCCTCGGTGATACTAATTAGAGCGCCCTGATGAC AAGGGAGAGGTGGATCTCCC
```

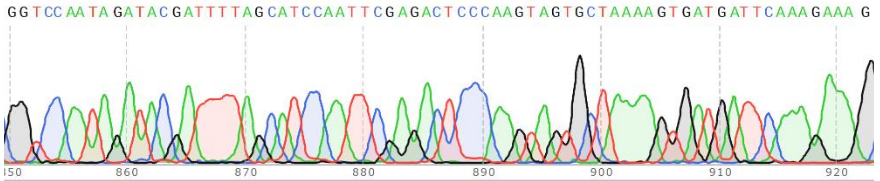

```
PCR_C2 : ggtccaatagatacgattttagcatccaattcgagactcccaagtagtgctaaaagtgatgattcaaagaaag : 873
r05_C2 : ggtccaatagatacgattttagcatccaattcgagactcccaagtagtgctaaaagtgatgattcaaagaaag : 872
         GGTCCAATAGATACGATTTTAGCATCCAATTCGAGACTCCCAAGTAGTGCTAAAAGTGATGATTCAAAGAAAG
```

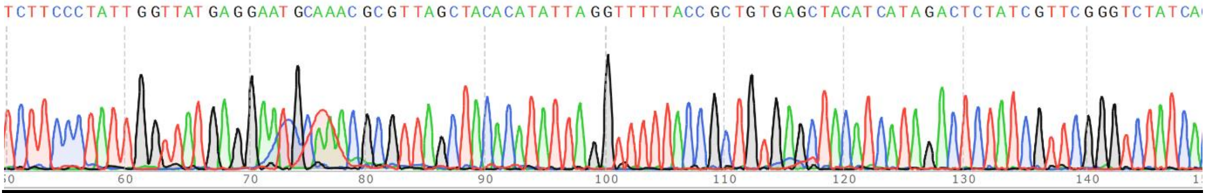

```
PCR_C3 : tcttccctattggttatgaggaatgcaaacgcgttagctacacatattaggtttttaccgctgtgagctacatcatagactctatcgttcgggtctatca : 100
r05_C3 : tcttccctattggttatgaggaatgcaaacgcgttagctacacatattaggtttttaccgctgtgagctacatcatagactctatcgttcgggtctatca : 100
         TCTTCCCTATTGGTTATGAGGAATGCAAACGCGTTAGCTACACATATTAGGTTTTTACCGCTGTGAGCTACATCATAGACTCTATCGTTCGGGTCTATCA
```

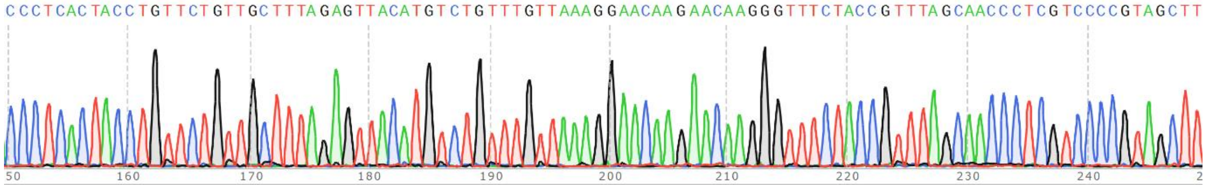

```
PCR_C3 : ccctcactacctgttctgttgctttagagttacatgtctgtttgttaaaggaacaagaacaagggtttctaccgtttagcaaccctcgtccccgtagctt : 200
r05_C3 : ccctcactacctgttctgttgctttagagttacatgtctgtttgttaaaggaacaagaacaagggtttctaccgtttagcaaccctcgtccccgtagctt : 200
         CCCTCACTACCTGTTCTGTTGCTTTAGAGTTACATGTCTGTTTGTTAAAGGAACAAGAACAAGGGTTTCTACCGTTTAGCAACCCTCGTCCCCGTAGCTT
```

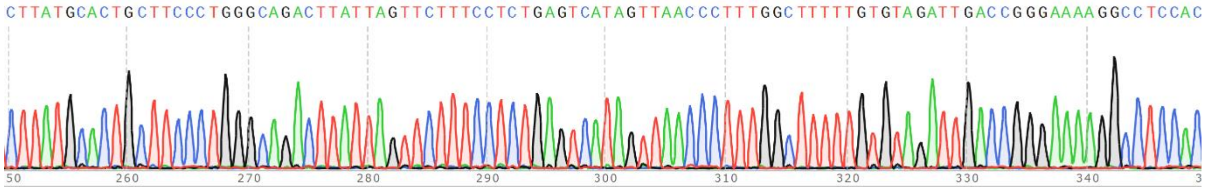

```
PCR_C3 : cttatgcactgcttccctgggcagacttattagttctttcctctgagtcatagttaaccctttggctttttgtgtagattgaccgggaaaaggcctccac : 300
r05_C3 : cttatgcactgcttccctgggcagacttattagttctttcctctgagtcatagttaaccctttggctttttgtgtagattgaccgggaaaaggcctccac : 300
         CTTATGCACTGCTTCCCTGGGCAGACTTATTAGTTCTTTCCTCTGAGTCATAGTTAACCCTTTGGCTTTTTGTGTAGATTGACCGGGAAAAGGCCTCCAC
```

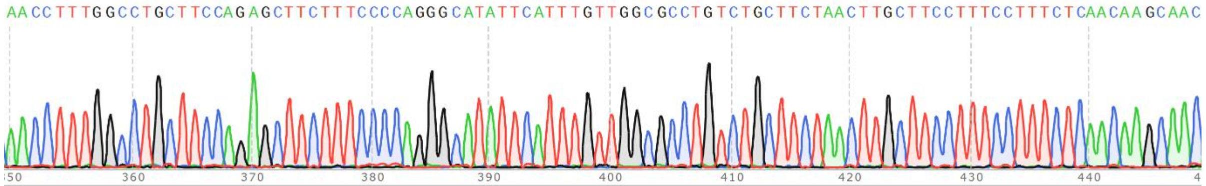

```
PCR_C3 : aacctttggcctgcttccagagcttctttccccagggcatattcatttgttggcgcctgtctgcttctaacttgcttcctttcctttctcaacaagcaac : 400
r05_C3 : aacctttggcctgcttccagagcttctttccccagggcatattcatttgttggcgcctgtctgcttctaacttgcttcctttcctttctcaacaagcaac : 400
         AACCTTTGGCCTGCTTCCAGAGCTTCTTTCCCCAGGGCATATTCATTTGTTGGCGCCTGTCTGCTTCTAACTTGCTTCCTTTCCTTTCTCAACAAGCAAC
```

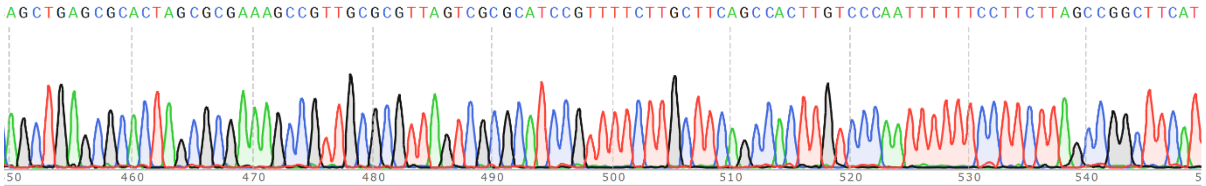

PCR_C3 : agctgagcgcactagcgcgaaagccgttgcgcgttagtcgcgcatccgttttcttgcttcagccacttgtcccaatttttttccttcttagccggcttcat : 500
r05_C3 : agctgagcgcactagcgcgaaagccgttgcgcgttagtcgcgcatccgttttcttgcttcagccacttgtcccaatttttttccttcttagccggcttcat : 500
AGCTGAGCGCACTAGCGCGAAAGCCGTTGCGCGTTAGTCGCGCATCCGTTTTCTTGCTTCAGCCACTTGTCCCAATTTTTTTCCTTCTTAGCCGGCTTCAT

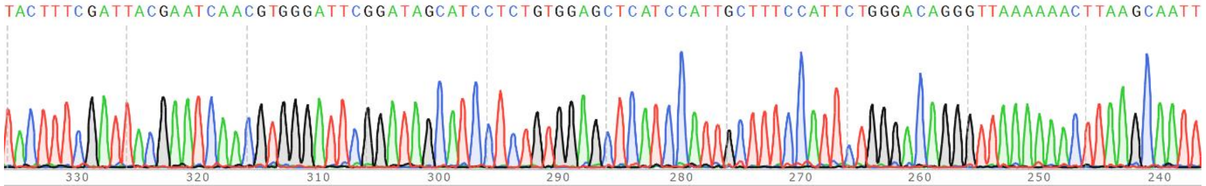

PCR_C3 : tactttcgattacgaatcaacgtgggattcggatagcatcctctgtggagctcatccattgctttccattctgggacagggttaaaaaacttaagcaatt : 600
r05_C3 : tactttcgattacgaatcaacgtgggattcggatagcatcctctgtggagctcatccattgctttccattctgggacagggttaaaaaacttaagcaatt : 600
TACTTTCGATTACGAATCAACGTGGGATTCGGATAGCATCCTCTGTGGAGCTCATCCATTGCTTTCCATTCTGGGACAGGGTTAAAAAACTTAAGCAATT

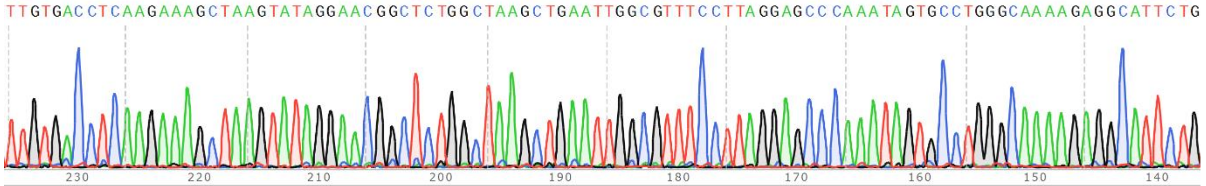

PCR_C3 : ttgtgacctcaagaaagctaagtataggaacggctctggctaagctgaattggcgtttccttaggagcccaaatagtgcctgggcaaaagaggcattctg : 700
r05_C3 : ttgtgacctcaagaaagctaagtataggaacggctctggctaagctgaattggcgtttccttaggagcccaaatagtgcctgggcaaaagaggcattctg : 700
TTGTGACCTCAAGAAAGCTAAGTATAGGAACGGCTCTGGCTAAGCTGAATTGGCGTTTCCTTAGGAGCCCAAATAGTGCCTGGGCAAAAGAGGCATTCTG

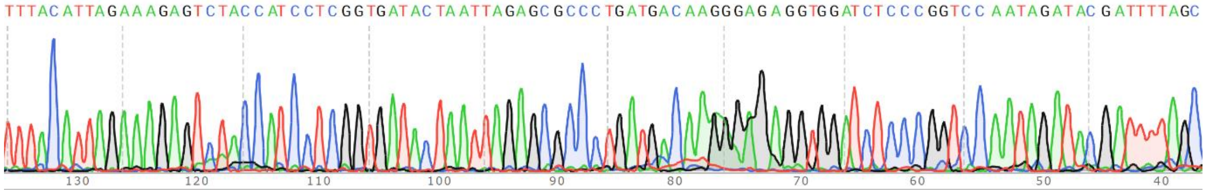

PCR_C3 : tttacattagaaagagtctaccatcctcggtgatactaattagagcgccctgatgacaagggagaggtggatctcccggtccaatagatacgattttagc : 800
r05_C3 : tttacattagaaagagtctaccatcctcggtgatactaattagagcgccctgatgacaagggagaggtggatctcccggtccaatagatacgattttagc : 800
TTTACATTAGAAAGAGTCTACCATCCTCGGTGATACTAATTAGAGCGCCCTGATGACAAGGGAGAGGTGGATCTCCCGGTCCAATAGATACGATTTTAGC

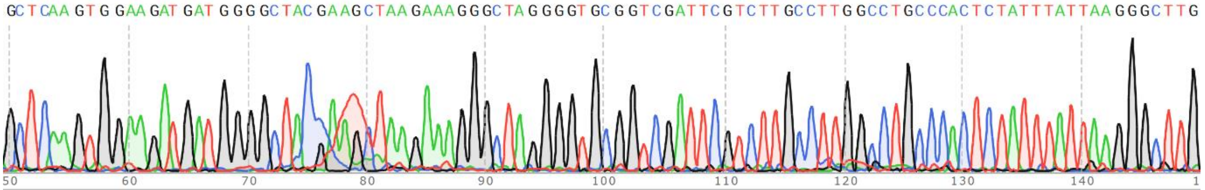

PCR_C4 : gctcaagtggaagatgatggggctacgaagctaagaaagggctaggggtgcggtcgattcgtcttgccttggcctgcccactctatttattaagggcttg : 100
r05_C4 : gctcaagtggaagatgatggggctacgaagctaagaaagggctaggggtgcggtcgattcgtcttgccttggcctgcccactctatttattaagggcttg : 100
GCTCAAGTGGAAGATGATGGGGCTACGAAGCTAAGAAAGGGCTAGGGGTGCGGTCGATTCGTCTTGCCTTGGCCTGCCCACTCTATTTATTAAGGGCTTG

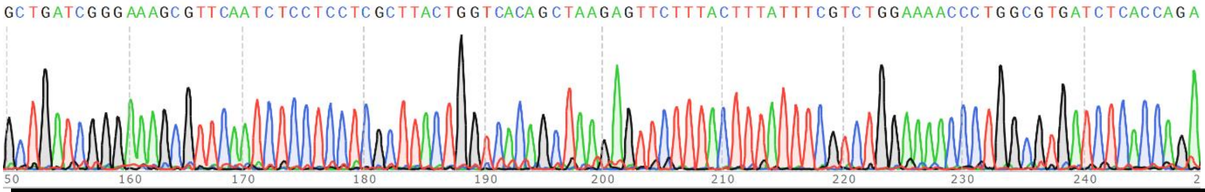

PCR_C4 : gctgatcgggaaagcgttcaatctcctcctcgcttactggtcacagctaagagttctttactttatttcgtctggaaaaccctggcgtgatctcaccaga : 200
r05_C4 : gctgatcgggaaagcgttcaatctcctcctcgcttactggtcacagctaagagttctttactttatttcgtctggaaaaccctggcgtgatctcaccaga : 200
GCTGATCGGGAAAGCGTTCAATCTCCTCCTCGCTTACTGGTCACAGCTAAGAGTTCTTTACTTTATTTCGTCTGGAAAACCCTGGCGTGATCTCACCAGA

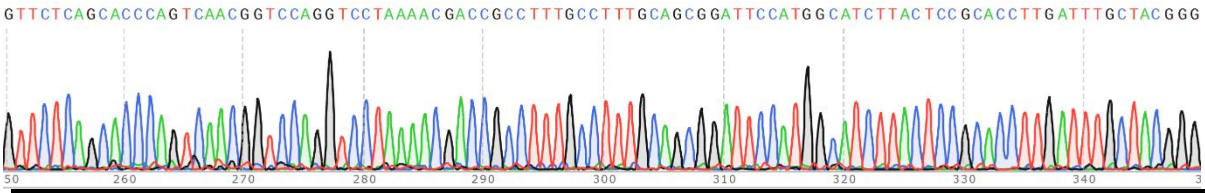

PCR_C4 : gttctcagcacccagtcaacggtccaggtcctaaaacgaccgcctttgcctttgcagcggattccatggcatcttactccgcaccttgatttgctacggg : 300

r05_C4 : gttctcagcacccagtcaacggtccaggtcctaaaacgaccgcctttgcctttgcagcggattccatggcatcttactccgcaccttgatttgctacggg : 300
GTTCTCAGCACCCAGTCAACGGTCCAGGTCCTAAAACGACCGCCTTTGCCTTTGCAGCGGATTCCATGGCATCTTACTCCGCACCTTGATTTGCTACGGG

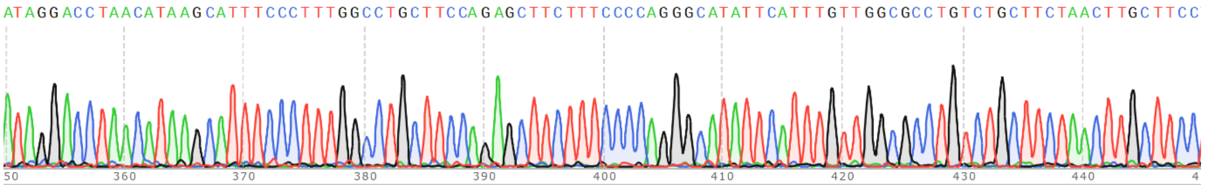

PCR_C4 : ataggacctaacataagcatttccctttggcctgcttccagagcttctttccccagggcatattcatttgttggcgcctgtctgcttctaacttgcttcc : 400
r05_C4 : ataggacctaacataagcatttccctttggcctgcttccagagcttctttccccagggcatattcatttgttggcgcctgtctgcttctaacttgcttcc : 400
ATAGGACCTAACATAAGCATTTCCCTTTGGCCTGCTTCCAGAGCTTCTTTCCCCAGGGCATATTCATTTGTTGGCGCCTGTCTGCTTCTAACTTGCTTCC

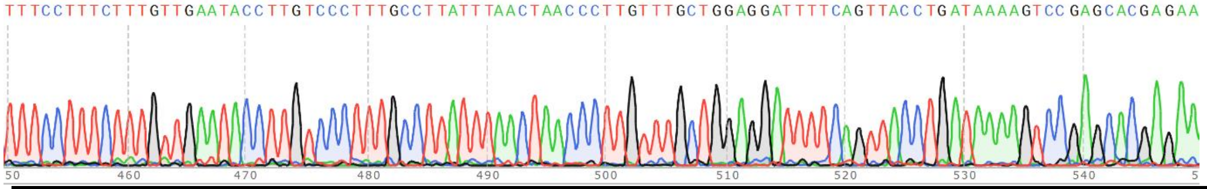

PCR_C4 : tttcctttctttgttgaataccttgtccctttgccttatttaactaacccttgtttgctggaggattttcagttacctgataaaagtccgagcacgagaa : 500
r05_C4 : ttcctttctttgttgaataccttgtccctttgccttatttaactaacccttgtttgctggaggattttcagttacctgataaaagtccgagcacgagaa : 500
TTTCCTTTCTTTGTTGAATACCTTGTCCCTTTGCCTTATTTAACTAACCCTTGTTTGCTGGAGGATTTTCAGTTACCTGATAAAAGTCCGAGCACGAGAA

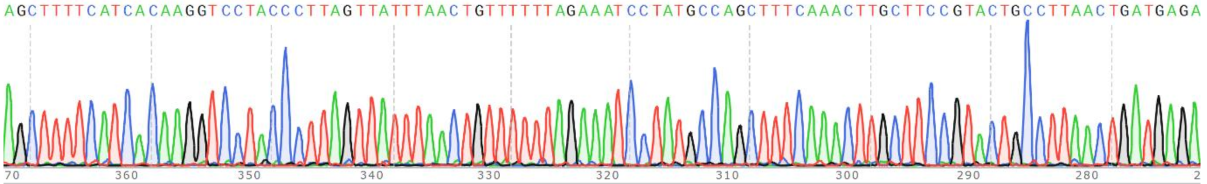

PCR_C4 : agcttttcatcacaaggtcctacccttagttatttaactgttttttagaaatcctatgccagctttcaaacttgcttccgtactgccttaactgatgaga : 600
r05_C4 : agcttttcatcacaaggtcctacccttagttatttaactgttttttagaaatcctatgccagctttcaaacttgcttccgtactgccttaactgatgaga : 600
AGCTTTTCATCACAAGGTCCTACCCTTAGTTATTTAACTGTTTTTTAGAAATCCTATGCCAGCTTTCAAACTTGCTTCCGTACTGCCTTAACTGATGAGA

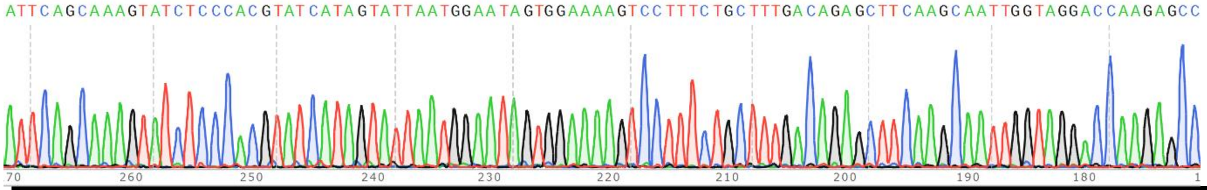

PCR_C4 : attcagcaaagtatctcccacgtatcatagtattaatggaatagtggaaaagtcctttctgctttgacagagcttcaagcaattggtaggaccaagagcc : 700
r05_C4 : attcagcaaagtatctcccacgtatcatagtattaatggaatagtggaaaagtcctttctgctttgacagagcttcaagcaattggtaggaccaagagcc : 700
ATTCAGCAAAGTATCTCCCACGTATCATAGTATTAATGGAATAGTGGAAAAGTCCTTTCTGCTTTGACAGAGCTTCAAGCAATTGGTAGGACCAAGAGCC

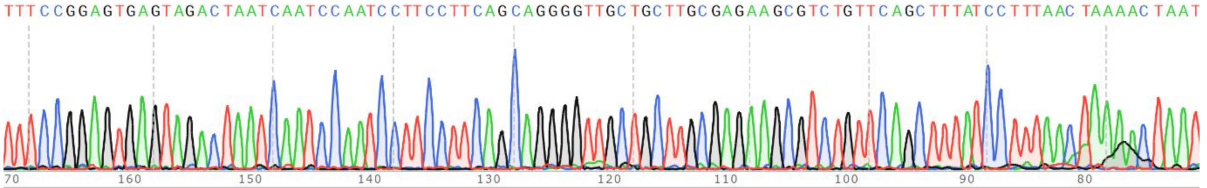

PCR_C4 : tttccggagtgagtagactaatcaatccaatccttccttcagcaggggttgctgcttgcgagaagcgtctgttcagctttatcctttaactaaaactaat : 800
r05_C4 : tttccggagtgagtagactaatcaatccaatccttccttcagcaggggttgctgcttgcgagaagcgtctgttcagctttatcctttaactaaaactaat : 800
TTTCCGGAGTGAGTAGACTAATCAATCCAATCCTTCCTTCAGCAGGGGTTGCTGCTTGCGAGAAGCGTCTGTTCAGCTTTATCCTTTAACTAAAACTAAT

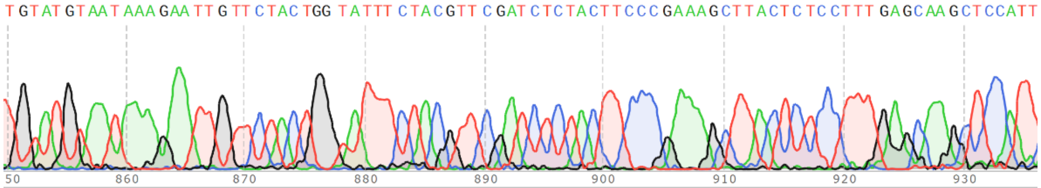

PCR_C4 : tgtatgtaataaagaattgttctactggtatttctacgttcgatctctacttcccgaaagcttactctcctttgagcaagctccatt : 887
r05_C4 : tgtatgtaataaagaattgttctactggtatttctacgttcgatctctacttcccgaaagcttactctcctttgagcaagctccatt : 887
TGTATGTAATAAAGAATTGTTCTACTGGTATTTCTACGTTCGATCTCTACTTCCCGAAAGCTTACTCTCCTTTGAGCAAGCTCCATT

f

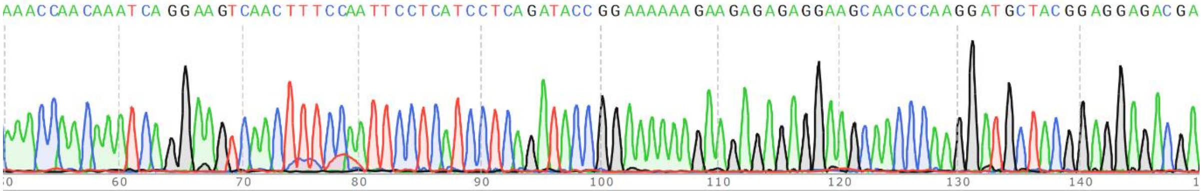

PCR_C1 : aaaccaacaaatcaggaagtcaactttccaattcctcatcctcagataccggaaaaaagaagagagaggaagcaacccaaggatgctacggaggagacga : 100
r06_C1 : aaaccaacaaatcaggaagtcaactttccaattcctcatcctcagataccggaaaaaagaagagagaggaagcaacccaaggatgctacggaggagacga : 100
AAACCAACAAATCAGGAAGTCAACTTTCCAATTCCTCATCCTCAGATACCGGAAAAAAGAAGAGAGAGGAAGCAACCCAAGGATGCTACGGAGGAGACGA

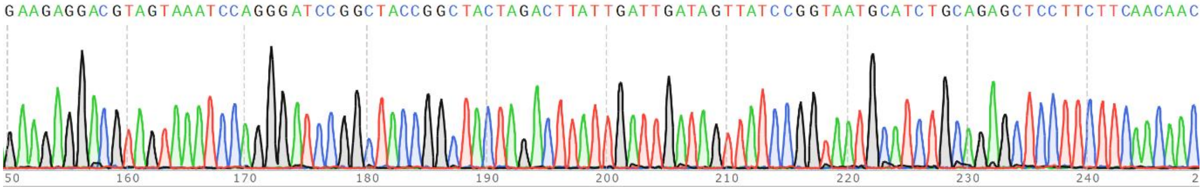

PCR_C1 : gaagaggacgtagtaaatccagggatccggctaccggctactagacttattgattgatagttatccggtaatgcatctgcagagctccttcttcaacaac : 200
r06_C1 : gaagaggacgtagtaaatccagggatccggctaccggctactagacttattgattgatagttatccggtaatgcatctgcagagctccttcttcaacaac : 200
GAAGAGGACGTAGTAAATCCAGGGATCCGGCTACCGGCTACTAGACTTATTGATTGATAGTTATCCGGTAATGCATCTGCAGAGCTCCTTCTTCAACAAC

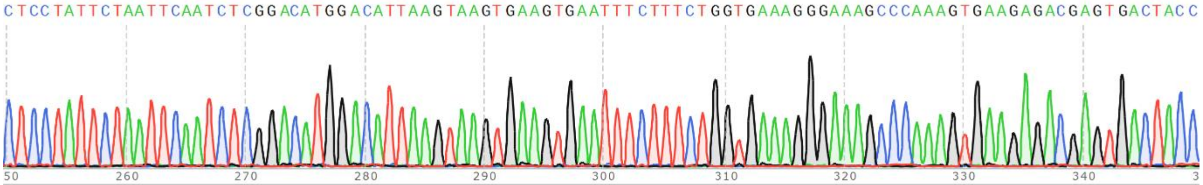

PCR_C1 : ctcctattctaattcaatctcggacatggacattaagtaagtgaagtgaatttctttctggtgaaagggaaagcccaaagtgaagagacgagtgactacc : 300
r06_C1 : ctcctattctaattcaatctcggacatggacattaagtaagtgaagtgaatttctttctggtgaaagggaaagcccaaagtgaagagacgagtgactacc : 300
CTCCTATTCTAATTCAATCTCGGACATGGACATTAAGTAAGTGAAGTGAATTTCTTTCTGGTGAAAGGGAAAGCCCAAAGTGAAGAGACGAGTGACTACC

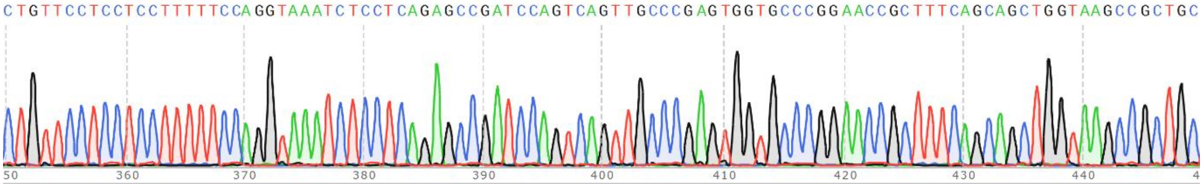

PCR_C1 : ctgttcctcctcctttttccaggtaaatctcctcagagccgatccagtcagttgcccgagtggtgcccggaaccgctttcagcagctggtaagccgctgc : 400
r06_C1 : ctgttcctcctcctttttccaggtaaatctcctcagagccgatccagtcagttgcccgagtggtgcccggaaccgctttcagcagctggtaagccgctgc : 400
CTGTTCCTCCTCCTTTTTCCAGGTAAATCTCCTCAGAGCCGATCCAGTCAGTTGCCCGAGTGGTGCCCGGAACCGCTTTCAGCAGCTGGTAAGCCGCTGC

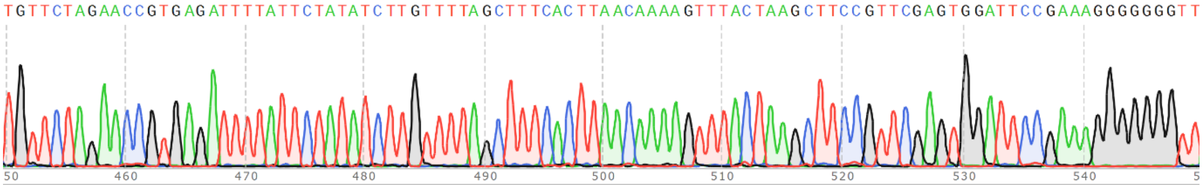

PCR_C1 : tgttctagaaccgtgagattttattctatatcttgttttagctttcacttaacaaaagtttactaagcttccgttcgagtggattccgaaagggggggtt : 500
r06_C1 : tgttctagaaccgtgagattttattctatatcttgttttagctttcacttaacaaaagtttactaagcttccgttcgagtggattccgaaagggggggtt : 500
TGTTCTAGAACCGTGAGATTTTATTCTATATCTTGTTTTAGCTTTCACTTAACAAAAGTTTACTAAGCTTCCGTTCGAGTGGATTCCGAAAGGGGGGGTT

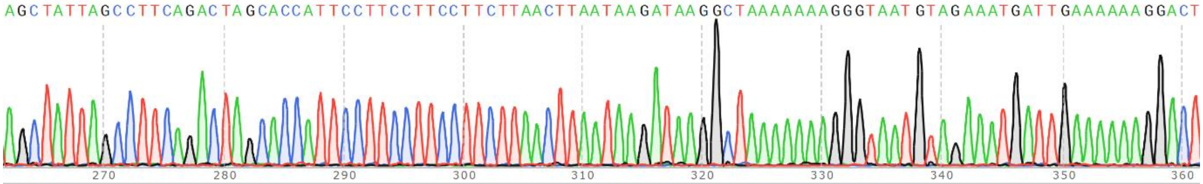

PCR_C1 : agtcctttttttcaatcatttctacattaccctttttttagccttatcttattaagttaagaaggaaggaaggaatggtgctagtctgaaggctaatagct : 600
r06_C1 : agtcctttttttcaatcatttctacattaccctttttttagccttatcttattaagttaagaaggaaggaaggaatggtgctagtctgaaggctaatagct : 600
AGTCCTTTTTTTCAATCATTTCTACATTACCCTTTTTTTAGCCTTATCTTATTAAGTTAAGAAGGAAGGAAGGAATGGTGCTAGTCTGAAGGCTAATAGCT

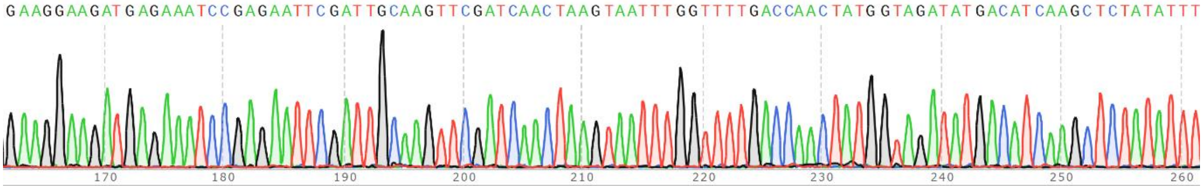

PCR_C1 : aaatatagagcttgatgtcatatctaccatagttggtcaaaaccaaattacttagttgatcgaacttgcaatcgaattctcggatttctcatcttccttc : 700
r06_C1 : aaatatagagcttgatgtcatatctaccatagttggtcaaaaccaaattacttagttgatcgaacttgcaatcgaattctcggatttctcatcttccttc : 700
AAATATAGAGCTTGATGTCATATCTACCATAGTTGGTCAAAACCAAATTACTTAGTTGATCGAACTTGCAATCGAATTCTCGGATTTCTCATCTTCCTTC

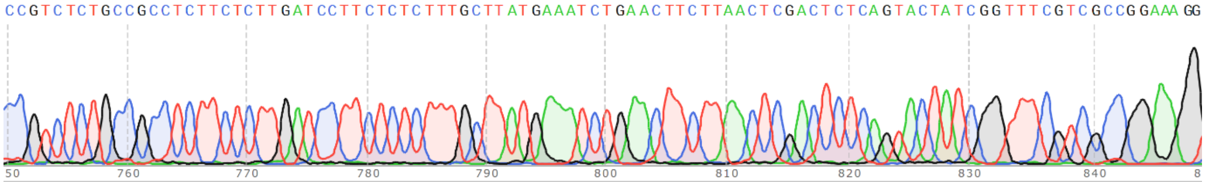


```
PCR_C1 : ccgtctctgccgcctcttctcttgatccttctctctttgcttatgaaatctgaacttcttaactcgactctcagtactatcggtttcgtcgccggaaagg : 800
r06_C1 : ccgtctctgccgcctcttctcttgatccttctctctttgcttatgaaatctgaacttcttaactcgactctcagtactatcggtttcgtcgccgg-aagg : 799
         CCGTCTCTGCCGCCTCTTCTCTTGATCCTTCTCTCTTTGCTTATGAAATCTGAACTTCTTAACTCGACTCTCAGTACTATCGGTTTCGTCGCCGG AAGG
```

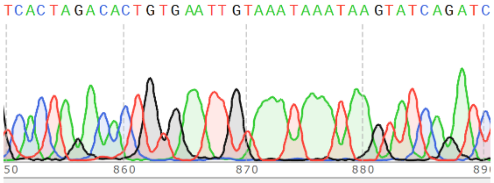


```
PCR_C1 : tcactagacactgtgaattgtaaataaataagtatcagatc : 841
r06_C1 : tcactagacactgtgaattgtaaataaataagtatcagatc : 840
         TCACTAGACACTGTGAATTGTAAATAAATAAGTATCAGATC
```

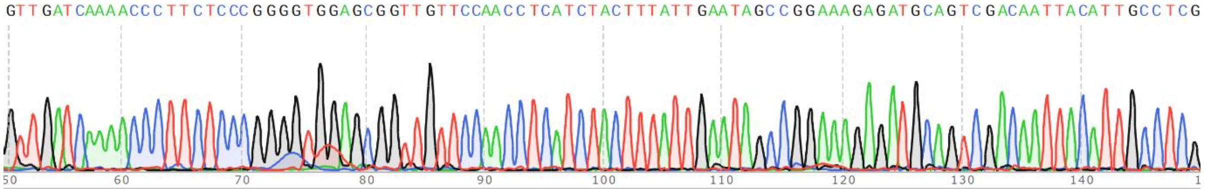


```
PCR_C2 : gttgatcaaaacccttctcccggggtggagcggttgttccaacctcatctactttattgaatagccggaaagagatgcagtcgacaattacattgcctcg : 100
r06_C2 : gttgatcaaaacccttctcccggggtggagcggttgttccaacctcatctactttattgaatagccggaaagagatgcagtcgacaattacattgcctcg : 100
         GTTGATCAAAACCCTTCTCCCGGGGTGGAGCGGTTGTTCCAACCTCATCTACTTTATTGAATAGCCGGAAAGAGATGCAGTCGACAATTACATTGCCTCG
```

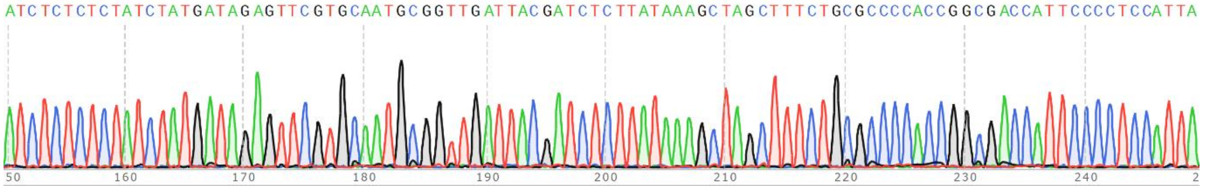


```
PCR_C2 : atctctctctatctatgatagagttcgtgcaatgcggttgattacgatctcttataaagctagctttctgcgccccaccggcgaccattcccctccatta : 200
r06_C2 : atctctctctatctatgatagagttcgtgcaatgcggttgattacgatctcttataaagctagctttctgcgccccaccggcgaccattcccctccatta : 200
         ATCTCTCTCTATCTATGATAGAGTTCGTGCAATGCGGTTGATTACGATCTCTTATAAAGCTAGCTTTCTGCGCCCCACCGGCGACCATTCCCCTCCATTA
```

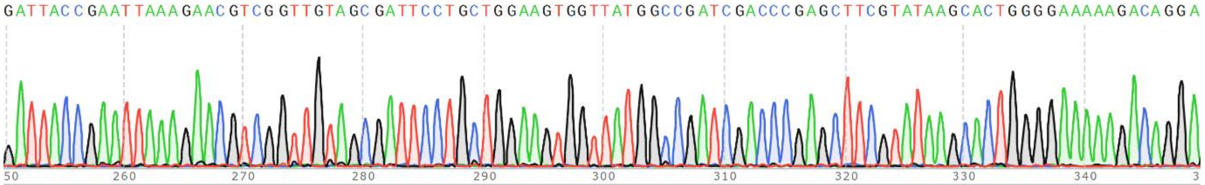


```
PCR_C2 : gattaccgaattaaagaacgtcggttgtagcgattcctgctggaagtggttatggccgatcgacccgagcttcgtataagcactggggaaaaagacagga : 300
r06_C2 : gattaccgaattaaagaacgtcggttgtagcgattcctgctggaagtggttatggccgatcgacccgagcttcgtataagcactggggaaaaagacagga : 300
         GATTACCGAATTAAAGAACGTCGGTTGTAGCGATTCCTGCTGGAAGTGGTTATGGCCGATCGACCCGAGCTTCGTATAAGCACTGGGGAAAAAGACAGGA
```

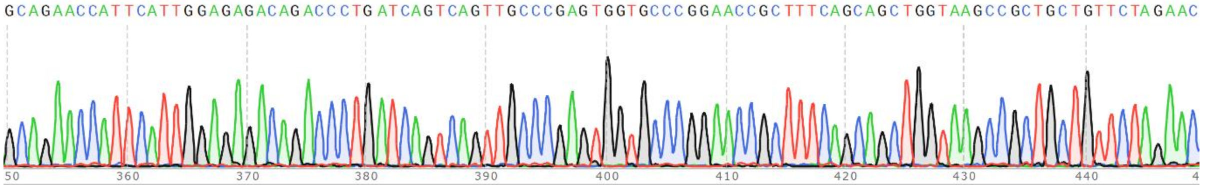


```
PCR_C2 : gcagaaccattcattggagagacagaccctgatcagtcagttgcccgagtggtgcccggaaccgctttcagcagctggtaagccgctgctgttctagaac : 400
r06_C2 : gcagaaccattcattggagagacagaccctgatcagtcagttgcccgagtggtgcccggaaccgctttcagcagctggtaagccgctgctgttctagaac : 400
         GCAGAACCATTCATTGGAGAGACAGACCCTGATCAGTCAGTTGCCCGAGTGGTGCCCGGAACCGCTTTCAGCAGCTGGTAAGCCGCTGCTGTTCTAGAAC
```

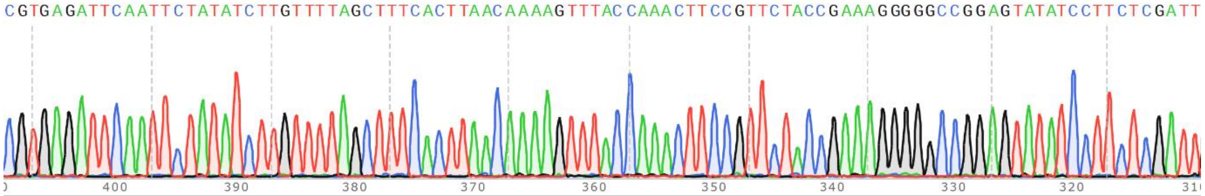


```
PCR_C2 : cgtgagattcaattctatatcttgttttagctttcacttaacaaaagtttaccaaacttccgttctaccgaaagggggccggagtatatccttctcgatt : 500
r06_C2 : cgtgagattcaattctatatcttgttttagctttcacttaacaaaagtttaccaaacttccgttctaccgaaagggggccggagtatatccttctcgatt : 500
         CGTGAGATTCAATTCTATATCTTGTTTTAGCTTTCACTTAACAAAAGTTTACCAAACTTCCGTTCTACCGAAAGGGGGCCGGAGTATATCCTTCTCGATT
```

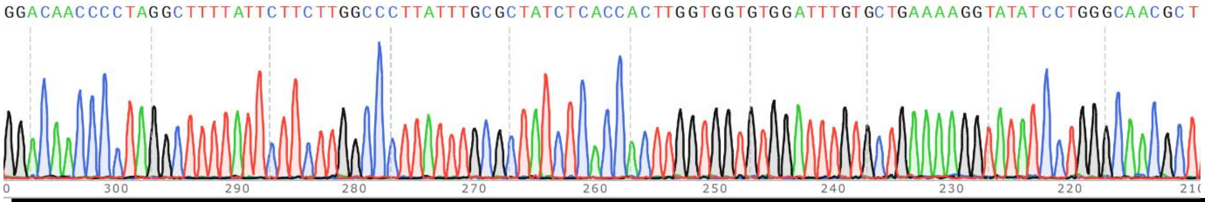


```
PCR_C2 : ggacaacccctaggcttttattcttcttggcccttatttgcgctatctcaccacttggtggtgtggatttgtgctgaaaaggtatatcctgggcaacgct : 600
r06_C2 : ggacaacccctaggcttttattcttcttggcccttatttgcgctatctcaccacttggtggtgtggatttgtgctgaaaaggtatatcctgggcaacgct : 600
         GGACAACCCCTAGGCTTTTATTCTTCTTGGCCCTTATTTGCGCTATCTCACCACTTGGTGGTGTGGATTTGTGCTGAAAAGGTATATCCTGGGCAACGCT
```

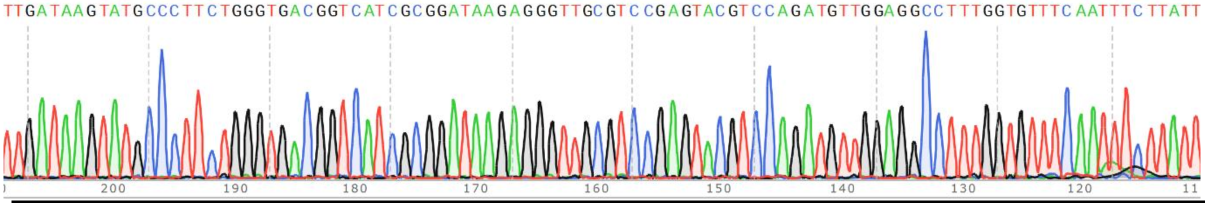


```
PCR_C2 : ttgataagtatgcccttctgggtgacggtcatcgcggataagagggttgcgtccgagtacgtccagatgttggaggcctttggtgtttcaatttcttatt : 700
r06_C2 : ttgataagtatgcccttctgggtgacggtcatcgcggataagagggttgcgtccgagtacgtccagatgttggaggcctttggtgtttcaatttcttatt : 700
         TTGATAAGTATGCCCTTCTGGGTGACGGTCATCGCGGATAAGAGGGTTGCGTCCGAGTACGTCCAGATGTTGGAGGCCTTTGGTGTTTCAATTTCTTATT
```

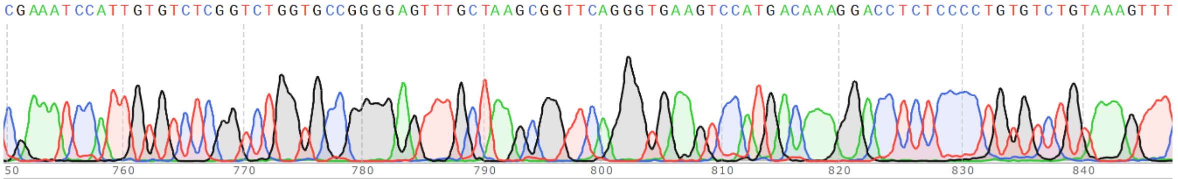


```
PCR_C2 : cgaaatccattgtgtctcggtctggtgccggggagtttgctaagcggttcagggtgaagtccatgacaaaggacctctcccctgtgtctgtaaagttt : 798
r06_C2 : cgaaatccattgtgtctcggtctggtgccggggagtttgctaagcggttcagggtgaagtccatgacaaaggacctctcccctgtgtctgtaaagttt : 798
         CGAAATCCATTGTGTCTCGGTCTGGTGCCGGGGAGTTTGCTAAGCGGTTCAGGGTGAAGTCCATGACAAAGGACCTCTCCCCTGTGTCTGTAAAGTTT
```

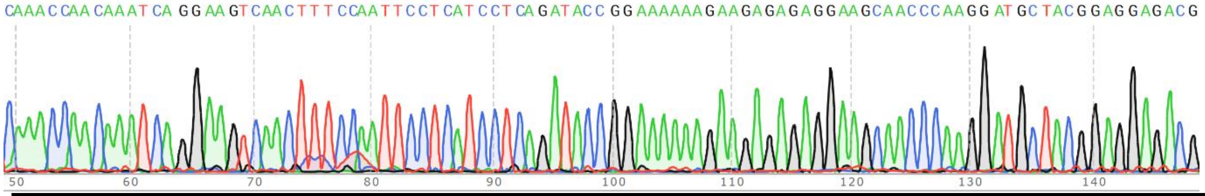


```
PCR_C3 : caaaccaacaaatcaggaagtcaactttccaattcctcatcctcagataccggaaaaaaagaagagagaggaagcaacccaaggatgctacggaggagacg : 100
r06_C3 : caaaccaacaaatcaggaagtcaactttccaattcctcatcctcagataccggaaaaaaagaagagagaggaagcaacccaaggatgctacggaggagacg : 100
         CAAACCAACAAATCAGGAAGTCAACTTTCCAATTCCTCATCCTCAGATACCGGAAAAAAAGAAGAGAGAGGAAGCAACCCAAGGATGCTACGGAGGAGACG
```

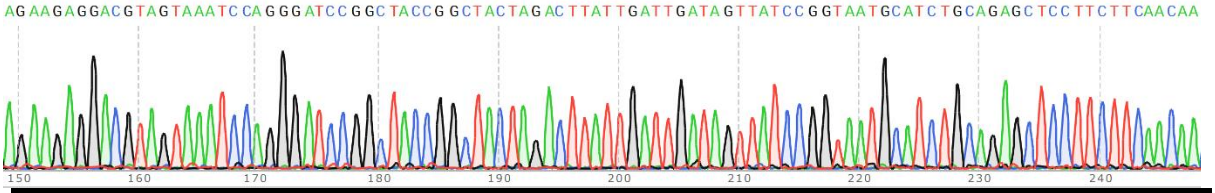


```
PCR_C3 : agaagaggacgtagtaaatccagggatccggctaccggctactagacttattgattgatagttatccggtaatgcatctgcagagctccttcttcaacaa : 200
r06_C3 : agaagaggacgtagtaaatccagggatccggctaccggctactagacttattgattgatagttatccggtaatgcatctgcagagctccttcttcaacaa : 200
         AGAAGAGGACGTAGTAAATCCAGGGATCCGGCTACCGGCTACTAGACTTATTGATTGATAGTTATCCGGTAATGCATCTGCAGAGCTCCTTCTTCAACAA
```

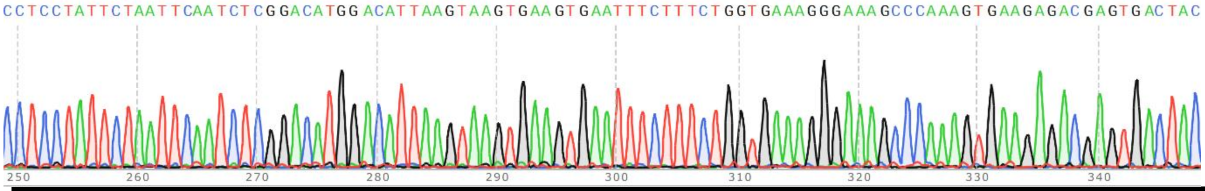


```
PCR_C3 : cctcctattctaattcaatctcggacatggacattaagtaagtgaagtgaatttctttctggtgaaagggaaagcccaaagtgaagagacgagtgactac : 300
r06_C3 : cctcctattctaattcaatctcggacatggacattaagtaagtgaagtgaatttctttctggtgaaagggaaagcccaaagtgaagagacgagtgactac : 300
         CCTCCTATTCTAATTCAATCTCGGACATGGACATTAAGTAAGTGAAGTGAATTTCTTTCTGGTGAAAGGGAAAGCCCAAAGTGAAGAGACGAGTGACTAC
```

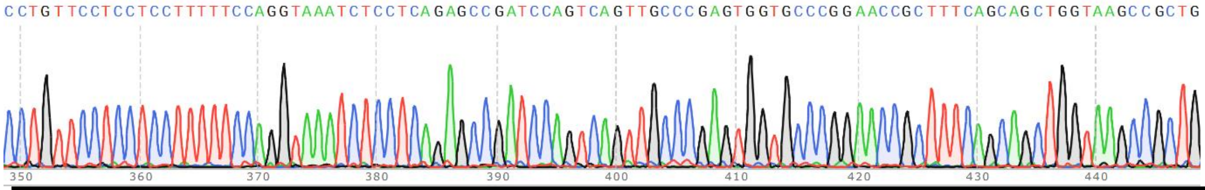


```
PCR_C3 : cctgttcctcctcctttttccaggtaaatctcctcagagccgatccagtcagttgcccgagtggtgcccggaaccgctttcagcagctggtaagccgctg : 400
r06_C3 : cctgttcctcctcctttttccaggtaaatctcctcagagccgatccagtcagttgcccgagtggtgcccggaaccgctttcagcagctggtaagccgctg : 400
         CCTGTTCCTCCTCCTTTTTCCAGGTAAATCTCCTCAGAGCCGATCCAGTCAGTTGCCCGAGTGGTGCCCGGAACCGCTTTCAGCAGCTGGTAAGCCGCTG
```

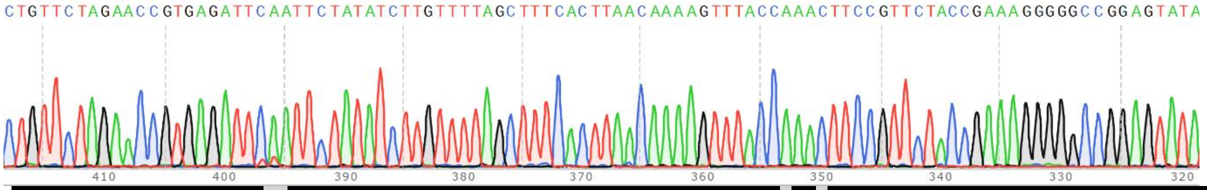


```
PCR_C3 : ctgttctagaaccgtgagattcaattctatatcttgttttagctttcacttaacaaaagtttaccaaacttccgttctaccgaaagggggccggagtata : 500
r06_C3 : ctgttctagaaccgtgagatttt attctatatcttgttttagctttcacttaacaaaagtttactaagcttccgttctaccgaaagggggccggagtata : 500
         CTGTTCTAGAACCGTGAGATT  ATTCTATATCTTGTTTTAGCTTTCACTTAACAAAAGTTTAC AA CTTCCGTTCTACCGAAAGGGGGCCGGAGTATA
```

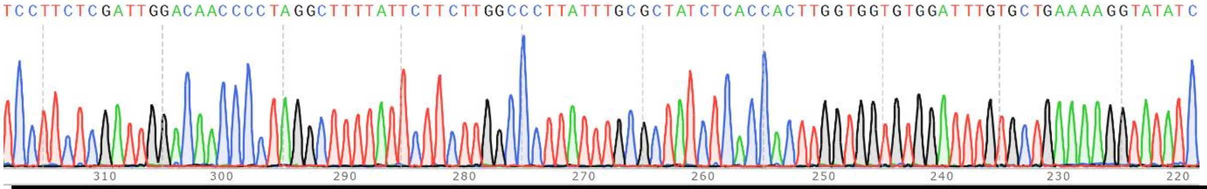


```
PCR_C3 : tccttctcgattggacaacccctaggcttttattcttcttggccctta tttgcgctatctcaccacttggtggtgtggatttgtgctgaaaaggtatatc : 600
r06_C3 : tccttctcgattggacaacccctaggcttttattcttcttggccctta tttgcgctatctcaccacttggtggtgtggatttgtgctgaaaaggtatatc : 600
         TCCTTCTCGATTGGACAACCCCTAGGCTTTTATTCTTCTTGGCCCTTATTTGCGCTATCTCACCACTTGGTGGTGTGGATTTGTGCTGAAAAGGTATATC
```

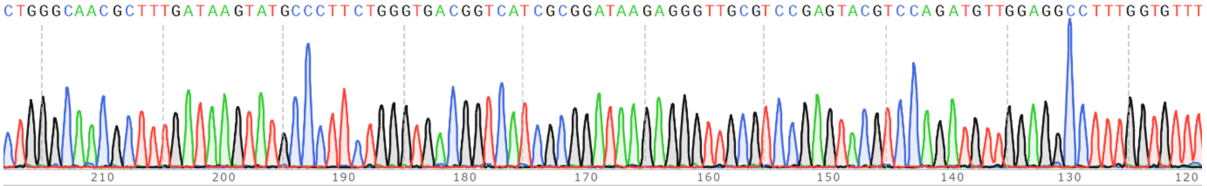


```
PCR_C3 : ctgggcaacgctttgataagtatgcccttctgggtgacggtcatcgcggataagagggttgcgtccgagtacgtccagatgttggaggcctttggtgttt : 700
r06_C3 : ctgggcaacgctttgataagtatgcccttctgggtgacggtcatcgcggataagagggttgcgtccgagtacgtccagatgttggaggcctttggtgttt : 700
         CTGGGCAACGCTTTGATAAGTATGCCCTTCTGGGTGACGGTCATCGCGGATAAGAGGGTTGCGTCCGAGTACGTCCAGATGTTGGAGGCCTTTGGTGTTT
```

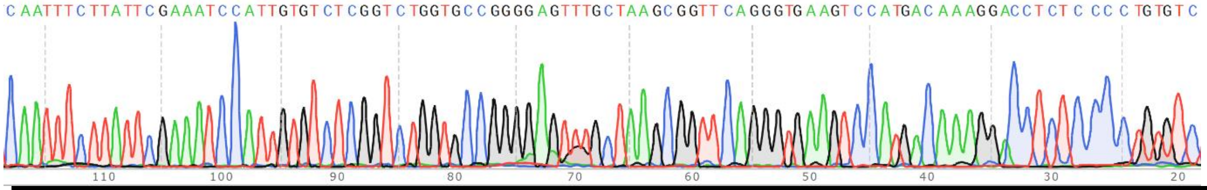


```
PCR_C3 : caatttcttattcgaaatccattgtgtctcggtctggtgccggggagtttgctaagcggttcagggtgaagtccatgacaaaggacctctcccctgtgtc : 800
r06_C3 : caatttcttattcgaaatccattgtgtctcggtctggtgccggggagtttgctaagcggttcagggtgaagtccatgacaaaggacctctcccctgtgtc : 800
         CAATTTCTTATTCGAAATCCATTGTGTCTCGGTCTGGTGCCGGGGAGTTTGCTAAGCGGTTCAGGGTGAAGTCCATGACAAAGGACCTCTCCCCTGTGTC
```

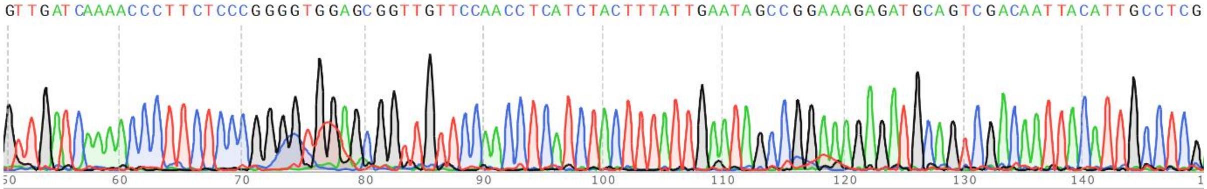


```
PCR_C4 : gttgatcaaaaccccttctcccggggtggagcggttgttccaacctcatctactttattgaatagccggaaagagatgcagtcgacaattacattgcctcg : 100
r06_C4 : gttgatcaaaaccccttctcccggggtggagcggttgttccaacctcatctactttattgaatagccggaaagagatgcagtcgacaattacattgcctcg : 100
         GTTGATCAAAACCCCTTCTCCCGGGGTGGAGCGGTTGTTCCAACCTCATCTACTTTATTGAATAGCCGGAAAGAGATGCAGTCGACAATTACATTGCCTCG
```

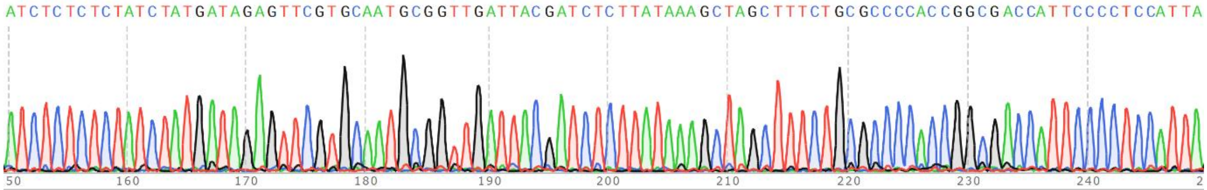


```
PCR_C4 : atctctctctatctatgatagagttcgtgcaatgcggttgattacgatctcttataaagctagctttctgcgccccaccggcgaccattcccctccatta : 200
r06_C4 : atctctctctatctatgatagagttcgtgcaatgcggttgattacgatctcttataaagctagctttctgcgccccaccggcgaccattcccctccatta : 200
         ATCTCTCTCTATCTATGATAGAGTTCGTGCAATGCGGTTGATTACGATCTCTTATAAAGCTAGCTTTCTGCGCCCCACCGGCGACCATTCCCCTCCATTA
```

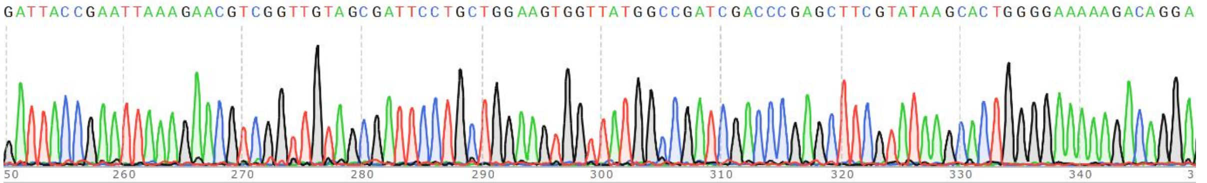


```
PCR_C4 : gattaccgaattaaagaacgtcggttgtagcgattcctgctggaagtggttatggccgatcgacccgagcttcgtataagcactggggaaaaagacagga : 300
r06_C4 : gattaccgaattaaagaacgtcggttgtagcgattcctgctggaagtggttatggccgatcgacccgagcttcgtataagcactggggaaaaagacagga : 300
```

GATTACCGAATTAAAGAACGTCGGTTGTAGCGATTCCTGCTGGAAGTGGTTATGGCCGATCGACCCGAGCTTCGTATAAGCACTGGGGAAAAAGACAGGA

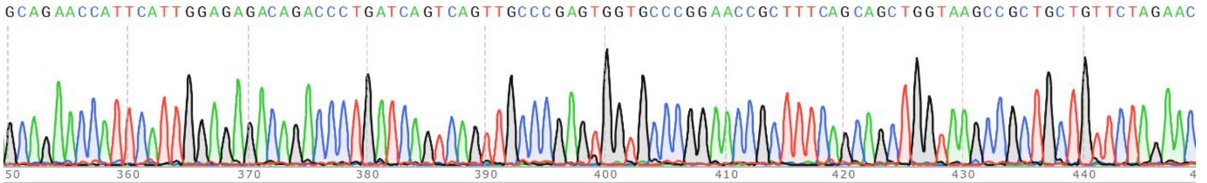


```
PCR_C4 : gcagaaccattcattggagagacagaccctgatcagtcagttgcccgagtggtgcccggaaccgctttcagcagctggtaagccgctgctgttctagaac : 400
r06_C4 : gcagaaccattcattggagagacagaccctgatcagtcagttgcccgagtggtgcccggaaccgctttcagcagctggtaagccgctgctgttctagaac : 400
         GCAGAACCATTCATTGGAGAGACAGACCCTGATCAGTCAGTTGCCCGAGTGGTGCCCGGAACCGCTTTCAGCAGCTGGTAAGCCGCTGCTGTTCTAGAAC
```

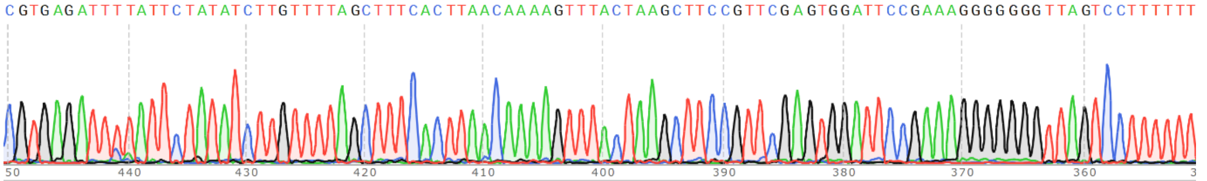


```
PCR_C4 : cgtgagatttattctatatcttgttttagctttcacttaacaaaagtttactaagcttccgttcgagtggattccgaaagggggggttagtcctttttt : 500
r06_C4 : cgtgagattcaattctatatcttgttttagctttcacttaacaaaagtttaccaaacttccgttcgagtggattccgaaagggggggttagtcctttttt : 500
         CGTGAGATT  ATTCTATATCTTGTTTTAGCTTTCACTTAACAAAAGTTTAC AA CTTCCGTTCGAGTGGATTCCGAAAGGGGGGGTTAGTCCTTTTTT
```

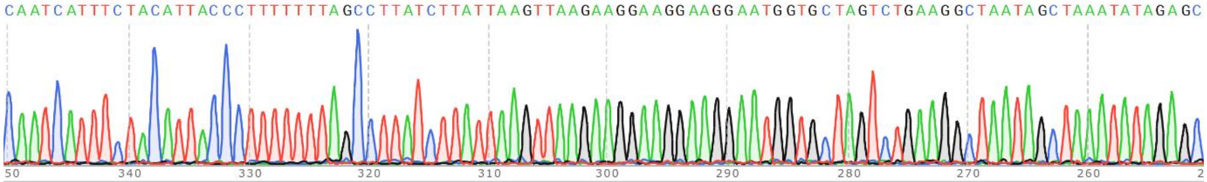


```
PCR_C4 : caatcatttctacattacccttttttttagccttatcttattaagttaagaaggaaggaaggaatggtgctagtctgaaggctaatagctaaatatagagc : 600
r06_C4 : caatcatttctacattacccttttttttagccttatcttattaagttaagaaggaaggaaggaatggtgctagtctgaaggctaatagctaaatatagagc : 600
         CAATCATTTCTACATTACCCTTTTTTTAGCCTTATCTTATTAAGTTAAGAAGGAAGGAAGGAATGGTGCTAGTCTGAAGGCTAATAGCTAAATATAGAGC
```

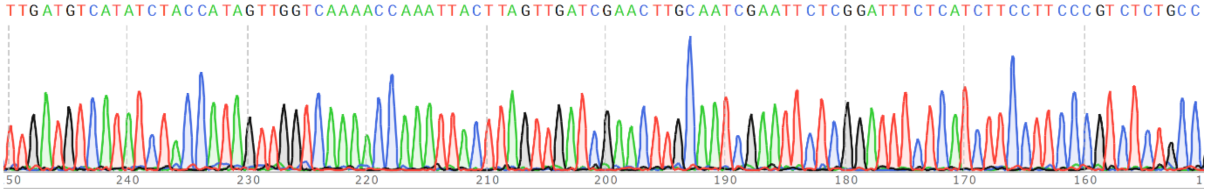


```
PCR_C4 : ttgatgtcatatctaccatagttggtcaaaaccaaattacttagttgatcgaacttgcaatcgaattctcggatttctcatcttccttcccgtctctgcc : 700
r06_C4 : ttgatgtcatatctaccatagttggtcaaaaccaaattacttagttgatcgaacttgcaatcgaattctcggatttctcatcttccttcccgtctctgcc : 700
         TTGATGTCATATCTACCATAGTTGGTCAAAACCAAATTACTTAGTTGATCGAACTTGCAATCGAATTCTCGGATTTCTCATCTTCCTTCCCGTCTCTGCC
```

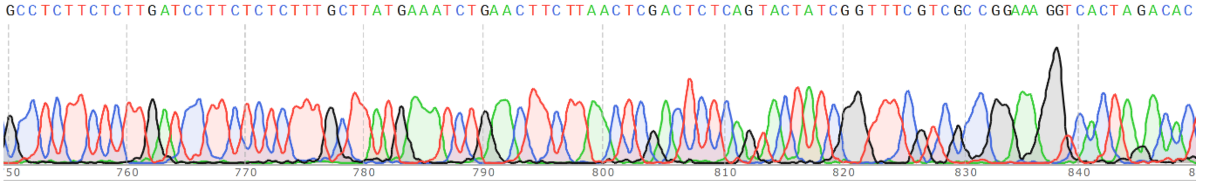


```
PCR_C4 : gcctcttctcttgatccttctctctttgcttatgaaatctgaacttcttaactcgactctcagtactatcggtttcgtcgccggaaaggtcactagacac : 800
r06_C4 : gcctcttctcttgatccttctctctttgcttatgaaatctgaacttcttaactcgactctcagtactatcggtttcgtcgccgg-aaaggtcactagacac : 799
         GCCTCTTCTCTTGATCCTTCTCTCTTTGCTTATGAAATCTGAACTTCTTAACTCGACTCTCAGTACTATCGGTTTCGTCGCCGG AAAGGTCACTAGACAC
```

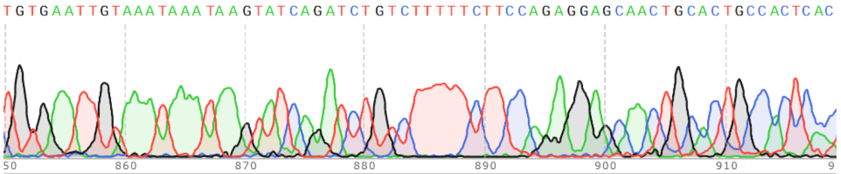


```
PCR_C4 : tgtgaattgtaaataaataagtatcagatctgtctttttcttccagaggagcaactgcactgccactcac : 870
r06_C4 : tgtgaattgtaaataaataagtatcagatctgtctttttcttccagaggagcaactgcactgccactcac : 869
         TGTGAATTGTAAATAAATAAGTATCAGATCTGTCTTTTTCTTCCAGAGGAGCAACTGCACTGCCACTCAC
```

g

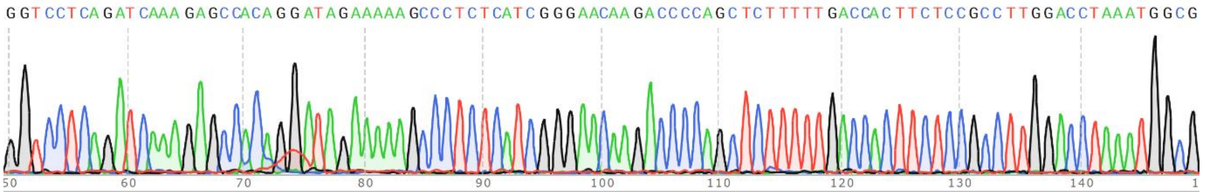

PCR_C1 : ggtcctcagatcaaagagccacaggatagaaaaagccctctcatcgggaacaagaccccagctctttttgaccacttctccgccttggacctaaatggcg : 100
r07_C1 : ggtcctcagatcaaagagccacaggatagaaaaagccctctcatcgggaacaagaccccagctctttttgaccacttctccgccttggacctaaatggcg : 100
GGTCCTCAGATCAAAGAGCCACAGGATAGAAAAAGCCCTCTCATCGGGAACAAGACCCCAGCTCTTTTTGACCACTTCTCCGCCTTGGACCTAAATGGCG

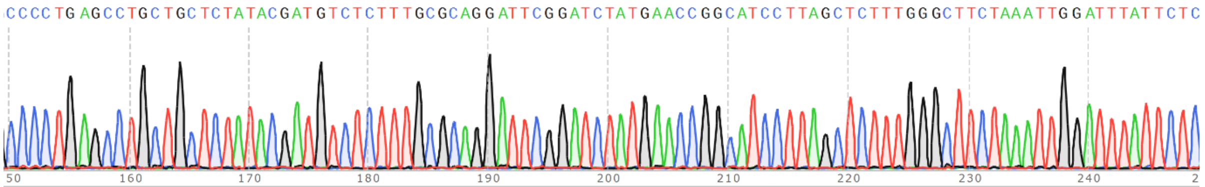

PCR_C1 : cccctgagcctgctgctctatacgatgtctctttgcgcaggattcggatctatgaaccggcatccttagctctttgggcttctaaattggatttattctc : 200
r07_C1 : cccctgagcctgctgctctatacgatgtctctttgcgcaggattcggatctatgaaccggcatccttagctctttgggcttctaaattggatttattctc : 200
CCCCTGAGCCTGCTGCTCTATACGATGTCTCTTTGCGCAGGATTCGGATCTATGAACCGGCATCCTTAGCTCTTTGGGCTTCTAAATTGGATTTATTCTC

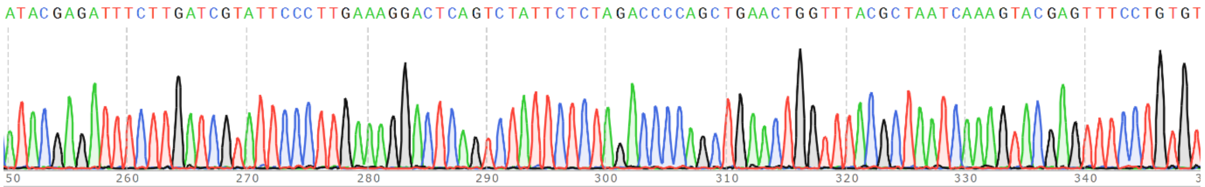

PCR_C1 : atacgagatttcttgatcgtattcccttgaaaggactcagtctattctctagaccccagctgaactggtttacgctaatcaaagtacgagtttcctgtgt : 300
r07_C1 : atacgagatttcttgatcgtattcccttgaaaggactcagtctattctctagaccccagctgaactggtttacgctaatcaaagtacgagtttcctgtgt : 300
ATACGAGATTTCTTGATCGTATTCCCTTGAAAGGACTCAGTCTATTCTCTAGACCCCAGCTGAACTGGTTTACGCTAATCAAAGTACGAGTTTCCTGTGT

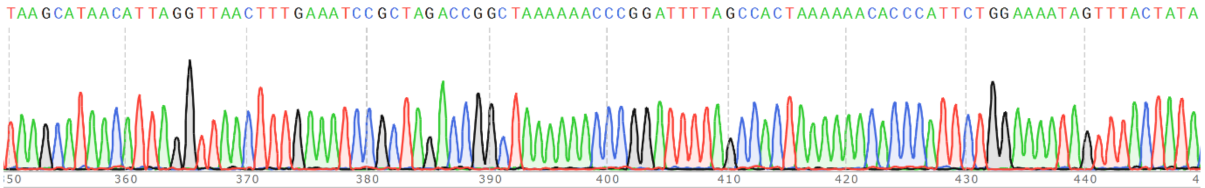

PCR_C1 : taagcataacattaggttaactttgaaatccgctagaccggctaaaaaacccggattttagccactaaaaaacacccattctggaaaatagtttactata : 400
r07_C1 : taagcataacattaggttaactttgaaatccgctagaccggctaaaaaacccggattttagccactaaaaaacacccattctggaaaatagtttactata : 400
TAAGCATAACATTAGGTTAACTTTGAAATCCGCTAGACCGGCTAAAAAACCCGGATTTTAGCCACTAAAAAACACCCATTCTGGAAAATAGTTTACTATA

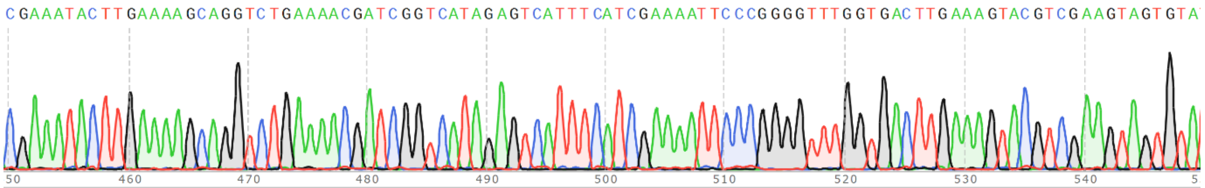

PCR_C1 : cgaaatacttgaaaagcaggtctgaaaacgatcggtcatagagtcatttcatcgaaaattcccggggtttggtgacttgaaagtacgtcgaagtagtgta : 500
r07_C1 : cgaaatacttgaaaagcaggtctgaaaacgatcggtcatagagtcatttcatcgaaaattcccggggtttggtgacttgaaagtacgtcgaagtagtgta : 500
CGAAATACTTGAAAAGCAGGTCTGAAAACGATCGGTCATAGAGTCATTTCATCGAAAATTCCCGGGGTTTGGTGACTTGAAAGTACGTCGAAGTAGTGTA

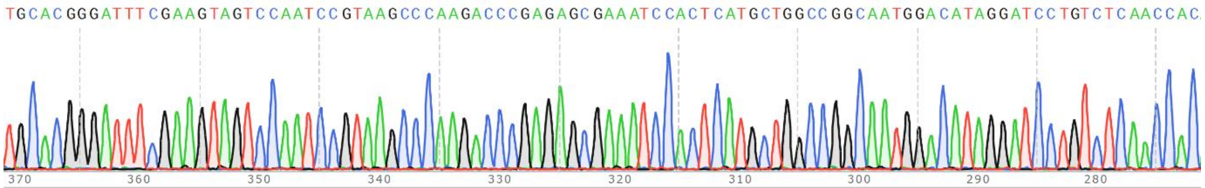

PCR_C1 : tgcacgggatttcgaagtagtccaatccgtaagcccaagacccgagagcgaaatccactcatgctggccggcaatggacataggatcctgtctcaaccac : 600
r07_C1 : tgcacgggatttcgaagtagtccaatccgtaagcccaagacccgagagcgaaatccactcatgctggccggcaatggacataggatcctgtctcaaccac : 600
TGCACGGGATTTCGAAGTAGTCCAATCCGTAAGCCCAAGACCCGAGAGCGAAATCCACTCATGCTGGCCGGCAATGGACATAGGATCCTGTCTCAACCAC

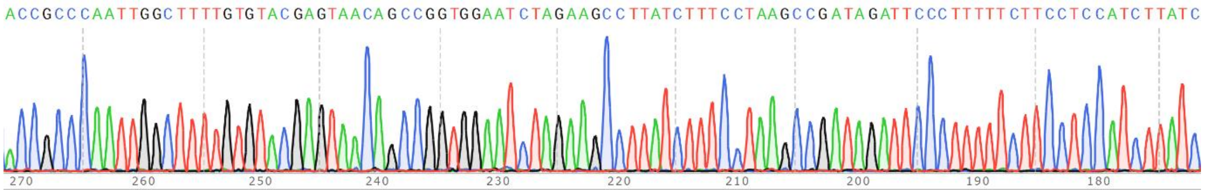

PCR_C1 : accgcccaattggcttttgtgtacgagtaacagccggtggaatctagaagccttatctttcctaagccgatagattccctttttcttcctccatcttatc : 700
r07_C1 : accgcccaattggcttttgtgtacgagtaacagccggtggaatctagaagccttatctttcctaagccgatagattccctttttcttcctccatcttatc : 700
ACCGCCCAATTGGCTTTTGTGTACGAGTAACAGCCGGTGGAATCTAGAAGCCTTATCTTTCCTAAGCCGATAGATTCCCTTTTTCTTCCTCCATCTTATC

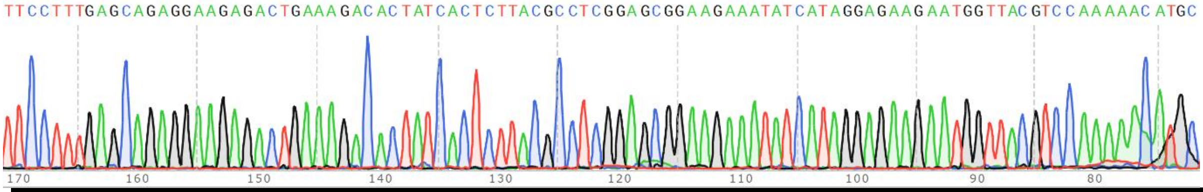


```
PCR_C1 : ttcctttgagcagaggaagagactgaaagacactatcactcttacgcctcggagcggaagaaatatcataggagaagaatggttacgtccaaaaacatgc : 800
r07_C1 : ttcctttgagcagaggaagagactgaaagacactatcactcttacgcctcggagcggaagaaatatcataggagaagaatggttacgtccaaaaacatgc : 800
         TTCCTTTGAGCAGAGGAAGAGACTGAAAGACACTATCACTCTTACGCCTCGGAGCGGAAGAAATATCATAGGAGAAGAATGGTTACGTCCAAAAACATGC
```

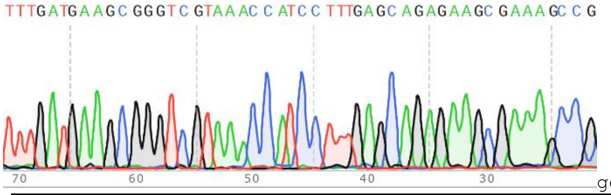

ge

```
PCR_C1 : tttgatgaagcgggtcgtaaaccatcctttgagcagagaagcgaaagccg : 850
r07_C1 : tttgatgaagcgggtcgtaaaccatcctttgagcagagaagcgaaagccg : 850
         TTTGATGAAGCGGGTCGTAAACCATCCTTTGAGCAGAGAAGCGAAAGCCG
```

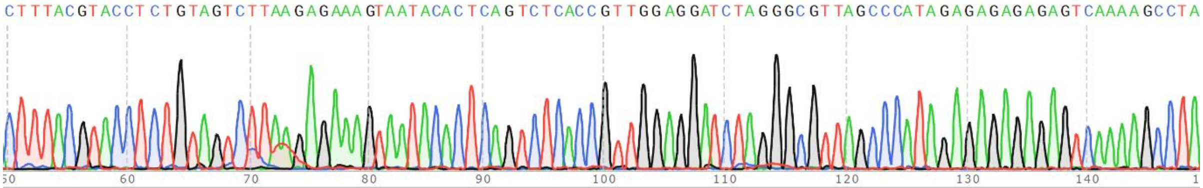


```
PCR_C2 : ctttacgtacctctgtagtcttaagagaaagtaatacactcagtctcaccgttggaggatctagggcgttagcccatagagagagagagagtcaaaagccta : 100
r07_C2 : ctttacgtacctctgtagtcttaagagaaagtaatacactcagtctcaccgttggaggatctagggcgttagcccatagagagagagagagtcaaaagccta : 100
         CTTTACGTACCTCTGTAGTCTTAAGAGAAAGTAATACACTCAGTCTCACCGTTGGAGGATCTAGGGCGTTAGCCCATAGAGAGAGAGAGAGTCAAAAGCCTA
```

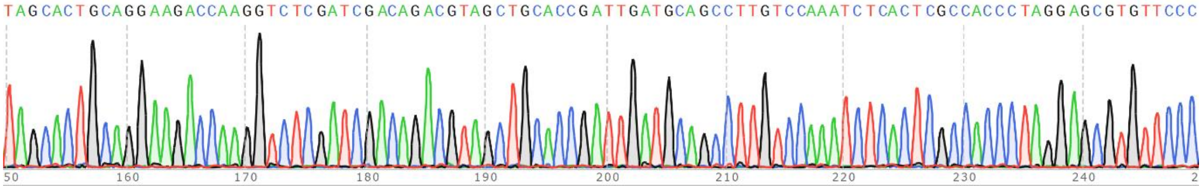


```
PCR_C2 : tagcactgcaggaagaccaaggtctcgatcgacagacgtagctgcaccgattgatgcagccttgtccaaatctcactcgccaccctaggagcgtgttccc : 200
r07_C2 : tagcactgcaggaagaccaaggtctcgatcgacagacgtagctgcaccgattgatgcagccttgtccaaatctcactcgccaccctaggagcgtgttccc : 200
         TAGCACTGCAGGAAGACCAAGGTCTCGATCGACAGACGTAGCTGCACCGATTGATGCAGCCTTGTCCAAATCTCACTCGCCACCCTAGGAGCGTGTTCCC
```

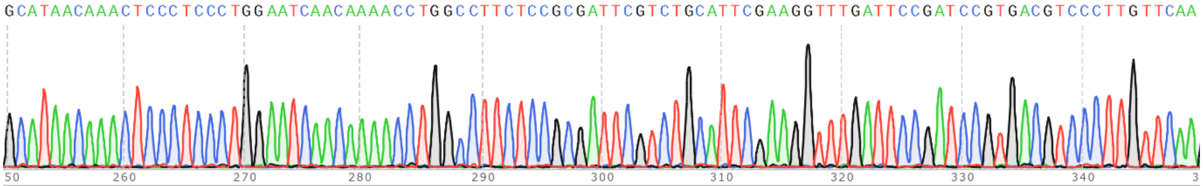


```
PCR_C2 : gcataacaaactccctccctggaatcaacaaaacctggccttctccgcgattcgtctgcattcgaaggtttgattccgatccgtgacgtcccttgttcaa : 300
r07_C2 : gcataacaaactccctccctggaatcaacaaaacctggccttctccgcgattcgtctgcattcgaaggtttgattccgatccgtgacgtcccttgttcaa : 300
         GCATAACAAACTCCCTCCCTGGAATCAACAAAACCTGGCCTTCTCCGCGATTCGTCTGCATTCGAAGGTTTGATTCCGATCCGTGACGTCCCTTGTTCAA
```

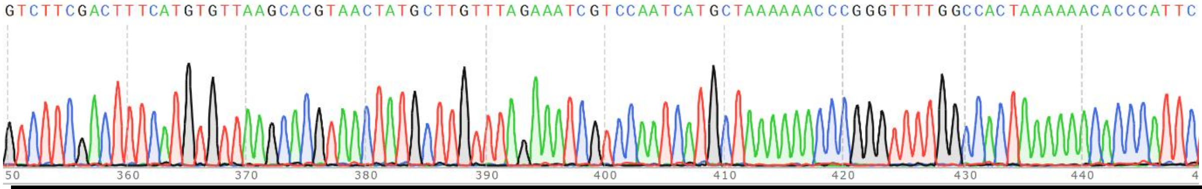


```
PCR_C2 : gtcttcgactttcatgtgttaagcacgtaactatgcttgtttagaaatcgtccaatcatgctaaaaaacccgggttttggccactaaaaaacacccattc : 400
r07_C2 : gtcttcgactttcatgtgttaagcacgtaactatgcttgtttagaaatcgtccaatcatgctaaaaaacccgggttttggccactaaaaaacacccattc : 400
         GTCTTCGACTTTCATGTGTTAAGCACGTAACTATGCTTGTTTAGAAATCGTCCAATCATGCTAAAAAACCCGGGTTTTGGCCACTAAAAAACACCCATTC
```

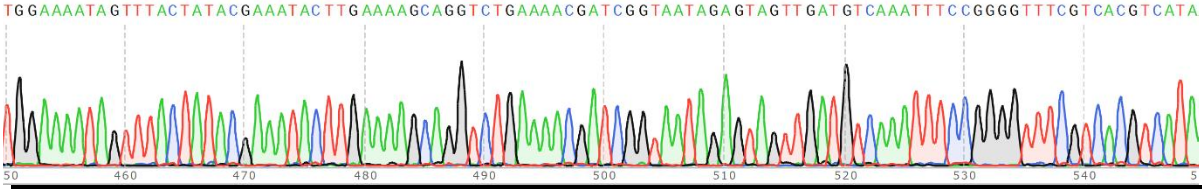


```
PCR_C2 : tggaaaatagtttactatacgaaatacttgaaaagcaggtctgaaaacgatcggtaatagagtagttgatgtcaaatttccggggtttcgtcacgtcata : 500
r07_C2 : tggaaaatagtttactatacgaaatacttgaaaagcaggtctgaaaacgatcggtaatagagtagttgatgtcaaatttccggggtttcgtcacgtcata : 500
         TGGAAAATAGTTTACTATACGAAATACTTGAAAAGCAGGTCTGAAAACGATCGGTAATAGAGTAGTTGATGTCAAATTTCCGGGGTTTCGTCACGTCATA
```

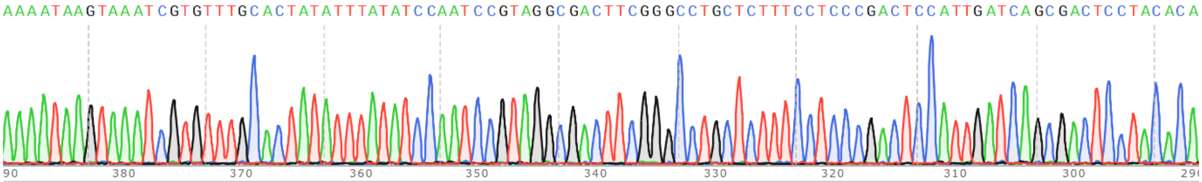

PCR_C2 : aaataagtaaatcgtgtttgcactatatttatatccaatccgtaggcgacttcgggcctgctctttcctcccgactccattgatcagcgactcctacaca : 600
r07_C2 : aaataagtaaatcgtgtttgcactatatttatatccaatccgtaggcgacttcgggcctgctctttcctcccgactccattgatcagcgactcctacaca : 600
AAATAAGTAAATCGTGTTTGCACTATATTTATATCCAATCCGTAGGCGACTTCGGGCCTGCTCTTTCCTCCCGACTCCATTGATCAGCGACTCCTACACA

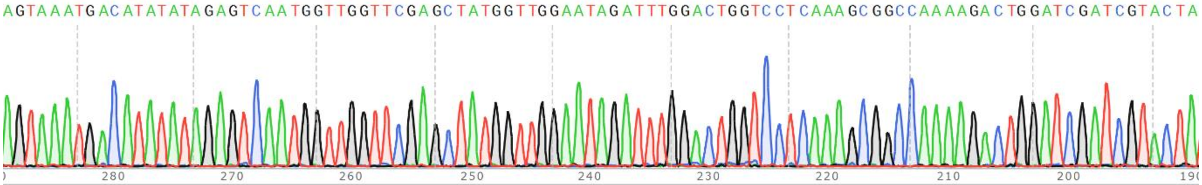

PCR_C2 : agtaaatgacatatatagagtcaatggttggttcgagctatggttggaatagatttggactggtcctcaaagcggccaaaagactggatcgatcgtacta : 700
r07_C2 : agtaaatgacatatatagagtcaatggttggttcgagctatggttggaatagatttggactggtcctcaaagcggccaaaagactggatcgatcgtacta : 700
AGTAAATGACATATATAGAGTCAATGGTTGGTTCGAGCTATGGTTGGAATAGATTTGGACTGGTCCTCAAAGCGGCCAAAAGACTGGATCGATCGTACTA

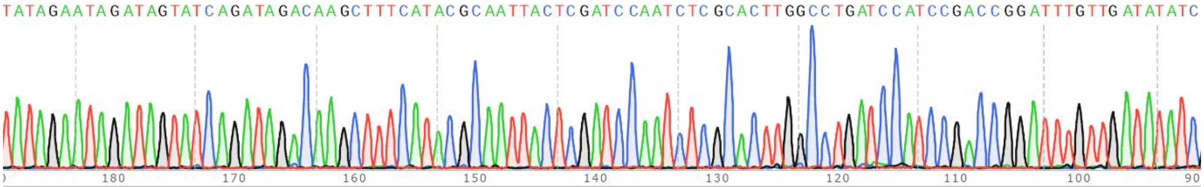

PCR_C2 : tatagaatagatagtatcagatagacaagctttcatacgcaattactcgatccaatctcgcacttggcctgatccatccgaccggatttgttgatatatc : 800
r07_C2 : tatagaatagatagtatcagatagacaagctttcatacgcaattactcgatccaatctcgcacttggcctgatccatccgaccggatttgttgatatatc : 800
TATAGAATAGATAGTATCAGATAGACAAGCTTTCATACGCAATTACTCGATCCAATCTCGCACTTGGCCTGATCCATCCGACCGGATTTGTTGATATATC

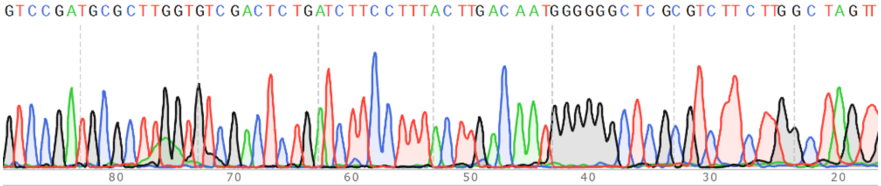

PCR_C2 : gtccgatgcgcttggtgtcgactctgatcttcctttacttgacaatggggggctcgcgtcttcttggctagtt : 873
r07_C2 : gtccgatgcgcttggtgtcgactctgatcttcctttacttgacaatggggggctcgcgtcttcttggctagtt : 873
GTCCGATGCGCTTGGTGTCGACTCTGATCTTCCTTTACTTGACAATGGGGGGCTCGCGTCTTCTTGGCTAGTT

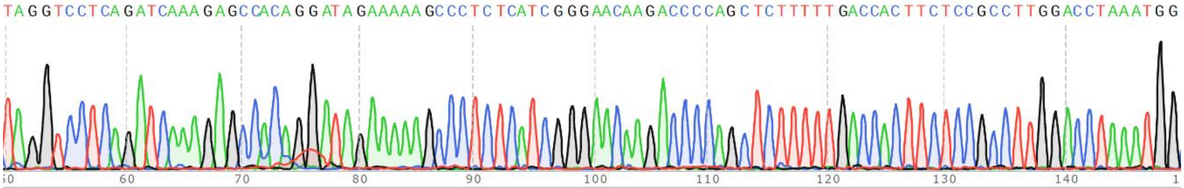

PCR_C3 : taggtcctcagatcaaagagccacaggatagaaaaagccctctcatcgggaacaagaccccagctctttttgaccacttctccgccttggacctaaatgg : 100
r07_C3 : taggtcctcagatcaaagagccacaggatagaaaaagccctctcatcgggaacaagaccccagctctttttgaccacttctccgccttggacctaaatgg : 100
TAGGTCCTCAGATCAAAGAGCCACAGGATAGAAAAAGCCCTCTCATCGGGAACAAGACCCCAGCTCTTTTTGACCACTTCTCCGCCTTGGACCTAAATGG

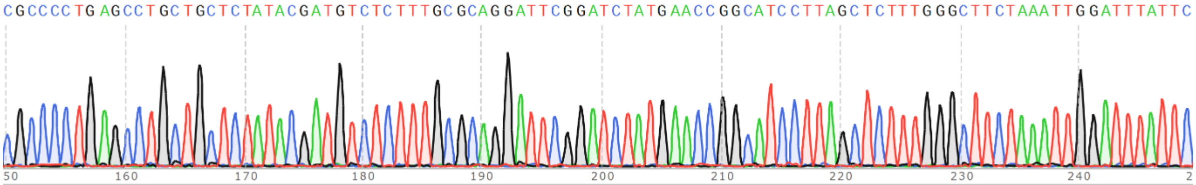

PCR_C3 : cgcccctgagcctgctgctctatacgatgtctctttgcgcaggattcggatctatgaaccggcatccttagctctttgggcttctaaattggatttattc : 200
r07_C3 : cgcccctgagcctgctgctctatacgatgtctctttgcgcaggattcggatctatgaaccggcatccttagctctttgggcttctaaattggatttattc : 200
CGCCCCTGAGCCTGCTGCTCTATACGATGTCTCTTTGCGCAGGATTCGGATCTATGAACCGGCATCCTTAGCTCTTTGGGCTTCTAAATTGGATTTATTC

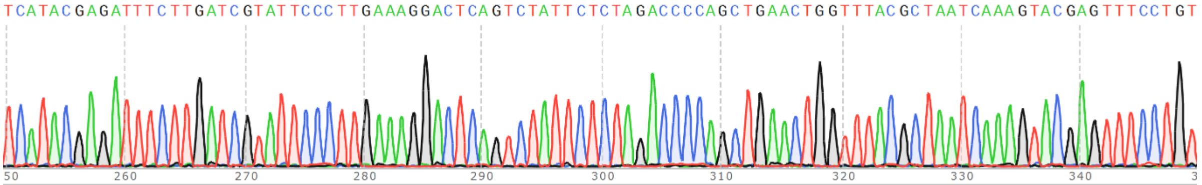

PCR_C3 : tcatacgagatttcttgatcgtattcccttgaaaggactcagtctattctctagaccccagctgaactggtttacgctaatcaaagtacgagtttcctgt : 300
r07_C3 : tcatacgagatttcttgatcgtattcccttgaaaggactcagtctattctctagaccccagctgaactggtttacgctaatcaaagtacgagtttcctgt : 300

```
TCATACGAGATTTCTTGATCGTATTCCCTTGAAAGGACTCAGTCTATTCTCTAGACCCCAGCTGAACTGGTTTACGCTAATCAAAGTACGAGTTTCCTGT
```

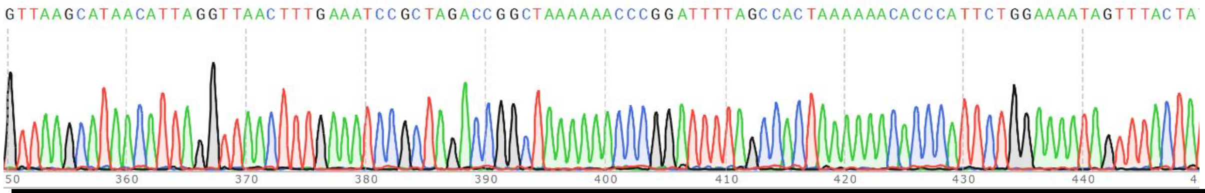

```
PCR_C3 : gttaagcataacattaggttaactttgaaatccgctagaccggctaaaaaacccggattttagccactaaaaaacacccattctggaaaatagtttacta : 400
r07_C3 : gttaagcataacattaggttaactttgaaatccgctagaccggctaaaaaacccggattttagccactaaaaaacacccattctggaaaatagtttacta : 400
         GTTAAGCATAACATTAGGTTAACTTTGAAATCCGCTAGACCGGCTAAAAAACCCGGATTTTAGCCACTAAAAAACACCCATTCTGGAAAATAGTTTACTA
```

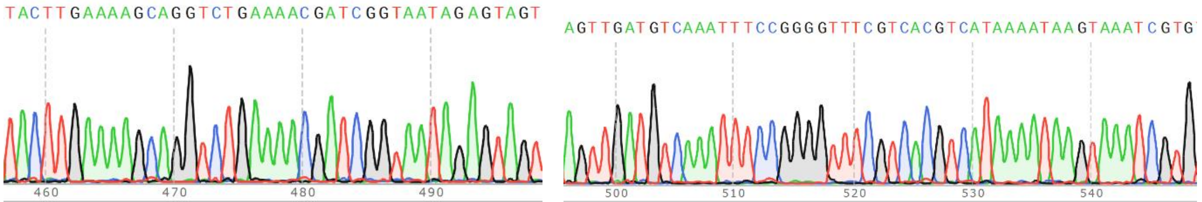

```
PCR_C3 : tacgaaatacttgaaaagcaggtctgaaaacgatcggtaatagagt-agttgatgtcaaatttccggggtttcgtcacgtcataaaataagtaaatcgtg : 499
r07_C3 : tacgaaatacttgaaaagcaggtctgaaaacgatcggtcatagagtcatttcatcgaaaattcccggggtttggtgacgtcataaaataagtaaatcgtg : 500
         TACGAAATACTTGAAAAGCAGGTCTGAAAACGATCGGT ATAGAGT A TT AT   AAATT CCGGGGTTT GT ACGTCATAAAATAAGTAAATCGTG
```

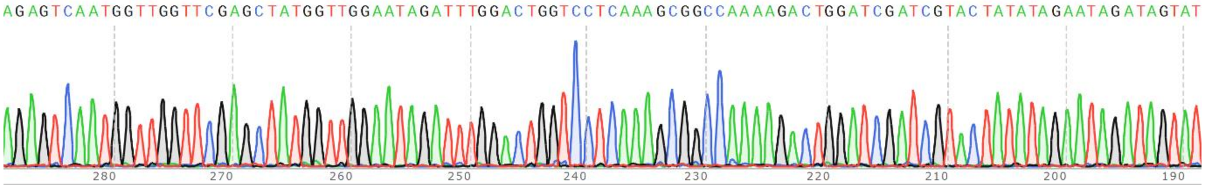

```
PCR_C3 : tttgcactatatttatatccaatccgtaggcgacttcgggcctgctctttcctcccgactccattgatcagcgactcctacacaagtaaatgacatatat : 599
r07_C3 : tttgcactatatttatatccaatccgtaggcgacttcgggcctgctctttcctcccgactccattgatcagcgactcctacacaagtaaatgacatatat : 600
         TTTGCACTATATTTATATCCAATCCGTAGGCGACTTCGGGCCTGCTCTTTCCTCCCGACTCCATTGATCAGCGACTCCTACACAAGTAAATGACATATAT
```

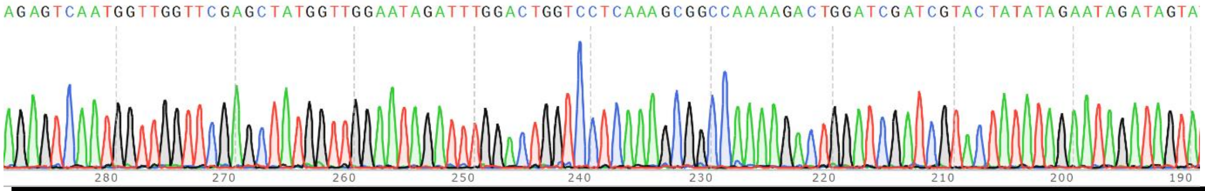

```
PCR_C3 : agagtcaatggttggttcgagctatggttggaatagatttggactggtcctcaaagcggccaaaagactggatcgatcgtactatatagaatagatagta : 699
r07_C3 : agagtcaatggttggttcgagctatggttggaatagatttggactggtcctcaaagcggccaaaagactggatcgatcgtactatatagaatagatagta : 700
         AGAGTCAATGGTTGGTTCGAGCTATGGTTGGAATAGATTTGGACTGGTCCTCAAAGCGGCCAAAAGACTGGATCGATCGTACTATATAGAATAGATAGTA
```

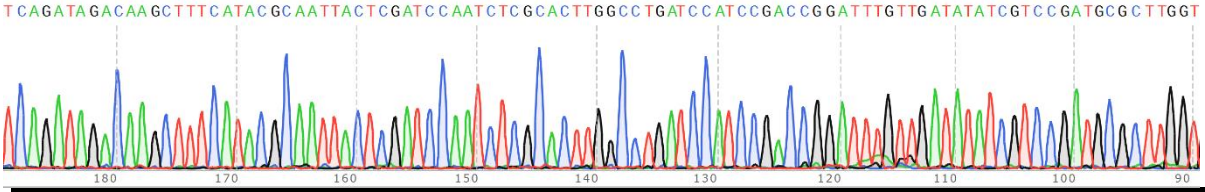

```
PCR_C3 : tcagatagacaagctttcatacgcaattactcgatccaatctcgcacttggcctgatccatccgaccggatttgttgatatatcgtccgatgcgcttggt : 799
r07_C3 : tcagatagacaagctttcatacgcaattactcgatccaatctcgcacttggcctgatccatccgaccggatttgttgatatatcgtccgatgcgcttggt : 800
         TCAGATAGACAAGCTTTCATACGCAATTACTCGATCCAATCTCGCACTTGGCCTGATCCATCCGACCGGATTTGTTGATATATCGTCCGATGCGCTTGGT
```

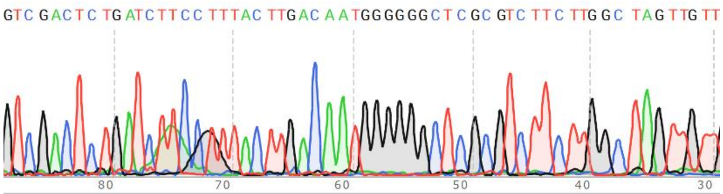

```
PCR_C3 : gtcgactctgatcttcctttacttgacaatggggggctcgcgtcttcttggctagttgtt : 859
r07_C3 : gtcgactctgatcttcctttacttgacaatggggggctcgcgtcttcttggctagttgtt : 859
         GTCGACTCTGATCTTCCTTTACTTGACAAT GGGGGGCTCGCGTCTTCTTGGCTAGTTGTT
```

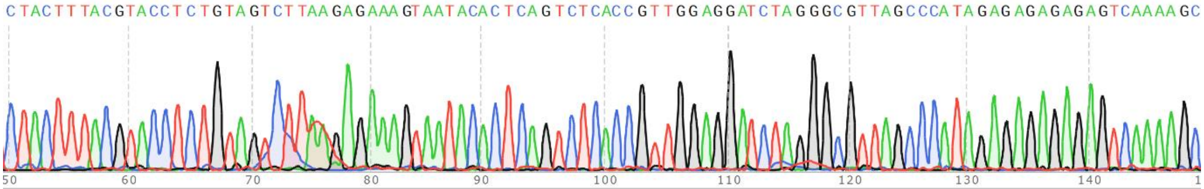


```
PCR_C4 : ctactttacgtacctctgtagtcttaagagaaagtaatacactcagtctcaccgttggaggatctagggcgttagcccatagagagagagagtcaaaagc : 100
r07_C4 : ctactttacgtacctctgtagtcttaagagaaagtaatacactcagtctcaccgttggaggatctagggcgttagcccatagagagagagagtcaaaagc : 100
         CTACTTTACGTACCTCTGTAGTCTTAAGAGAAAGTAATACACTCAGTCTCACCGTTGGAGGATCTAGGGCGTTAGCCCATAGAGAGAGAGAGTCAAAAGC
```

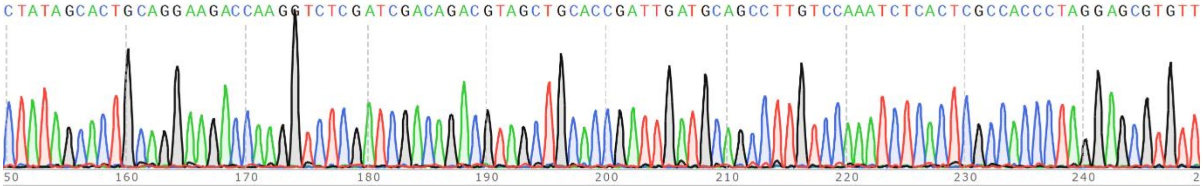


```
PCR_C4 : ctatagcactgcaggaagaccaaggtctcgatcgacagacgtagctgcaccgattgatgcagccttgtccaaatctcactcgccaccctaggagcgtgtt : 200
r07_C4 : ctatagcactgcaggaagaccaaggtctcgatcgacagacgtagctgcaccgattgatgcagccttgtccaaatctcactcgccaccctaggagcgtgtt : 200
         CTATAGCACTGCAGGAAGACCAAGGTCTCGATCGACAGACGTAGCTGCACCGATTGATGCAGCCTTGTCCAAATCTCACTCGCCACCCTAGGAGCGTGTT
```

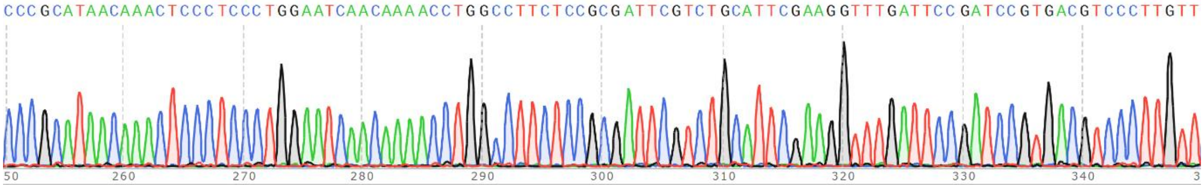


```
PCR_C4 : cccgcataacaaactccctccctggaatcaacaaaacctggccttctccgcgattcgtctgcattcgaaggtttgattccgatccgtgacgtcccttgtt : 300
r07_C4 : cccgcataacaaactccctccctggaatcaacaaaacctggccttctccgcgattcgtctgcattcgaaggtttgattccgatccgtgacgtcccttgtt : 300
         CCCGCATAACAAACTCCCTCCCTGGAATCAACAAAACCTGGCCTTCTCCGCGATTCGTCTGCATTCGAAGGTTTGATTCCGATCCGTGACGTCCCTTGTT
```

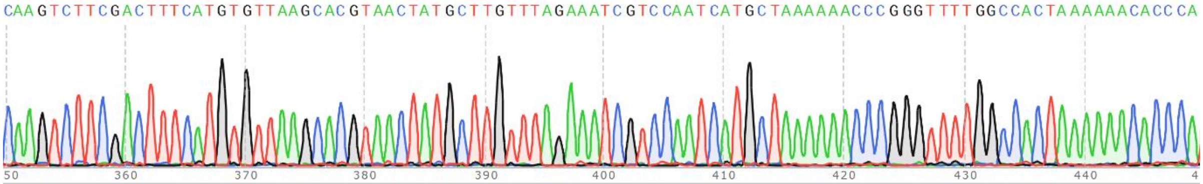


```
PCR_C4 : caagtcttcgactttcatgtgttaagcacgtaactatgcttgtttagaaatcgtccaatcatgctaaaaaacccgggttttggccactaaaaaacaccca : 400
r07_C4 : caagtcttcgactttcatgtgttaagcacgtaactatgcttgtttagaaatcgtccaatcatgctaaaaaacccggattttagccactaaaaaacaccca : 400
         CAAGTCTTCGACTTTCATGTGTTAAGCACGTAACTATGCTTGTTTAGAAATCGTCCAATCATGCTAAAAAACCCGG TTTT GCCACTAAAAAACACCCA
```

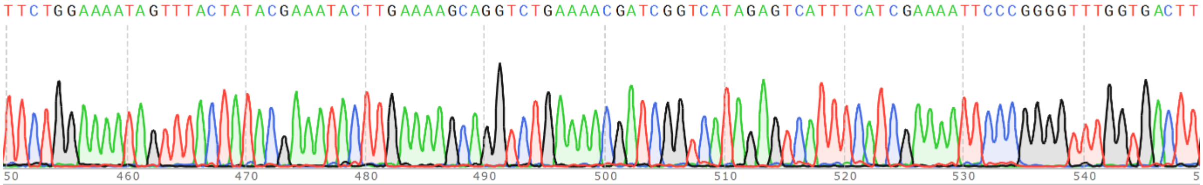


```
PCR_C4 : ttctggaaaatagtttactatacgaaatacttgaaaagcaggtctgaaaacgatcggtcatagagtcatttcatcgaaaattcccggggtttggtgactt : 500
r07_C4 : ttctggaaaatagtttactatacgaaatacttgaaaagcaggtctgaaaacgatcggtcatagagtcatttcatcgaaaattcccggggtttggtgactt : 500
         TTCTGGAAAATAGTTTACTATACGAAATACTTGAAAAGCAGGTCTGAAAACGATCGGTCATAGAGTCATTTCATCGAAAATTCCCGGGGTTTGGTGACTT
```

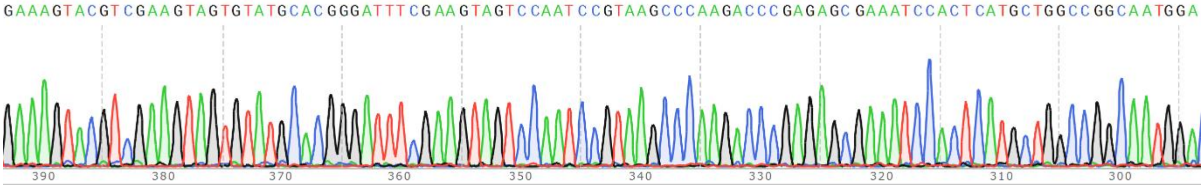


```
PCR_C4 : gaaagtacgtcgaagtagtgtatgcacgggatttcgaagtagtccaatccgtaagcccaagacccgagagcgaaatccactcatgctggccggcaatgga : 600
r07_C4 : gaaagtacgtcgaagtagtgtatgcacgggatttcgaagtagtccaatccgtaagcccaagacccgagagcgaaatccactcatgctggccggcaatgga : 600
         GAAAGTACGTCGAAGTAGTGTATGCACGGGATTTCGAAGTAGTCCAATCCGTAAGCCCAAGACCCGAGAGCGAAATCCACTCATGCTGGCCGGCAATGGA
```

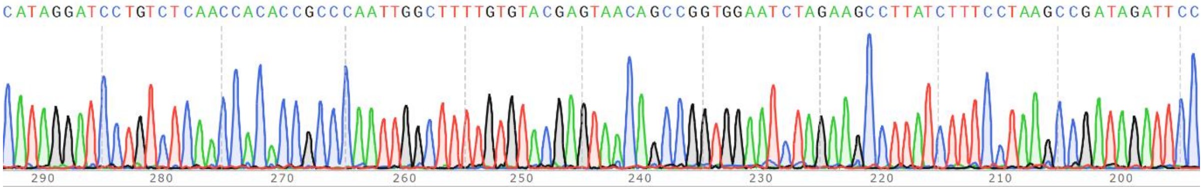


```
PCR_C4 : cataggatcctgtctcaaccacaccgcccaattggcttttgtgtacgagtaacagccggtggaatctagaagccttatctttcctaagccgatagattcc : 700
r07_C4 : cataggatcctgtctcaaccacaccgcccaattggcttttgtgtacgagtaacagccggtggaatctagaagccttatctttcctaagccgatagattcc : 700
         CATAGGATCCTGTCTCAACCACACCGCCCAATTGGCTTTTGTGTACGAGTAACAGCCGGTGGAATCTAGAAGCCTTATCTTTCCTAAGCCGATAGATTCC
```

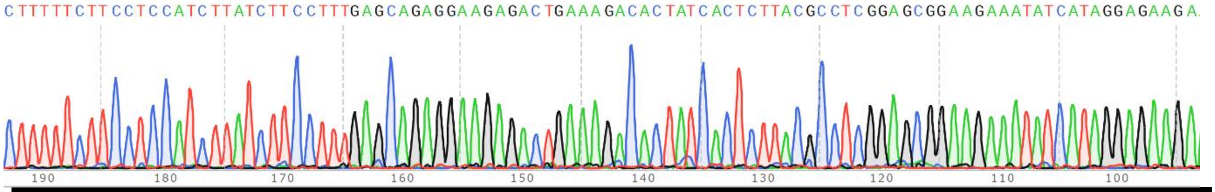

```
PCR_C4 : cttttcttcctccatcttatcttcctttgagcagaggaagagactgaaagacactatcactcttacgcctcggagcggaagaaatatcataggagaaga : 799
r07_C4 : cttttcttcctccatcttatcttcctttgagcagaggaagagactgaaagacactatcactcttacgcctcggagcggaagaaatatcataggagaaga : 800
         CTTTTTCTTCCTCCATCTTATCTTCCTTTGAGCAGAGGAAGAGACTGAAAGACACTATCACTCTTACGCCTCGGAGCGGAAGAAATATCATA GAGAAGA
```

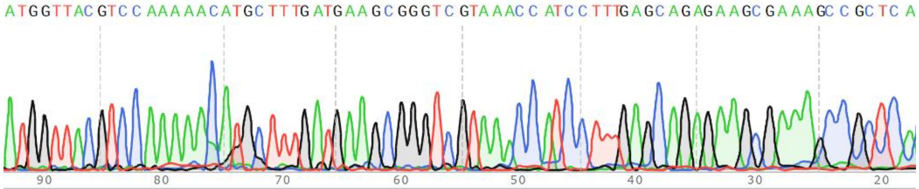

```
PCR_C4 : atggttacgtccaaaaacatgctttgatgaagcgggtcgtaaaccatcctttgagcagagaagcgaaagccgctca : 876
r07_C4 : atggttacgtccaaaaacatgctttgatgaagcgggtcgtaaaccatcctttgagcagagaagcgaaagccgctca : 876
         ATGGTTACGTCCAAAAACATGCTTTGATGAAGCGGGTCGTAAACCATCCTTTGAGCAGAGAAGCGAAAGCCGCTCA
```

h

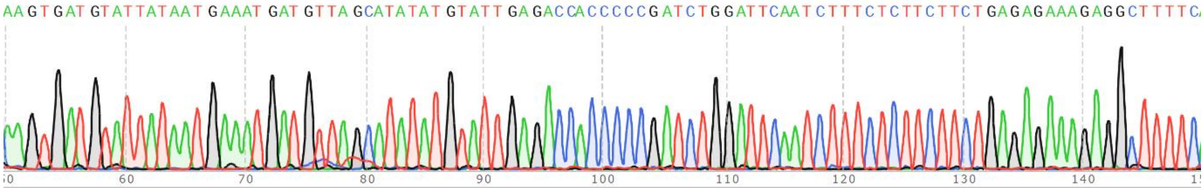

```
PCR_C1 : aagtgatgtattataatgaaatgatgttagcatatatgtattgagaccacccccgatctggattcaatctttctcttcttctgagagaaagaggcttttc : 100
r08_C1 : aagtgatgtattataatgaaatgatgttagcatatatgtattgagaccacccccgatctggattcaatctttctcttcttctgagagaaagaggcttttc : 100
         AAGTGATGTATTATAATGAAATGATGTTAGCATATATGTATTGAGACCACCCCCGATCTGGATTCAATCTTTCTCTTCTTCTGAGAGAAAGAGGCTTTTC
```

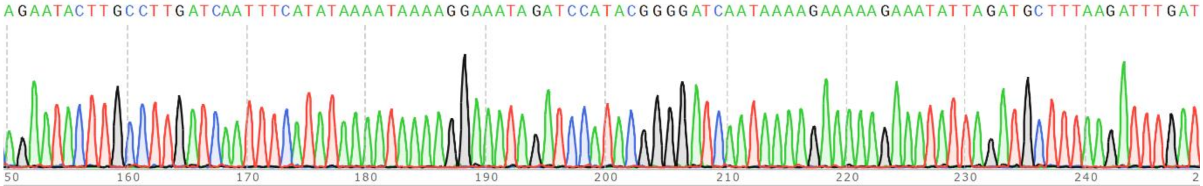

```
PCR_C1 : agaatacttgccttgatcaatttcatataaaataaaaggaaatagatccatacggggatcaataaaagaaaaagaaatattagatgctttaagatttgat : 200
r08_C1 : agaatacttgccttgatcaatttcatataaaataaaaggaaatagatccatacggggatcaataaaagaaaaagaaatattagatgctttaagatttgat : 200
         AGAATACTTGCCTTGATCAATTTCATATAAAATAAAAGGAAATAGATCCATACGGGGATCAATAAAAGAAAAAGAAATATTAGATGCTTTAAGATTTGAT
```

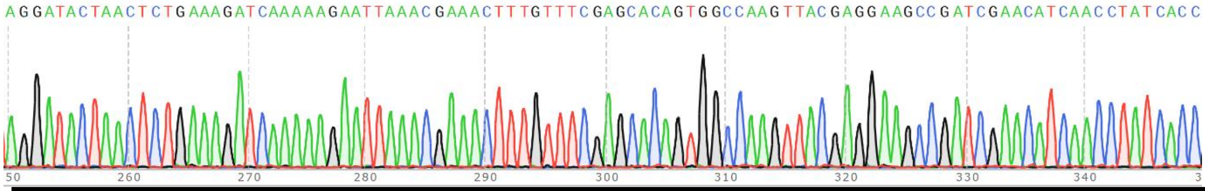

```
PCR_C1 : aggatactaactctgaaagatcaaaaagaattaaacgaaactttgtttcgagcacagtggccaagttacgaggaagccgatcgaacatcaacctatcacc : 300
r08_C1 : aggatactaactctgaaagatcaaaaagaattaaacgaaactttgtttcgagcacagtggccaagttacgaggaagccgatcgaacatcaacctatcacc : 300
         AGGATACTAACTCTGAAAGATCAAAAAGAATTAAACGAAACTTTGTTTCGAGCACAGTGGCCAAGTTACGAGGAAGCCGATCGAACATCAACCTATCACC
```

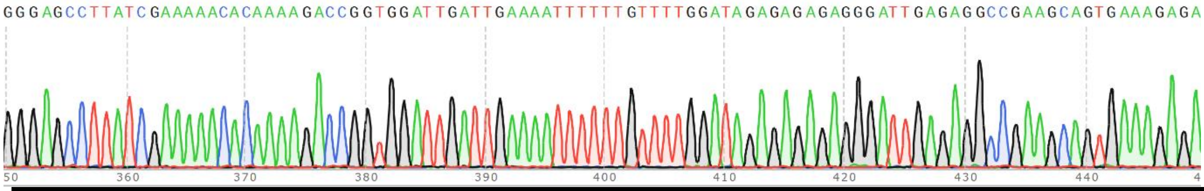

```
PCR_C1 : gggagccttatcgaaaaacacaaaagaccggtggattgattgaaaatttttttgttttggatagagagagagggattgagaggccgaagcagtgaaagaga : 400
r08_C1 : gggagccttatcgaaaaacacaaaagaccggtggattgattgaaaatttttttgttttggatagagagagagggattgagaggccgaagcagtgaaagaga : 400
         GGGAGCCTTATCGAAAAACACAAAAGACCGGTGGATTGATTGAAAATTTTTTTGTTTTGGATAGAGAGAGAGGGATTGAGAGGCCGAAGCAGTGAAAGAGA
```

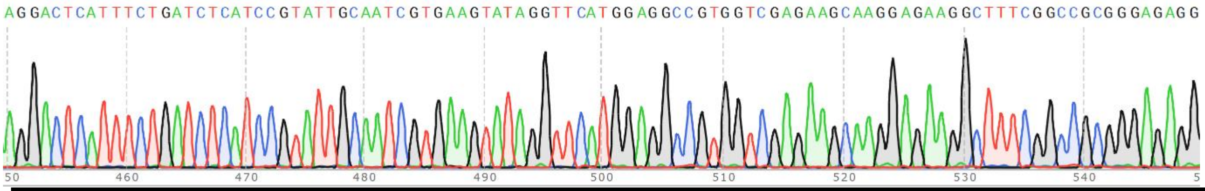

```
PCR_C1 : aggactcatttctgatctcatccgtattgcaatcgtgaagtataggttcatggaggccgtggtcgagaagcaaggagaaggctttcggccgcgggagagg : 500
r08_C1 : aggactcatttctgatctcatccgtattgcaatcgtgaagtataggttcatggaggccgtggtcgagaagcaaggagaaggctttcggccgcgggagagg : 500
         AGGACTCATTTCTGATCTCATCCGTATTGCAATCGTGAAGTATAGGTTCATGGAGGCCGTGGTCGAGAAGCAAGGAGAAGGCTTTCGGCCGCGGGAGAGG
```

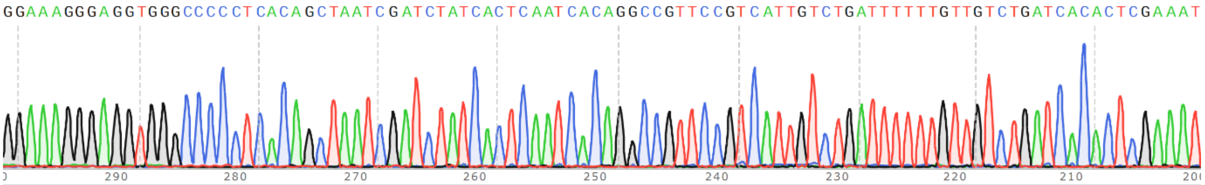

```
PCR_C1 : ggaaagggaggtgggccccctcacagctaatcgatctatcactcaatcacaggccgttccgtcattgtctgattttttgttgtctgatcacactcgaaat : 600
r08_C1 : ggaaagggaggtgggccccctcacagctaatcgatctatcactcaatcacaggccgttccgtcattgtctgattttttgttgtctgatcacactcgaaat : 600
         GGAAAGGGAGGTGGGCCCCCTCACAGCTAATCGATCTATCACTCAATCACAGGCCGTTCCGTCATTGTCTGATTTTTTGTTGTCTGATCACACTCGAAAT
```

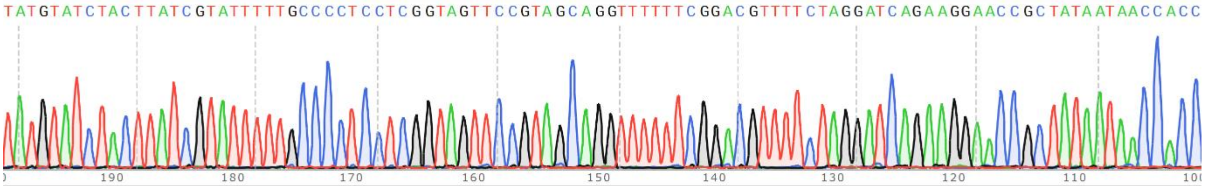

```
PCR_C1 : tatgtatctacttatcgtatttttgcccctcctcggtagttccgtagcaggttttttcggacgttttctaggatcagaaggaaccgctataataaccacc : 700
r08_C1 : tatgtatctacttatcgtatttttgcccctcctcggtagttccgtagcaggttttttcggacgttttctaggatcagaaggaaccgctataataaccacc : 700
         TATGTATCTACTTATCGTATTTTTGCCCCTCCTCGGTAGTTCCGTAGCAGGTTTTTTCGGACGTTTTCTAGGATCAGAAGGAACCGCTATAATAACCACC
```

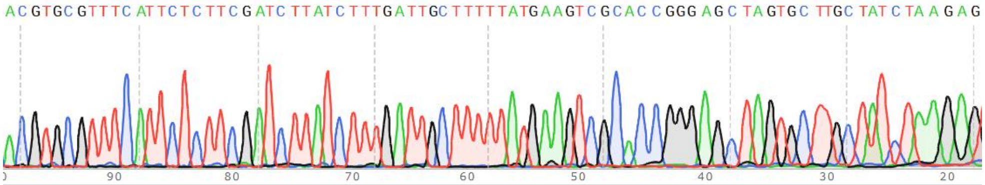

```
PCR_C1 : acgtgcgtttcattctcttcgatcttatctttgattgctttttatgaagtcgcaccgggagctagtgcttgctatctaagag : 782
r08_C1 : acgtgcgtttcattctcttcgatcttatctttgattgctttttatgaagtcgcaccgggagctagtgcttgctatctaagag : 782
         ACGTGCGTTTCATTCTCTTCGATCTTATCTTTGATTGCTTTTTATGAAGTCGCACCGGGAGCTAGTGCTTGCTATCTAAGAG
```

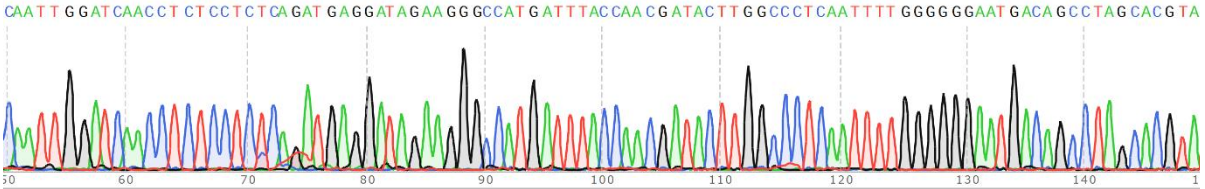

```
PCR_C2 : caattggatcaacctctcctctcagatgaggatagaagggccatgatttaccaacgatacttggccctcaattttggggggaatgacagcctagcacgta : 100
r08_C2 : caattggatcaacctctcctctcagatgaggatagaagggccatgatttaccaacgatacttggccctcaattttggggggaatgacagcctagcacgta : 100
         CAATTGGATCAACCTCTCCTCTCAGATGAGGATAGAAGGGCCATGATTTACCAACGATACTTGGCCCTCAATTTTGGGGGGAATGACAGCCTAGCACGTA
```

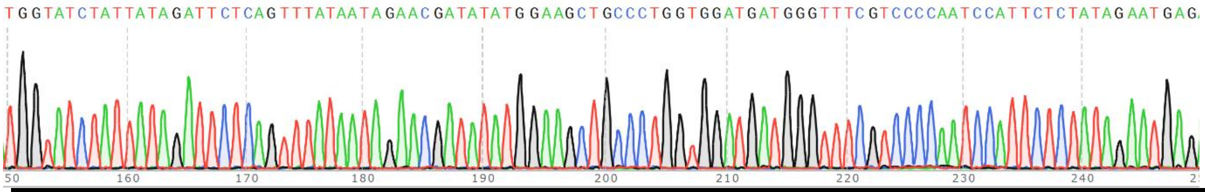

```
PCR_C2 : tggtatctattatagattctcagtttataatagaacgatatatggaagctgccctggtggatgatgggtttcgtccccaatccattctctatagaatgag : 200
r08_C2 : tggtatctattatagattctcagtttataatagaacgatatatggaagctgccctggtggatgatgggtttcgtccccaatccattctctatagaatgag : 200
         TGGTATCTATTATAGATTCTCAGTTTATAATAGAACGATATATGGAAGCTGCCCTGGTGGATGATGGGTTTCGTCCCCAATCCATTCTCTATAGAATGAG
```

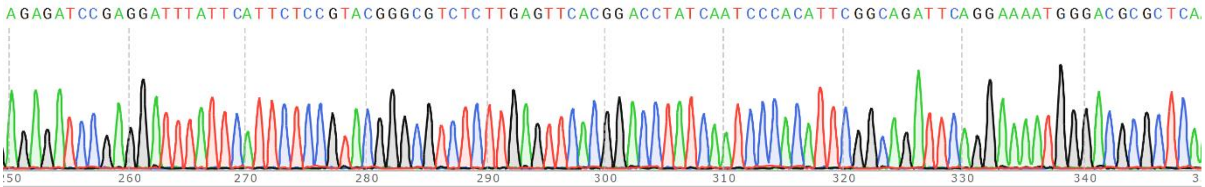

```
PCR_C2 : agagatccgaggatttattcattctccgtacgggcgtctcttgagttcacggacctatcaatcccacattcggcagattcaggaaaatgggacgcgctca : 300
```

r08_C2 : agagatccgaggatttattcattctccgtacgggcgtctcttgagttcacggacctatcaatcccacattcggcagattcaggaaaatgggacgcgctca : 300
AGAGATCCGAGGATTTATTCATTCTCCGTACGGGCGTCTCTTGAGTTCACGGACCTATCAATCCCACATTCGGCAGATTCAGGAAAATGGGACGCGCTCA

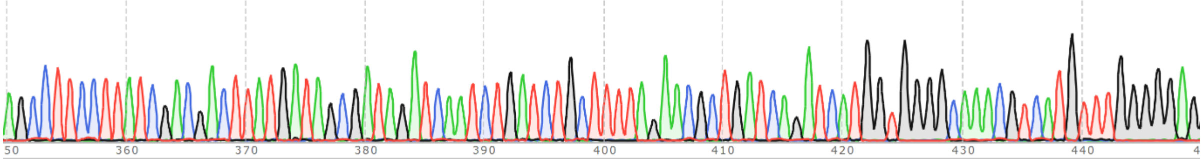

PCR_C2 : agccttccttatcgacgacttatgatagcgatagatcaatctgatctgcttttagaacgctgatcagatcatggtggggcaaacgtcatgtttgggggag : 400
r08_C2 : agccttccttatcgacgacttatgatagcgatagatcaatctgatctgcttttagaacgctgatcagatcatggtggggcaaacgtcatgtttgggggag : 400
AGCCTTCCTTATCGACGACTTATGATAGCGATAGATCAATCTGATCTGCTTTTAGAACGCTGATCAGATCATGGTGGGGCAAACGTCATGTTTGGGGGAG

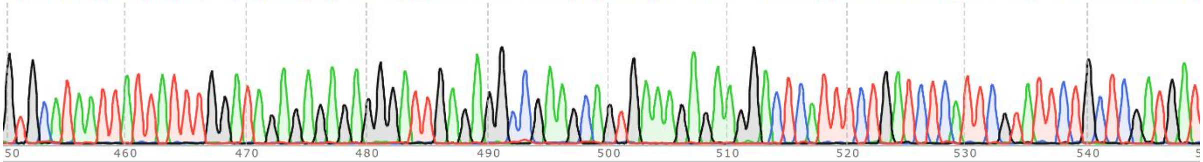

PCR_C2 : gtgcataattattatttggatagagagagagggattgagaggccgaagcagtgaaagagaaggactcatttctgatctcattcgtattctgctcgatgat : 500
r08_C2 : gtgcataattattatttggatagagagagagggattgagaggccgaagcagtgaaagagaaggactcatttctgatctcattcgtattctgctcgatgat : 500
GTGCATAATTATTATTTGGATAGAGAGAGAGGGATTGAGAGGCCGAAGCAGTGAAAGAGAAGGACTCATTTCTGATCTCATTCGTATTCTGCTCGATGAT

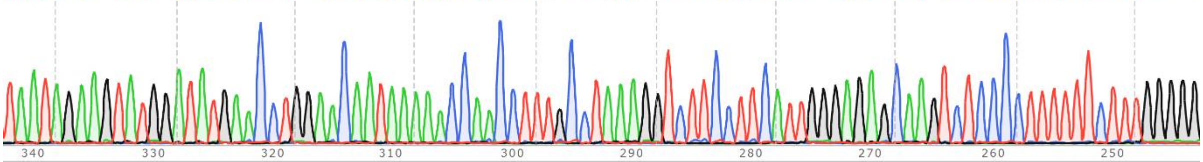

PCR_C2 : taatagaagtatggatatgaacctggaacaataaaaaccaacctttgcctaaaggtcttccttcattgggagagcaagtctcccctttttttctttggggg : 600
r08_C2 : taatagaagtatggatatgaacctggaacaataaaaaccaacctttgcctaaaggtcttccttcattgggagagcaagtctcccctttttttctttggggg : 600
TAATAGAAGTATGGATATGAACCTGGAACAATAAAAACCAACCTTTGCCTAAAGGTCTTCCTTCATTGGGAGAGCAAGTCTCCCCTTTTTTTCTTTGGGGG

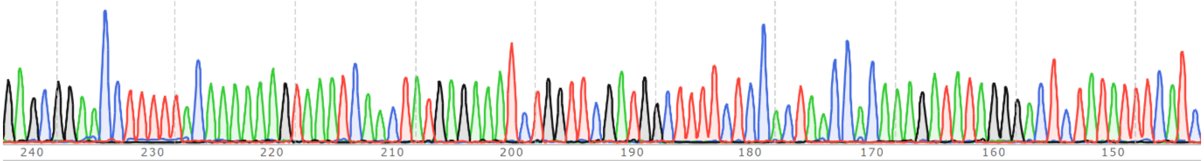

PCR_C2 : gagcggaacctttttacaaaaaagtaaatcaactatgagaaatctggttcgatggcttttctccactaaccacaaagatatagggactctatatttcatc : 700
r08_C2 : gagcggaacctttttacaaaaaagtaaatcaactatgagaaatctggttcgatggcttttctccactaaccacaaagatatagggactctatatttcatc : 700
GAGCGGAACCTTTTTACAAAAAAGTAAATCAACTATGAGAAATCTGGTTCGATGGCTTTTCTCCACTAACCACAAAGATATAGGGACTCTATATTTCATC

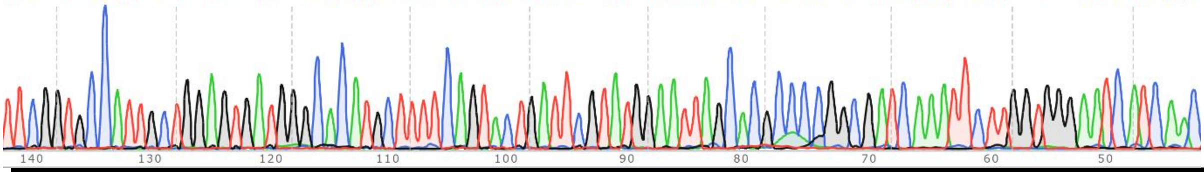

PCR_C2 : ttcggtgccattgctggagtgatgggcacatgcttttcagtactgattcgtatggaattagcacgccccggcgatcaaattcttggtgggaatcatcaac : 800
r08_C2 : ttcggtgccattgctggagtgatgggcacatgcttttcagtactgattcgtatggaattagcacgccccggcgatcaaattcttggtgggaatcatcaac : 800
TTCGGTGCCATTGCTGGAGTGATGGGCACATGCTTTTCAGTACTGATTCGTATGGAATTAGCACGCCCCGGCGATCAAATTCTTGGTGGGAATCATCAAC

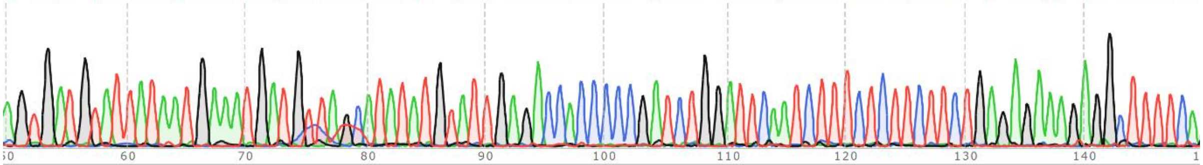

PCR_C3 : agtgatgtattataatgaaatgatgttagcatatatgtattgagaccacccccgatctggattcaatctttctcttcttctgagagaaagaggcttttca : 100
r08_C3 : agtgatgtattataatgaaatgatgttagcatatatgtattgagaccacccccgatctggattcaatctttctcttcttctgagagaaagaggcttttca : 100
AGTGATGTATTATAATGAAATGATGTTAGCATATATGTATTGAGACCACCCCCGATCTGGATTCAATCTTTCTCTTCTTCTGAGAGAAAGAGGCTTTTCA

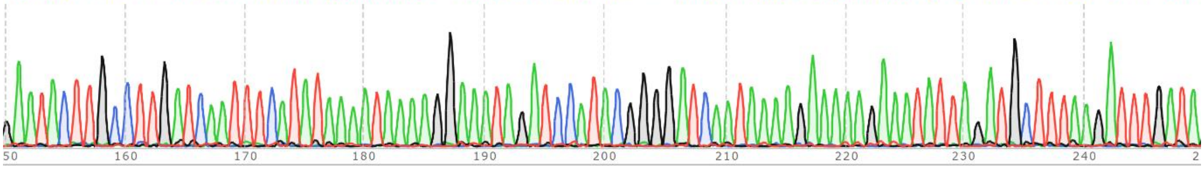

```
PCR_C3 : gaatacttgccttgatcaatttcatataaaataaaaggaaatagatccatacggggatcaataaaagaaaaagaaatattagatgctttaagatttgata : 200
r08_C3 : gaatacttgccttgatcaatttcatataaaataaaaggaaatagatccatacggggatcaataaaagaaaaagaaatattagatgctttaagatttgata : 200
         GAATACTTGCCTTGATCAATTTCATATAAAATAAAAGGAAATAGATCCATACGGGGATCAATAAAAGAAAAAGAAATATTAGATGCTTTAAGATTTGATA
```

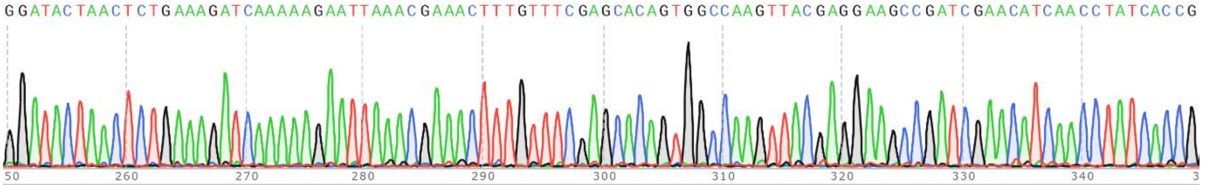


```
PCR_C3 : ggatactaactctgaaagatcaaaaagaattaaacgaaactttgtttcgagcacagtggccaagttacgaggaagccgatcgaacatcaacctatcaccg : 300
r08_C3 : ggatactaactctgaaagatcaaaaagaattaaacgaaactttgtttcgagcacagtggccaagttacgaggaagccgatcgaacatcaacctatcaccg : 300
         GGATACTAACTCTGAAAGATCAAAAAGAATTAAACGAAACTTTGTTTCGAGCACAGTGGCCAAGTTACGAGGAAGCCGATCGAACATCAACCTATCACCG
```

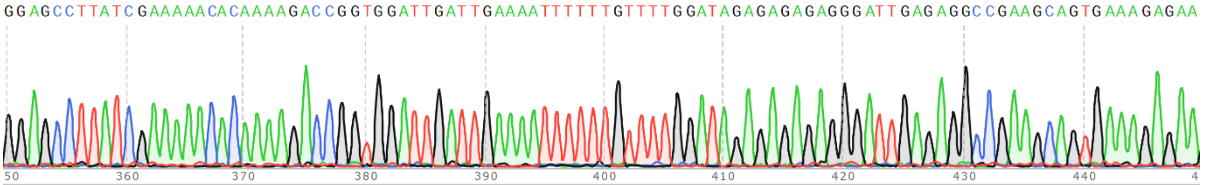


```
PCR_C3 : ggagccttatcgaaaaacacaaaagaccggtggattgattgaaaattttttgttttggatagagagagagggattgagaggccgaagcagtgaaagagaa : 400
r08_C3 : ggagccttatcgaaaaacacaaaagaccggtggattgattgaaaattttttgttttggatagagagagagggattgagaggccgaagcagtgaaagagaa : 400
         GGAGCCTTATCGAAAAACACAAAAGACCGGTGGATTGATTGAAAATTTTTTGTTTTGGATAGAGAGAGAGGGATTGAGAGGCCGAAGCAGTGAAAGAGAA
```

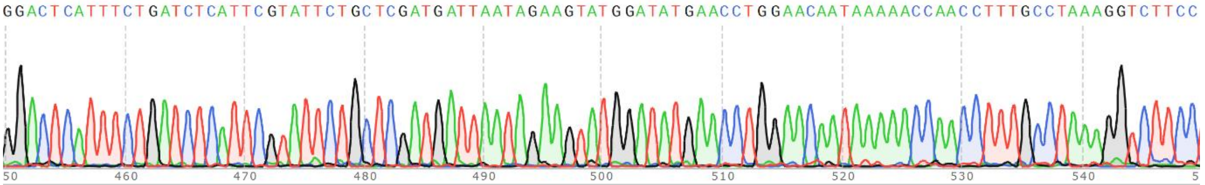


```
PCR_C3 : ggactcatttctgatctcattcgtattctgctcgatgattaatagaagtatggatatgaacctggaacaataaaaaccaacctttgcctaaaggtcttcc : 500
r08_C3 : ggactcatttctgatctcatccgtattctgctcgatgattaatagaagtatggatatgaacctggaacaataaaaaccaacctttgcctaaaggtcttcc : 500
         GGACTCATTTCTGATCTCAT CGTATTCTGCTCGATGATTAATAGAAGTATGGATATGAACCTGGAACAATAAAAACCAACCTTTGCCTAAAGGTCTTCC
```

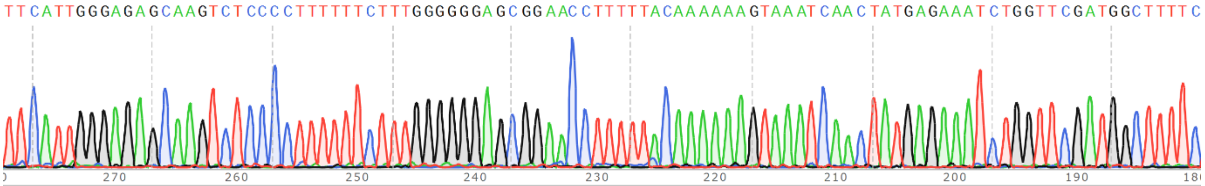


```
PCR_C3 : ttcattgggagagcaagtctccccttttttctttggggggagcggaacctttttacaaaaaagtaaatcaactatgagaaatctggttcgatggcttttc : 600
r08_C3 : ttcattgggagagcaagtctccccttttttctttggggggagcggaacctttttacaaaaaagtaaatcaactatgagaaatctggttcgatggcttttc : 600
         TTCATTGGGAGAGCAAGTCTCCCCTTTTTTCTTTGGGGGGAGCGGAACCTTTTTACAAAAAAGTAAATCAACTATGAGAAATCTGGTTCGATGGCTTTTC
```

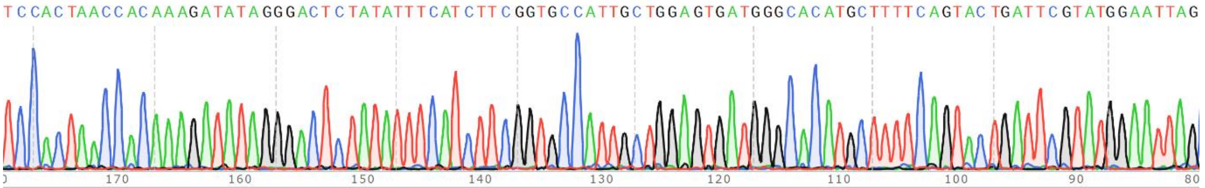


```
PCR_C3 : tccactaaccacaaagatatagggactctatatttcatcttcggtgccattgctggagtgatgggcacatgcttttcagtactgattcgtatggaattag : 700
r08_C3 : tccactaaccacaaagatatagggactctatatttcatcttcggtgccattgctggagtgatgggcacatgcttttcagtactgattcgtatggaattag : 700
         TCCACTAACCACAAAGATATAGGGACTCTATATTTCATCTTCGGTGCCATTGCTGGAGTGATGGGCACATGCTTTTCAGTACTGATTCGTATGGAATTAG
```

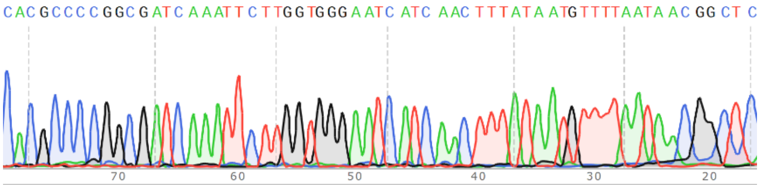


```
PCR_C3 : cacgccccggcgatcaaattcttggtgggaatcatcaactttataatgttttaataacggctc : 763
r08_C3 : cacgccccggcgatcaaattcttggtgggaatcatcaactttataatgttttaataacggctc : 763
         CACGCCCCGGCGATCAAATTCTTGGTGGGAATCATCAACTTTATAATGTTTTAATAACGGCTC
```

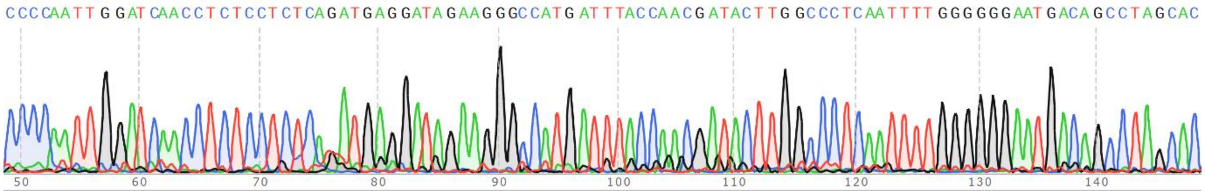

PCR_C4 : ccccaattggatcaacctctcctctcagatgaggatagaagggccatgatttaccaacgatacttggccctcaattttggggggaatgacagcctagcac : 100
r08_C4 : ccccaattggatcaacctctcctctcagatgaggatagaagggccatgatttaccaacgatacttggccctcaattttggggggaatgacagcctagcac : 100
CCCCAATTGGATCAACCTCTCCTCTCAGATGAGGATAGAAGGGCCATGATTTACCAACGATACTTGGCCCTCAATTTTGGGGGGAATGACAGCCTAGCAC

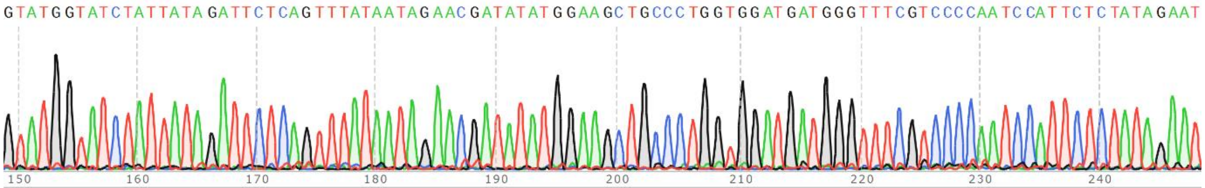

PCR_C4 : gtatggtatctattatagattctcagtttataatagaacgatatatggaagctgccctggtggatgatgggtttcgtccccaatccattctctatagaat : 200
r08_C4 : gtatggtatctattatagattctcagtttataatagaacgatatatggaagctgccctggtggatgatgggtttcgtccccaatccattctctatagaat : 200
GTATGGTATCTATTATAGATTCTCAGTTTATAATAGAACGATATATGGAAGCTGCCCTGGTGGATGATGGGTTTCGTCCCCAATCCATTCTCTATAGAAT

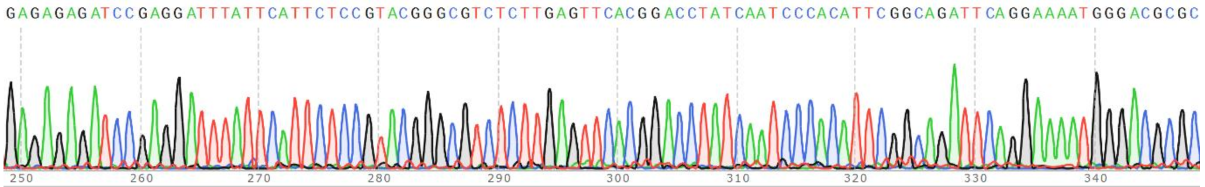

PCR_C4 : gagagagatccgaggatttattcattctccgtacgggcgtctcttgagttcacggacctatcaatcccacattcggcagattcaggaaaatgggacgcgc : 300
r08_C4 : gagagagatccgaggatttattcattctccgtacgggcgtctcttgagttcacggacctatcaatcccacattcggcagattcaggaaaatgggacgcgc : 300
GAGAGAGATCCGAGGATTTATTCATTCTCCGTACGGGCGTCTCTTGAGTTCACGGACCTATCAATCCCACATTCGGCAGATTCAGGAAAATGGGACGCGC

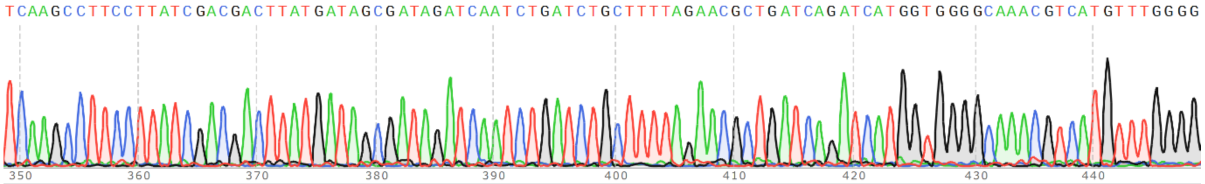

PCR_C4 : tcaagccttccttatcgacgacttatgatagcgatagatcaatctgatctgcttttagaacgctgatcagatcatggtggggcaaacgtcatgtttgggg : 400
r08_C4 : tcaagccttccttatcgacgacttatgatagcgatagatcaatctgatctgcttttagaacgctgatcagatcatggtggggcaaacgtcatgtttgggg : 400
TCAAGCCTTCCTTATCGACGACTTATGATAGCGATAGATCAATCTGATCTGCTTTTAGAACGCTGATCAGATCATGGTGGGGCAAACGTCATGTTTGGGG

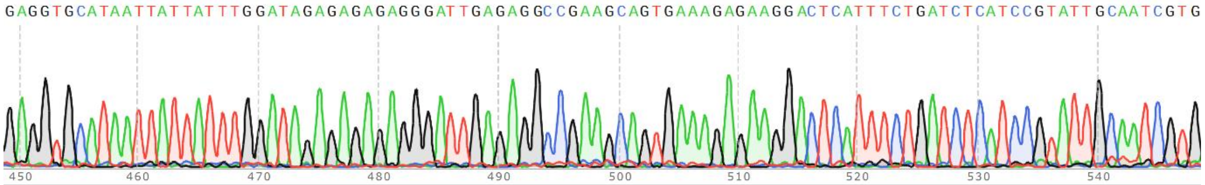

PCR_C4 : gaggtgcataattattatttggatagagagagagggattgagaggccgaagcagtgaaagagaaggactcatttctgatctcatccgtattgcaatcgtg : 500
r08_C4 : gaggtgcataattattatttggatagagagagagggattgagaggccgaagcagtgaaagagaaggactcatttctgatctcattcgtattgcaatcgtg : 500
GAGGTGCATAATTATTATTTGGATAGAGAGAGAGGGATTGAGAGGCCGAAGCAGTGAAAGAGAAGGACTCATTTCTGATCTCAT CGTATTGCAATCGTG

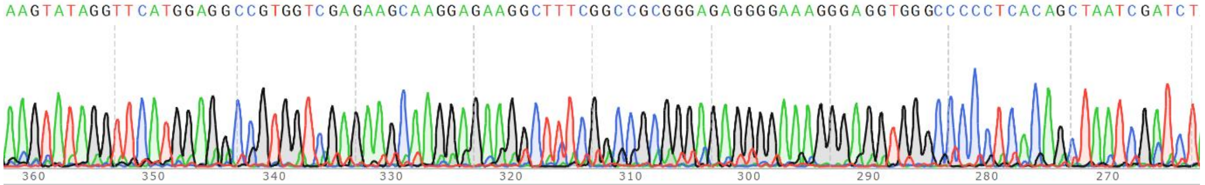

PCR_C4 : aagtataggttcatggaggccgtggtcgagaagcaaggagaaggctttcggccgcgggagaggggaaagggaggtgggccccctcacagctaatcgatct : 600
r08_C4 : aagtataggttcatggaggccgtggtcgagaagcaaggagaaggctttcggccgcgggagaggggaaagggaggtgggccccctcacagctaatcgatct : 600
AAGTATAGGTTCATGGAGGCCGTGGTCGAGAAGCAAGGAGAAGGCTTTCGGCCGCGGGAGAGGGGAAAGGGAGGTGGGCCCCCTCACAGCTAATCGATCT

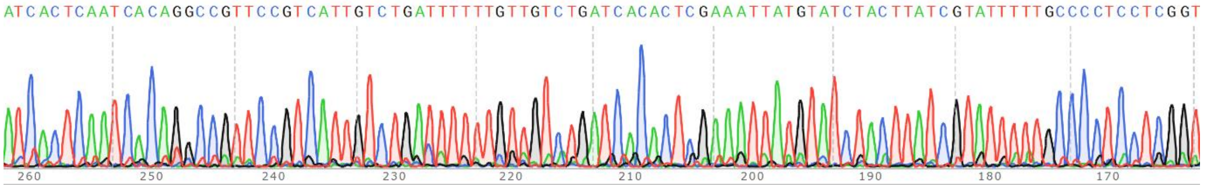

PCR_C4 : atcactcaatcacaggccgttccgtcattgtctgattttttgttgtctgatcacactcgaaattatgtatctacttatcgtatttttgcccctcctcggt : 700
r08_C4 : atcactcaatcacaggccgttccgtcattgtctgattttttgttgtctgatcacactcgaaattatgtatctacttatcgtatttttgcccctcctcggt : 700
ATCACTCAATCACAGGCCGTTCCGTCATTGTCTGATTTTTTGTTGTCTGATCACACTCGAAATTATGTATCTACTTATCGTATTTTTGCCCCTCCTCGGT

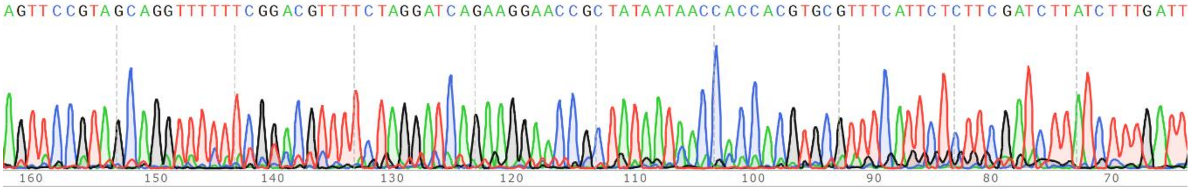

```
PCR_C4 : agttccgtagcaggttttttcggacgttttctaggatcagaaggaaccgctataataaccaccacgtgcgtttcattctcttcgatcttatctttgatt : 800
r08_C4 : agttccgtagcaggttttttcggacgttttctaggatcagaaggaaccgctataataaccaccacgtgcgtttcattctcttcgatcttatctttgatt : 799
         AGTTCCGTAGCAGGTTTTTTCGGACGTTTTCTAGGATCAG AAGGAACCGCTATAATAACCACCACGTGCGTTTCATTCTCTTCGATCTTATCTTTGATT
```

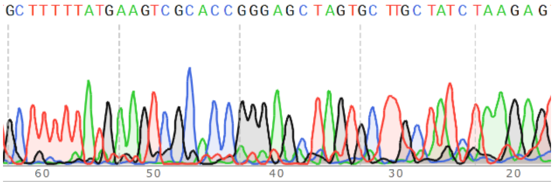

```
PCR_C4 : gctttttatgaagtcgcaccgggagctagtgcttgctatctaagag : 846
r08_C4 : gctttttatgaagtcgcaccgggagctagtgcttgctatctaagag : 845
         GCTTTTTATGAAGTCGCACCGGGAGCTAGTGCTTGCTATCTAAGAG
```

i

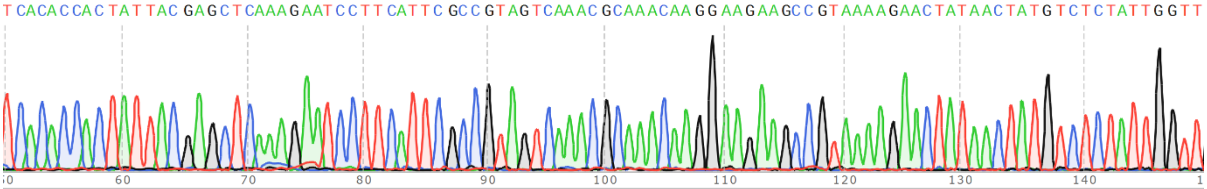

```
PCR_C1 : tcacaccactattacgagctcaaagaatccttcattcgccgtagtcaaacgcaaacaaggaagaagccgtaaaagaactataactatgtctctattggtt : 100
r09_C1 : tcacaccactattacgagctcaaagaatccttcattcgccgtagtcaaacgcaaacaaggaagaagccgtaaaagaactataactatgtctctattggtt : 100
         TCACACCACTATTACGAGCTCAAAGAATCCTTCATTCGCCGTAGTCAAACGCAAACAAGGAAGAAGCCGTAAAAGAACTATAACTATGTCTCTATTGGTT
```

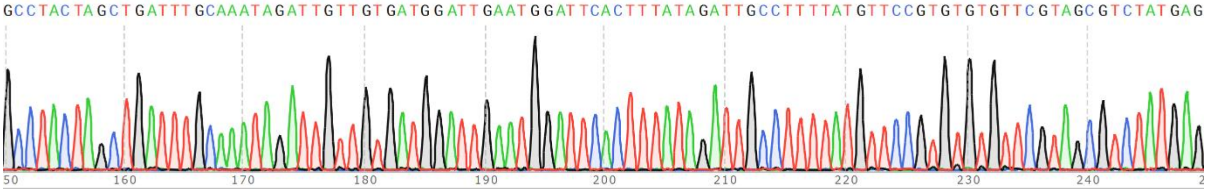

```
PCR_C1 : gcctactagctgatttgcaaatagattgttgtgatggattgaatggattcactttatagattgccttttatgttccgtgtgtgttcgtagcgtctatgag : 200
r09_C1 : gcctactagctgatttgcaaatagattgttgtgatggattgaatggattcactttatagattgccttttatgttccgtgtgtgttcgtagcgtctatgag : 200
         GCCTACTAGCTGATTTGCAAATAGATTGTTGTGATGGATTGAATGGATTCACTTTATAGATTGCCTTTTATGTTCCGTGTGTGTTCGTAGCGTCTATGAG
```

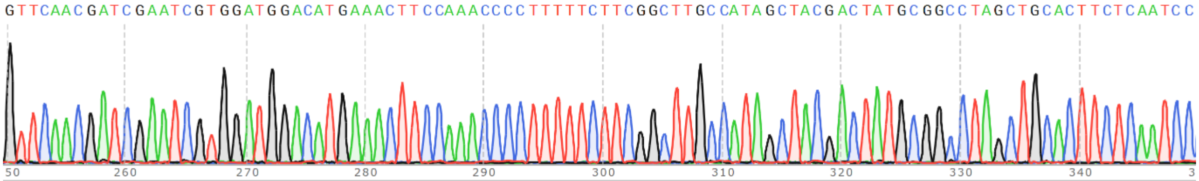

```
PCR_C1 : gttcaacgatcgaatcgtggatggacatgaaacttccaaacccctttttcttcggcttgccatagctacgactatgcggcctagctgcacttctcaatcc : 300
r09_C1 : gttcaacgatcgaatcgtggatggacatgaaacttccaaacccctttttcttcggcttgccatagctacgactatgcggcctagctgcacttctcaatcc : 300
         GTTCAACGATCGAATCGTGGATGGACATGAAACTTCCAAACCCCTTTTTCTTCGGCTTGCCATAGCTACGACTATGCGGCCTAGCTGCACTTCTCAATCC
```

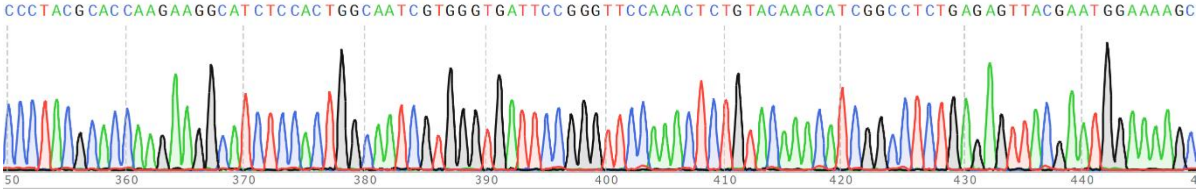

```
PCR_C1 : ccctacgcaccaagaaggcatctccactggcaatcgtgggtgattccgggttccaaactctgtacaaacatcggcctctgagagttacgaatggaaaagc : 400
r09_C1 : ccctacgcaccaagaaggcatctccactggcaatcgtgggtgattccgggttccaaactctgtacaaacatcggcctctgagagttacgaatggaaaagc : 400
         CCCTACGCACCAAGAAGGCATCTCCACTGGCAATCGTGGGTGATTCCGGGTTCCAAACTCTGTACAAACATCGGCCTCTGAGAGTTACGAATGGAAAAGC
```

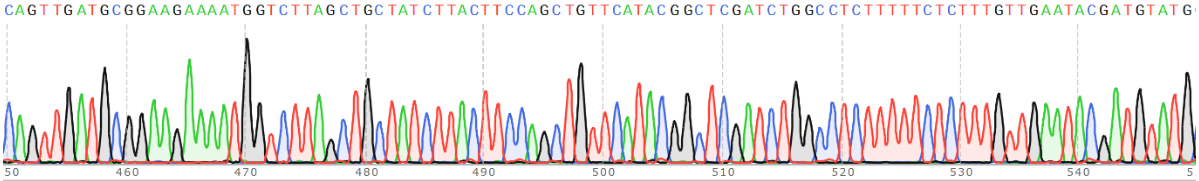


```
PCR_C1 : cagttgatgcggaagaaaatggtcttagctgctatcttacttccagctgttcatacggctcgatctggcctctttttctctttgttgaatacgatgtatg : 500
r09_C1 : cagttgatgcggaagaaaatggtcttagctgctatcttacttccagctgttcatacggctcgatctggcctctttttctctttgttgaatacgatgtatg : 500
         CAGTTGATGCGGAAGAAAATGGTCTTAGCTGCTATCTTACTTCCAGCTGTTCATACGGCTCGATCTGGCCTCTTTTTCTCTTTGTTGAATACGATGTATG
```

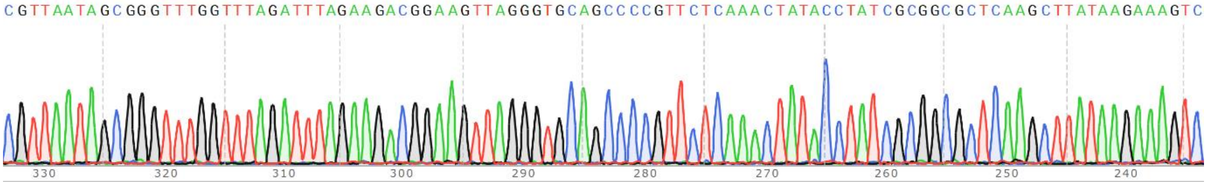


```
PCR_C1 : cgttaatagcgggtttggtttagatttagaagacggaagttagggtgcagccccgttctcaaactatacctatcgcggcgctcaagcttataagaaagtc : 600
r09_C1 : cgttaatagcgggtttggtttagatttagaagacggaagttagggtgcagccccgttctcaaactatacctatcgcggcgctcaagcttataagaaagtc : 600
         CGTTAATAGCGGGTTTGGTTTAGATTTAGAAGACGGAAGTTAGGGTGCAGCCCCGTTCTCAAACTATACCTATCGCGGCGCTCAAGCTTATAAGAAAGTC
```

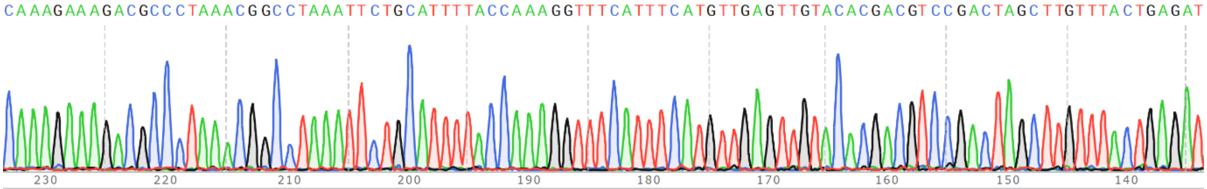


```
PCR_C1 : caaagaaagacgccctaaacggcctaaattctgcattttaccaaaggtttcatttcatgttgagttgtacacgacgtccgactagcttgtttactgagat : 700
r09_C1 : caaagaaagacgccctaaacggcctaaattctgcattttaccaaaggtttcatttcatgttgagttgtacacgacgtccgactagcttgtttactgagat : 700
         CAAAGAAAGACGCCCTAAACGGCCTAAATTCTGCATTTTACCAAAGGTTTCATTTCATGTTGAGTTGTACACGACGTCCGACTAGCTTGTTTACTGAGAT
```

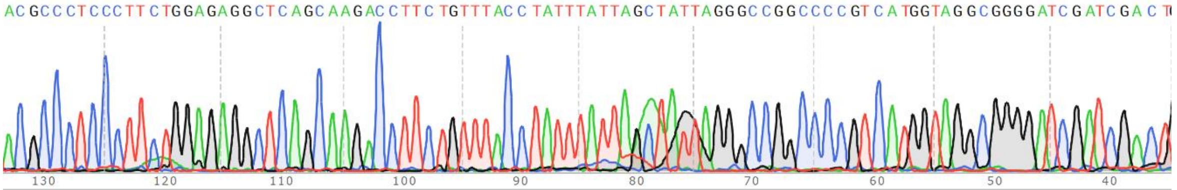


```
PCR_C1 : acgccctcccttctggagaggctcagcaagaccttctgtttacctatttattagctattagggccggccccgtcatggtaggcggggatcgatcgact : 798
r09_C1 : acgccctcccttctggagaggctcagcaagaccttctgtttacctatttattagctattagggccggccccgtcatggtaggcggggatcgatcgact : 798
         ACGCCCTCCCTTCTGGAGAGGCTCAGCAAGACCTTCTGTTTACCTATTTATTAGCTATTAGGGCCGGCCCCGTCATGGTAGGCGGGGATCGATCGACT
```

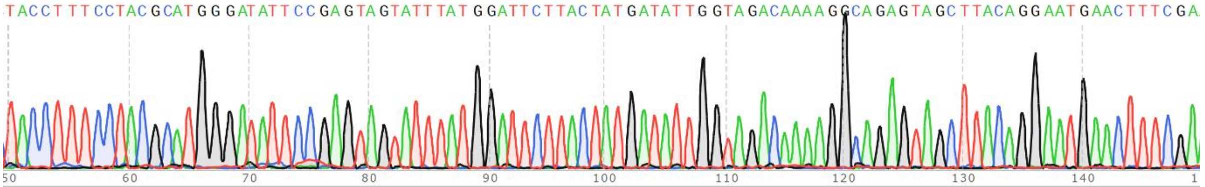


```
PCR_C2 : tacctttcctacgcatgggatattccgagtagtatttatggattcttactatgatattggtagacaaaaggcagagtagcttacaggaatgaactttcga : 100
r09_C2 : tacctttcctacgcatgggatattccgagtagtatttatggattcttactatgatattggtagacaaaaggcagagtagcttacaggaatgaactttcga : 100
         TACCTTTCCTACGCATGGGATATTCCGAGTAGTATTTATGGATTCTTACTATGATATTGGTAGACAAAAGGCAGAGTAGCTTACAGGAATGAACTTTCGA
```

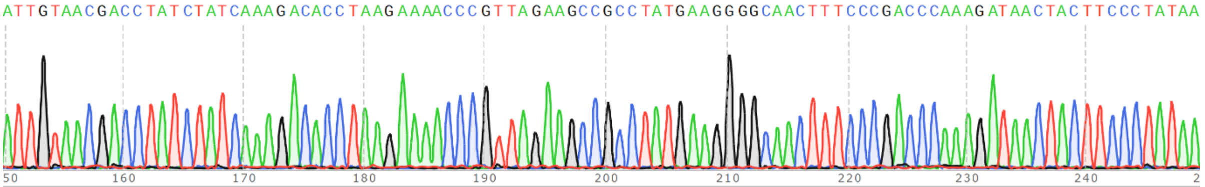


```
PCR_C2 : attgtaacgacctatctatcaaagacacctaagaaaacccgttagaagccgcctatgaaggggcaactttcccgacccaaagataactacttccctataa : 200
r09_C2 : attgtaacgacctatctatcaaagacacctaagaaaacccgttagaagccgcctatgaaggggcaactttcccgacccaaagataactacttccctataa : 200
         ATTGTAACGACCTATCTATCAAAGACACCTAAGAAAACCCGTTAGAAGCCGCCTATGAAGGGGCAACTTTCCCGACCCAAAGATAACTACTTCCCTATAA
```

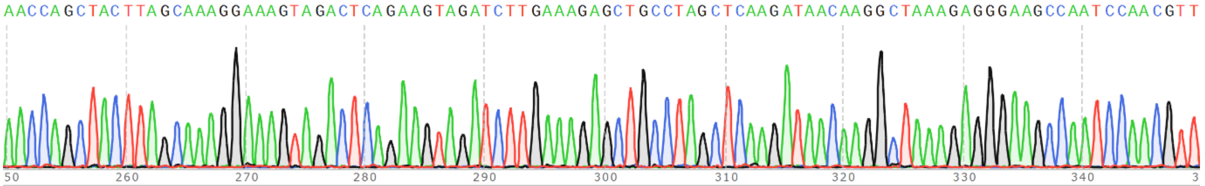


```
PCR_C2 : aaccagctacttagcaaaggaaagtagactcagaagtagatcttgaaagagctgcctagctcaagataacaaggctaaagagggaagccaatccaacgtt : 300
r09_C2 : aaccagctacttagcaaaggaaagtagactcagaagtagatcttgaaagagctgcctagctcaagataacaaggctaaagagggaagccaatccaacgtt : 300
         AACCAGCTACTTAGCAAAGGAAAGTAGACTCAGAAGTAGATCTTGAAAGAGCTGCCTAGCTCAAGATAACAAGGCTAAAGAGGGAAGCCAATCCAACGTT
```

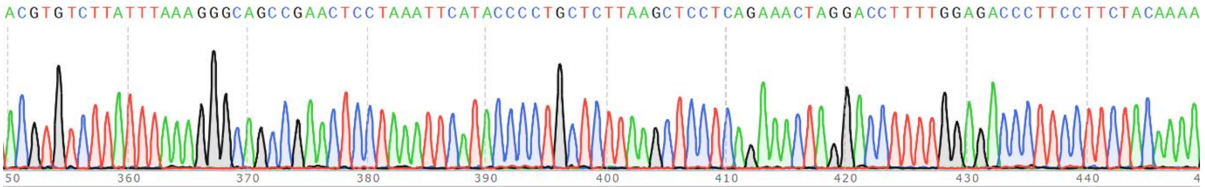


```
PCR_C2 : acgtgtcttatttaaagggcagccgaactcctaaattcataccccctgctcttaagctcctcagaaactaggaccttttggagacccttccttctacaaaa : 400
r09_C2 : acgtgtcttatttaaagggcagccgaactcctaaattcataccccctgctcttaagctcctcagaaactaggaccttttggagacccttccttctacaaaa : 400
         ACGTGTCTTATTTAAAGGGCAGCCGAACTCCTAAATTCATACCCCTGCTCTTAAGCTCCTCAGAAACTAGGACCTTTTGGAGACCCTTCCTTCTACAAAA
```

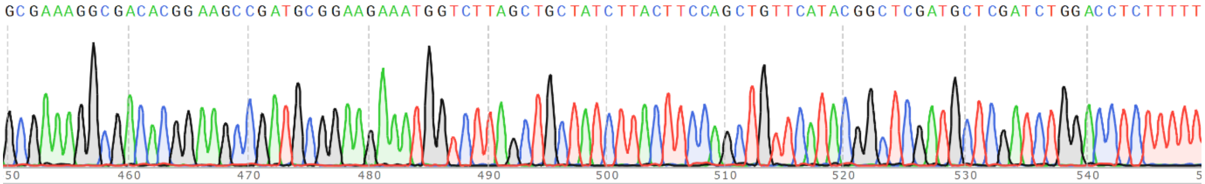


```
PCR_C2 : gcgaaaggcgacacggaagccgatgcggaagaaatggtcttagctgctatcttacttccagctgttcatacggctcgatgctcgatctggacctcttttt : 500
r09_C2 : gcgaaaggcgacacggaagccgatgcggaagaaatggtcttagctgctatcttacttccagctgttcatacggctcgatgctcgatctggacctcttttt : 500
         GCGAAAGGCGACACGGAAGCCGATGCGGAAGAAATGGTCTTAGCTGCTATCTTACTTCCAGCTGTTCATACGGCTCGATGCTCGATCTGGACCTCTTTTT
```

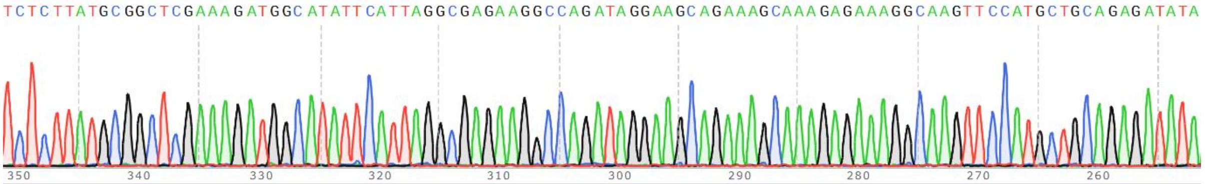


```
PCR_C2 : tctcttatgcggctcgaaagatggcatattcattaggcgagaaggccagataggaagcagaaagcaaagagaaaggcaagttccatgctgcagagatata : 600
r09_C2 : tctcttatgcggctcgaaagatggcatattcattaggcgagaaggccagataggaagcagaaagcaaagagaaaggcaagttccatgctgcagagatata : 600
         TCTCTTATGCGGCTCGAAAGATGGCATATTCATTAGGCGAGAAGGCCAGATAGGAAGCAGAAAGCAAAGAGAAAGGCAAGTTCCATGCTGCAGAGATATA
```

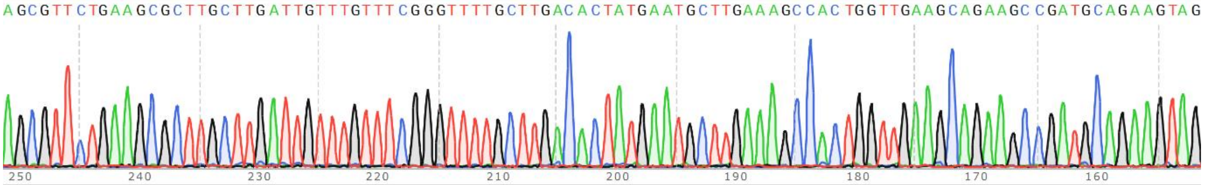


```
PCR_C2 : agcgttctgaagcgcttgcttgattgtttgtttcgggttttgcttgacactatgaatgcttgaaagccactggttgaagcagaagccgatgcagaagtag : 700
r09_C2 : agcgttctgaagcgcttgcttgattgtttgtttcgggttttgcttgacactatgaatgcttgaaagccactggttgaagcagaagccgatgcagaagtag : 700
         AGCGTTCTGAAGCGCTTGCTTGATTGTTTGTTTCGGGTTTTGCTTGACACTATGAATGCTTGAAAGCCACTGGTTGAAGCAGAAGCCGATGCAGAAGTAG
```

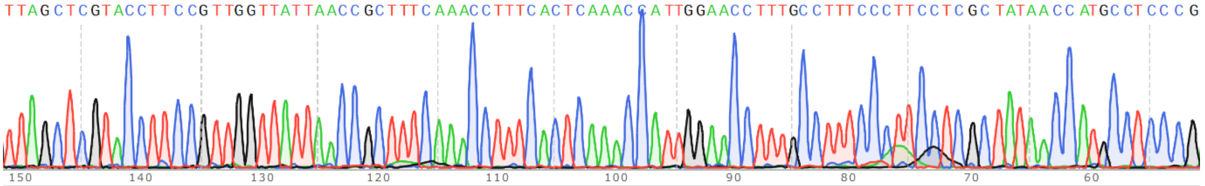


```
PCR_C2 : ttagctcgtaccttccgttggttattaaccgctttcaaacctttcactcaaaccattggaacctttgcctttcccttcctcgctataaccatgcctcccg : 800
r09_C2 : ttagctcgtaccttccgttggttattaaccgctttcaaacctttcactcaaaccattggaacctttgcctttcccttcctcgctataaccatgcctcccg : 800
         TTAGCTCGTACCTTCCGTTGGTTATTAACCGCTTTCAAACCTTTCACTCAAACCATTGGAACCTTTGCCTTTCCCTTCCTCGCTATAACCATGCCTCCCG
```

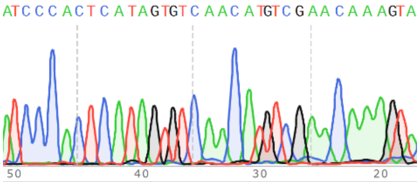


```
PCR_C2 : atcccactcatagtgtcaacatgtcgaacaaagta : 835
r09_C2 : atcccactcatagtgtcaacatgtcgaacaaagta : 835
         ATCCCACTCATAGTGTCAACATGTCGAACAAAGTA
```

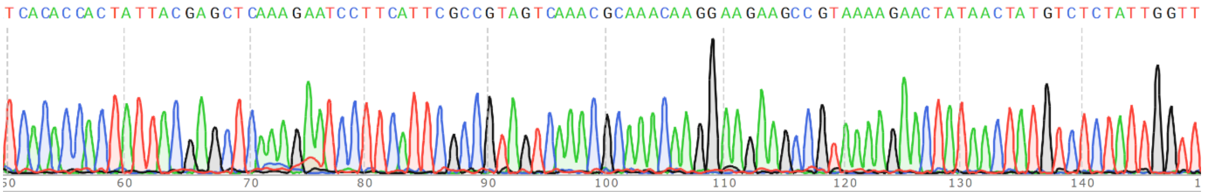


```
PCR_C3 : tcacaccactattacgagctcaaagaatccttcattcgccgtagtcaaacgcaaacaaggaagaagccgtaaaagaactataactatgtctctattggtt : 100
r09_C3 : tcacaccactattacgagctcaaagaatccttcattcgccgtagtcaaacgcaaacaaggaagaagccgtaaaagaactataactatgtctctattggtt : 100
         TCACACCACTATTACGAGCTCAAAGAATCCTTCATTCGCCGTAGTCAAACGCAAACAAGGAAGAAGCCGTAAAAGAACTATAACTATGTCTCTATTGGTT
```

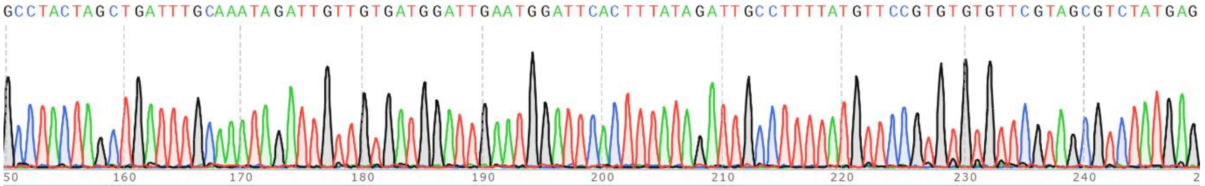

PCR_C3 : gcctactagctgatttgcaaatagattgttgtgatggattgaatggattcactttatagattgccttttatgttccgtgtgtgttcgtagcgtctatgag : 200
r09_C3 : gcctactagctgatttgcaaatagattgttgtgatggattgaatggattcactttatagattgccttttatgttccgtgtgtgttcgtagcgtctatgag : 200
GCCTACTAGCTGATTTGCAAATAGATTGTTGTGATGGATTGAATGGATTCACTTTATAGATTGCCTTTTATGTTCCGTGTGTGTTCGTAGCGTCTATGAG

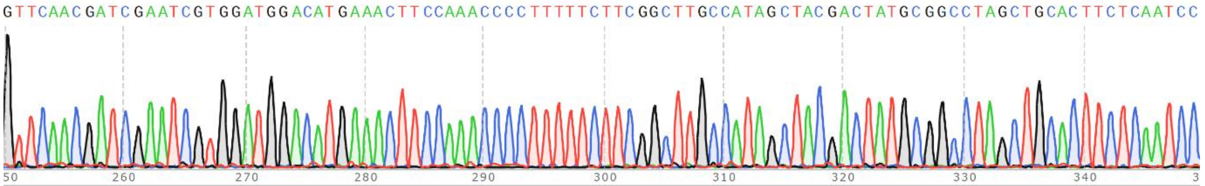

PCR_C3 : gttcaacgatcgaatcgtggatggacatgaaacttccaaacccctttttcttcggcttgccatagctacgactatgcggcctagctgcacttctcaatcc : 300
r09_C3 : gttcaacgatcgaatcgtggatggacatgaaacttccaaacccctttttcttcggcttgccatagctacgactatgcggcctagctgcacttctcaatcc : 300
GTTCAACGATCGAATCGTGGATGGACATGAAACTTCCAAACCCCTTTTTCTTCGGCTTGCCATAGCTACGACTATGCGGCCTAGCTGCACTTCTCAATCC

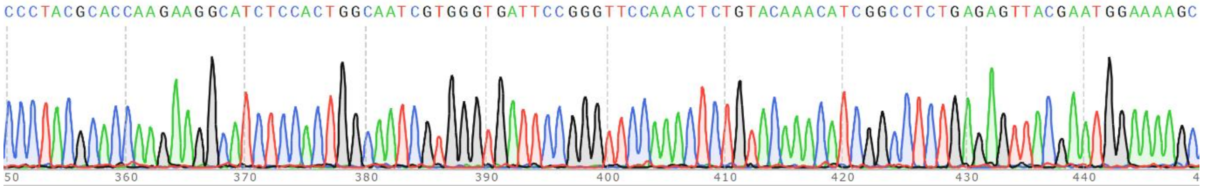

PCR_C3 : ccctacgcaccaagaaggcatctccactggcaatcgtgggtgattccgggttccaaactctgtacaaacatcggcctctgagagttacgaatggaaaagc : 400
r09_C3 : ccctacgcaccaagaaggcatctccactggcaatcgtgggtgattccgggttccaaactctgtacaaacatcggcctctgagagttacgaatggaaaagc : 400
CCCTACGCACCAAGAAGGCATCTCCACTGGCAATCGTGGGTGATTCCGGGTTCCAAACTCTGTACAAACATCGGCCTCTGAGAGTTACGAATGGAAAAGC

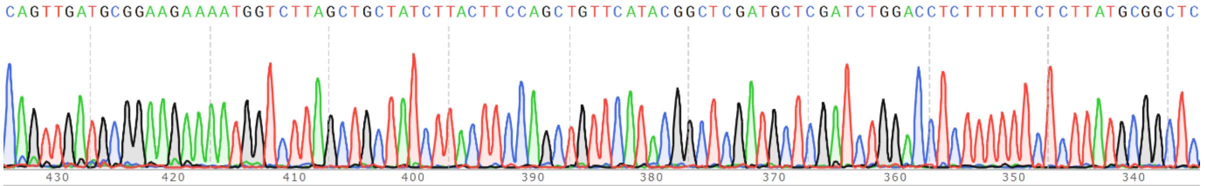

PCR_C3 : cagttgatgcggaagaaaatggtcttagctgctatcttacttccagctgttcatacggctcgatgctcgatctggacctcttttttctcttatgcggctc : 500
r09_C3 : cagttgatgcggaagaaaatggtcttagctgctatcttacttccagctgttcatacggctcgatgctcgatctggacctcttttttctcttatgcggctc : 500
CAGTTGATGCGGAAGAAAATGGTCTTAGCTGCTATCTTACTTCCAGCTGTTCATACGGCTCGATGCTCGATCTGGACCTCTTTTTTCTCTTATGCGGCTC

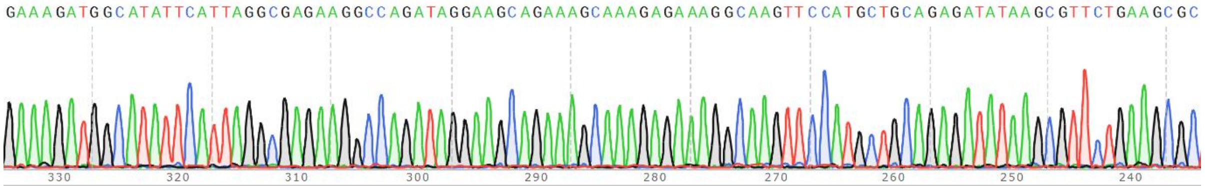

PCR_C3 : gaaagatggcatattcattaggcgagaaggccagataggaagcagaaagcaaagagaaaggcaagttccatgctgcagagatataagcgttctgaagcgc : 600
r09_C3 : gaaagatggcatattcattaggcgagaaggccagataggaagcagaaagcaaagagaaaggcaagttccatgctgcagagatataagcgttctgaagcgc : 600
GAAAGATGGCATATTCATTAGGCGAGAAGGCCAGATAGGAAGCAGAAAGCAAAGAGAAAGGCAAGTTCCATGCTGCAGAGATATAAGCGTTCTGAAGCGC

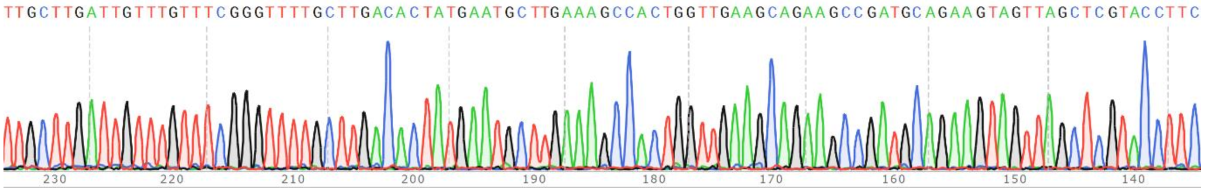

PCR_C3 : ttgcttgattgtttgtttcgggttttgcttgacactatgaatgcttgaaagccactggttgaagcagaagccgatgcagaagtagttagctcgtaccttc : 700
r09_C3 : ttgcttgattgtttgtttcgggttttgcttgacactatgaatgcttgaaagccactggttgaagcagaagccgatgcagaagtagttagctcgtaccttc : 700
TTGCTTGATTGTTTGTTTCGGGTTTTGCTTGACACTATGAATGCTTGAAAGCCACTGGTTGAAGCAGAAGCCGATGCAGAAGTAGTTAGCTCGTACCTTC

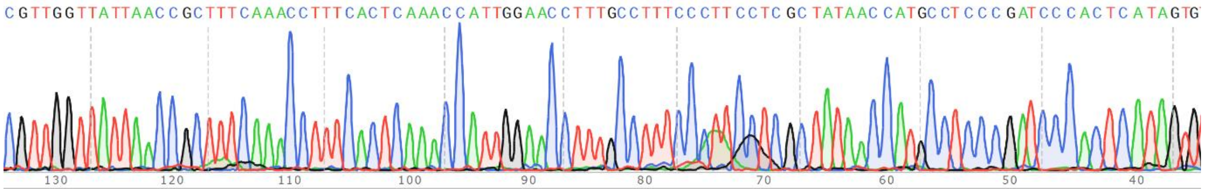

PCR_C3 : cgttggttattaaccgctttcaaacctttcactcaaaccattggaacctttgcctttcccttcctcgctataaccatgcctcccgatcccactcatagtg : 800
r09_C3 : cgttggttattaaccgctttcaaacctttcactcaaaccattggaacctttgcctttcccttcctcgctataaccatgcctcccgatcccactcatagtg : 800

CGTTGGTTATTAACCGCTTTCAAACCTTTCACTCAAACCATTGGAACCTTTGCCTTTCCCTTCCTCGCTATAACCATGCCTCCCGATCCCACTCATAGTG

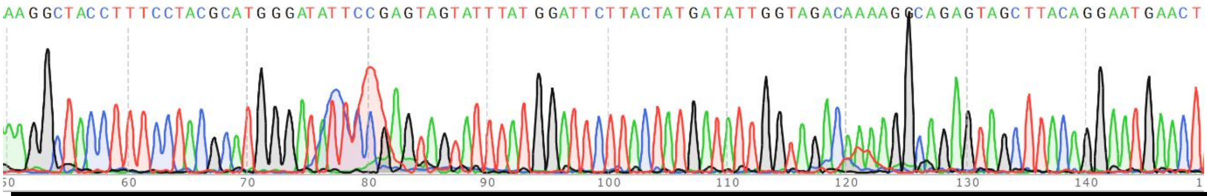

PCR_C4 : aaggctacctttcctacgcatgggatattccgagtagtatttatggattcttactatgatattggtagacaaaaggcagagtagcttacaggaatgaact : 100
r09_C4 : aaggctacctttcctacgcatgggatattccgagtagtatttatggattcttactatgatattggtagacaaaaggcagagtagcttacaggaatgaact : 100
AAGGCTACCTTTCCTACGCATGGGATATTCCGAGTAGTATTTATGGATTCTTACTATGATATTGGTAGACAAAAGGCAGAGTAGCTTACAGGAATGAACT

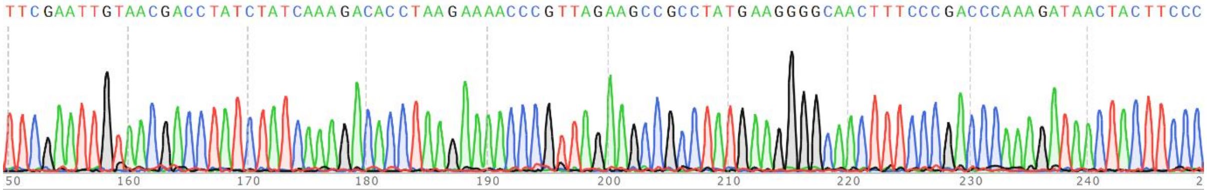

PCR_C4 : ttcgaattgtaacgacctatctatcaaagacacctaagaaaacccgttagaagccgcctatgaaggggcaactttcccgacccaaagataactacttccc : 200
r09_C4 : ttcgaattgtaacgacctatctatcaaagacacctaagaaaacccgttagaagccgcctatgaaggggcaactttcccgacccaaagataactacttccc : 200
TTCGAATTGTAACGACCTATCTATCAAAGACACCTAAGAAAACCCGTTAGAAGCCGCCTATGAAGGGGCAACTTTCCCGACCCAAAGATAACTACTTCCC

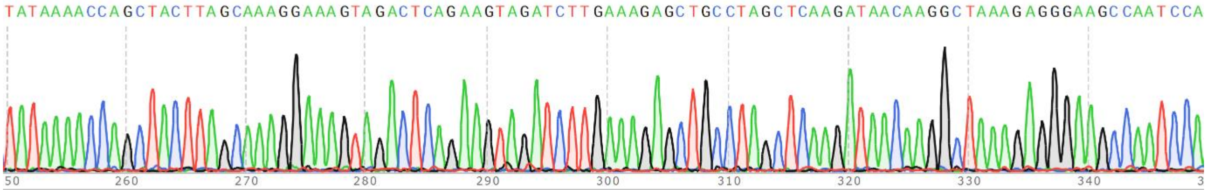

PCR_C4 : tataaaaccagctacttagcaaaggaaagtagactcagaagtagatcttgaaagagctgcctagctcaagataacaaggctaaagagggaagccaatcca : 300
r09_C4 : tataaaaccagctacttagcaaaggaaagtagactcagaagtagatcttgaaagagctgcctagctcaagataacaaggctaaagagggaagccaatcca : 300
TATAAAACCAGCTACTTAGCAAAGGAAAGTAGACTCAGAAGTAGATCTTGAAAGAGCTGCCTAGCTCAAGATAACAAGGCTAAAGAGGGAAGCCAATCCA

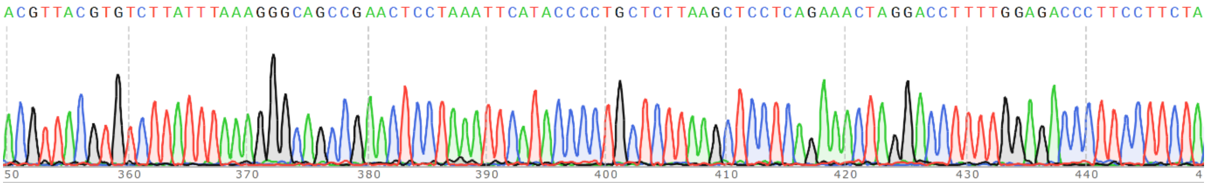

PCR_C4 : acgttacgtgtcttatttaaagggcagccgaactcctaaattcatacccctgctcttaagctcctcagaaactaggaccttttggagacccttccttcta : 400
r09_C4 : acgttacgtgtcttatttaaagggcagccgaactcctaaattcatacccctgctcttaagctcctcagaaactaggaccttttggagacccttccttcta : 400
ACGTTACGTGTCTTATTTAAAGGGCAGCCGAACTCCTAAATTCATACCCCTGCTCTTAAGCTCCTCAGAAACTAGGACCTTTTGGAGACCCTTCCTTCTA

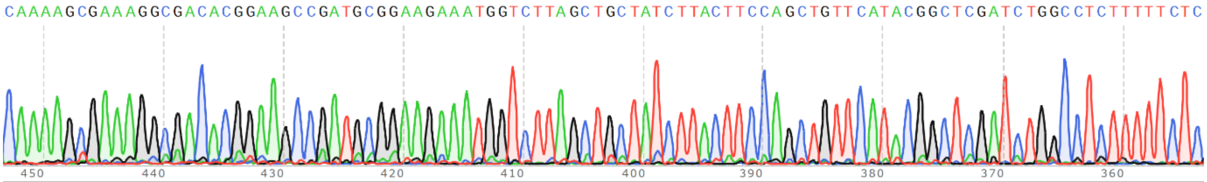

PCR_C4 : caaaagcgaaaggcgacacggaagccgatgcggaagaaatggtcttagctgctatcttacttccagctgttcatacggctcgatctggcctctttttctc : 500
r09_C4 : caaaagcgaaaggcgacacggaagccgatgcggaagaaatggtcttagctgctatcttacttccagctgttcatacggctcgatctggcctctttttctc : 500
CAAAAGCGAAAGGCGACACGGAAGCCGATGCGGAAGAAATGGTCTTAGCTGCTATCTTACTTCCAGCTGTTCATACGGCTCGATCTGGCCTCTTTTTCTC

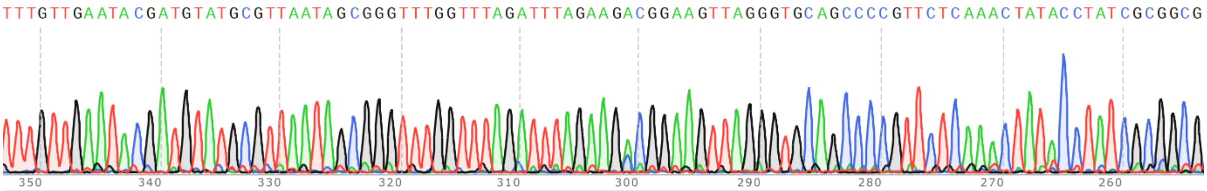

PCR_C4 : tttgttgaatacgatgtatgcgttaatagcgggtttggtttagatttagaagacggaagttagggtgcagccccgttctcaaactatacctatcgcggcg : 600
r09_C4 : tttgttgaatacgatgtatgcgttaatagcgggtttggtttagatttagaagacggaagttagggtgcagccccgttctcaaactatacctatcgcggcg : 600
TTTGTTGAATACGATGTATGCGTTAATAGCGGGTTTGGTTTAGATTTAGAAGACGGAAGTTAGGGTGCAGCCCCGTTCTCAAACTATACCTATCGCGGCG

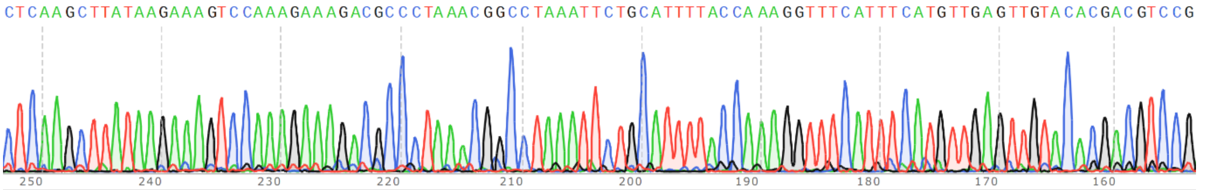

PCR_C4 : ctcaagcttataagaaagtccaaagaaagacgccctaaacggcctaaattctgcattttaccaaaggtttcatttcatgttgagttgtacacgacgtccg : 700
r09_C4 : ctcaagcttataagaaagtccaaagaaagacgccctaaacggcctaaattctgcattttaccaaaggtttcatttcatgttgagttgtacacgacgtccg : 700
CTCAAGCTTATAAGAAAGTCCAAAGAAAGACGCCCTAAACGGCCTAAATTCTGCATTTTACCAAAGGTTTCATTTCATGTTGAGTTGTACACGACGTCCG

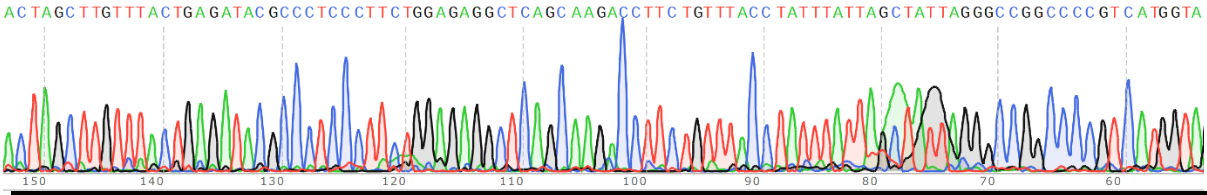


```
PCR_C4 : actagcttgtttactgagatacgccctcccttctggagaggctcagcaagaccttctgtttacctatttattagctattagggccggccccgtcatggta : 800
r09_C4 : actagcttgtttactgagatacgccctcccttctggagaggctcagcaagaccttctgtttacctatttattagctattagggccggccccgtcatggta : 800
         ACTAGCTTGTTTACTGAGATACGCCCTCCCTTCTGGAGAGGCTCAGCAAGACCTTCTGTTTACCTATTTATTAGCTATTAGGGCCGGCCCCGTCATGGTA
```

Figure S8. Alignments of the long PacBio reads to the MTPT fragments in the mitogenome of S. miltiorrhiza. Panels a–p show the alignments of long PacBio reads to the ten MTPT fragments in MC1 (sami-mtpt-001 to sami-mtpt-010) and six MTPT fragments in MC2 (sami-mtpt-011 to sami-mtpt-016). The MTPT regions are indicated with the red lines having arrow heads at each end. The 2000 bp-long flanking sequences are indicated with a red line without arrow heads.

a

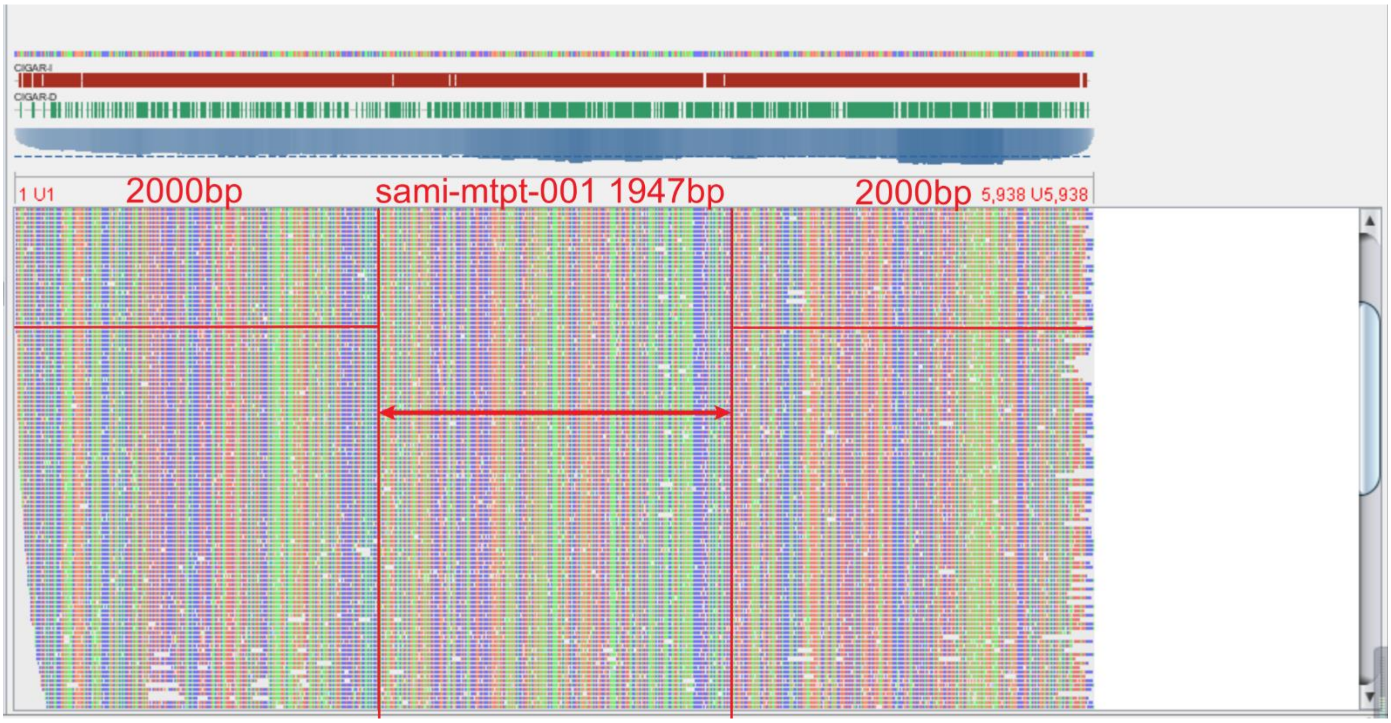

b

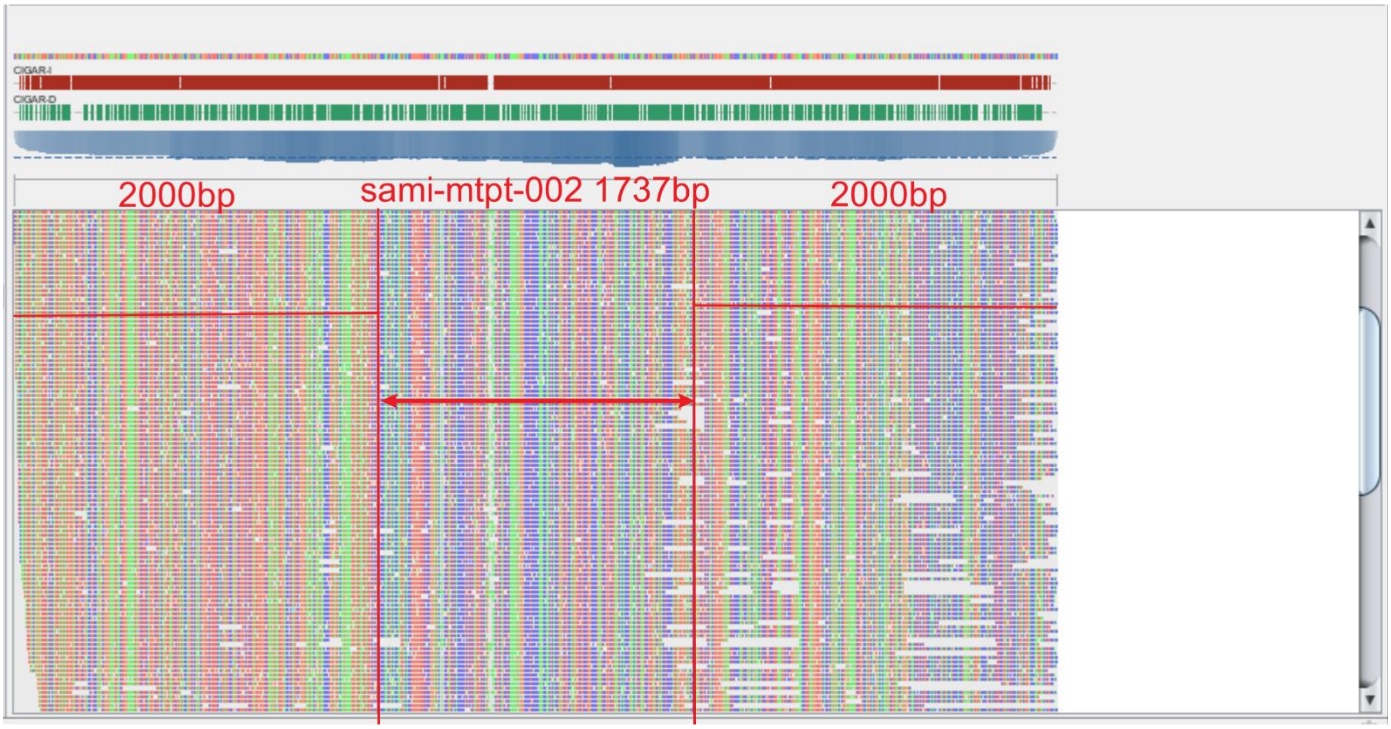

c

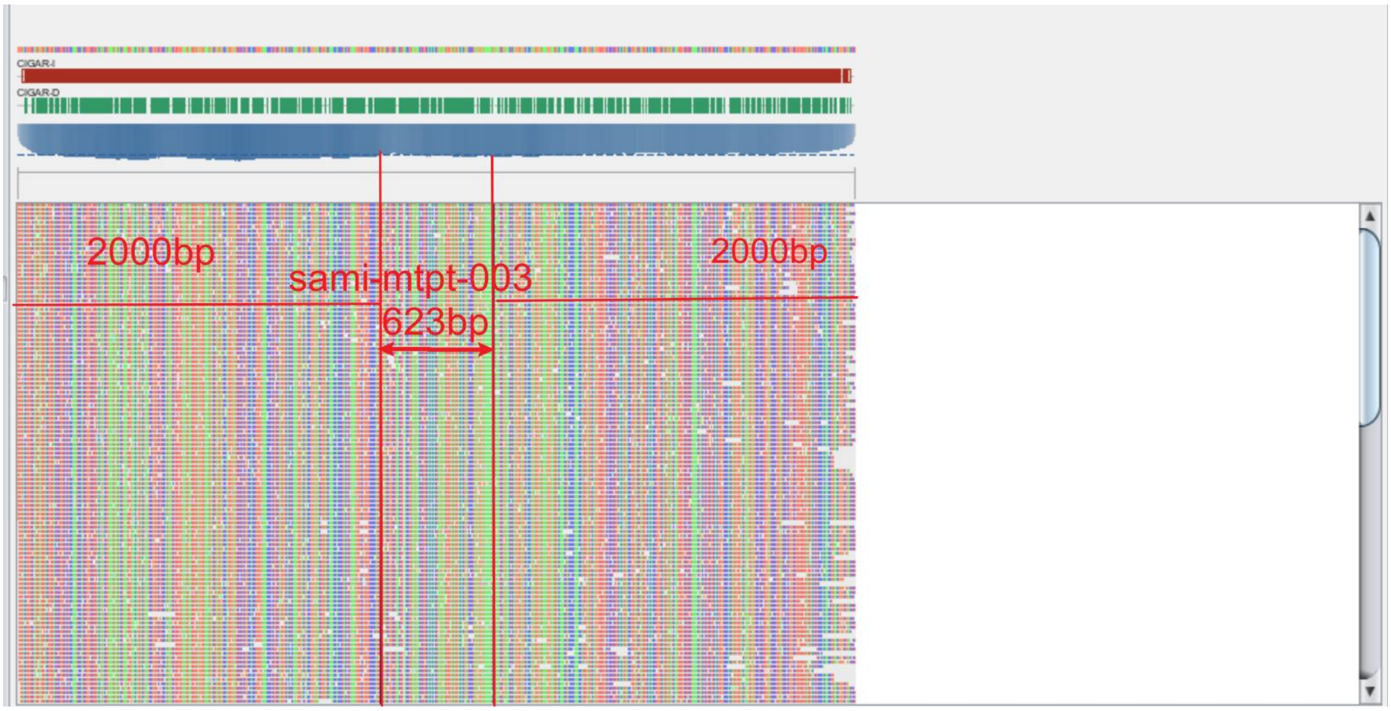

d

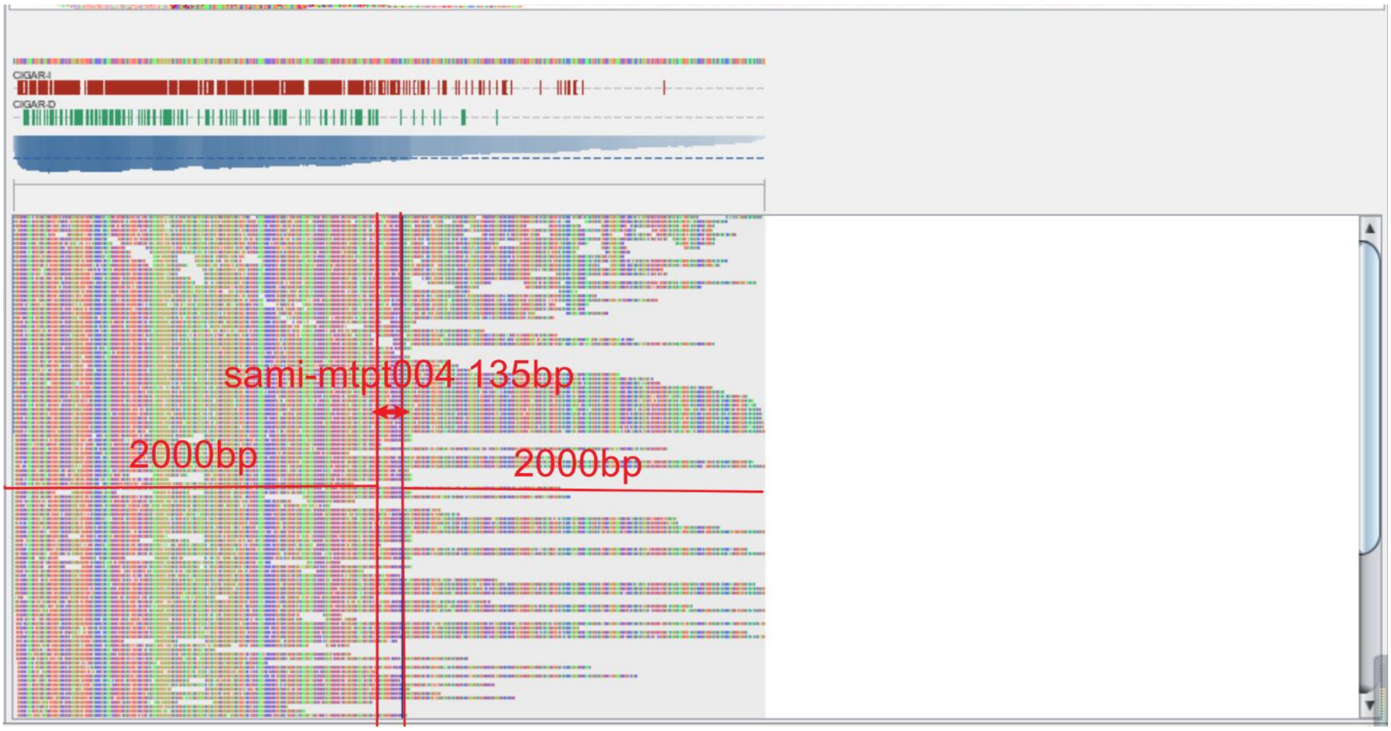

e

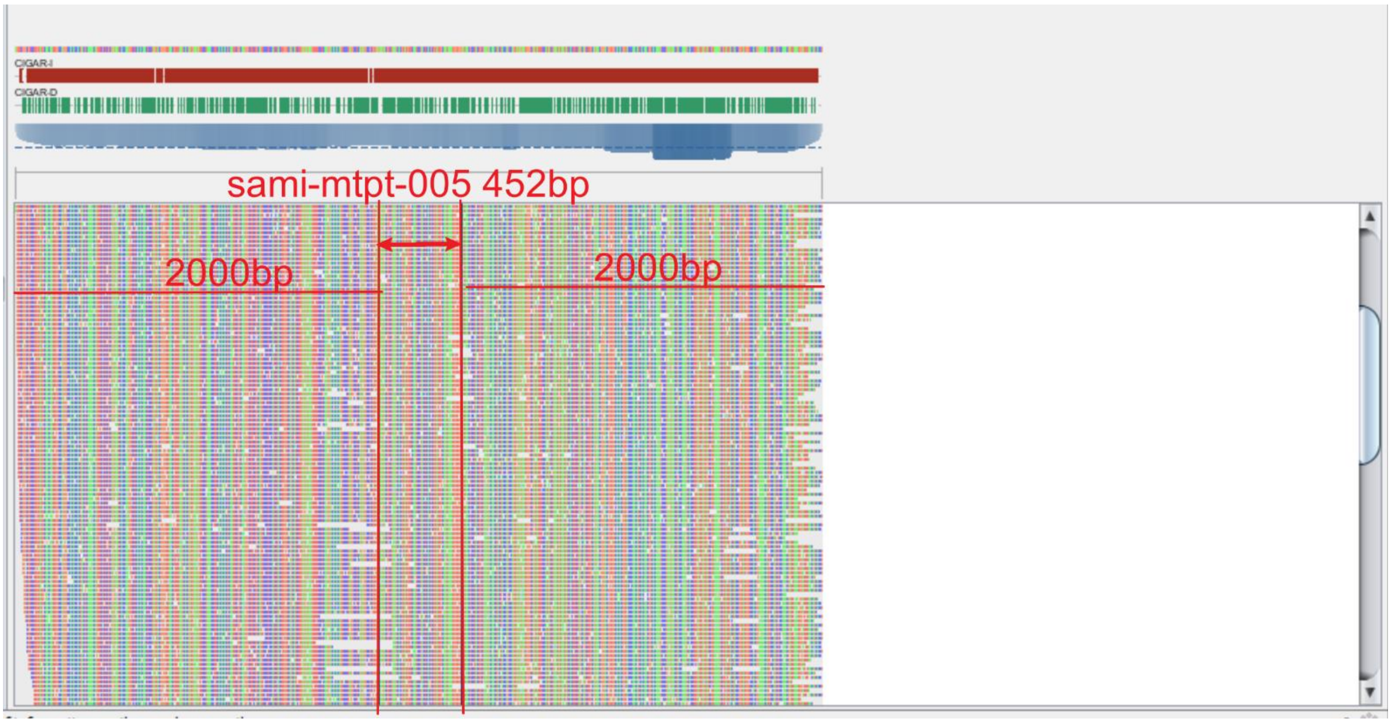

f

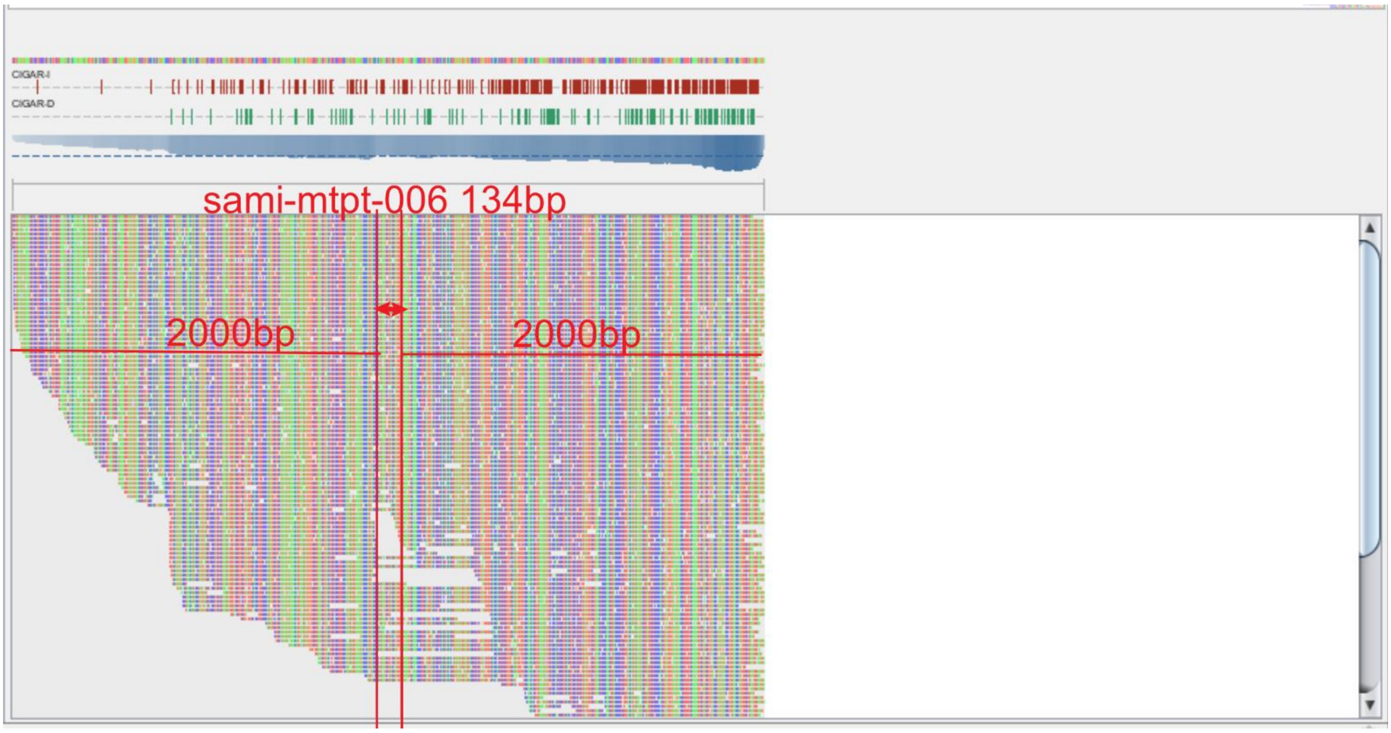

g

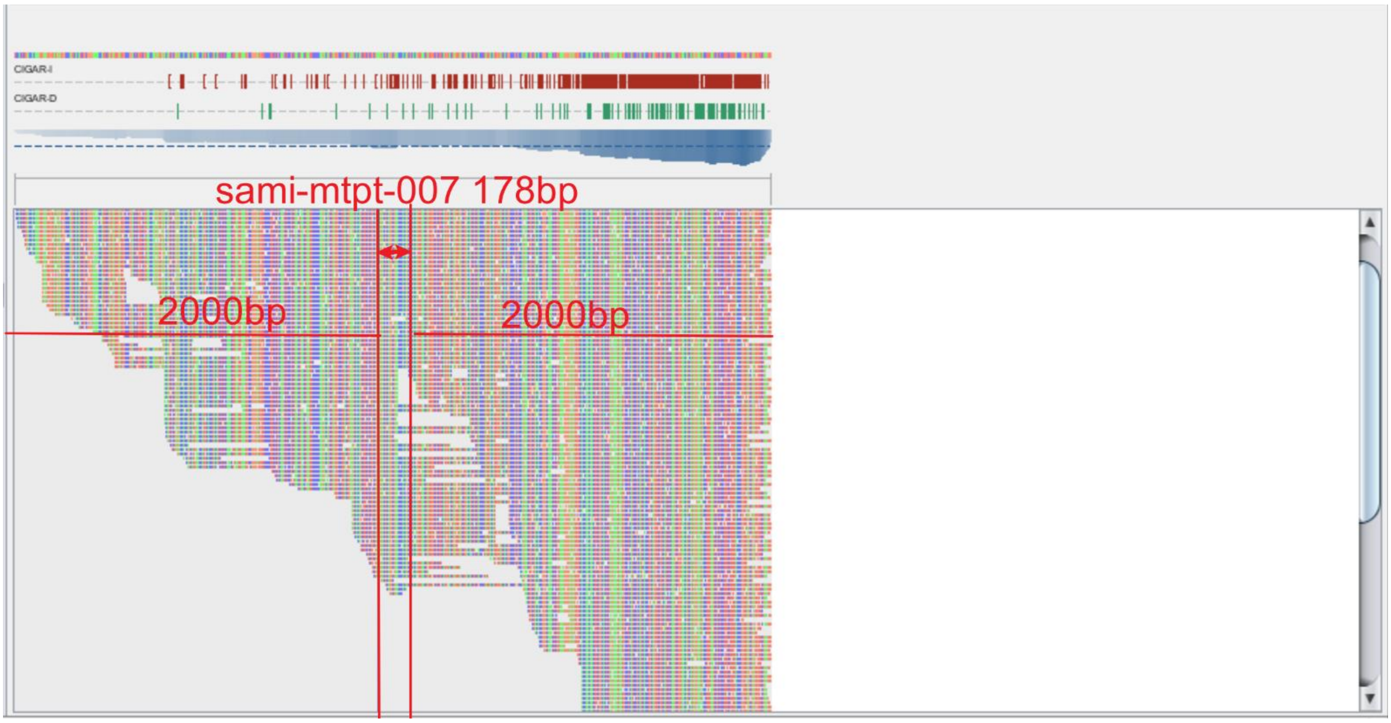

h

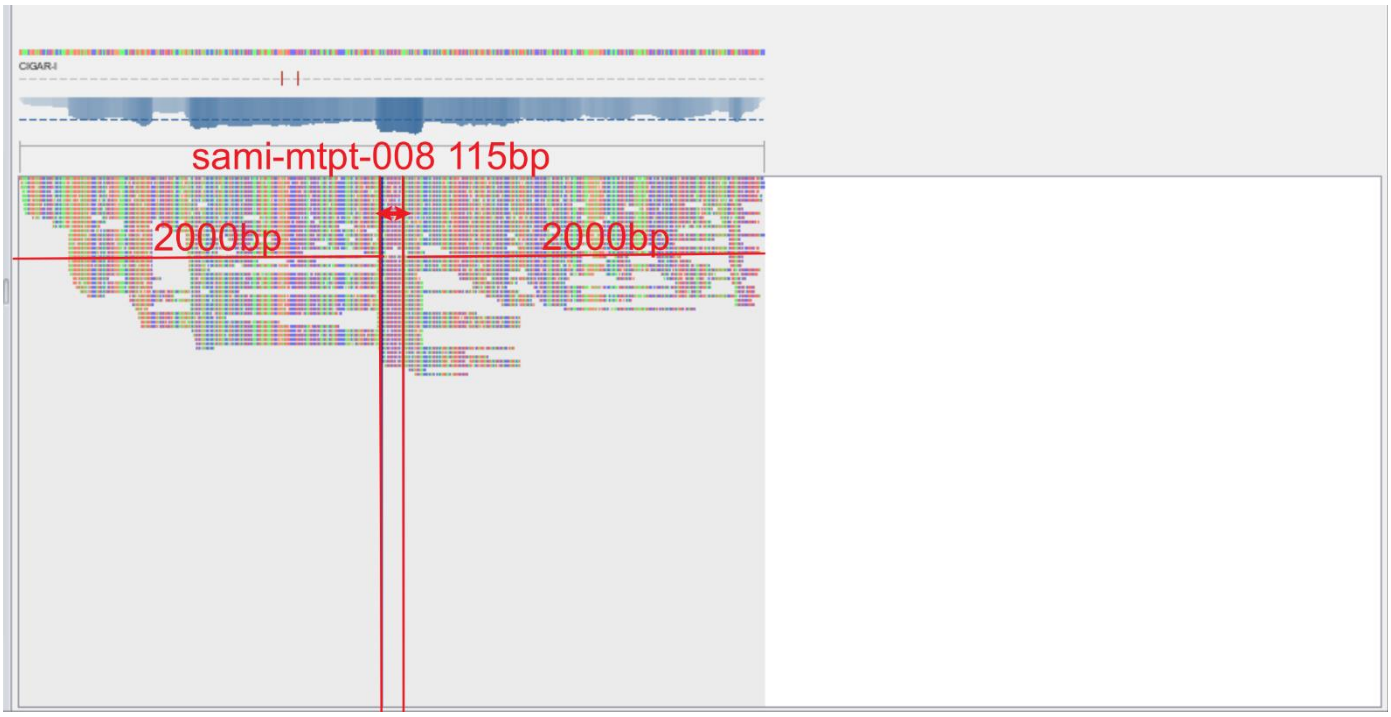

i

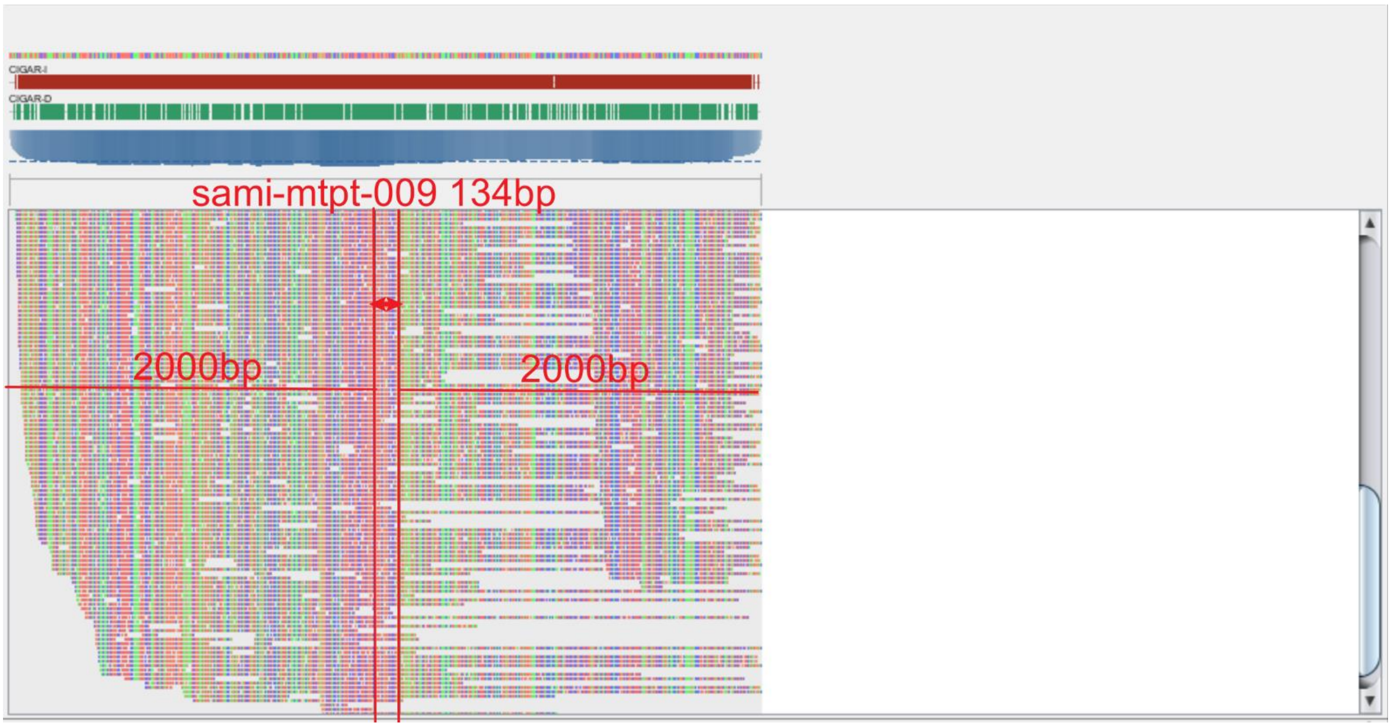

j

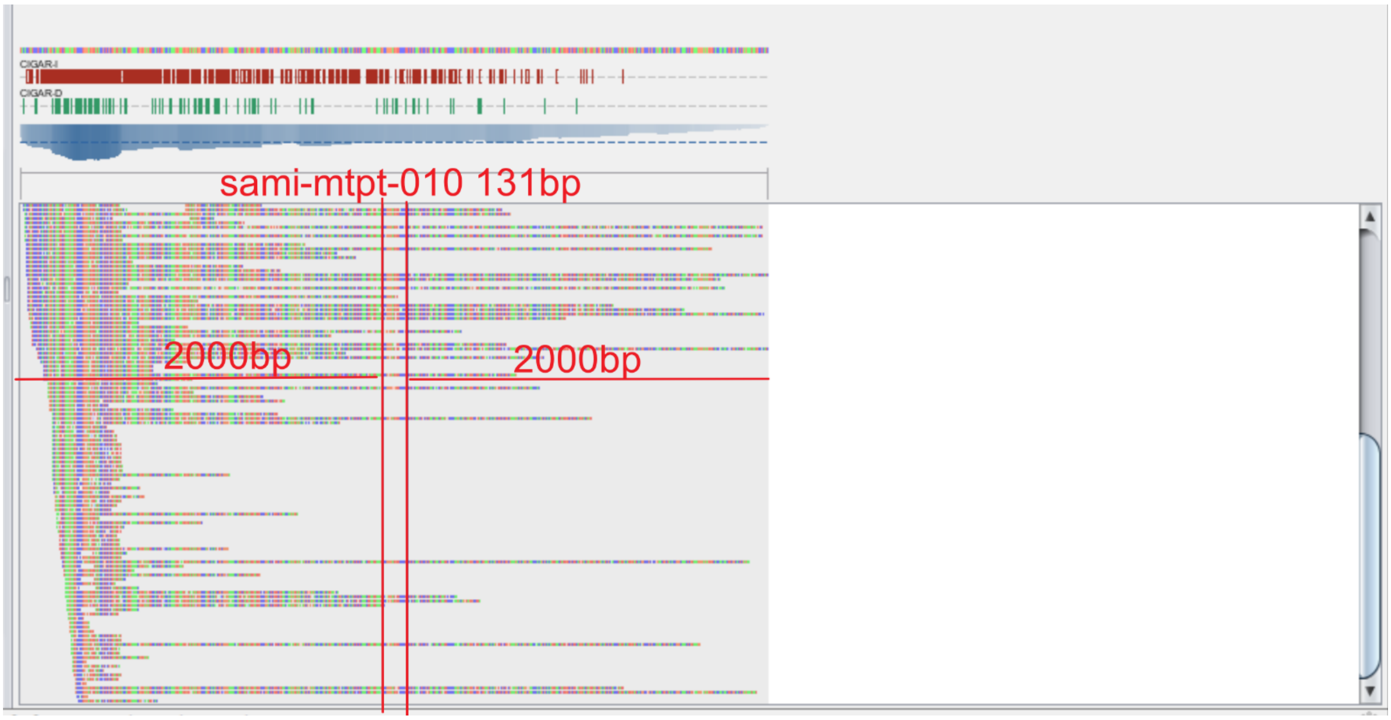

k

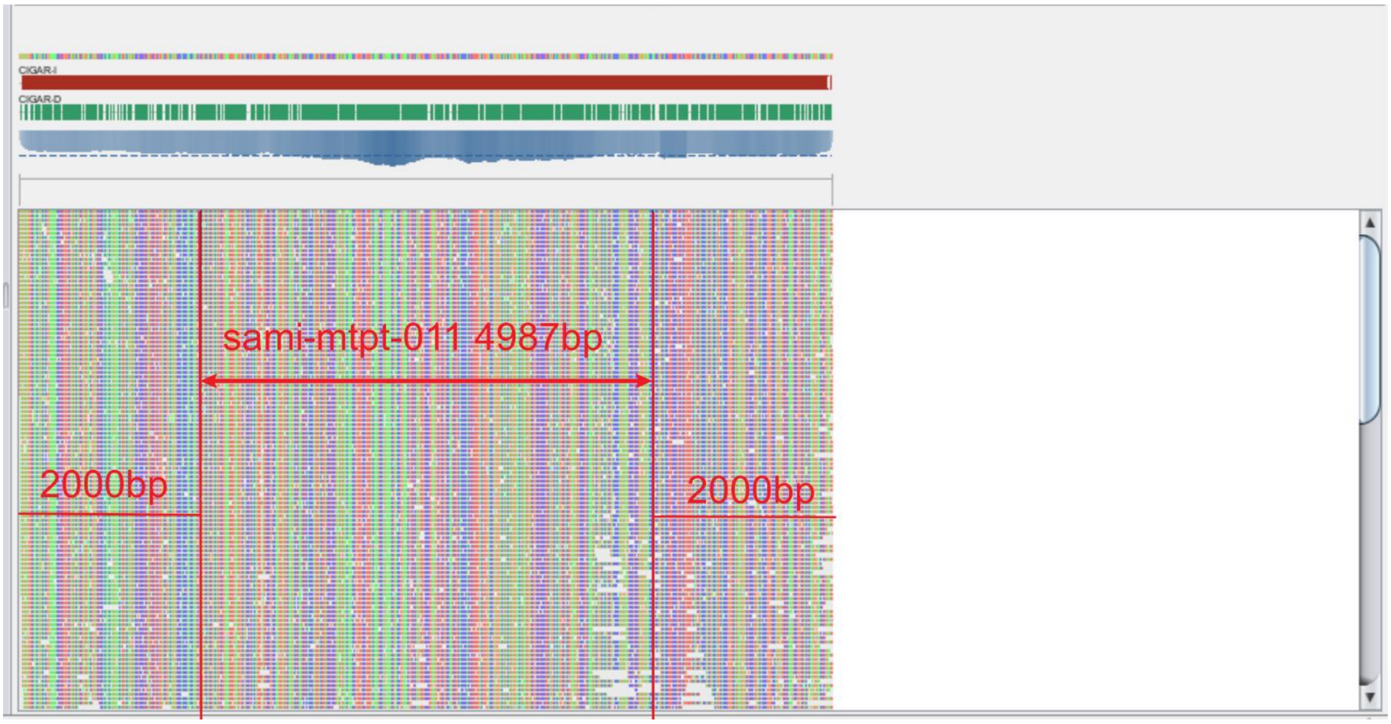

1

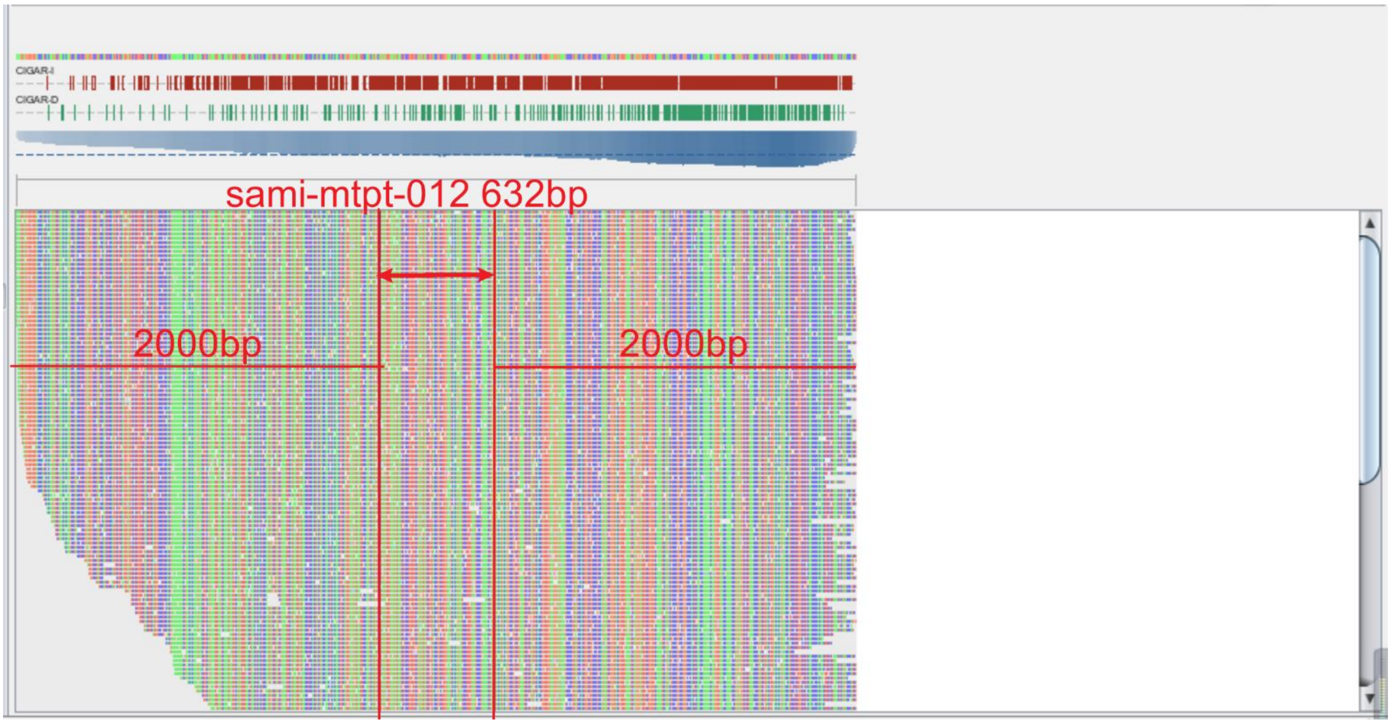

m

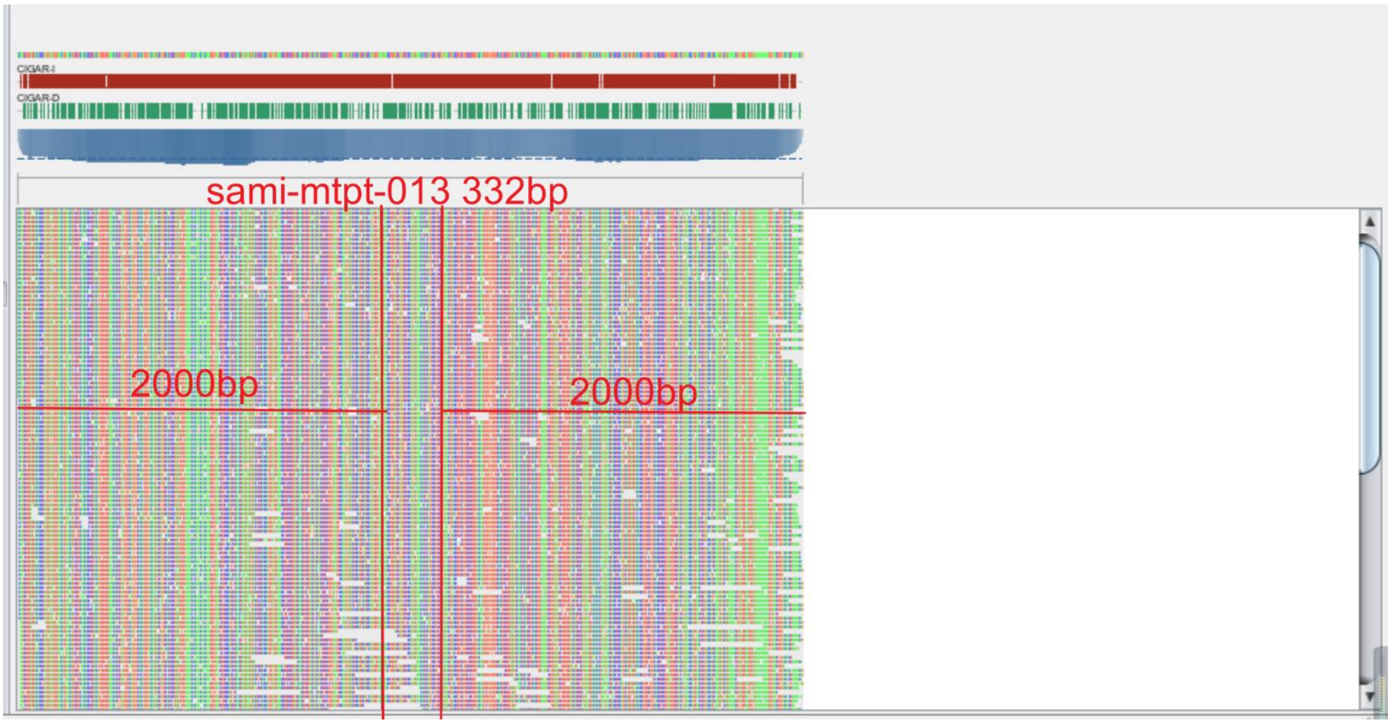

n

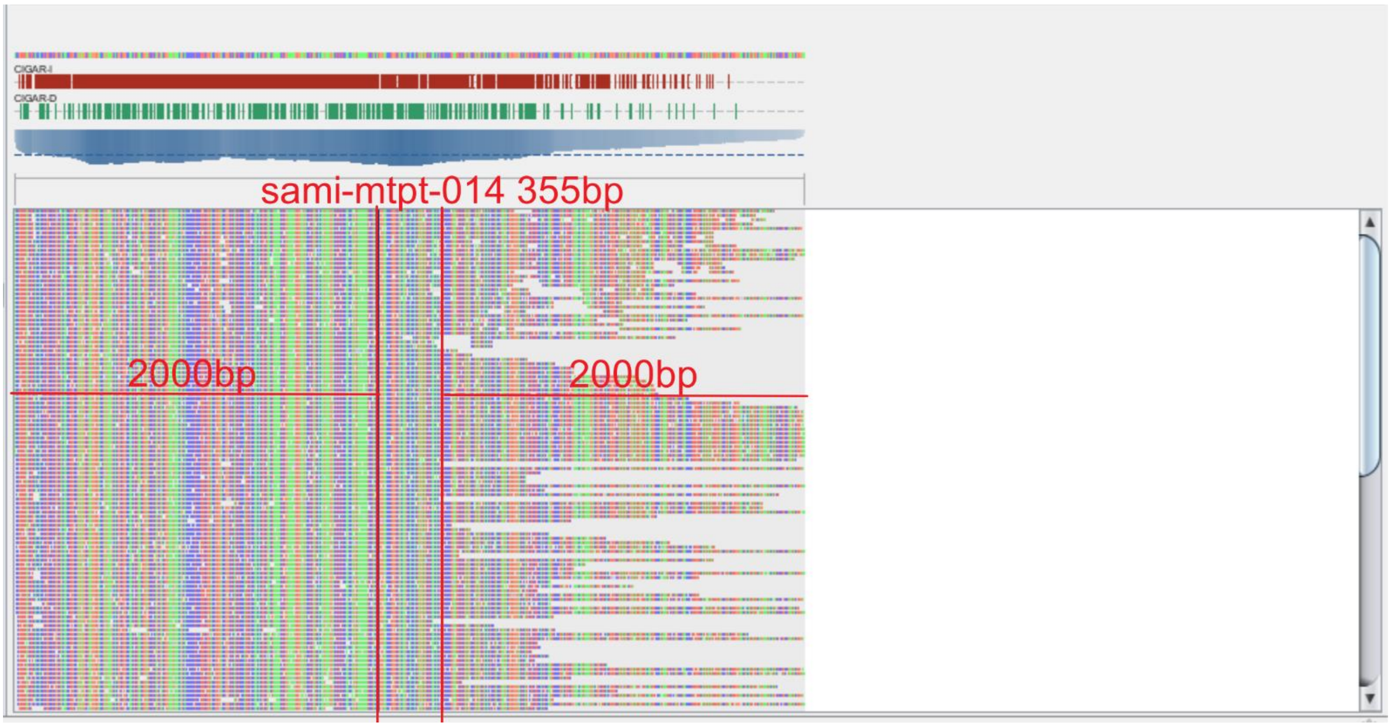

o

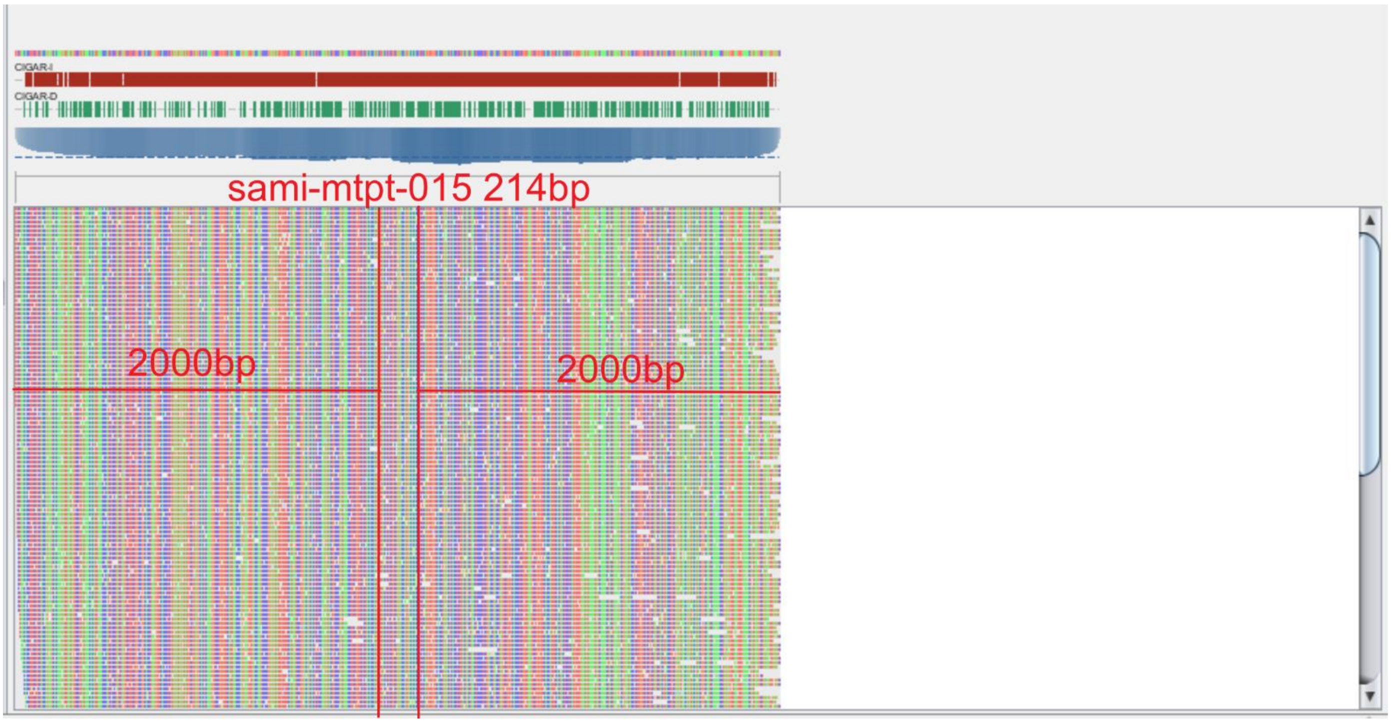

p

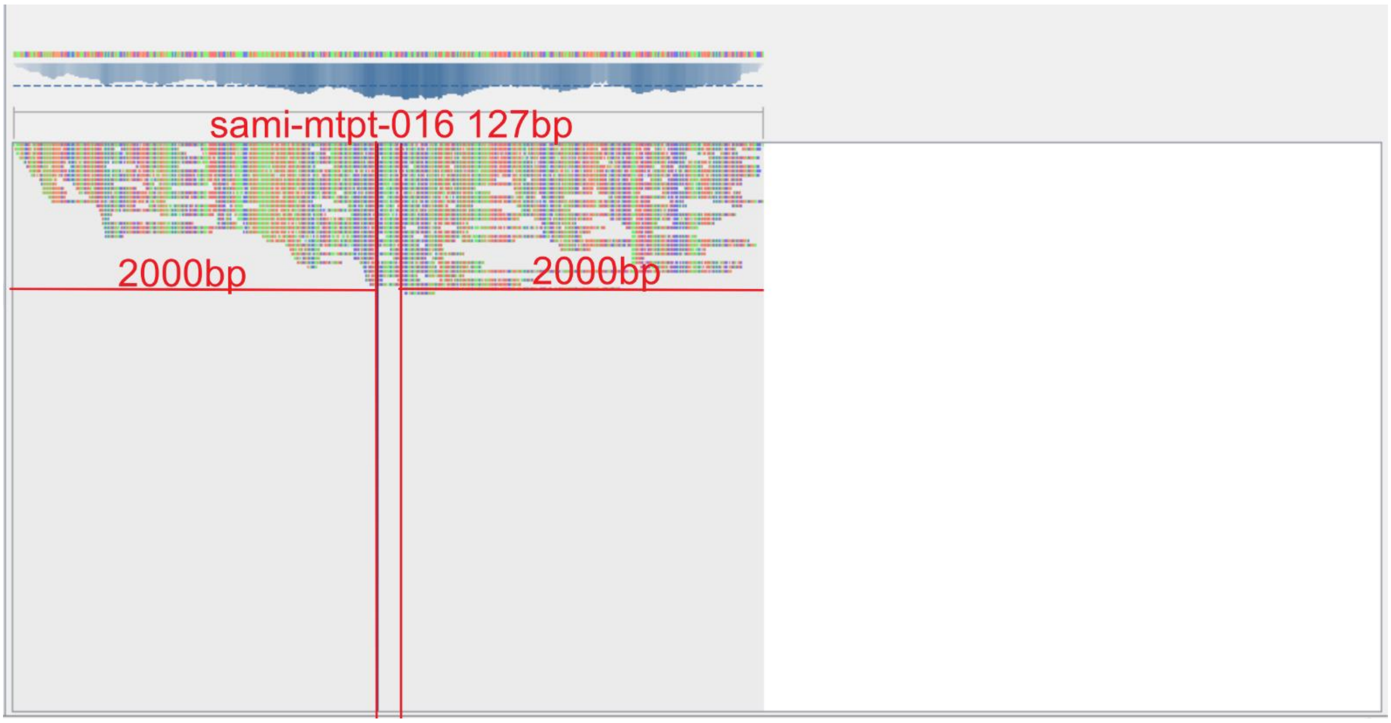

Figure S9. Boxplots of pairwise dN values for mitochondrial genes among the ten Lamiales plants.

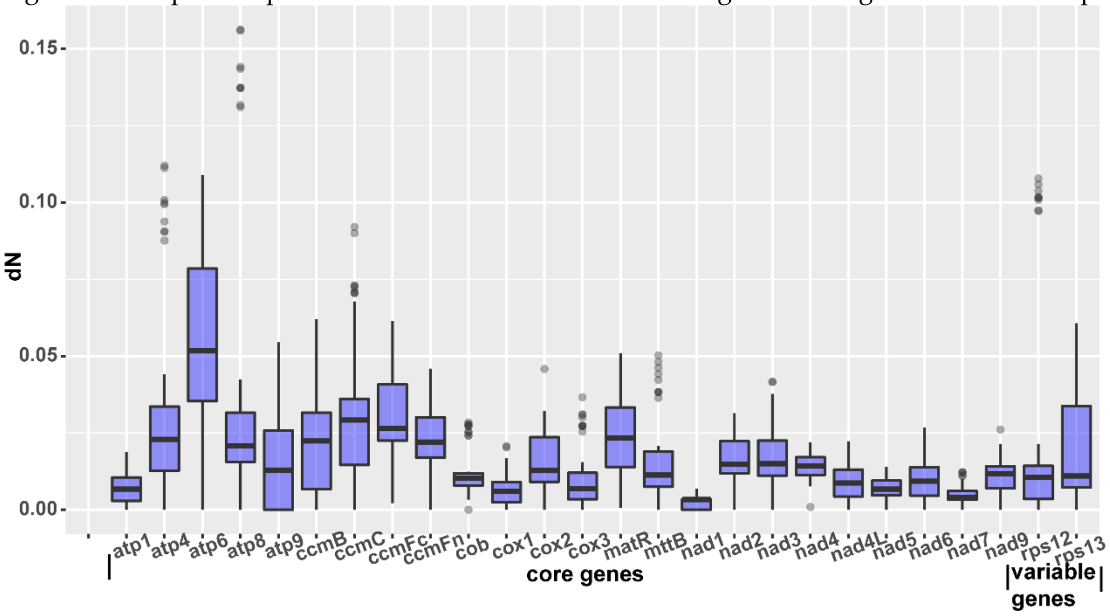

Figure S10. Boxplots of pairwise dS values for mitochondrial genes among the ten Lamiales plants.

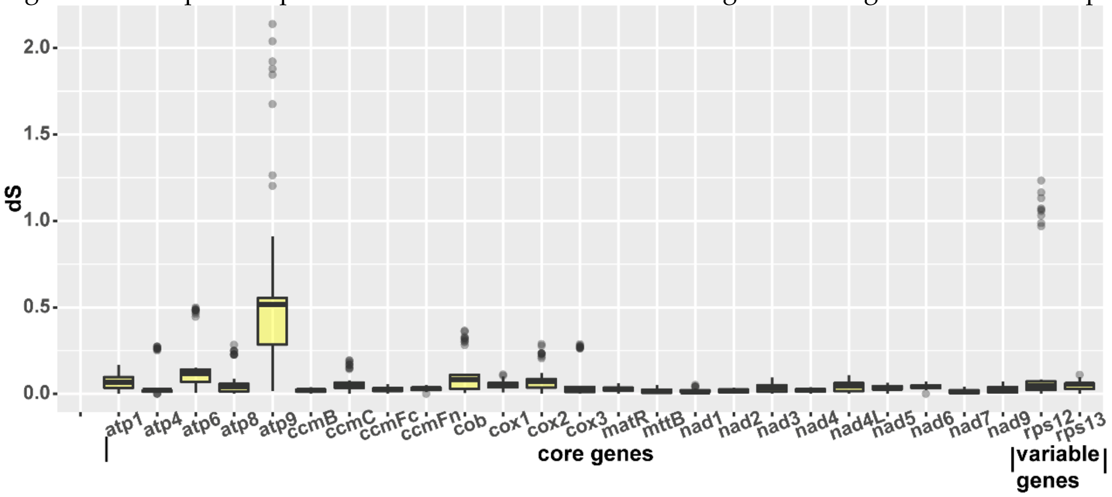

Supplementary File S1. DNA sequence of the plastome of S. miltiorrhiza assembled using the GetOrganelle toolkit.

Supplementary File S2. DNA sequences of the four possible conformations associated with the eleven HSPs (r01-r11) and their corresponding seven DBSs (bs01-bs07) in the mitogenome of S. miltiorrhiza.

Supplementary File S3. The alignment of the sequences of the 26 protein-coding genes of the ten Lamiales mitogenomes and the results of the pairwise dN, dS, and dN/dS values of each two species.
